# Bis(phenylethynyl)arene Linkers in Tetracationic Bis‐triarylborane Chromophores Control Fluorimetric and Raman Sensing of Various DNAs and RNAs

**DOI:** 10.1002/chem.202005141

**Published:** 2021-02-24

**Authors:** Matthias Ferger, Željka Ban, Ivona Krošl, Sanja Tomić, Lena Dietrich, Sabine Lorenzen, Florian Rauch, Daniel Sieh, Alexandra Friedrich, Stefanie Griesbeck, Adriana Kenđel, Snežana Miljanić, Ivo Piantanida, Todd B. Marder

**Affiliations:** ^1^ Institut für Anorganische Chemie and Institute for Sustainable Chemistry & Catalysis with Boron Julius-Maximilians-Universität Würzburg Am Hubland 97074 Würzburg Germany; ^2^ Division of Organic Chemistry & Biochemistry Ruđer Bošković Institute, Bijenička 54 10000 Zagreb Croatia; ^3^ Division of Analytical Chemistry Department of Chemistry, Faculty of Science University of Zagreb, Horvatovac 102a 10000 Zagreb Croatia

**Keywords:** boranes, DNA/RNA sensors, fluorescent probes, molecular modelling, Raman probes

## Abstract

We report four new luminescent tetracationic bis‐triarylborane DNA and RNA sensors that show high binding affinities, in several cases even in the nanomolar range. Three of the compounds contain substituted, highly emissive and structurally flexible bis(2,6‐dimethylphenyl‐4‐ethynyl)arene linkers (**3**: arene=5,5′‐2,2′‐bithiophene; **4**: arene=1,4‐benzene; **5**: arene=9,10‐anthracene) between the two boryl moieties and serve as efficient dual Raman and fluorescence chromophores. The shorter analogue **6** employs 9,10‐anthracene as the linker and demonstrates the importance of an adequate linker length with a certain level of flexibility by exhibiting generally lower binding affinities than **3**–**5**. Pronounced aggregation–deaggregation processes are observed in fluorimetric titration experiments with DNA for compounds **3** and **5**. Molecular modelling of complexes of **5** with AT‐DNA, suggest the minor groove as the dominant binding site for monomeric **5**, but demonstrate that dimers of **5** can also be accommodated. Strong SERS responses for **3**–**5** versus a very weak response for **6**, particularly the strong signals from anthracene itself observed for **5** but not for **6**, demonstrate the importance of triple bonds for strong Raman activity in molecules of this compound class. The energy of the characteristic stretching vibration of the C≡C bonds is significantly dependent on the aromatic moiety between the triple bonds. The insertion of aromatic moieties between two C≡C bonds thus offers an alternative design for dual Raman and fluorescence chromophores, applicable in multiplex biological Raman imaging.

## Graphical abstract

**Aromatic alternative**: Four bis‐triarylborane chromophores with high binding affinities to DNA and RNA are reported. Three of them employ bis(phenylethynyl)arene linkers and are strongly fluorescent and highly Raman active. Fluorimetric titration experiments reveal distinct aggregation–deaggregation processes for two of the compounds, and molecular modelling of complexes of the compounds with DNA illustrates and corroborates the experimental findings.

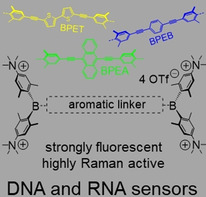

## Introduction

One of the most essential tasks of living organisms is the reproduction of their own genome.^[1]^ This requires the production of proteins from information that is stored in their DNA. The central dogma of molecular biology,^[2]^ which is under ongoing debate,[^3^, ^4^, ^5^] explains this protein production as a one‐way information flow, where DNA is the source of genetic information, DNA sequences are transcribed into RNA and RNA is translated into proteins. Detailed investigation of these bio‐macromolecules and understanding of the interactions that influence their communication on a molecular level is a broad and interdisciplinary research field. One well‐established way to approach the subject is to study the interactions and binding behaviours of small molecules with DNA and RNA.[^6^, ^7^, ^8^, ^9^, ^10^, ^11^, ^12^, ^13^, ^14^, ^15^, ^16^, ^17^]

**Figure 1:**
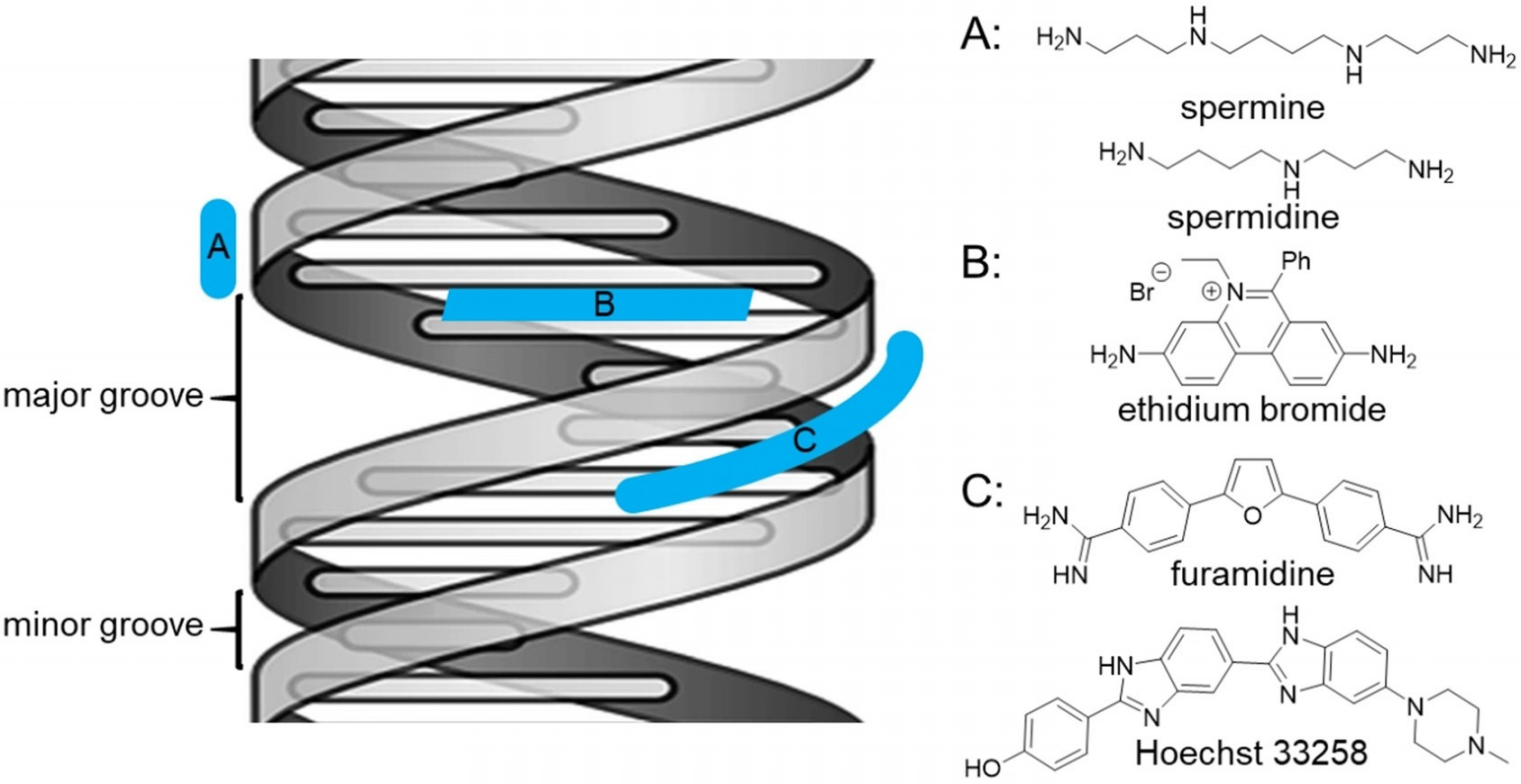
Simplified illustration of the three main binding modes of small molecules (blue) to a helical bio‐macromolecule (grey helical ribbon represents phosphate backbone; grey horizontal sticks represent two interacting nucleobases) and selected examples for each binding mode. A: external binder (polyamines^[18]^); B: intercalator (ethidium bromide^[19]^); C: groove binder (furamidine,^[20]^ Hoechst 33 258^[21]^).

There are three main binding modes, namely intercalation, groove binding and external binding (Figure 1).[^22^, ^23^] For intercalation to occur, the helical structure of the bio‐macromolecule needs to unwind to allow for a small molecule to insert in between the coplanarly arranged nucleobases. Typical intercalators possess rigid and planar polycyclic aromatic moieties, which are required for efficient π–π stacking with the nucleobases.^[24]^ Groove binding can occur into the major or the minor groove. Larger molecules, such as natural and synthetic oligonucleotides and proteins, usually bind in the major groove, while synthetic small molecules prefer the minor groove.^[25]^ Such small molecules are curved and consist of several flexibly connected aromatic moieties.^[26]^ Most groove binders possess functional groups which form hydrogen bonds with the nucleobases of the bio‐macromolecule. Other important driving forces are van der Waals interactions^[27]^ and a significant energy gain when the hydrophobic part of the small molecule is transferred from the aqueous environment into the less polar groove of the bio‐macromolecule, accompanied by a transfer of the respective amount of water molecules from the groove into the aqueous environment.^[28]^ External binding is mostly caused by attractive electrostatic interactions between the negatively charged phosphate backbone and a small molecule. Furthermore, the release of positively charged counterions (from the so‐called ion atmosphere that surrounds charged bio‐macromolecules in solution) provides a positive entropic contribution.[^29^, ^30^, ^31^] Some dye aggregates bind to bio‐macromolecules via external binding, as they are too large to fit into any of the binding sites.[^23^, ^24^, ^32^] It should be noted that more than one binding mode may be relevant to explain the binding event of a small molecule with a bio‐macromolecule, and that relatively small changes in the design of a small molecule can significantly alter the predominant mode of binding.^[33]^

If the binding event significantly changes the structure of the bio‐macromolecule, which usually occurs upon intercalation, but is also possible when the groove has to adjust its size, that is, for the binding of a sterically demanding dye aggregate, it influences their behaviour in biological processes.[^34^, ^35^] Thus, such small molecules have great potential as anticancer, antiviral and anti‐infective drugs, with DNA[^35^, ^36^, ^37^, ^38^, ^39^] as well as RNA[^40^, ^41^, ^42^, ^43^, ^44^] as possible targets. If the binding event significantly changes the properties of the small molecule, these property changes can be monitored to visualize DNA and RNA in vitro and in vivo. Thus, a further, widely studied application of small molecules binding to bio‐macromolecules is DNA and RNA staining in biological imaging.[^45^, ^46^, ^47^, ^48^]

**Figure 2:**
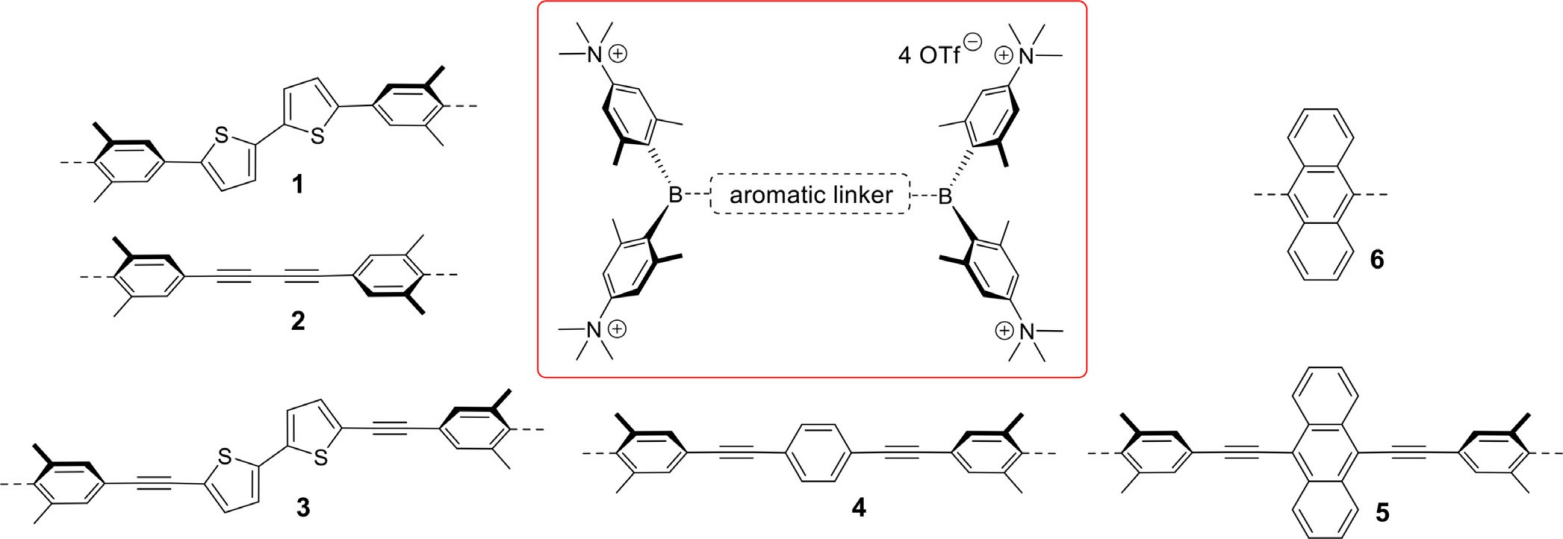
General structure of our novel class of DNA/RNA sensors with different aromatic linkers including the target molecules **3**–**6** of this study.

Within the past few years, several chromophores containing triarylboryl moieties have been shown to be applicable in biological imaging.[^49^, ^50^, ^51^, ^52^, ^53^, ^54^, ^55^, ^56^, ^57^, ^58^, ^59^, ^60^, ^61^, ^62^, ^63^, ^64^, ^65^] Since their first report in the literature 135 years ago,[^66^, ^67^] triarylboranes have found applications in many different fields, such as metal–organic framework (MOF) chemistry, anion sensing and optoelectronics.[^68^, ^69^, ^70^] The three‐coordinate boron in a triarylboryl moiety serves as a strong π‐acceptor and as a strong Lewis acid, due to its vacant p_z_ orbital. When employing triarylboranes in functional materials, bulky substituents have to be employed to stabilize the three‐coordinate boron against decomposition by air and moisture.[^71^, ^72^, ^73^, ^74^, ^75^, ^76^, ^77^, ^78^, ^79^, ^80^, ^81^, ^82^, ^83^, ^84^, ^85^, ^86^] Using Gabbaï’s approach,^[87]^ we recently developed compound **1** (Figure 2) as a water‐stable, water‐soluble and non‐cytotoxic live cell imaging agent.^[53]^

In further studies, it was found that **1** serves as an efficient sensor for DNA, RNA and protein.^[88]^ The fluorescence emission from **1** was found to increase upon addition of all bio‐macromolecules which were examined. However, emission maxima were strongly dependent on the type of bio‐macromolecule (DNA/RNA vs. protein), which allowed us to suggest a protein‐like binding site for **1** in the cell. In contrast, the emission of buta‐1,3‐diyne analogue **2** (Figure 2) was strongly quenched by DNA, RNA and protein.^[89]^ Thus, it was indicated that the linker connecting the two triarylborane moieties in tetracationic bis‐triarylboranes has a profound impact on its fluorescence response when sensing bio‐macromolecules. In contrast to **1**, compound **2** can be applied as a combined fluorimetric and Raman probe for simultaneous and selective sensing of various DNA, RNA and proteins, due to its strong Raman signal. Dual Raman and fluorescence spectroscopy has attracted increasing interest in recent years, as a method that circumvents some of the intrinsic problems of Raman spectroscopy, that is, long acquisition times and low signal strengths.[^90^, ^91^, ^92^] This multimodal approach, has been successfully used in cell imaging,[^93^, ^94^] disease diagnostics[^95^, ^96^, ^97^, ^98^] and monitoring drug delivery.[^99^, ^100^] As fluorescence can adversely interfere with Raman measurements by causing significant background noise,^[101]^ the design of suitable small molecules for the specific purpose of dual Raman and fluorescence imaging[^102^, ^103^] is a rather novel approach and remains challenging. Extended conjugated poly‐ynes have been employed to shift the energy of the Raman active stretching of the C≡C bonds systematically, thus allowing selective labelling and multiplex imaging.^[104]^ The question remained whether these multiple triple bonds need to be directly conjugated to gain the selectivity, or if it is possible to insert various aromatics in between the triple bonds possibly gaining additional selectivity, while maintaining the high Raman signal intensity. This is an important question, as current research in bio‐applicable Raman‐based sensing[^105^, ^106^] requires a broad choice of dyes, preferably ones suitable for dual Raman and fluorescent operation modes. Therefore, we synthesized several dual Raman and fluorescent chromophores, following the basic design of compounds **1** and **2**, thus extending our novel class of bio‐macromolecule sensors (Figure 2).

In our design of the new compounds, we combined the favourable fluorescence increase upon binding to DNA and RNA observed for the bithiophene **1** with the two triple bonds, responsible for the strong Raman signal of compound **2**, which led to compound **3** (Figure 2), resembling 2,5‐bis(phenylethynyl)thiophene (BPET) chromophores investigated by our group and others.[^107^, ^108^] For comparison, a reference compound in the form of the 1,4‐phenylene analogue **4** was prepared, a bis(phenylethynyl)benzene (BPEB) derivative[^109^, ^110^, ^111^, ^112^, ^113^, ^114^, ^115^] in which the overall length of the linker is somewhat shorter than in compound **3**. The bis(phenylethynyl)anthracene (BPEA) derivative **5**, was chosen because BPEAs are strong fluorophores[^116^, ^117^, ^118^, ^119^, ^120^, ^121^, ^122^, ^123^] and the long axis of anthracene in compound **5** is perpendicular to the bis‐triarylborane longitudinal axis. Such an orientation of a large and rigid aromatic moiety is expected to have a significant effect on the nature of the binding interactions with DNA/RNA. The much shorter anthracene analogue **6** prepared previously,^[60]^ which showed intriguing live cell imaging properties was studied to test the importance of linker length and rigidity between the two triarylborane units.

## Results and Discussion

### Synthesis and solid‐state structures

**Scheme 1:**
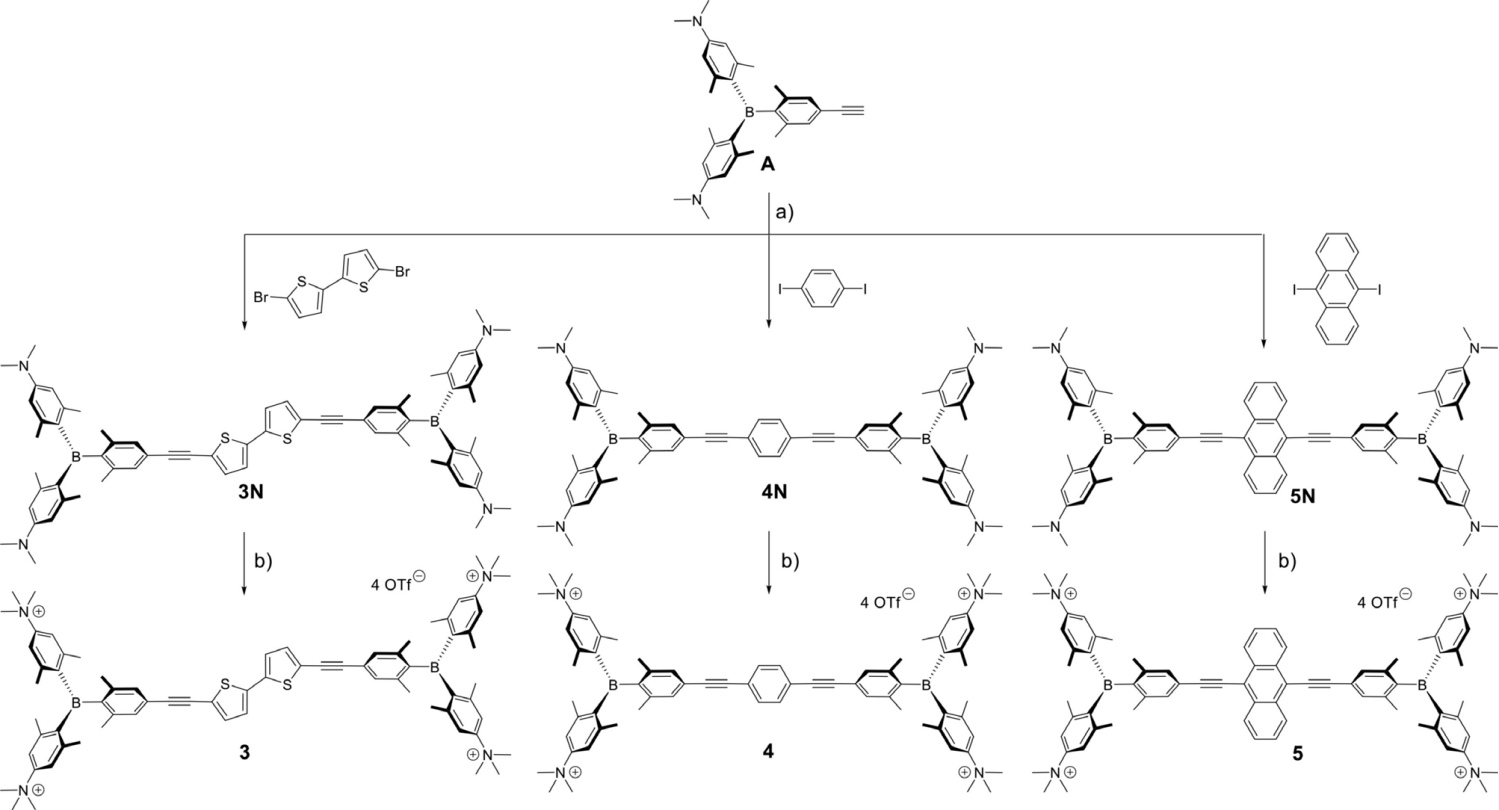
Synthesis of the compounds **3N**, **4N**, **5N** and **3**, **4**, **5**. a) Pd(PPh_3_)_2_Cl_2_, CuI. THF/NEt_3_, RT; b) MeOTf, CH_2_Cl_2_, RT.

Starting material **A** was synthesized according to the procedure previously reported by our group.^[89]^ The bisethynyl arenes **3N**, **4N** and **5N** were prepared via Sonogashira coupling reactions of **A** with the respective aryl halides using Pd(PPh_3_)_2_Cl_2_ and CuI as the catalytic system and NEt_3_ in THF as the base (Scheme 1). The neutral compounds **3** **N**, **4** **N** and **5** **N** were methylated at the amine groups with MeOTf in dichloromethane, to afford the tetracationic species **3**, **4** and **5**.

**Figure 3:**
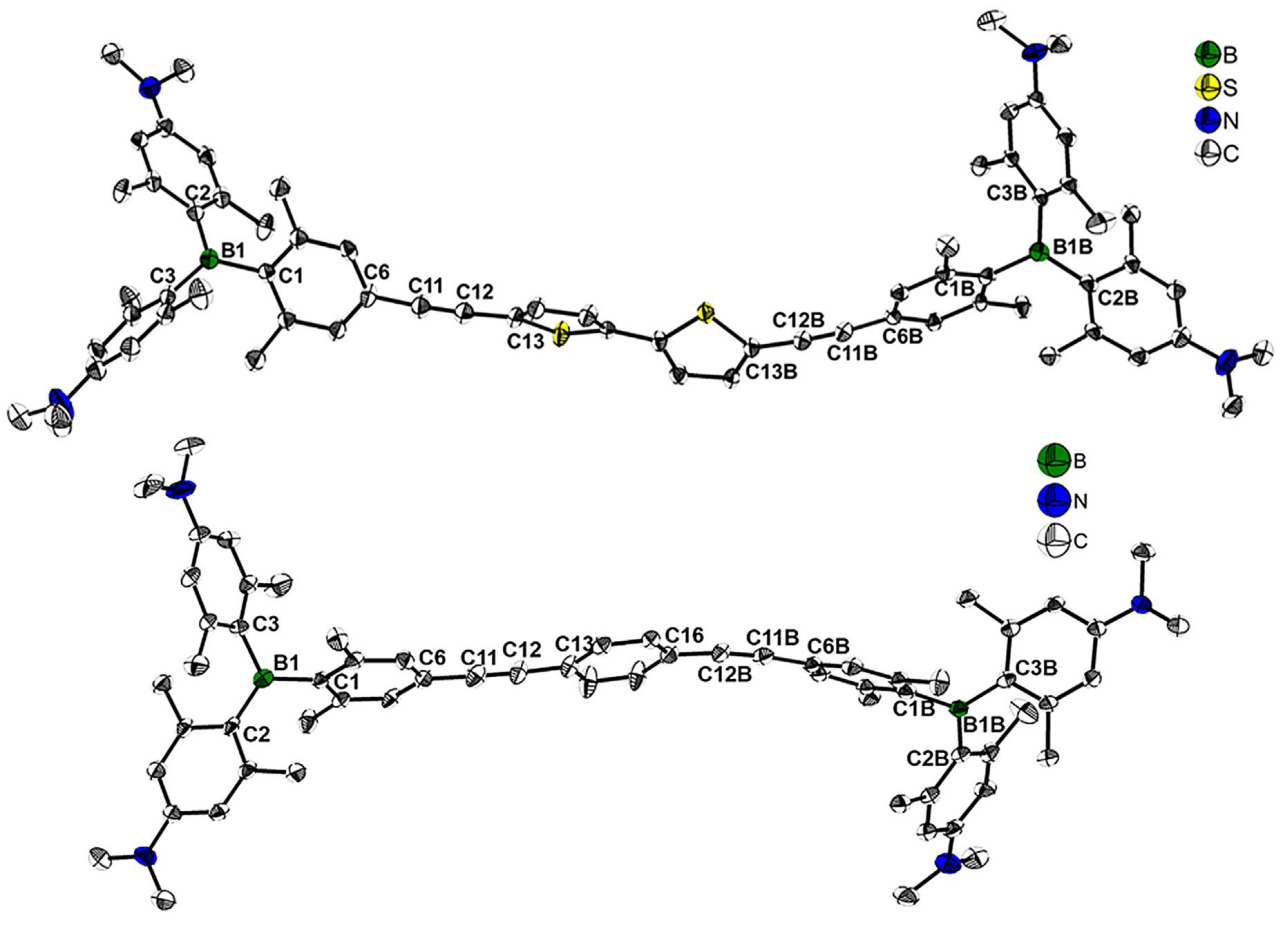
Molecular structures of **3N** (top) and **4N** (bottom) in the solid state at 100 K. Atomic displacement ellipsoids are drawn at the 50 % probability level, and H atoms and co‐crystallized solvent molecules (EtOAc) are omitted for clarity. Selected distances and angles for **3N**: B1−B1B 22.471(12) Å, B1−C1 1.600(5) Å, B1B−C1B 1.592(5) Å, B1−C2 1.569(6) Å, B1B−C2B 1.573(5) Å, B1−C3 1.567(6) Å, B1B−C3B 1.551(6) Å, C6−C11 1.449(5) Å, C6B−C11B 1.437(5) Å, C11−C12 1.191(5) Å, C11B−C12B 1.190(5) Å, C12−C13 1.429(5) Å, C12B−C13B 1.421(5) Å, C1−B1−C2 120.8(3)°, C1B−B1B−C2B 119.6(3)°, C1−B1−C3 118.4(3)°, C1B−B1B−C3B 119.5(3)°, C2−B1−C3 120.7(3)°, C2B−B1B−C3B 120.9(3)°, C6−C11−C12 175.7(5)°, C6B−C11B−C12B 174.7(4)°, C11−C12−C13 175.7(4)°, C11B−C12B−C13B 175.5(4)°, BC_3_−aryl (C1) 55.10(14)°, BC_3_−aryl (C2) 42.53(14)°, BC_3_−aryl (C3) 51.82(14)°, BC_3_−aryl (C1B) 48.54(13)°, BC_3_−aryl (C2B) 51.66(13)°, BC_3_−aryl (C3B) 43.71(13)°. Selected distances and angles for **4N**: B1−B1B 19.525(14) Å, B1−C1 1.592(7) Å, B1B−C1B 1.593(6) Å, B1−C2 1.568(7) Å, B1B−C2B 1.570(6) Å, B1−C3 1.562(6) Å, B1B−C3B 1.561(7) Å, C6−C11 1.436(7) Å, C6B−C11B 1.432(6) Å, C11−C12 1.199(6) Å, C11B−C12B 1.203(6) Å, C12−C13 1.433(7) Å, C12B−C16 1.432(6) Å, C1−B1−C2 121.0(4)°, C1B−B1B−C2B 118.5(4)°, C1−B1−C3 118.0(4)°, C1B−B1B−C3B 120.4(3)°, C2−B1−C3 120.9(4)°, C2B−B1B−C3B 121.0(4)°, C6−C11−C12 178.2(5)°, C6B−C11B−C12B 173.9(4)°, C11−C12−C13 177.8(5)°, C11B−C12B−C16 173.2(4)°, BC_3_−aryl (C1) 55.25(13)°, BC_3_−aryl (C2) 43.82(13)°, BC_3_−aryl (C3) 49.94(14)°, BC_3_−aryl (C1B) 47.56(13)°, BC_3_−aryl (C2B) 42.21(13)°, BC_3_−aryl (C3B) 52.71(13)°.

Single crystals of **3** **N** and **4** **N** suitable for X‐ray diffraction analysis were obtained. Even though the following investigations on interactions with DNA and RNA were performed with the tetracationic analogues, the solid‐state molecular structures of the neutral precursors are given in Figure 3, to provide an indication of the size and shape of this class of compounds. The B1−B1B distance is 22.471(12) Å for **3N** and 19.525(14) Å for **4N**. The linkers in both structures are slightly curved, which reveals a degree of flexibility for both compounds as is often the case for alkynyl systems. This is in accordance with the respective C6−C11−C12, C6B−C11B−C12B, C11−C12−C13, C11B−C12B−C13B and C16−C12B−C11B angles, which differ slightly from 180° in all cases (Figure 3). The evident flexibility in the solid state suggests at least a similar flexibility in solution, which was further corroborated by our binding experiments with DNA and RNA (vide infra). Single crystals suitable for X‐ray diffraction analysis for the trimethylsilyl‐protected precursor to compound **A** were obtained. The solid‐state molecular structure of **C** ^[89]^ is reported in Table S1 and Figure S13 in the Supporting Information, and the geometry of **C** does not exhibit a significant deviation from those of the triarylborane groups in **3N** and **4N**.

### Physicochemical properties

All of our positively charged compounds (**3**–**6**) were found to be moderately soluble in water (*c=*1×10^−4^ m) and, when stored in the dark, their aqueous solutions were stable for months. Photophysical data for the novel compounds **3**–**5** in acetonitrile and water are summarized in Table 1, while the photophysical data for **6** are reported in our previous publication.^[60]^ Additionally, computational studies on the compounds **3**–**5** were carried out. Results of those studies and a short discussion can be found in the Supporting Information.

**Table 1:** Photophysical data for compounds **3**–**5** in acetonitrile and water.

	Solvent	*λ*_abs_ [nm]	*ϵ* [m ^−1^ cm^−1^]	*λ*_em_ [nm]	Stoke's shift [cm^−1^]	*Φ*_f_	*τ* [ns]	*k*_r_ [10^8^ s^−1^]	*k*_nr_ [10^8^ s^−1^]
**3**	MeCN	413	65 000	535	5 500	0.31	1.27	2.4	5.4
	H_2_O	413	62 000	558	6 300	0.26	<1	–	–
**4**	MeCN	373	60 000	452	4 700	0.58	3.27	1.8	1.3
	H_2_O	371	62 000	452	4 800	0.73	3.63	2.0	0.7
**5**	MeCN	483	52 000	505	900	0.72	2.45	2.9	1.1
	H_2_O^[a]^	485	47 000	501	700	0.72	2.33	3.1	1.2

[a] At concentrations >1×10^−5^ m, compound **5** begins to aggregate. This results in a gradual shift of the emission maximum towards *λ*_em_=578 nm (Figure S18).

**Figure 4:**
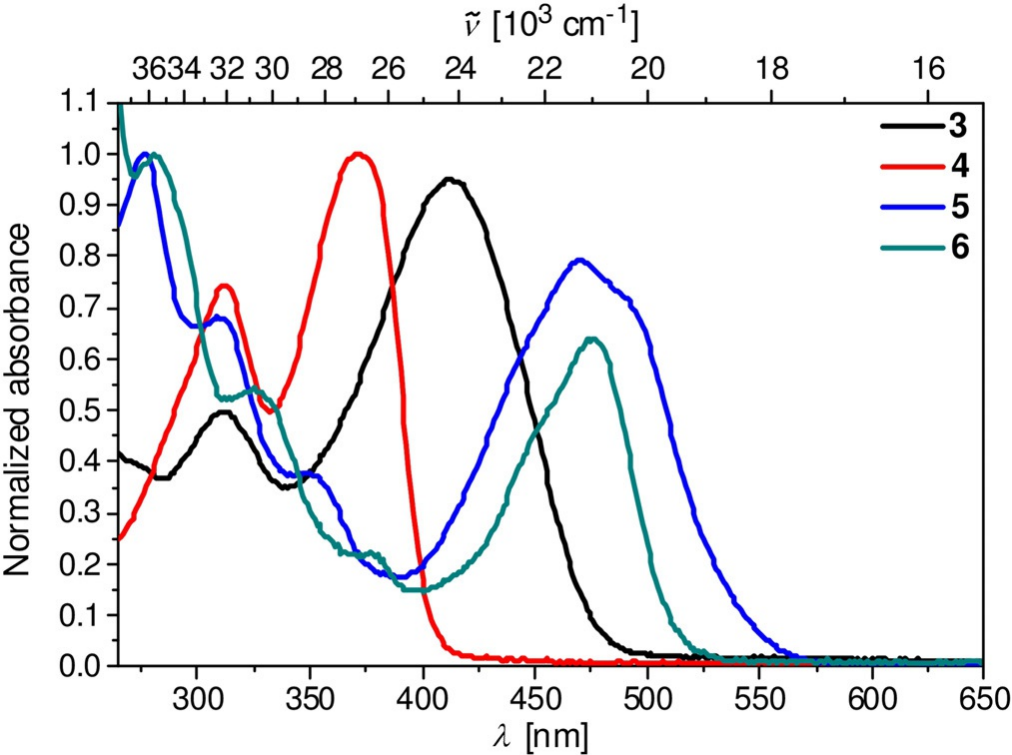
UV/Vis spectra of compounds **3**–**6** at *c=*1×10^−6^ m in sodium cacodylate buffer solution at pH 7, *I*=0.05 M.

The UV/Vis spectra of the aqueous sodium cacodylate buffer solutions (Figure 4, pH 7) of the compounds studied were proportional to their concentration in the *c=*5–15×10^−6^ m range, and the corresponding absorption maxima and molar extinction coefficients are listed in the Supporting Information (Table S3).

**Figure 5:**
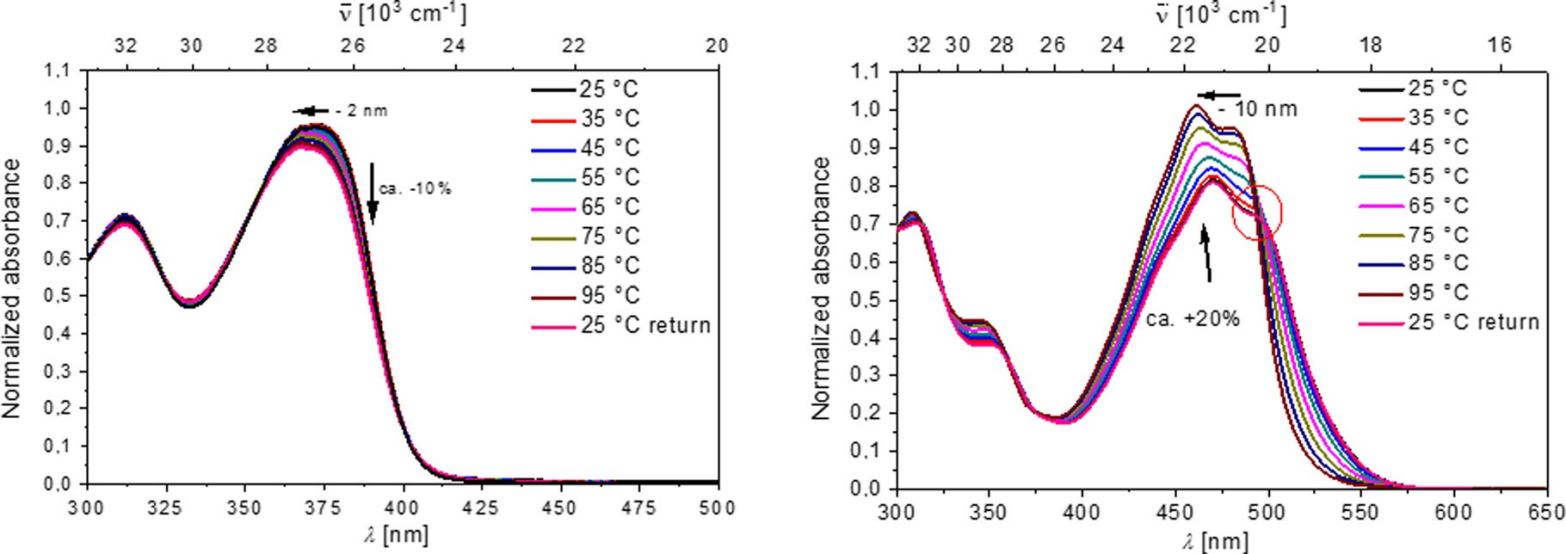
Temperature dependence at *c=*1.3–1.7×10^−5^ m in buffered solution at pH 7, *I*=0.05 M. Left: hypochromic effect in the absorption spectra of **4** (>10 %); right: hyperchromic effect (+20 %) in the absorption spectra of **5**, suggesting that **5** is aromatically stacked; the red circle indicates the absence of an isosbestic point.

Upon heating the solutions, the UV/Vis spectra of **3**–**6** exhibited quite different properties. While the UV/Vis spectra of **4** and **6** showed only a negligible decrease with temperature (Figure 5, left, and Figure S23) the spectra of **3** and **5** changed significantly, showing a strong increase in absorbance and a pronounced hypsochromic shift (Figure 5, right, and Figure S23). In all cases, the spectral changes were fully reversible upon cooling back to 25 °C, thus suggesting that no chemical changes took place.

**Figure 6:**
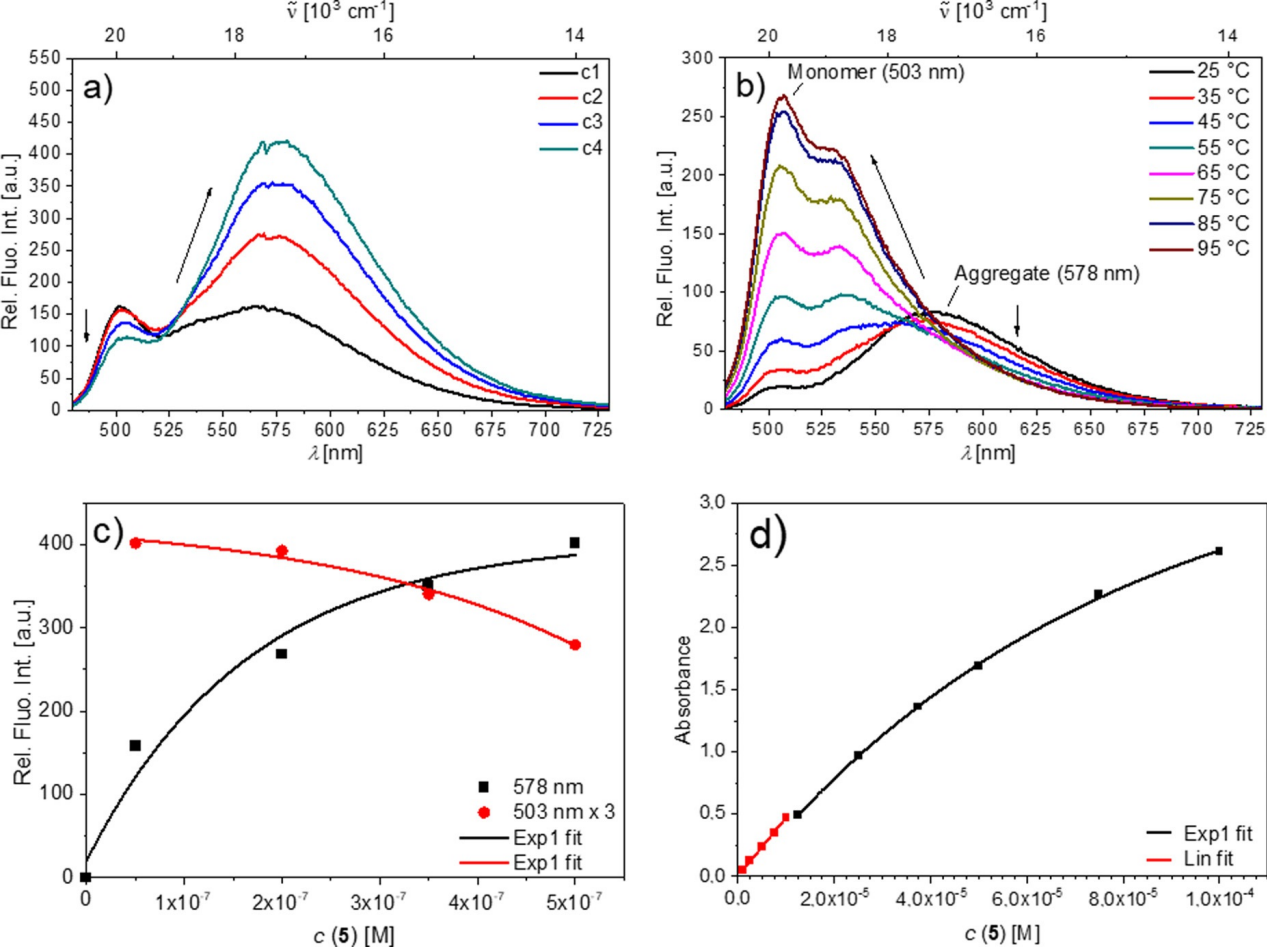
a) Fluorescence spectra of **5** (*λ*_ex_=470 nm) in sodium cacodylate buffer (pH 7.0, *I*=0.05 m) at *c*(**5**)=5, 20, 35, and 50×10^−8^ m. b) Temperature dependence of emission *c*(**5**)=50×10^−8^ m in sodium cacodylate buffer (pH 7.0, *I*=0.05 m). c) Concentration dependence of the emission of **5** (*λ*_ex_=470 nm) in sodium cacodylate buffer (pH 7.0, *I*=0.05 m) at 578 and 503 nm (multiplied by 3 for improved visibility); note the good fit of the experimental points to a first exponential. d) Concentration dependence of the absorbance of **5** in pure water, with a linear fit for *c*(**5**)<1×10^−5^ m and first‐exponential fit for *c*(**5**)>1×10^−5^ m.

Aqueous sodium cacodylate solutions of compounds **3**–**6** were strongly fluorescent (Figures S24–S26); however, their emission properties differed remarkably. The 1,4‐diethynylbenzene‐derivative **4** and the shortest 9,10‐anthraceneylene compound **6** were characterized by emission intensities proportional to compound concentrations up to 5×10^−7^ m (**4**) and 8×10^−6^ m (**6**), respectively, and their spectra (Figures S24 and S25) revealed only minor changes upon heating to 95 °C, thus suggesting the absence of intermolecular interactions. In contrast, emission from the 9,10‐diethynylanthracene analogue **5** was strongly non‐proportional to concentration, even at *c*<1×10^−7^ m (Figure 6 c), and for both, **3** (Figure S26) and **5** (Figure 6 b), the emission spectra at 5×10^−7^ m changed significantly with increasing temperature.

This temperature dependence of fluorescence and UV/Vis spectra of **3** and **5** strongly support intermolecular noncovalent aromatic stacking interactions between the chromophores. Particularly interesting is the emission spectrum of **5** (Figure 6), which is characterized by two distinct maxima: 503 and 578 nm. The ratio of the intensities of the maxima (*r*=*I*_503_/*I*_578_) changes strongly and nonlinearly with concentration and temperature. The maximum at 503 nm can be attributed[^116^, ^124^, ^125^, ^126^] to individual, nonstacked molecule **5**, whereas the maximum at 578 nm corresponds to aggregate emission.^[127a]^ The emission changes plotted against the concentration of **5** fit well to a first exponential (Figure 6 c), suggesting a well‐defined, one‐type aggregation process.

In pure water, the aggregation processes appear to be significantly less favourable. For compound **3**, a linear dependence of the absorbance on the concentration was found throughout the whole measurable range from 1×10^−6^ to 3.75×10^−5^ m (Figure S16). Only in the case of compound **5** was a nonlinear dependence of the absorbance on the concentration found for concentrations >1×10^−5^ m (Figure 6 d). The emission of **5** in pure water changes accordingly (Figure S18). Between 1×10^−6^ and 1×10^−5^ m, only emission from the monomer is detectable. At concentrations >1×10^−5^ m, the emission maximum gradually shifts bathochromically and at 1×10^−4^ m, only aggregate emission with a maximum at 578 nm is detectable.

Thus, in sodium cacodylate buffer solution, aggregates are predominant for compounds **3** and **5** even at concentrations below 5×10^−7^ m (Figures 6 and S21), whereas in pure water, aggregates were only observed for compound **5** at concentrations higher than 1×10^−5^ m (Figures S18 and 6 d). The tendency of our compounds to form aggregates is, therefore, strongly dependent on the ionic strength of the solution, a phenomenon previously observed^[127b]^ and is in the order **5**>**3**≫**4**, **6**.

### Study of interactions with DNA and RNA

We have chosen several typical types of DNA and RNA to investigate the interaction of our compounds **3**–**6** with those macromolecules (Table S4). Calf thymus (ct)‐DNA, which is naturally occurring, represents a typical B‐helix structure with a balanced ratio of GC (48 %) and AT (52 %) base pairs. The synthetic alternating polynucleotides poly (dGdC)_2_ and poly (dAdT)_2_, consist of only GC or AT base pairs. Thus, they represent two extreme situations with very different secondary structures and a very different level of minor groove availability for binding of a small molecule. The sterically demanding guanine amino group, for example, hinders the deep penetration of small molecules. We chose double‐stranded (ds) poly(rA)–poly(rU) RNA, as an A‐helical structure with a major groove that is generally available^[128]^ for binding of bulky small molecules, for comparison between dsDNA and dsRNA.

To understand better the DNA/RNA binding of our novel chromophores, the single‐stranded (ss) synthetic RNA polynucleotides poly(rG), poly(rA), poly(rU) and poly(rC), which are each characterized by different properties, were also investigated. Poly(rG) is related to guanine‐rich sequences in both DNA and RNA. Adenine ssRNA (poly(rA)) mimics 50 to 250 adenine nucleotides at the 3′‐end of mRNA. Poly(rC) and poly(rU) represent less organized secondary structures and are significantly more flexible than purine RNAs. Also, we studied ssDNA poly(dA) and poly(dT), which are analogous to the afore‐mentioned ssRNA.

### Thermal denaturation experiments

Thermal denaturation, which is the dissociation of ds‐helices of polynucleotides into two single‐stranded polynucleotides, occurs at characteristic and well‐defined temperatures (*T*_m_ value). The thermal stability of ds‐helices is generally increased upon noncovalent binding of small molecules to ds‐polynucleotides. This causes a significant increase of the *T*_m_ value (Δ*T*_m_), which can be indicative of various binding modes.^[129]^

The three compounds with longer linkers (**3**–**5**) all strongly stabilized the dsDNA/RNA even at rather low ratios of compound to polynucleotide (in the following, this ratio is generally given as: *r*_[compound]/[polynucleotide]_; Figures S27–S35), with anthracene analogue **5** causing the strongest stabilization (Table 2). Our previously reported bithiophene compound **1** ^[88]^ and buta‐1,3‐diyne compound **2** ^[89]^ stabilized DNA and RNA to a similar extent (Δ*T*_m_=7–10 °C). Intriguingly, the short anthracene compound **6** had a very weak effect on the thermal stability of DNA/RNA (Figures S36 and S37). These findings suggest that once a certain length and level of flexibility is exceeded, the nature of the aromatic linker in our tetracationic bis‐triarylboranes does not strongly influence the thermal stabilization effect of those compounds.

**Table 2:** The Δ*T*_m_ values^[a]^ of the ds‐polynucleotides studied upon addition of compounds **3**–**6** at pH 7.0 (sodium cacodylate buffer, *I*=0.05 m) at different ratios.^[b]^

		Δ*T*_m_ [°C]		
	*r* ^[b]^	ctDNA	poly(rA)–poly(rU)	poly(dAdT)_2_
**3**	0.1	4	7	3
**4**	0.1	2	9	2
**5**	0.1	10	8	6
**6**	0.1	1	0	n.d.

[a] Error in Δ*T*_m_=±0.5 °C. [b] *r*=*r*_[compound]/[polynucleotide]_.

### Fluorimetric titrations with DNA and RNA

As all compounds are highly emissive (Table 1), fluorimetric titration experiments were performed for a variety of dsDNA/RNA, as well as ssDNA/RNA. Due to the aggregation properties of **3** and **5**, the titration experiments proved to be nontrivial and had to be performed at as low a concentration of the compound as possible. To obtain comparable data, compounds **4** and **6** were also studied at the lowest possible concentrations, although these compounds did not show aggregation‐related effects. The calculation of binding constants was possible for most titrations by nonlinear fitting of the data by means of the Scatchard equation[^130^, ^131^] (Table 3).

**Table 3:** Binding constants (log *K*_S_) of **3**–**6** with polynucleotides calculated by analyses of fluorimetric titrations;^[a]^ at pH 7.0 in sodium cacodylate buffer, *I*=0.05 M.

		**3**	**4**	**5**	**6**
ds	ctDNA	8.8	8.6	8.5	7.5
	poly(dAdT)_2_	5.1^[c]^	7.8	7.7	7.4
	poly(dGdC)_2_	6.6^[c]^	8.4	7.9	7.8
	poly(rA)–poly(rU)	6.0^[c]^	8.6	>9	7.9
ss	poly(rA)	>9^[b]^	8.4	7.8	7.1
	poly(dA)	7.6	>9^[b]^	>9^[b]^	n.d.
	poly(rU)	7.0	7.4	8.8	7.1
	poly(dT)	7.6	8.5	7.4	n.d.
	poly(rG)	7.1	8.7	8.7	7.3
	poly(rC)	7.4	7.1	>9^[b]^	7.2

[a] Analyses of titration data by means of the Scatchard equation[^130^, ^131^] gave values of the ratio *n* [bound compd.]/[polynucleotide]=0.2–0.5; for easier comparison, all log *K*_S_ values were re‐calculated for fixed *n=*0.25 (ds‐polynucleotides) and *n=*0.5 (ssRNA/RNA). Correlation coefficients were >0.99 for all calculated *K*_S_ values. [b] The first addition of DNA/RNA even at the lowest *c*(dye) yielded strong and maximum emission change, not allowing accurate calculation of the binding constant. [c] Due to competition between single‐molecule binding and aggregation, apparent binding constants are lower.

Detailed analysis of the binding constants (Table 3) revealed that all of our compounds bind to DNA/RNA with comparatively high affinities^[130]^ for small molecules (log *K*_S_>7). In many cases, the affinity is even in the nanomolar range, which is considered exceptionally strong for small molecule/polynucleotide interactions. Intriguingly, **3**–**6** show similar affinities for dsDNA/RNA and ssDNA/RNA, which is uncommon, as single‐stranded polynucleotides usually bind small molecules at least 2–3 orders of magnitude weaker than double‐stranded polynucleotides.^[22]^

The somewhat weaker binding of **6** compared to **3**, **4**, **5** to dsDNA/RNA agrees nicely with the thermal denaturation results (Table 2), again indicating that the short and very rigid linker of **6** interferes to some extent with binding to the double‐stranded polynucleotides. Below, selected examples are discussed in greater detail, starting with the results for the nonaggregating compounds **4** and **6**.

**Figure 7:**
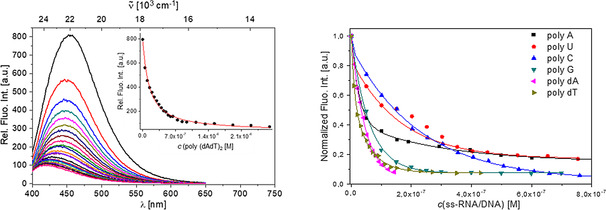
Left: Fluorimetric titration of **4** (*c=*5×10^−9^ m ^;^ *λ*_ex_=372 nm) with poly (dAdT)_2_ as representative of all dsDNA/RNA titrations (Figures S38–S41); inset: dependence of relative fluorescence intensity at 448 nm on *c*(poly (dAdT)_2_. Right: Dependence of normalized fluorescence at *λ*_max_=448 nm on *c*(ssDNA/RNA), fitting to the Scatchard equation[^130^, ^131^] yielded the parameters shown in Table 3. All measurements were made at pH 7 in sodium cacodylate buffer, *I*=0.05 M.

**Figure 8:**
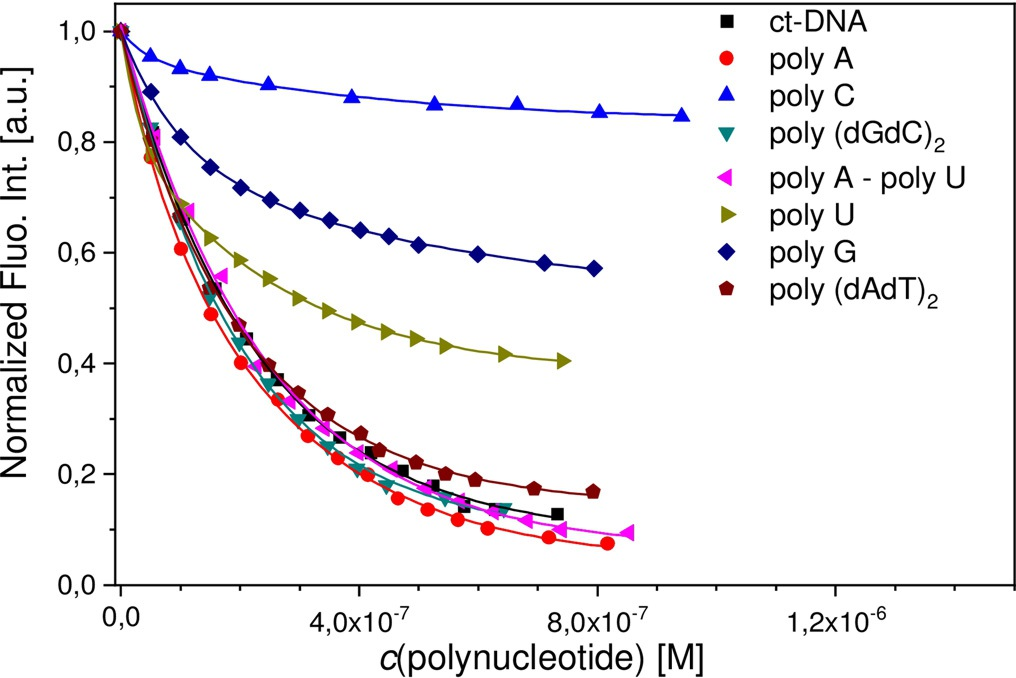
Fluorimetric titrations of compound **6** (*c=*5×10^−8^ m; *λ*_ex_=476 nm) with various dsDNA/RNA and ssRNA; dependence of fluorescence at *λ*_max_=527 nm on *c*(polynucleotide), fitting to the Scatchard equation[^130^, ^131^] yielded the parameters shown in Table 3. All measurements were made at pH 7 in sodium cacodylate buffer, *I*=0.05 M.

Addition of any dsDNA/RNA or ssDNA/RNA resulted in very similar, strong quenching (ca. 90 %) of the emission of compound **4**, accompanied by a hypsochromic shift of the emission maximum of around 30 nm (2000 cm^−1^; Figure 7). The short anthracene compound **6** showed similar, nonselective quenching for all dsDNA/RNA. Very intriguingly, the fluorescence response of compound **6** was highly sensitive to the base composition of ssRNA (Figure 8): poly(rC) (10 % quenching); poly(rG) (40 %); poly(rU) (60 %) and poly(rA) (>95 %). This selectivity concerning emission quenching was not observed for the binding affinity, as all ssRNA show very similar binding constants (Table 3). In addition, it does not correlate with the redox potentials of the nucleobases and their impact on quenching efficiency.^[132]^ Therefore, such a selective fluorimetric response could be correlated with the positioning of the very rigid fluorophore **6** within a particular ssRNA polynucleotide, whereby a combination of the nucleobase size, electronic properties and flexibility control interactions of the fluorophore with the target and the consequent emission of the complex formed.

However, aggregation‐inclined analogues **3** and **5** showed more complex behaviour in the fluorimetric titrations with DNA/RNA.

**Figure 9:**
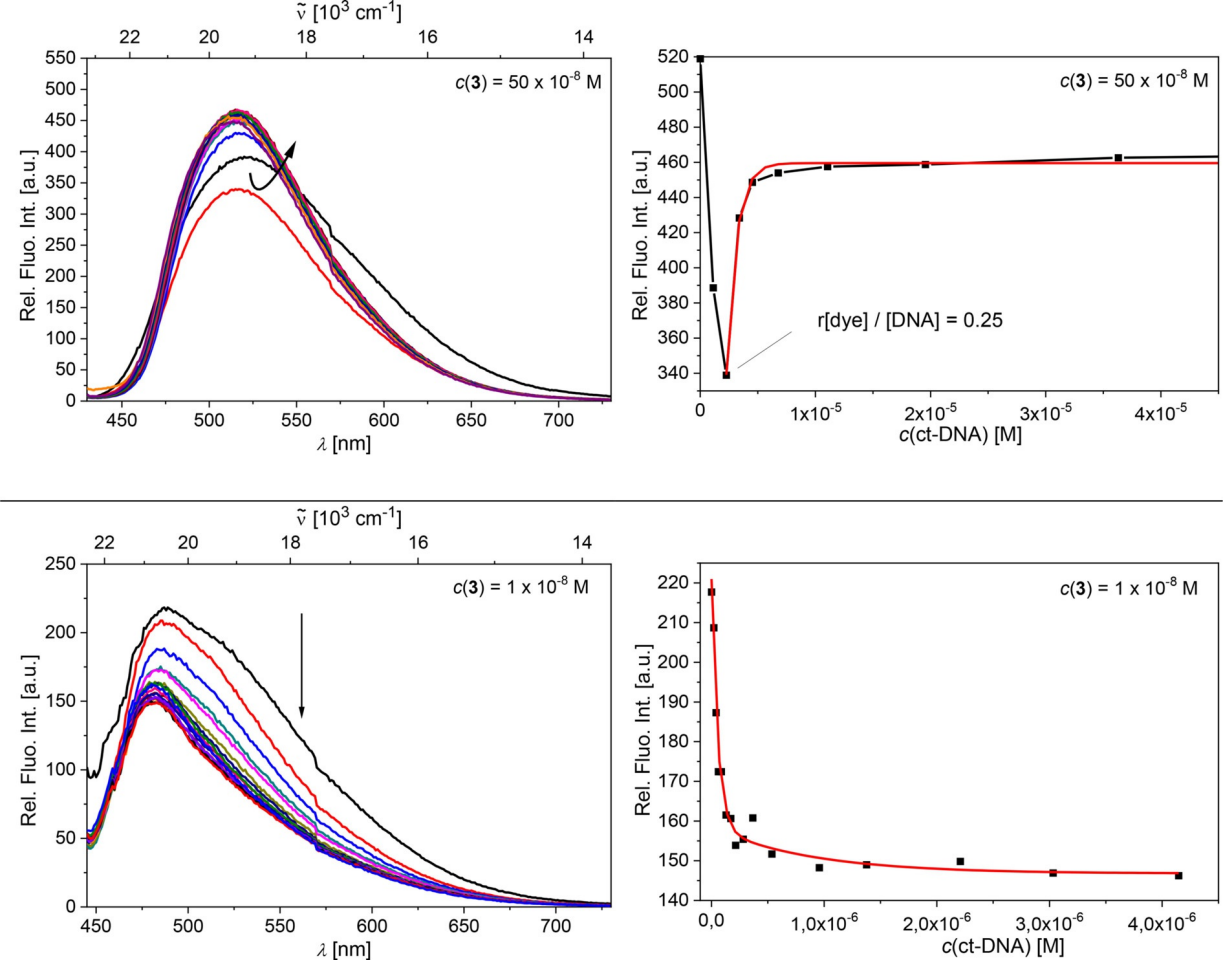
Top: Fluorimetric titrations of **3** at *c=*50×10^−8^ m and dependence of fluorescence at *λ*_max_=519 nm on *c*(ctDNA). Bottom: Fluorimetric titrations of **3** at *c=*1×10^−8^ m with ctDNA and dependence of fluorescence at *λ*_max_=519 nm on *c*(ctDNA), fitting to the Scatchard equation[^130^, ^131^] yielded the parameters shown in Table 3. All measurements were made at pH 7 in sodium cacodylate buffer, *I*=0.05 m, *λ*_ex_=412 nm.

For example, titration with ctDNA at two different concentrations of **3** (1 and 50 ×10^−8^ m), revealed significantly different profiles (Figure 9). Thus, at higher concentration (Figure 9 top) and at an excess of **3** with respect to DNA (ratio *r*_[**3**]/[DNA]_>0.25) the dye aggregated along the DNA helix and emission of **3** was quenched. At an excess of DNA (*r*_[**3**]/[DNA]_≪0.2) the dye molecules were redistributed along the DNA helix, each to a separate binding site, and emission of **3** was partially restored; however, it did not reach the starting intensity of free **3**. At 50 times lower concentration (Figure 9 bottom), DNA‐induced aggregation of **3** was not observed, and the dominant process was quenching of emission, attributed to a single type of binding process.

**Figure 10:**
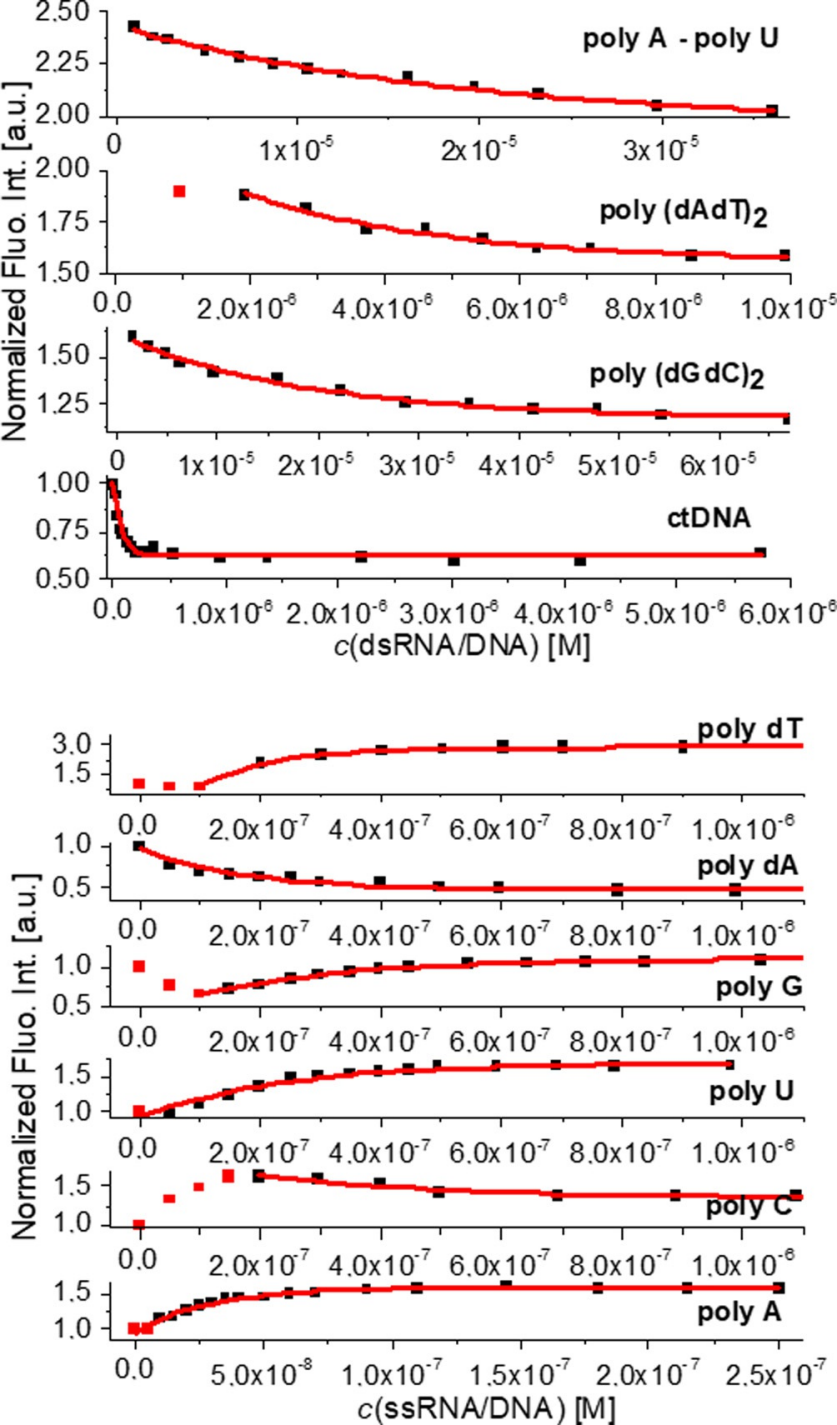
Fluorimetric titration of **3** (*c=*5×10^−8^ m; *λ*_ex_=412 nm) with DNA/RNA; dependence of normalized fluorescence at *λ*_max_=500 nm on *c*(DNA/RNA), fitting to the Scatchard equation[^130^, ^131^] yielded the parameters shown in Table 3. All measurements were made at pH 7 in sodium cacodylate buffer, *I*=0.05 M.

For the other ds‐ or ss‐polynucleotides, all titrations were performed at the lowest possible concentration of **3**. The titration profiles are summarized in Figure 10. However, as the secondary structure and consequently the availability of binding sites of the various DNA/RNA differ strongly from each other (Table S4), in some cases (poly(rG), poly(rC), all dsDNA/RNA except ctDNA) the aggregation of **3** along the polynucleotide at ratios *r*_[compound]/[polynucleotide]_>0.25 could not be avoided. Nevertheless, considering changes at large excesses of DNA/RNA with respect to dye (*r*_[compound]/[polynucleotide]_≪0.2) as representative for single molecule binding, analyses of this part of the titration data via the Scatchard equation[^130^, ^131^] can give a good estimate of the binding constants (Table 3).

**Figure 11:**
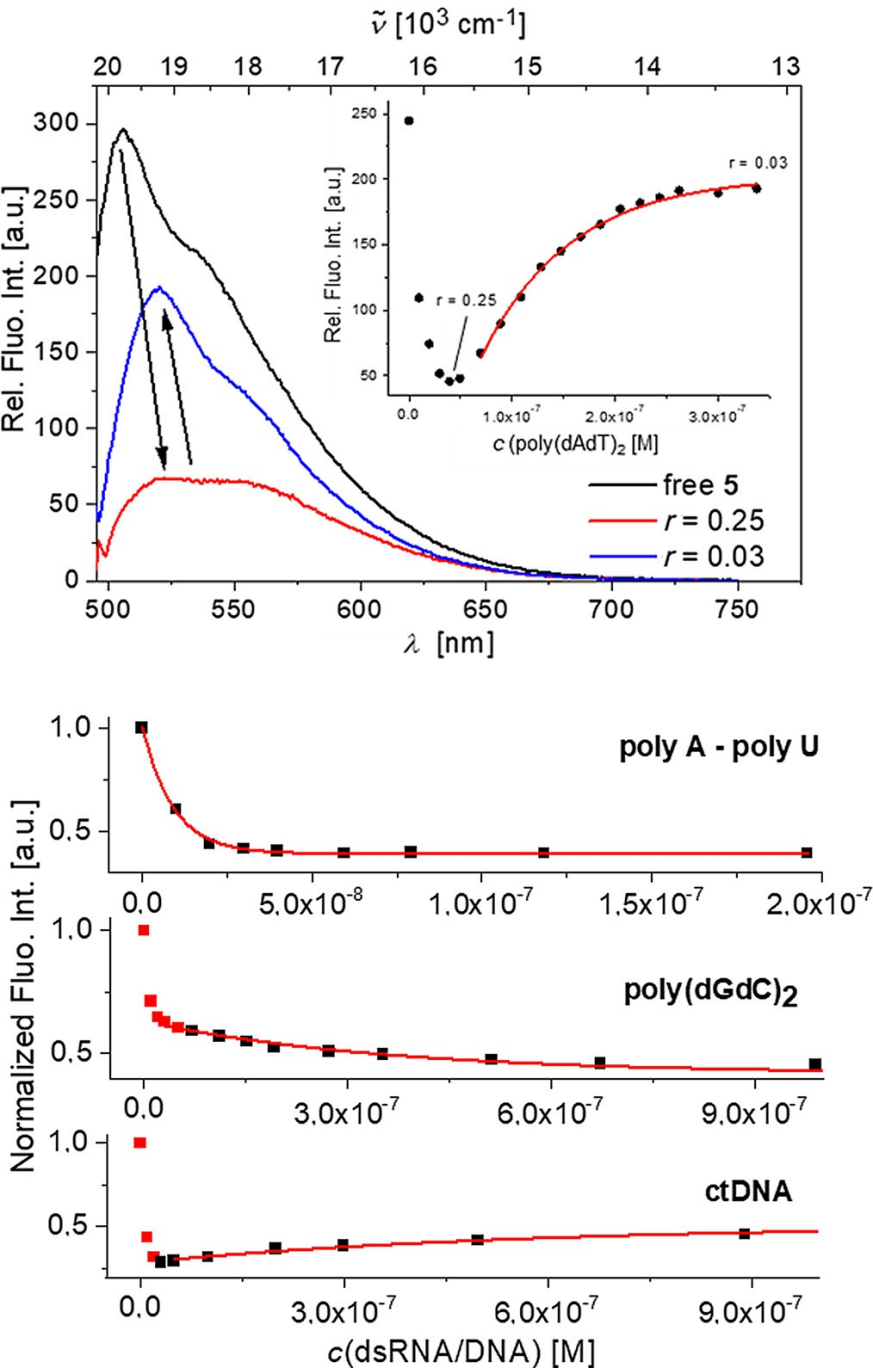
Top: Changes in the emission spectra of **5** upon titration with poly(dAdT)_2_, inset: dependence of relative fluorescence intensity at 520 nm on *c*(poly(dAdT)_2_). Bottom: Fluorimetric titrations of **5** (*c=*1×10^−8^ m; *λ*_ex_=470 nm) with dsDNA/RNA; dependence of normalized fluorescence at *λ*_max_=550 nm on *c*(DNA/RNA), fitting to the Scatchard equation[^130^, ^131^] yielded the parameters shown in Table 3. All measurements were made at pH 7 in sodium cacodylate buffer, *I*=0.05 M.

Similarly, DNA/RNA‐induced aggregation in the presence of an excess of dye was observed for anthracene analogue **5** (Figure 11). The titration of compound **5** with poly (dAdT)_2_ (Figure 11 top) showed a very well resolved aggregation–deaggregation process, as indicated by a gradual red shift of the emission maximum toward 550 nm for ratios *r* ${}_{\left[5\right]/[\mathrm{poly}\left(\mathrm{dAdT}\right){}_{2}]}$ >0.25, followed by a gradual blue shift of the emission maximum back to 520 nm for ratios *r* ${}_{\left[5\right]/[\mathrm{poly}\left(\mathrm{dAdT}\right){}_{2}]}$ <0.25. This again suggests aggregation along the polynucleotide helix for comparatively high dye concentrations and redistribution of the dye molecules into separate binding sites for an excess of polynucleotide. For the titration with poly(rA)–poly(rU), such an aggregation‐deaggregation process was not observed. Only one binding process was observed, as indicated by a systematic shift of the emission maximum toward 550 nm, even at a large excess of RNA (*r*_[**5**]/[poly(A)–poly(U)]_<0.05; Figure S65). Thus, it is suggested that the much deeper major groove of RNA (Table S4) can accommodate dimeric aggregates of **5** much more efficiently than the smaller minor groove of poly (dAdT)_2_.

### CD experiments

Having studied the changes of the spectroscopic properties of our compounds upon interaction with polynucleotides, we chose circular dichroism (CD) spectroscopy as a highly sensitive method to gain insight into the conformational changes of the secondary structure of polynucleotides induced by small molecule binding.^[133]^ In addition, achiral compounds **3**–**6** might display induced circular dichroism (ICD) upon interaction with polynucleotides, which would provide information on the type of interaction present.[^134^, ^135^]

The short anthracene compound **6** did not have any measurable influence on the CD spectra of dsDNA/RNA (Figure S67); this suggests that its binding does not disturb the secondary structure of the respective polynucleotide. Also, for compound **6**, no ICD bands >300 nm were observed upon binding to any dsDNA/RNA. Thus, molecules of **6** were either not uniformly oriented with respect to the chiral axis of dsDNA/RNA or the transition vectors of **6** were oriented with respect to the chiral axis of dsDNA/RNA to yield ICD bands of negligible intensity.[^134^, ^135^] Similarly, addition of **3** or **4** caused only minor decreases in intensity in the CD spectra of dsDNA/RNA and no measurable ICD bands for the compounds (Figures S68 and S69), also suggesting only small changes in the secondary structure of the polynucleotides. These CD results along with the strong binding affinities of **3**, **4** and **6** to dsDNA/RNA (Table 3) and the observed thermal stabilization effects (Table 2), support binding within the minor groove of dsDNA and the major groove of dsRNA, respectively. This is in accordance with our earlier studies on this novel class of DNA/RNA sensors.[^88^, ^89^]

**Figure 12:**
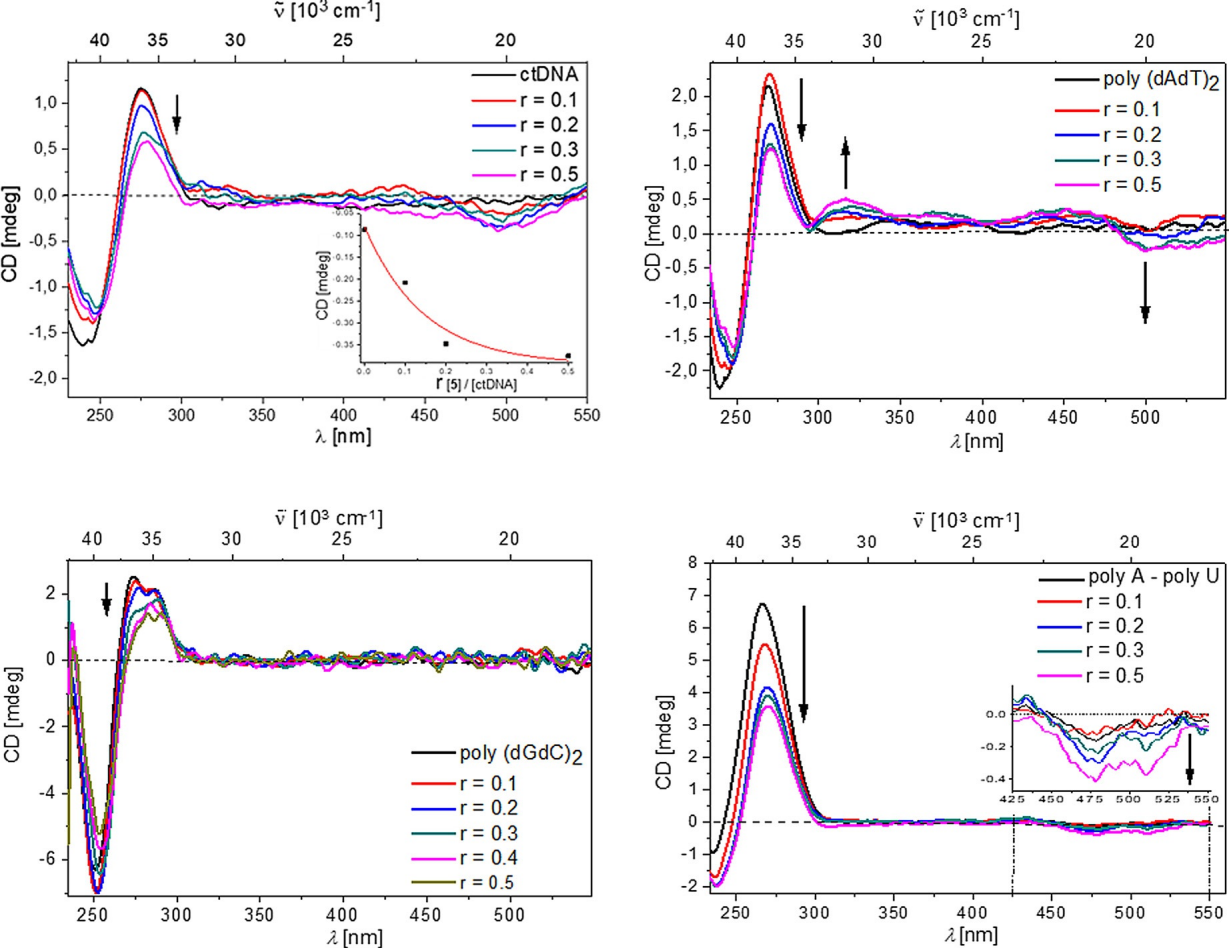
CD titration of ctDNA (inset: dependence of CD at 500 nm on *r*_[**5**]/[ctDNA]_), polyA–polyU, poly(dAdT)_2_ and poly(dGdC)_2_ (all DNA/RNA *c=*2×10^−5^ m) with **5** at molar ratios *r*_[**5**]/[polynucleotide]_=0.1–0.5. All measurements were made at pH 7.0 in sodium cacodylate buffer, *I*=0.05 M.

In contrast, addition of the longer anthracene derivative **5** significantly decreased the intensity of the CD spectra of dsDNA/RNA (Figure 12, *λ*=270–290 nm), suggesting unwinding of helical structures, which causes a partial loss of chirality. Complexes of compound **5** with polynucleotides containing A, T and U base pairs displayed ICD bands in the absorption range of the compound (Figure 12, *λ*=450–550 nm), which suggests a very uniform orientation of the transition vectors of the molecules (*λ*=450–550 nm) with respect to the chiral axis of DNA.[^130^, ^131^] Intriguingly, the complex of **5** with GC‐DNA (poly (dGdC)_2_) showed no measurable ICD bands and the CD spectrum of GC‐DNA (*λ*<300 nm) changed only marginally upon addition of **5**. The main structural difference between GC‐DNA and AT‐DNA (poly(dAdT)_2_) is a better availability of the minor groove of the latter for binding of small molecules (Table S4). Thus, our CD results suggest that only in the case of AT‐DNA does compound **5** insert deeply enough into the minor groove to yield a uniform orientation of the molecules with respect to the chiral axis of DNA, while simultaneously disturbing the DNA helicity by this insertion process. The same is suggested, analogously, for the major groove of AU‐RNA (poly(A)–poly(U)), which is the common binding site for small molecules with RNA.^[128]^ In GC‐DNA, amino groups of guanine in the minor groove sterically hinder small molecule insertion and, thus, molecules of **5** occupy a more heterogeneous orientation along the DNA helix, consequently displaying negligible ICD bands.

### Molecular modelling

To corroborate and explain further our experimental findings, especially concerning compound **5**, a better structural understanding of the observed intramolecular interactions was required.

The observed aggregation of the compounds, particularly that of anthracene derivative **5**, is not trivial to explain due to the sterically demanding triarylboron dications attached to both sides of the linker. Those dications would additionally impose charge repulsion when two anthracene moieties are stacked in a dimer.

**Figure 13:**
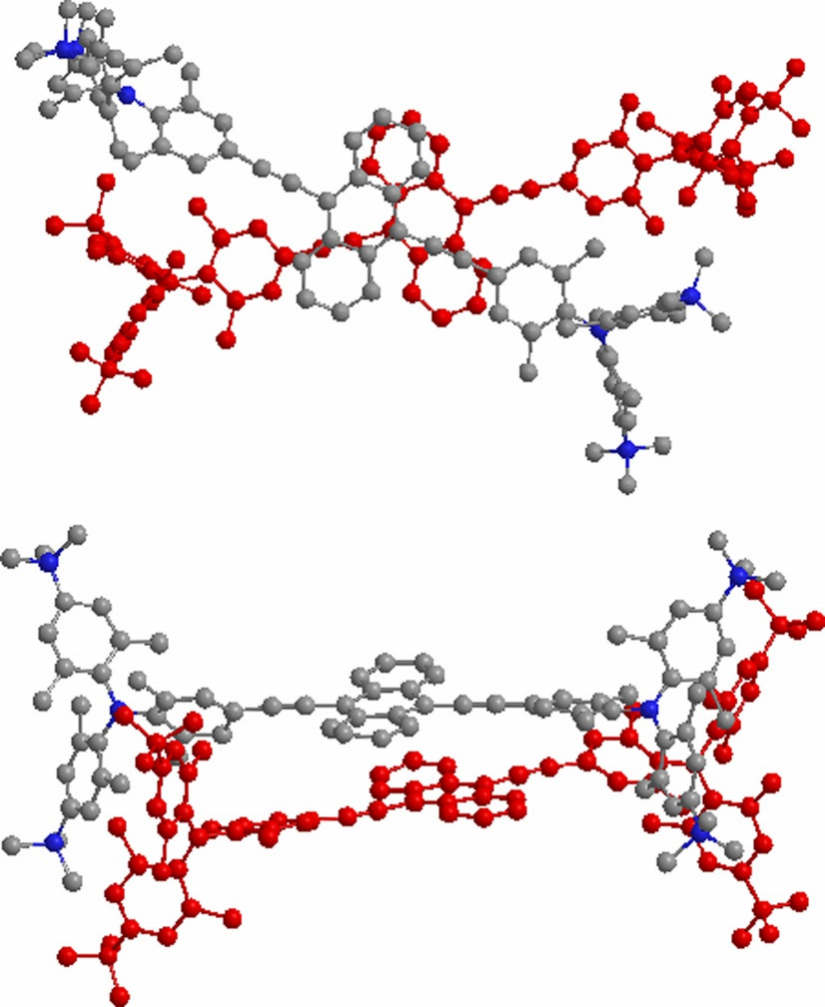
Structure of the dimer aggregate of **5**, displayed from two different views, obtained after 200 ns of MD simulation in water. Hydrogen atoms are omitted for clarity.

Thus, we first performed molecular modelling of a dimer of compound **5** in water (for details, see the General Information in the Supporting Information). The minimized structure obtained after 200 ns of molecular dynamics (MD) simulation in water is shown in Figure 13. It is characterized by the asymmetric off‐set of two anthracenes, which are partly overlapping.

**Figure 14:**
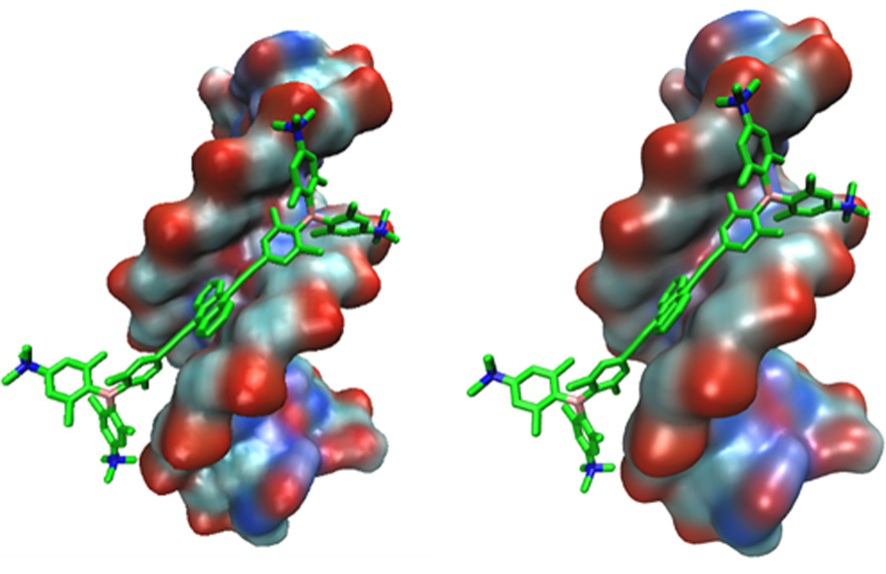
The result of 100 (left) and 300 ns (right) of molecular dynamics simulation of **5**/DNA complexes. Hydrogen atoms are omitted for clarity.

The fluorimetric results suggest two binding modes for complexes of **5** with AT‐containing DNAs (poly(dAdT)_2_). One (at ratios *r*_[**5**]/[DNA]_>0.25) is characterized by emission of aggregated **5**, and the other (at ratios *r*_[**5**]/[DNA]_≪0.25) by monomer emission (cf. Figure 11). The binding mode of the monomer is supported by induced CD bands and suggests AT‐DNA minor groove binding of **5**. To determine the structural arrangement of **5** within the minor groove of AT‐DNA, docking was performed using PyMOL software and compound **5** was docked into the minor groove of AT‐DNA using the optimized position previously determined for analogue **1** ^[88]^ as a template. Compound **5** slightly reoriented within the groove and remained inside the groove during the entire 300 ns of MD simulation (Figure 14). Multipoint measurements of the distances between opposite strand backbones (P atoms of paired nucleobases) revealed only a slight broadening (<10 %) of the minor groove upon binding of **5**.

**Figure 15:**
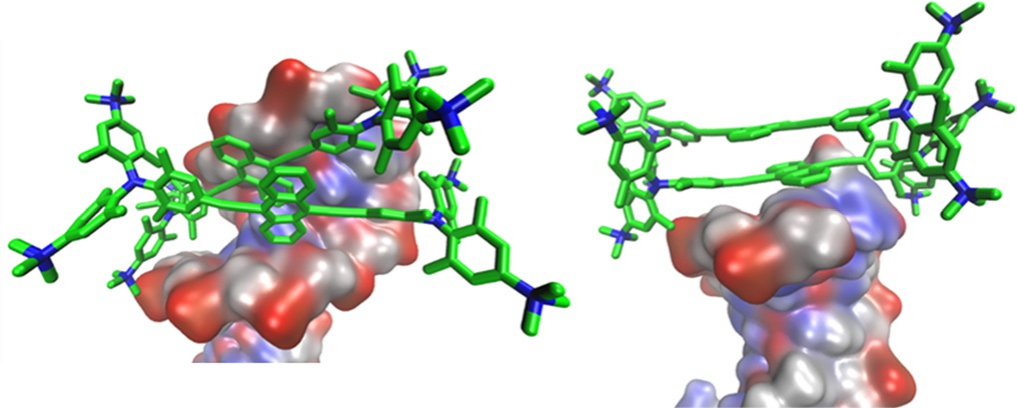
The MM results after 150 (left) and 300 ns (right) of molecular dynamics simulation of the **5**/DNA complex with **5** bound initially in the minor groove. Hydrogen atoms are omitted for clarity.

The aggregation process observed at ratios *r*_[**5**]/[DNA]_>0.25, can either be explained by insertion of dimers of **5** into the minor or major groove of DNA or by the stacking of molecules of **5** in long stacks parallel to the DNA helix (similar to a process observed for porphyrins^[136]^). To investigate this aggregation process, a dimer of **5** was first inserted into the minor groove of AT‐DNA using the obtained monomer complex (Figure 14) as a template. During the first 150 ns of MD, the dimer remained bound into the minor groove (Figure 15), revealing a further slight broadening (<5 %) of the minor groove with respect to the monomer complex. However, at about 200 ns the dimer started to migrate through the minor groove towards the polynucleotide termini and, after 300 ns of MD simulation, it nested there, stabilized by stacking interactions with the terminal base pair (Figure 15). Such a “capping” effect is frequently observed when studying the binding of large aromatic moieties to short oligonucleotides due to the very stable stacking interactions with base pairs at the termini.^[137]^ Thus, our results suggest that a dimer of **5** can efficiently bind into the minor groove of a long AT‐polynucleotide.

**Figure 16:**
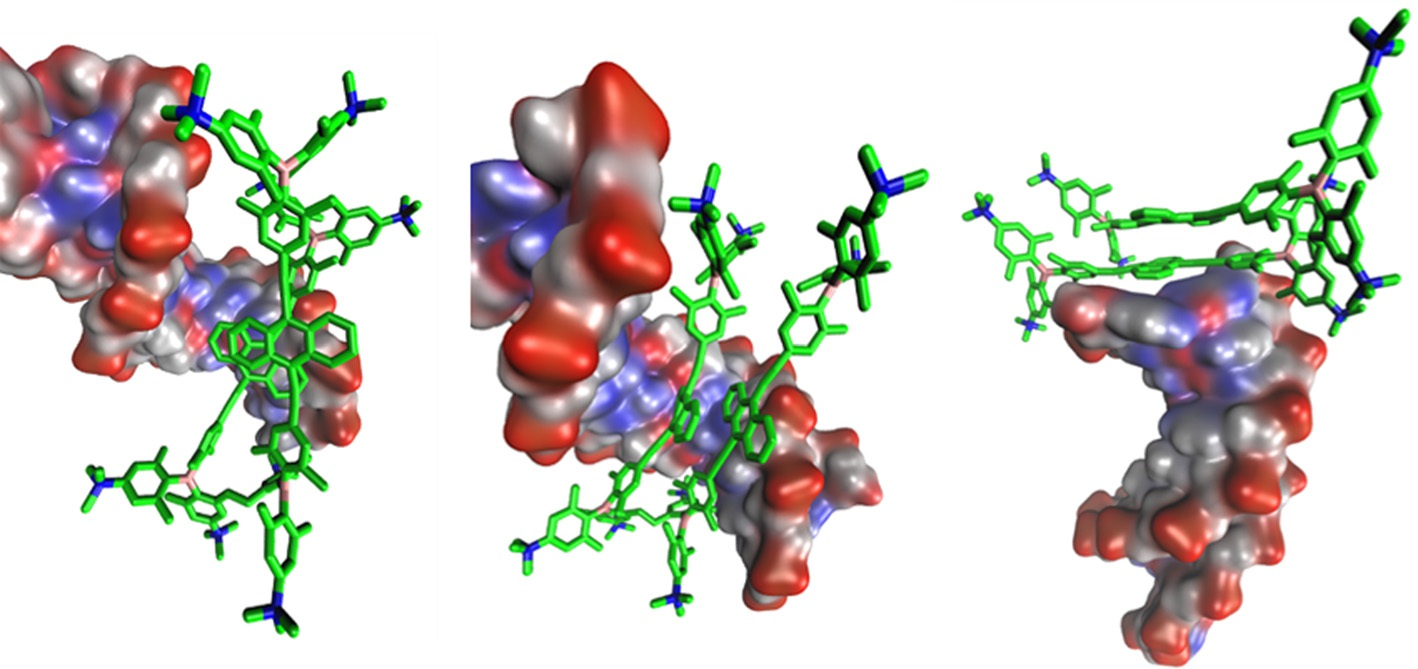
The results of 90 (left), 95 (middle) and 300 ns (right) of molecular dynamics simulation of the **5**/DNA complex with the dimer of **5** bound initially into the major groove. Hydrogen atoms are omitted for clarity.

Secondly, we inserted a dimer of **5** into the major groove of AT‐DNA (Figure 16), to see if a stable complex within the major groove of AT‐DNA can be formed, as determined for other bulky molecules.^[138]^ This time, the dimer started to migrate out of the groove after 90 ns. For the following 150 ns, it oscillated around the groove entrance, never staying deeply inserted within the groove. At the end of 300 ns of MD simulation, it adopted an almost identical position at the polynucleotide end to that observed for the dimer bound into the minor groove. The major groove of AT‐DNA is, thus, considered a much less adequate binding site for dimers of **5**.

Thus, our molecular modelling results strongly support binding of **5** into the minor groove of DNA as a dominant binding mode. However, at “crowding conditions”, that is, an excess of **5** over DNA binding sites, dimers of **5** can also form within the minor groove, without significantly disturbing the DNA helix. Binding of dimers of **5** within the major groove of DNA is less probable due to the much larger size of this binding site, not allowing all positive charges of the dimer of **5** to reach the negatively charged backbones efficiently at the same time. In addition, insertion of a dimer of **5** would not displace all water molecules inside the major groove and, thus, the hydrophobic driving force is diminished.

Due to the structural similarity and the fact that, for compounds **1** and **5**, the minor groove was determined to be the most likely binding site via molecular modelling, it is suggested that compounds **3**, **4** and **6** form similar complexes with DNA.

### Raman and surface‐enhanced Raman scattering (SERS) spectroscopy

As we observed strong Raman responses for previously reported compound **2**,^[89]^ we studied analogues **3**–**5**, as well as the short anthracene derivative **6** under the same conditions.

The Raman spectra of **3**–**5** in aqueous solutions (*c=*1×10^−4^ m) were of low intensity, dominated by the broad water stretching and bending bands around 3220 and 1640 cm^−1^, respectively (Figure S71). Nevertheless, some bands originating from our compounds were observed and could be assigned (Table S5). Calculated Raman spectra for compounds **3**–**5** are in good agreement with the experimental data (Table S10). The characteristic band in the C≡C stretching region (ca. 2200 cm^−1^) was observed in the spectra of **4** and **5** and the exact band position was affected by the aromatic substituent between the triple bonds. The C≡C stretching bands occured at 2209 and 2182 cm^−1^ for the compounds containing benzene (**4**) and anthracene (**5**) cores, respectively, in agreement with the fact that lower C≡C stretching frequencies characterize larger π‐conjugated systems.^[139]^ A characteristic band at around 1590 cm^−1^ was observed in the spectra of all three compounds, which can be attributed to aromatic stretching, distributed over the respective aromatic linker between the two boron atoms. In the Raman spectrum of **3**, possessing a bithiophene moiety between the triple bonds, the C≡C band was hardly observable, while bands attributed to thiophene ring stretching modes (1472 and 1453 cm^−1^) were observed. According to our calculations, the energy of the C≡C stretching mode of **3** should be between the values obtained for **4** and **5**. This is consistent with the data obtained by SERS spectroscopy (vide infra). In contrast to compounds **3**–**5**, the Raman scattering of anthracene derivative **6**, not containing triple bonds, was overlapped by fluorescence and thus not observed at all, even at higher concentration (*c=*2×10^−3^ m). This clearly pointed out the significance of the triple bonds in the structure, which provide strong Raman bands, allowing Raman detection of the molecule in the sub‐millimolar concentration range.

**Figure 17:**
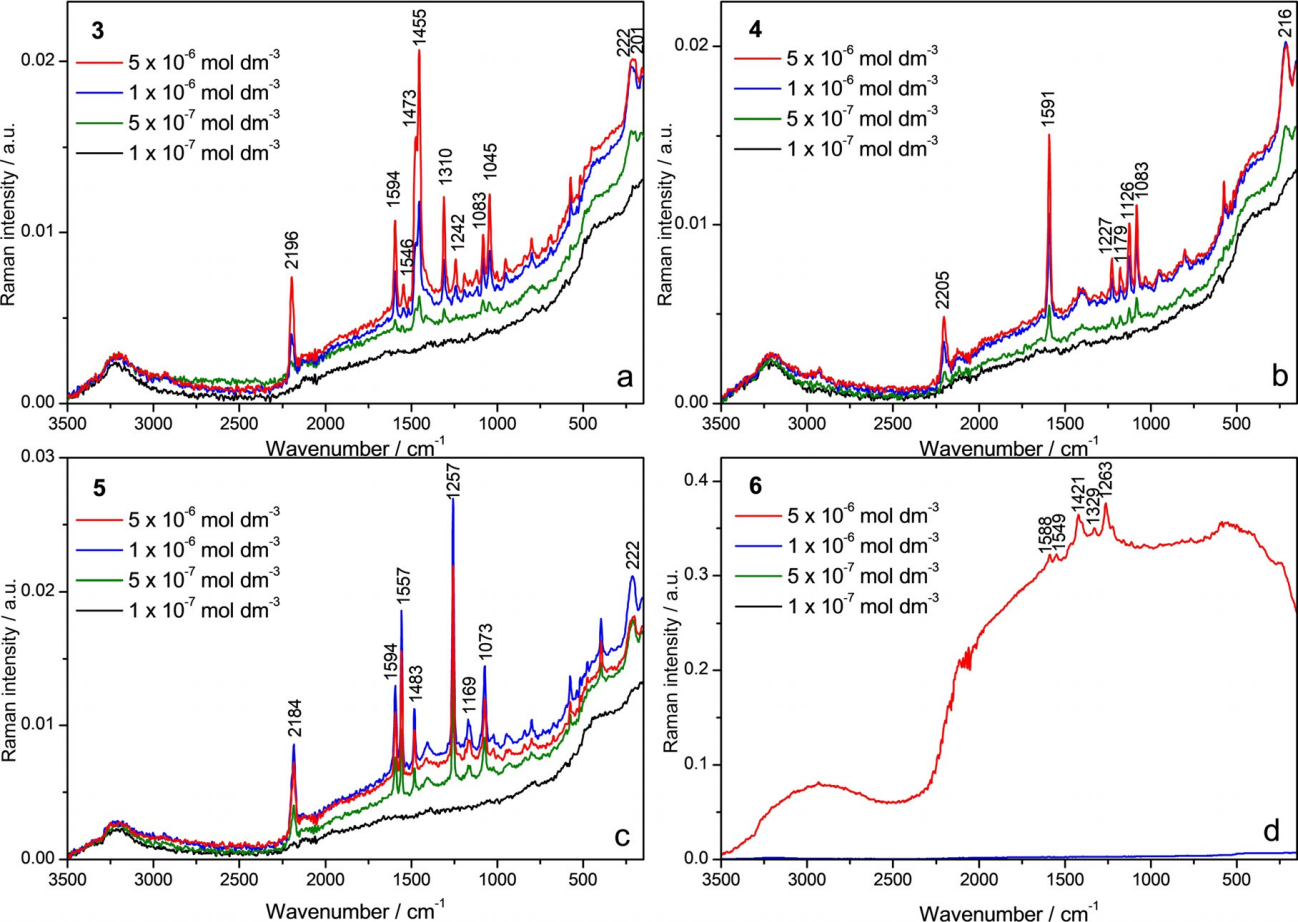
Concentration‐dependent SERS spectra of **3**–**6** in the silver colloid, *c=*1×10^−7^, 5×10^−7^, 1×10^−6^, 5×10^−6^ m; *λ*_ex_=1064 nm.

Unlike the Raman spectra, the SERS spectra of all compounds, **3**–**6**, were obtained and preliminarily assigned (Figure 17, Table S6). The observed surface‐enhanced Raman scattering pointed to adsorption of the molecules onto the enhancing silver nanoparticles, mostly driven by attractive electrostatic interactions between the negatively charged citrate ions on the silver surface and positively charged trimethylamino groups of the compounds. For compounds **3**–**5**, the characteristic SERS bands were observable at a concentration as low as 5×10^−7^ m, which is an order of magnitude higher than the lowest detectable concentration for **2**.^[89]^ In contrast, the SERS response of the short anthracene analogue **6** was very weak, with weak bands characteristic of anthracene (1549, 1329 and 1263 cm^−1^) and phenyl (1588 and 1421 cm^−1^) moieties, and observed only at the highest measured concentration of 5×10^−6^ m (Figure 17 d). This confirms the essential role of the triple bonds in the molecular structure for the Raman scattering ability of our molecules. It also demonstrates that, even though directly connected triple bonds increase the Raman intensity, the insertion of different aromatic moieties between them still gives satisfying Raman responses and is thus an alternative approach for the design of dual Raman and fluorescent chromophores.

In the SERS spectra of **3**, **4** and **5**, the following common bands were observed, respectively: a band at about 2200 cm^−1^, assigned to the stretching of the C≡C bonds, a band at about 1595 cm^−1^, attributed to the aryl stretching distributed over the respective aromatic linker between the two boron atoms, and a band at about 1080 cm^−1^, associated with the stretching of the bonds between the boron atom and the aryl rings. The position of the C≡C bond stretching bands at 2184 (**5**), 2196 (**3**) and 2205 cm^−1^ (**4**) was significantly dependent on the respective aromatic moiety, namely, anthracene, thiophene and benzene. In addition, moderate to strong bands associated with thiophene were observed at 1473, 1455, 1310, 1242 and 1045 cm^−1^ in the spectrum of **3**, while medium to intense bands distinctive of anthracene were observed at 1557, 1483, 1257 and 1169 cm^−1^ in the spectrum of **5**.

By decreasing concentration from 5×10^−6^ to 1×10^−7^ m, the SERS intensity diminished for all compounds except for **5**, for which the most intense SERS spectrum was observed at a concentration of 1×10^−6^ m (Figure 17 c). In accordance with UV/Vis absorbance and fluorescence measurements which indicated stacking of **5** in aqueous solution, by lowering the concentration the equilibrium was shifted to monomeric molecules, which upon adsorption onto the enhancing metal surface adopted a position different from that of the aggregated molecules. Thus, it was very likely that at 1×10^−6^ m the bis‐triarylborane longitudinal axes of the monomeric molecules were oriented perpendicular to the silver surface, giving rise to the most enhanced scattering.

**Figure 18:**
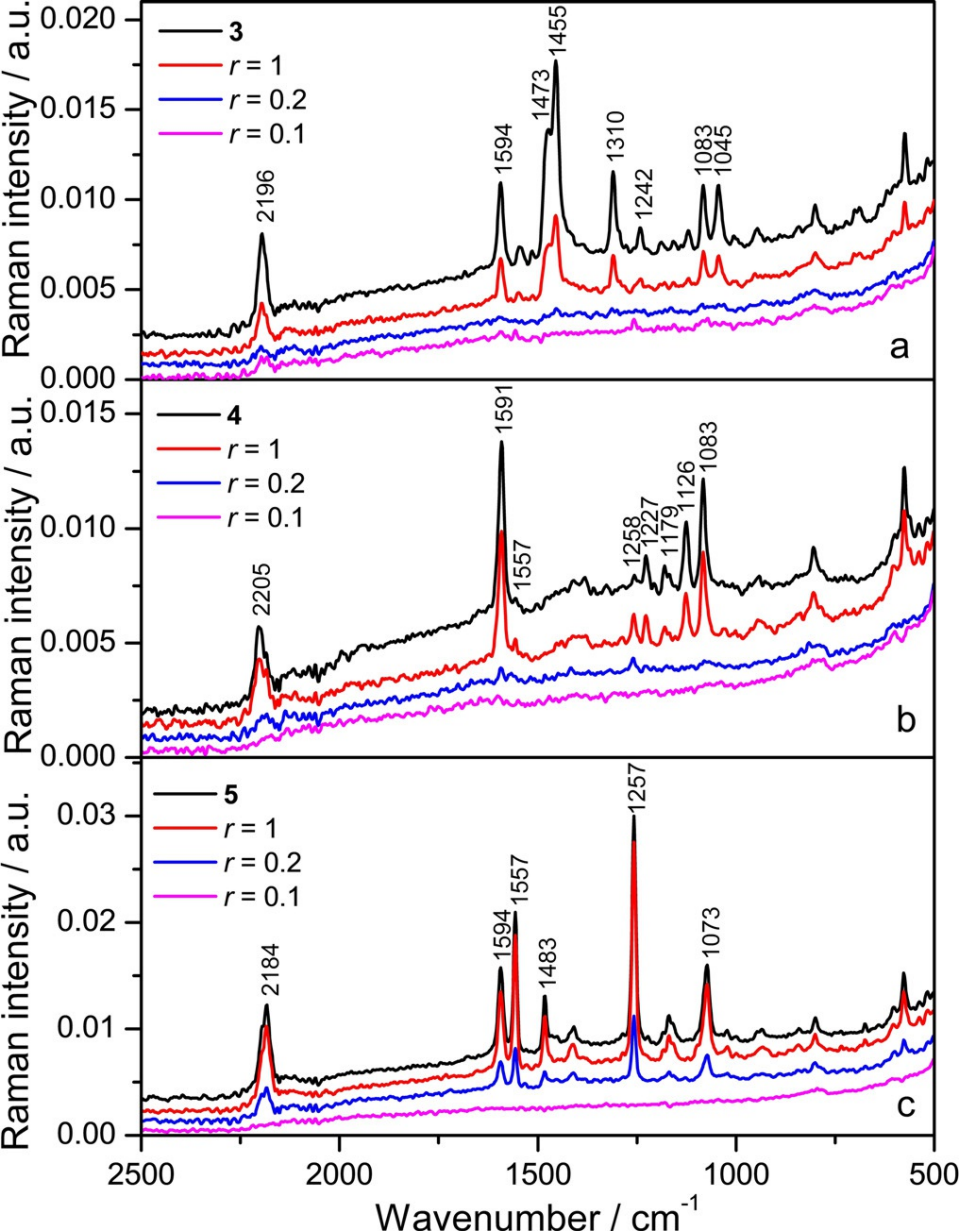
SERS spectra of **3**–**5** and their complexes with ctDNA in molar ratios *r*_[compound]/[ctDNA]_=1, 0.2 and 0.1; *c*(**3**–**5**)=1×10^−6^ m; *λ*_ex_=1064 nm. The spectra are displaced for visual clarity.

Furthermore, the SERS spectra of **3**, **4** and **5** were studied upon addition of ctDNA. When compared to the spectrum of the neat compound (*c=*1×10^−6^ m) measured in the buffered silver colloid, the SERS spectra of the complexes of **3**–**5** with ctDNA were very weak at the molar ratio *r*_[compound]/[ctDNA]_=0.2, and not observed at all at the molar ratio *r*_[compound]/[ctDNA]_=0.1 (Figure 18). Presumably, bis‐triarylborane molecules are efficiently bound to the nucleic acid when there is an excess of ctDNA, resulting in a loss of the SERS intensity as the highly negatively charged phosphate backbone of ctDNA prevented efficient adsorption of the complexes onto the silver nanoparticles. Nevertheless, for the complexes of **4** and **5** in equimolar ratio with ctDNA, *r*_[compound]/[ctDNA]_=1, the bands at 1557 and 1258 cm^−1^ (**4**/ctDNA) and at 1557 and 1257 cm^−1^ (**5**/ctDNA) were selectively enhanced (Figures 18 b and c). The former band (1557 cm^−1^) was attributed to stretching of the aromatic moieties, and the latter (1258/1257 cm^−1^) to in‐plane deformation of the aromatic CH groups. Both bands, in essence, originate from arene ring vibrations and can be easily associated with three phenyl substituents as well. Thus, it can be assumed that, upon binding with the nucleic acid, either the central part of the molecule (benzene, anthracene) or the phenyl moieties linked to boron were placed closer and/or more perpendicular towards the enhancing silver surface. New bands originating from the nucleic acid were not observed in the SERS spectra of the complexes.

## Conclusions

We have successfully extended our novel class of tetracationic bis‐triarylborane DNA and RNA sensors by four molecules **3**–**6**. Three of them contain long bis(phenylethynyl)aryl (**3**: aryl=5,5′‐2,2′‐bithiophene; **4**: aryl=1,4‐benzene; **5**: 9,10‐anthracene) linkers between the two boryl moieties and can be considered as dual Raman and fluorescence chromophores, while the short analogue **6** possesses only an anthracene moiety as the linker and can be considered a fluorophore probe. Analysis of the solid‐state structures of the neutral precursors to **3** and **4** reveal a high level of flexibility for ethynyl‐containing aryl linkers. Concentration‐ and temperature‐dependent UV/Vis and fluorescence experiments suggest a tendency of compounds **3** and **5** to aggregate, increasing with the ionic strength of the solution. Thermal denaturation experiments revealed strong stabilization of dsDNA/RNA for complexes formed with compound **3**–**5**, similar to that observed for our previously studied compounds **1** and **2**. The very weak stabilization observed for compound **6** demonstrates that a certain length and flexibility of the aromatic linker need to be exceeded for efficient thermal stabilization to occur. All four compounds bind to dsDNA/RNA and ssDNA/RNA with similar affinities; this contrasts with the fact that binding affinities for single‐stranded polynucleotides are typically 2–3 orders of magnitude lower than for double‐stranded polynucleotides. This is consistent with our previous studies[^88^, ^89^] and it is suggested that ssRNA is chain wrapping around the tetracationic bis‐triarylborane motif like a thread around a spindle.

In general, binding of compound **6** to the polynucleotides tested is somewhat weaker than that of the other compounds, which agrees nicely with the thermal denaturation experiments and, again, demonstrates the importance of the linker length and flexibility. For compounds **3** and **5**, aggregation–deaggregation processes were observed in fluorimetric titration experiments with DNA and RNA. Thus, at an excess of dye, an aggregation of the dye along the helical axis of the polynucleotide or in dimeric form inside a groove is suggested, while at an excess of polynucleotide, the molecules are separated and are each transferred to separate binding sites. Based on all of our experimental data, including CD results, we suggest the minor groove as the dominant binding site for dsDNA and the major groove for dsRNA for compounds **3**, **4** and **6**. This is in accordance with our previous findings for compounds **1** and **2**.[^88^, ^89^] For compound **5**, the CD results suggest an unwinding of the helical structure of the polynucleotide upon binding and very uniform orientation with respect to the helical axis for all ds‐polynucleotides tested containing A, T and U base pairs. A molecular modelling study on complexes of compound **5** with AT‐DNA also suggests the minor groove as the dominant binding site and that even dimers of **5** can be accommodated by the minor groove, whereas their binding into the major groove is much less efficient. Strong SERS responses were obtained for **3**–**5** at low concentrations. For compound **6**, weak SERS signals were observed only at the highest concentration measured. This confirms the important role of the triple bonds for strong Raman scattering and demonstrates that the insertion of aromatic moieties between two triple bonds, by which the absorption and emission properties of a molecule can be conveniently tuned, is a feasible alternative for the design of dual Raman and fluorescence chromophores. In addition, the energy of the stretching vibration of the characteristic C≡C bonds was found to be significantly dependent on the aromatic moiety between the triple bonds. In analogy to a variation of the length of the poly‐yne chain,^[104]^ this might be applied as a useful tool in the design of new chromophores suitable for multiplex Raman imaging purposes.

## Conflict of interest

The authors declare no conflict of interests.

## Supporting information

As a service to our authors and readers, this journal provides supporting information supplied by the authors. Such materials are peer reviewed and may be re‐organized for online delivery, but are not copy‐edited or typeset. Technical support issues arising from supporting information (other than missing files) should be addressed to the authors.

Supplementary
