# Supplementary material for: Bis(phenylethynyl)arene Linkers in Tetracationic Bis‐triarylborane Chromophores Control Fluorimetric and Raman Sensing of Various DNAs and RNAs
Source: Chemistry. 2021 Feb 24;27(16):5142–59. doi: 10.1002/chem.202005141 (PMC8048639; doi:10.1002/chem.202005141)
Supplement: Supplementary file 1 — Supplementary [file CHEM-27-5142-s001.pdf]

Chemistry–A European Journal

Supporting Information

# Bis(phenylethynyl)arene Linkers in Tetracationic Bis-triarylborane Chromophores Control Fluorimetric and Raman Sensing of Various DNAs and RNAs

Matthias Ferger,[a] Željka Ban,[b] Ivona Krošl,[b] Sanja Tomić,*[b] Lena Dietrich,[a] Sabine Lorenzen,[a] Florian Rauch,[a] Daniel Sieh,[a] Alexandra Friedrich,[a] Stefanie Griesbeck,[a] Adriana Kenđel,[c] Snežana Miljanić,[c] Ivo Piantanida,*[b] and Todd B. Marder*[a]

# Contents

General Information .... S3

Synthesis .... S8

NMR Spectra .... S14

Single-Crystal X-Ray Diffraction .... S20

Linear Optical Properties .... S22

Optical Properties in Sodium Cacodylate .... S26

Studies of Interactions with DNA and RNA .... S34

Raman and SERS Measurements .... S53

Theoretical Studies .... S55

References .... S71

## General Information

**Synthesis and Routine Characterization.** Unless otherwise noted, the following conditions apply. Reactions were performed using standard Schlenk or glovebox (Innovative Technology Inc.) techniques under an atmosphere of argon. Only oven-dried glassware was used. Solvents used for reactions were HPLC grade, dried using an Innovative Technology Inc. Solvent Purification System, and further deoxygenated.

Compounds **1**,[1] **2**,[2] **6**,[3] Bis[4-(*N*,*N*-dimethylamino)-2,6-dimethylphenyl]-(4-ethynyl-2,6-dimethylphenyl)borane,[2] 9,10-diiodoanthracene[4] and $Pd(PPh_3)_2Cl_2$[5] were synthesized according to literature procedures. All other starting materials were purchased from commercial sources and were used without further purification.

Reaction progress was monitored using thin layer chromatography (TLC) plates pre-coated with a layer of silica (Polygram® Sil G/UV254) with fluorescent indicator UV254 from Marchery-Nagel. Automated flash column chromatography was performed using a Biotage® Isolera Four system with silica gel (Biotage SNAP cartridge KP-Sil 50g or KP-Sil 100g obtained from Biotage) as the stationary phase and the solvent system indicated. Solvents were generally removed *in vacuo* using a rotary evaporator at a maximum temperature of 50 °C.

$^{1}H$ and $^{13}C\{^{1}H\}$ solution NMR spectroscopic data were obtained at ambient temperature using a Bruker Avance 300 III (operating at 300 MHz for $^{1}H$ and 75 MHz for $^{13}C\{^{1}H\}$), or a Bruker Avance 500 NMR spectrometer (operating at 500 MHz for $^{1}H$, 125 MHz for $^{13}C\{^{1}H\}$). Chemical shifts ($\delta$) were referenced to solvent peaks as follows. $^{1}H$ NMR spectra were referenced via residual proton resonances of $CD_2Cl_2$ (5.32 ppm) and $CD_3OD$ (3.31 ppm). $^{13}C$ spectra were referenced to $CD_2Cl_2$ (53.84 ppm) and $CD_3OD$ (49.00 ppm).

Elemental analyses were performed on an Elementar vario MICRO cube elemental analyser. As is common for related organo-$B(Aryl)_2$ compounds, carbon analyses of **3**, **4** and **5** were up to 2.3% below the calculated value, while hydrogen, nitrogen and sulphur analyses were satisfactory. This has been ascribed previously to the formation of boron carbide.[6] High resolution mass spectrometry (HRMS) was performed with a Thermo Fisher Scientific Exactive Plus Orbitrap MS System. ESI measurements were performed with a HESI source at 50 °C. APCI measurements were performed with an ACPI source and Corona needle at 400 °C, unless otherwise noted.

**Single-Crystal X-Ray Diffraction.** Crystals suitable for single-crystal X-ray diffraction were selected, coated in perfluoropolyether oil, and mounted on MiTeGen sample holders. Diffraction data of **3N** and **4N** were collected on a Bruker X8-APEX II diffractometer with a CCD area detector and multi-layer mirror monochromated Mo-$K_\alpha$ radiation. Diffraction data of **C**

were collected on a RIGAKU OXFORD DIFFRACTION XtaLAB Synergy diffractometer with a semiconductor HPA-detector (HyPix-6000) and multi-layer mirror monochromated Cu-$K_\alpha$ radiation. The crystals were cooled using an Oxford Cryostream or Bruker Kryoflex low-temperature device. Data were collected at 100 K (**3N**, **4N**) or 120 K (**C**). The images were processed and corrected for Lorentz-polarization effects and absorption as implemented in the Bruker software packages (**3N**, **4N**) or in the CrysAlis$^{Pro}$ software (**C**), respectively. The structures were solved using the intrinsic phasing method (SHELXT)[7] and Fourier expansion technique. All non-hydrogen atoms were refined in anisotropic approximation, with hydrogen atoms 'riding' in idealized positions, by full-matrix least squares against $F^2$ of all data, using SHELXL[8] software and the SHELXLE graphical user interface.[9] For both, **3N** and **4N**, each asymmetric unit contains one ethylacetate molecule disordered over three positions, while for **C** it contains two hexane molecules disordered over two positions each in addition to the main molecules. Diamond[10] software was used for graphical representation. Crystal data and experimental details are listed in Table S3; full structural information has been deposited with Cambridge Crystallographic Data Centre. CCDC-1997113 (**3N**), 1997114 (**4N**), and 1997115 (**C**).

**Linear Optical Properties.** All measurements were performed in standard quartz cuvettes (1 cm x 1 cm cross-section) under ambient conditions. UV-visible absorption spectra were recorded using an Agilent 8453 diode array UV-visible spectrophotometer. The molar extinction coefficients were calculated from three independently prepared samples in hexane (**3N**-**5N**) and MeCN and $H_2O$ (**3**-**5**) solution. The emission spectra were recorded using an Edinburgh Instruments FLSP920 spectrometer equipped with a double monochromator for both excitation and emission, operating in right-angle geometry mode, and all spectra were fully corrected for the spectral response of the instrument. All solutions used in photophysical measurements had a concentration lower than $5 \times 10^{-6}$ M to minimize inner filter effects during fluorescence measurements. The **fluorescence quantum yields** were measured using a calibrated integrating sphere (inner diameter: 150 mm) from Edinburgh Instruments combined with the FLSP920 spectrometer described above. For solution-state measurements, the longest-wavelength absorption maximum of the compound in the respective solvent was chosen as the excitation wavelength, unless stated otherwise. **Fluorescence lifetimes** were recorded using the time-correlated single-photon counting (TCSPC) method using an Edinburgh Instruments FLS980 spectrometer equipped with a high speed photomultiplier tube positioned after a single emission monochromator. Measurements were made in right-angle geometry mode, and the emission was collected through a polarizer set to the magic angle. Solutions were excited with a pulsed diode laser at a wavelength of 376.6 nm (**3N**, **4N**, **3**, **4**) and 472.6 nm (**5N**, **5**) at repetition rates of 10 or 20 MHz, as appropriate. The full-width-at-half-maximum (FWHM) of the pulse from the diode laser was ca. 80 ps with an instrument response

function (IRF) of ca. 230 ps FWHM and ca. 200 ps with an instrument response function (IRF) of ca. 1120 ps FWHM, respectively. The IRFs were measured from the scatter of an aqueous suspension of Ludox at the excitation wavelength. Decays were recorded to 10 000 counts in the peak channel with a record length of 8 192 channels. The band pass of the emission monochromator and a variable neutral density filter on the excitation side were adjusted to give a signal count rate of <60 kHz. Iterative reconvolution of the IRF with one decay function and non-linear least-squares analysis were used to analyse the data. The quality of all decay fits was judged to be satisfactory, based on the calculated values of the reduced $\chi^2$ and Durbin-Watson parameters and visual inspection of the weighted residuals.

**Optical Properties in Sodium Cacodylate.** UV-visible absorption spectra were recorded on a Varian Cary 100 Bio spectrometer; excitation and emission spectra were recorded on a Varian Cary Eclipse fluorimeter

**Study of Interactions with DNA and RNA.** Polynucleotides were purchased as noted: poly dGdC – poly dGdC, poly dAdT – poly dAdT, poly A – poly U, poly A, poly G, poly C, poly U (Sigma), *calf thymus* (ct)-DNA (Aldrich) and dissolved in sodium cacodylate buffer, $I$ = 0.05 M, pH=7.0. The ct-DNA was additionally sonicated and filtered through a 0.45 mm filter to obtain mostly short (ca. 100 base pairs) rod-like B-helical DNA fragments.[11] The polynucleotide concentration was determined spectroscopically[12] as the concentration of phosphates (corresponds to $c$(nucleobase)). **Thermal melting experiments** were performed on a Varian Cary 100 Bio spectrometer in quartz cuvettes (1 cm). The measurements were carried out in aqueous buffer solution at pH 7.0 (sodium cacodylate buffer $I$ = 0.05 M). Thermal melting curves for ds-DNA, ds-RNA and their complexes with **3-6** were determined by following the absorption change at 260 nm as a function of temperature.[13] $Tm$ values are the midpoints of the transition curves determined from the maximum of the first derivative and checked graphically by the tangent method. The $\Delta Tm$ values were calculated subtracting $Tm$ of the free nucleic acid from $Tm$ of the complex. Every $\Delta Tm$ value reported here was the average of at least two measurements. The error in $\Delta Tm$ is $\pm$ 0.5 °C. **Fluorimetric titrations** were performed by adding portions of polynucleotide solution into the solution of the compound studied and excitation wavelengths of $\lambda_{exc}$ > 300 nm were used to avoid absorption of excitation light by added polynucleotides. After mixing polynucleotides with the compound, equilibrium was reached in less than 120 s. Fluorescence spectra were analyzed at an excess of DNA/RNA ($r_{\mathbf{[compd]}/[DNA]}$ <0.2) to assure one dominant binding mode. To obtain binding constants ($Ks$), titration data were processed by means of non-linear fitting to the Scatchard equation (McGhee, von Hippel formalism),[14, 15] which gave values of the ratio of [bound com-pound] / [polynucleotide] in the range 0.1–0.3, but for easier comparison, all $Ks$ values were re-calculated for the fixed n = 0.25 (for ds-DNA/RNA) or 0.5 (for ss-RNA). Calculated values for

*Ks* have satisfactory correlation coefficients (>0.99). For fluorimetric titrations fluorescence spectra were recorded on Varian Cary Eclipse fluorimeter in quartz cuvettes (1 cm) by adding portions of polynucleotide solution into the solution of the studied compound. **Circular dichroism (CD)** spectra were recorded on a JASCO J-815 spectropolarimeter at room temperature using appropriate 1 cm path quartz cuvettes with scanning speed of 200 nm/min. A background spectrum of the buffer was subtracted from each spectrum and each spectrum was the result of three accumulations. CD experiments were performed by adding portions of a stock solution of the compound into the solution of polynucleotide ($c = 2\times10^{-5}$ M).

**Molecular Modelling.** Compound **5** was optimized using Gaussian, version g09-D0.1 software, at the B3LYP/6-31G(d) level of theory, and the parametrization procedure was performed using the Antechamber module within the AMBER16 program suite wherein the Mullikan charges were used as the partial atomic charges. Parametrization, energy minimization, and molecular dynamics (MD) simulations of the complexes between compound **5** and DNA were performed using the AMBER16 suite of programs.[16] The solutes were prepared using the AMBER16 utility program tLeap wherein ligand and DNA were parametrized within general AMBER force field gaff[17] and ff99bsc0[18] force fields, respectively. For details of the parametrization procedure see our previous work.[19] Initial conformations were prepared in PyMOL (The PyMOL Molecular Graphics System, Version 1.7 SchroÈdinger, LLC), wherein compound **5** was docked into the DNA minor groove using DNA-1 complex[19] as a template. The systems were solvated in the truncated octahedron box filled with TIP3P water molecules[20, 21] whereas the sodium ions were added to achieve electroneutrality. The complexes were minimized, equilibrated and simulated for 300 ns by the programs sander.MPI and pmemd.MPI. The simulations were performed using periodic boundary conditions (PBC). The particle mesh Ewald (PME) method was used for calculation of the long-range electrostatic interactions, and in the direct space the pairwise interactions were calculated within the cut-off distance of 8 Å. The solvated complexes were geometry optimized by using steepest descent and conjugate gradient methods (altogether 7000 steps), and equilibrated for 100 ps with time step of 1 fs. During the first stage of equilibration (30 ps), the temperature was linearly increased from 0 to 300 K and the volume was held constant. In the second stage (NPT ensemble with T and P about 300 K and 1 atm, respectively) the solution density was optimized. The equilibrated complexes were subjected to productive molecular dynamics simulation using NPT conditions and a time step of 2 fs. The temperature was held constant using a Langevin thermostat[22] with a collision frequency of 0.2 ps, and the pressure was regulated by a Berendsen barostat.[23]

**Raman and SERS measurements.** Raman and SERS spectra were measured on a Bruker Equinox 55 interferometer equipped with a FRA 106/S Raman module using Nd-YAG laser

excitation at 1064 nm of 500 mW laser power. The spectra were acquired in the 3500–100 $cm^{-1}$ spectral range at 4 $cm^{-1}$ resolution. A total of 512 and 128 scans were averaged for a Raman and SERS spectrum, respectively. Quartz cuvettes were used for handling samples. Solutions of the bis-triarylborane compounds were prepared by dissolution of the solid substance in water, the concentration of which was determined spectroscopically using the respective molar absorption coefficient. The stock solutions were further on diluted in water to obtain solutions of $1 \times 10^{-4}$ M (**3**, **4** and **5**) and $2 \times 10^{-3}$ M (**6**), used for Raman measurements. For the SERS measurement a silver colloidal suspension was used as the SERS active substrate, prepared by reduction of silver nitrate with trisodium citrate according to the procedure described in our previous work.[2] The resulting colloidal suspension was gray colored, characterized by a maximum at 416 nm in the UV/Vis spectrum, pointing to the typical silver plasmon resonance frequency. The pH value of the silver colloid was 7.5. Working samples for the SERS measurements were prepared by dissolution of bis-triarylborane compound solution in an appropriate volume of water, followed by addition of 400 μL of the silver colloid. The total sample volume was 500 μL. For the concentration dependent measurements the final concentrations of **3**-**6** in the Ag colloid were $1 \times 10^{-7}$, $5 \times 10^{-7}$, $1 \times 10^{-6}$ and $5 \times 10^{-6}$ M. To measure the SERS spectra of the complexes of **3**-**5** with ct-DNA, samples were prepared in the buffered solution by mixing the appropriate volume of the bis-triarylborane compound with ct-DNA in molar ratios $r_{[compound]/[ct\text{-}DNA]}$ = 1, 0.2 and 0.1, followed by addition of the of 400 μL silver colloid. The total volume of the working sample was 500 μL and the final concentration of the bis-triarylborane compound was $1 \times 10^{-6}$ M.

**Theoretical Studies.** All calculations (DFT and TD-DFT) were carried out with the Gaussian 16 (16.A.03)[24] program package and were performed on a parallel cluster system. GaussView (6.0.16), Avogadro (1.2.0)[25] and multiwfn[26] were used to visualize the results, to measure calculated structural parameters, and to plot orbital surfaces (isovalue: ± 0.030 $[e\ a_0^{-3}]^{1/2}$). The ground-state geometries were optimized using the B3LYP functional[27] in combination with the 6-31G(d) basis set.[28, 29] The ultrafine integration grid and no symmetry constraints were used for all molecules. Frequency calculations were performed on the optimized structures to confirm them to be local minima showing no negative (imaginary) frequencies. Based on these optimized structures, the lowest-energy vertical transitions (gas-phase) were calculated (singlets, 25 states) by TD-DFT, using the CAM-B3LYP functional in combination with the 6-31G(d) basis set.[28, 29] For calculations of the Raman spectra the ground state geometries were optimized at the B3LYP/6-31+G(d, p) level of theory. The resulting frequencies were multiplied by a scaling factor of 0.964 (as suggested by the Computational Chemistry Comparison and Benchmark database (https://cccbdb.nist.gov/vibscalejustx.asp)).

# Synthesis

**5,5'-Bis[4-(bis(4-(*N*,*N*-dimethylamino)-2,6-dimethylphenyl)boryl)-3,5-dimethyl-phenylethynyl]-2,2' bithiophene (3N)**

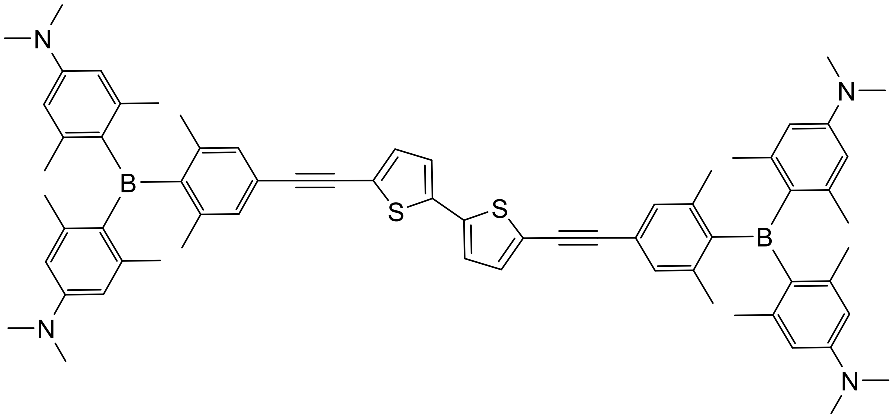


Bis[4-(*N*,*N*-dimethylamino)-2,6-dimethylphenyl]-(4-ethynyl-2,6-dimethylphenyl)borane (500 mg, 1.15 mmol, 2.1 eq), 5,5'-dibromo-2,2'-bithiophene (178 mg, 0.55 mmol, 1 eq), $Pd(PPh_3)_2Cl_2$ (8 mg, 11 $\mu$mol, 2 mol%) and CuI (2 mg, 11 $\mu$mol, 2 mol%) were dissolved in THF (10 mL) and $NEt_3$ (5 mL) was added. The reaction mixture was stirred at r.t. for 19 h, until the starting materials were consumed according to TLC (1% $NEt_3$ in hexane : EtOAc 4:1). After removing the solvent, the solid was purified by automated flash column chromatography (Biotage SNAP cartridge KP-Sil 50 g; 1% $NEt_3$ additive to 5% EtOAc in hexane). The solid was dissolved in $CH_2Cl_2$ and precipitated with MeOH to yield compound **3N** as a yellow solid (165 mg, 29%).

**$^1$H NMR** (300 MHz, $CD_2Cl_2$): $\delta$ = 7.17 (d, $J$ = 4 Hz, 2H), 7.11 (d, $J$ = 4 Hz, 2H), 7.07 (m, 4H), 6.31 (s, 8H), 2.95 (s, 24H), 2.03 (s, 12H), 2.00 (s, 12H), 1.93 (s, 12H) ppm.

**$^{13}$C{$^1$H} NMR** (75 MHz, $CD_2Cl_2$): $\delta$ = 151.8, 151.4, 143.4, 142.9, 140.8, 138.2, 135.8, 133.1, 130.2, 124.5, 123.3, 122.2, 111.9, 111.9, 95.9, 82.3, 40.1, 24.1, 23.8, 22.8 ppm.

**HRMS** (APCI) m/z: $[M+H]^+$ found: 1035.5758; calc. for $[C_{68}H_{77}B_2N_4S_2]^+$: 1035.5770 ($|\Delta|$ = 1.16 ppm).

**Elemental analysis** Calc. (%) for $C_{68}H_{76}B_2N_4S_2$: C 78.90, H 7.40, N 5.41, S 6.19; found: C 78.57, H 7.51, N 5.34, S 6.05.

**5,5'-Bis[4-(*bis*(4-(*N*,*N*,*N*-trimethylammonium)-2,6-dimethylphenyl)boryl)-3,5-dimethyl-phenylethynyl]-2,2'-bithiophene tetratriflate (3)**

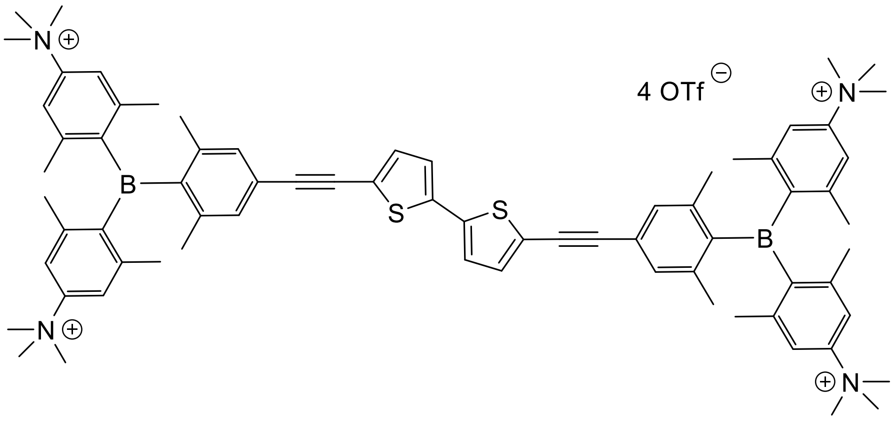


Compound **3N** (15.5 mg, 15.0 μmol, 1 eq) was dissolved in $CH_2Cl_2$ (2 mL). Methyl triflate (7.63 μL, 67.4 μmol, 4.5 eq) was added and the reaction was stirred at r.t. for 42 h. The precipitate was collected by filtration and washed with $CH_2Cl_2$ (3 x 5 mL) to afford compound **3** as a yellow solid (22 mg, 88%).

**$^1$H NMR** (500 MHz, $CD_3OD$): $\delta$ = 7.58 (m, 8H), 7.27 (d, $J$ = 4 Hz, 2H), 7.25 (d, $J$ = 4 Hz, 2H), 7.19 (m, 4H), 3.66 (s, 36H), 2.22 (s, 12H), 2.16 (s, 12H), 2.04 (s, 12H) ppm.

**$^{13}$C{$^1$H} NMR** (125 MHz, $CD_3OD$): $\delta$ = 149.8, 149.0, 145.0, 144.6,142.4, 139.5, 134.7, 131.8, 126.6, 125.8, 123.6, 121.8 (q, $J$ = 318 Hz), 121.4, 120.3, 120.2, 95.4, 84.5, 57.5, 23.5, 23.5, 23.2 ppm.

**HRMS** (ESI pos) m/z: $[M\text{-}2OTf]^{2+}$ found: 696.2839; calc. for $[C_{74}H_{88}B_4N_4S_2F_6O_6]^{2+}$: 696.2833 (|Δ| = 0.86 ppm).

**Elemental analysis** Calc. (%) for $C_{76}H_{88}B_2F_{12}N_4O_{12}S_6$: C 53.97, H 5.24, N 3.31, S 11.37; found: C 51.70, H 5.47, N 3.08, S 10.62.

**1,4-Bis[4-(*bis*(4-(*N*,*N*-dimethylamino)-2,6-dimethylphenyl)boryl)-3,5-dimethyl-phenylethynyl]-benzene (4N)**

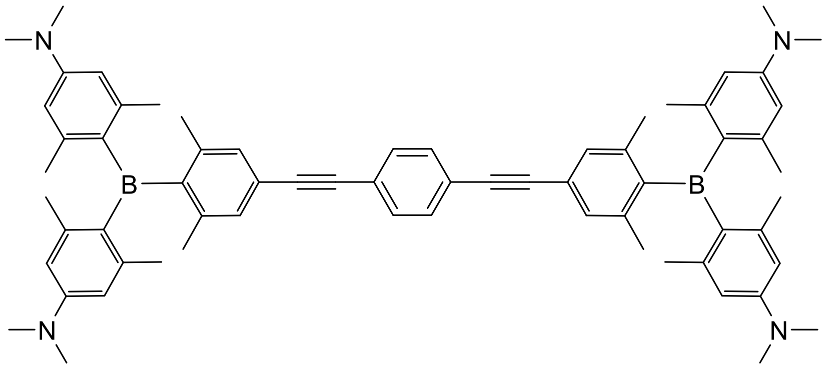


Bis[4-(*N*,*N*-dimethylamino)-2,6-dimethylphenyl]-(4-ethynyl-2,6-dimethylphenyl)borane (500 mg, 1.15 mmol, 2.2 eq), 1,4-diiodobenzene (172 mg, 0.52 mmol, 1 eq), $Pd(PPh_3)_2Cl_2$ (7.2 mg, 11 $\mu$mol, 2 mol%) and CuI (2 mg, 11 $\mu$mol, 2 mol%) were dissolved in THF (10 mL) and $NEt_3$ (5 mL) was added. The reaction mixture was stirred at r.t. for 20 h, until the starting materials were consumed according to TLC (10% EtOAc in hexane). After removing the solvent, the solid was purified by automated flash column chromatography (Biotage SNAP cartridge KP-Sil 50 g; 5% EtOAc in hexane). The solid was dissolved in EtOAc and precipitated with MeOH and then recrystallized from EtOAc to yield compound **4N** as a yellow solid (234 mg, 48%).

**$^1$H NMR** (300 MHz, $CD_2Cl_2$): $\delta$ = 7.49 (s, 4H), 7.09 (s, 4H), 6.31 (s, 8H), 2.95 (s, 24H), 2.03 (s, 12 H), 2.00 (s, 12H),1.93 (s, 12H) ppm.

**$^{13}$C{$^1$H} NMR** (75 MHz, $CD_2Cl_2$): $\delta$ = 151.8, 151.2, 143.4, 142.9, 140.7, 135.8, 131.9, 130.5, 123.6, 122.6, 111.9, 111.9, 92.5, 89.0, 40.1, 24.1, 23.8, 22.8 ppm.

**HRMS** (APCI) m/z: found $[M+H]^+$: 947.6336; calc. for $[C_{66}H_{77}B_2N_4]^+$: 947.6329 (|Δ| = 0.74 ppm).

**Elemental analysis** Calc. for $C_{66}H_{76}B_2N_4$: C 83.71, H 8.09, N 5.92; found: C 83.52, H 8.24, N 5.91.

**1,4-Bis[4-(*bis*(4-(*N,N,N*-trimethylammonium*)*-2,6-dimethylphenyl)boryl)-3,5-dimethyl-phenylethynyl]-benzene tetratriflate (4)**

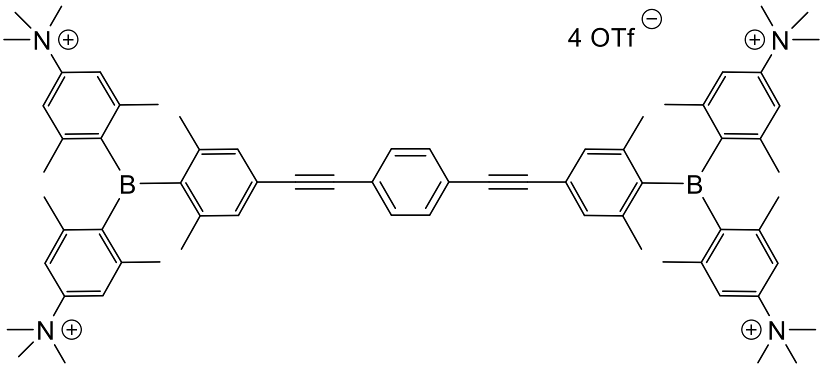


Compound **4N** (15.0 mg, 15.8 *μ*mol, 1 eq) was dissolved in $CH_2Cl_2$ (2 mL). Methyl triflate (8.96 *μ*L, 79.2 *μ*mol, 5.0 eq) was added and the reaction was stirred at r.t. for 48 h. The precipitate was collected by filtration and washed with $CH_2Cl_2$ (3 x 5 mL) to afford compound **4** as a colorless solid (21 mg, 84%).

**$^1$H NMR** (500 MHz, $CD_3OD$): $\delta$ = 7.58 (m, 8H), 7.54 (s, 4H), 7.22 (m, 4H), 3.66 (s, 36H), 2.22 (s, 12H) 2.16 (s, 12H), 2.05 (s, 12H) ppm.

**$^{13}$C{$^1$H} NMR** (75 MHz, $CD_3OD$): $\delta$ = 149.8, 149.1, 146.9, 144.9, 144.6, 142.3, 132.8, 132.1, 126.9, 124.5, 121.8 (q, *J* = 318 Hz), 120.2, 92.1, 91.1, 57.5, 23.5, 23.5, 23.2 ppm.

**HRMS** (ESI pos) m/z: $[M\text{-}2OTf]^{2+}$ found: 652.3112; calc. for $[C_{74}H_{88}B_4N_4S_2F_6O_6]^{2+}$: 652.3112 (|Δ| = 0 ppm).

**Elemental analysis** Calc. (%) for $C_{74}H_{88}B_2F_{12}N_4O_{12}S_4$: C 55.43, H 5.53, N 3.49, S 8.00; found: C 54.09, H 5.68, N 3.61, S 7.65.

**9,10-Bis[4-(*bis*(4-(*N*,*N*-dimethylamino)-2,6-dimethylphenyl)boryl)-3,5-dimethyl-phenylethynyl]-anthracene (5N)**

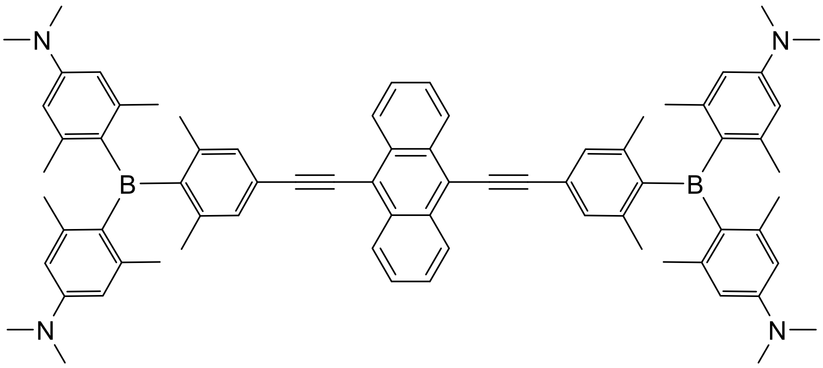

Bis[4-(*N*,*N*-dimethylamino)-2,6-dimethylphenyl]-(4-ethynyl-2,6-dimethylphenyl)borane (500 mg, 1.15 mmol, 2.2 eq), 9,10-diiodoanthracene (225 mg, 0.52 mmol, 1 eq), $Pd(PPh_3)_2Cl_2$ (18.2 mg,22 *μ*mol, 5 mol%) and CuI (5 mg, 22 *μ*mol, 5 mol%) were dissolved in $NEt_3$ (50 mL). The reaction mixture was stirred at r.t. for 3d, until the starting materials were consumed according to TLC (10% EtOAc in hexane). After removing the solvent, the solid was purified by automated flash column chromatography (Biotage SNAP cartridge KP-Sil 10 g; 10% EtOAc in hexane). The solid was recrystallized from EtOAc to yield compound **5N** as a red-orange solid (193mg, 35%).

**$^1$H NMR** (300 MHz, $CD_2Cl_2$): $\delta$ = 8.73 (m, 4H), 7.67 (m, 4H), 7.37 (m, 4H), 6.34 (s, 8H), 2.97 (s, 24H), 2.13 (s, 12H), 2.05 (s, 12H), 2.00 (s, 12H) ppm.

**$^{13}$C{$^1$H} NMR** (75 MHz, $CD_2Cl_2$): $\delta$ = 151.8, 151.6, 143.5, 143.0, 140.9, 135.8, 132.4, 130.6, 127.7, 127.3, 122.9, 118.9, 111.9, 111.9, 104.2, 86.4, 40.2, 24.1, 23.9, 22.9 ppm.

**HRMS** (APCI) m/z: $[M+H]^+$ found: 1047.6634; calc. for $[C_{74}H_{81}B_2N_4]^+$: 1047.6642 (|Δ| = 0.76 ppm).

**Elemental analysis** Calc. (%) for $C_{74}H_{80}B_2N_4$: C 84.88, H 7.70, N 5.35; found: C 84.58, H 7.84, N 5.24.

**9,10-Bis[4-(*bis*(4-(*N*,*N*,*N*-trimethylammonium)-2,6-dimethylphenyl)boryl)-3,5-dimethyl-phenylethynyl]-anthracene tetratriflate (5)**

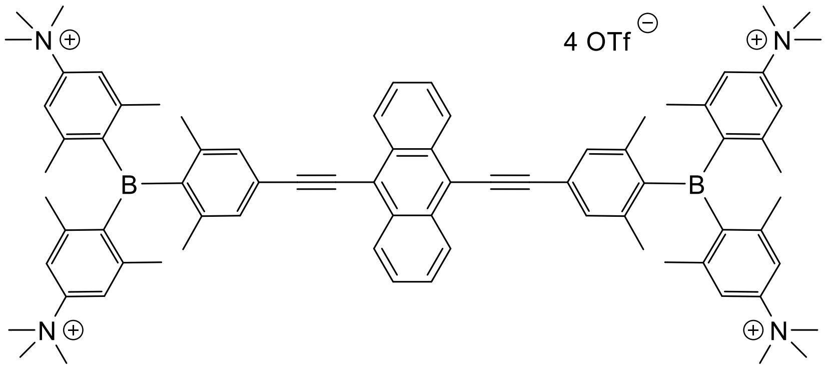


Compound **5N** (15.2 mg, 14.5 *μ*mol, 1 eq) was dissolved in $CH_2Cl_2$ (2 mL). Methyl triflate (7.39 *μ*L, 65.3 *μ*mol, 4.5 eq) was added and the reaction was stirred at r.t. for 48 h. The precipitate was collected by filtration and washed with $CH_2Cl_2$ (3 x 5 mL) to afford compound **5** as a red solid (16 mg, 64%).

**$^1$H NMR** (500 MHz, $CD_3OD$): $\delta$ = 8.70 (m, 4H), 7.73 (m, 4H), 7.60 (m, 8H), 7.49 (s, 4H), 3.68 (s, 36H), 2.28 (s, 12H), 2.19 (s, 12H), 2.14 (s, 12H) ppm.

**$^{13}$C{$^1$H} NMR** (125 MHz, $CD_3OD$): $\delta$ = 149.8, 149.1, 147.3, 145.0, 144.7, 142.6, 133.3, 132.1, 128.4, 128.1, 127.0, 121.8 (q, $J$ = 318 Hz), 120.3, 119.5, 103.9, 88.4, 57.5, 23.6, 23.5, 23.3 ppm.

**HRMS** (ESI pos) m/z: $[M\text{-}2OTf]^{2+}$ found: 702.3266; calc. for $[C_{74}H_{88}B_4N_4S_2F_6O_6]^{2+}$: 702.3269 (|Δ| = 0.43 ppm).

**Elemental analysis** Calc. (%) for $C_{82}H_{92}B_2F_{12}N_4O_{12}S_4$: C 57.82, H 5.44, N 3.29, S 7.53; found: C 56.74, H 5.32, N 3.29, S 7.16.

# NMR Spectra

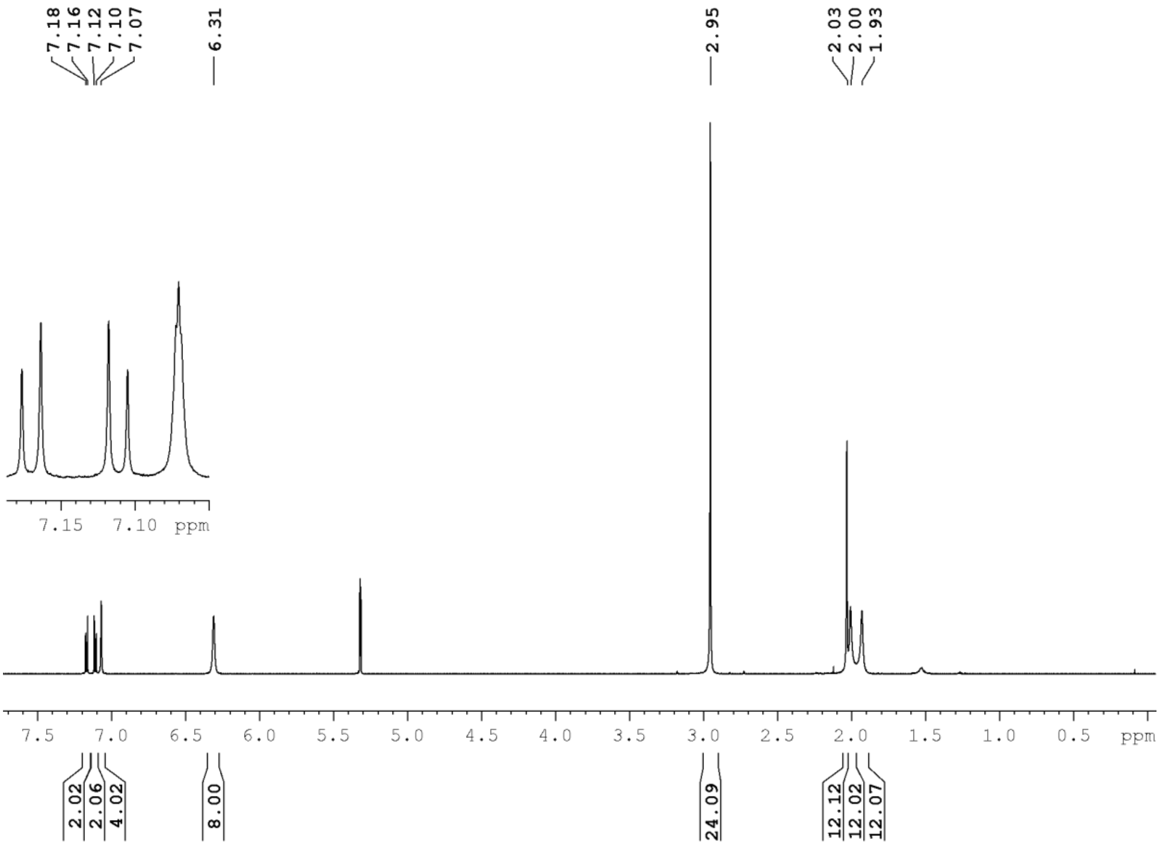


**Figure S1.** $^{1}$H NMR spectrum of **3N** in $CD_2Cl_2$ at 300 MHz.

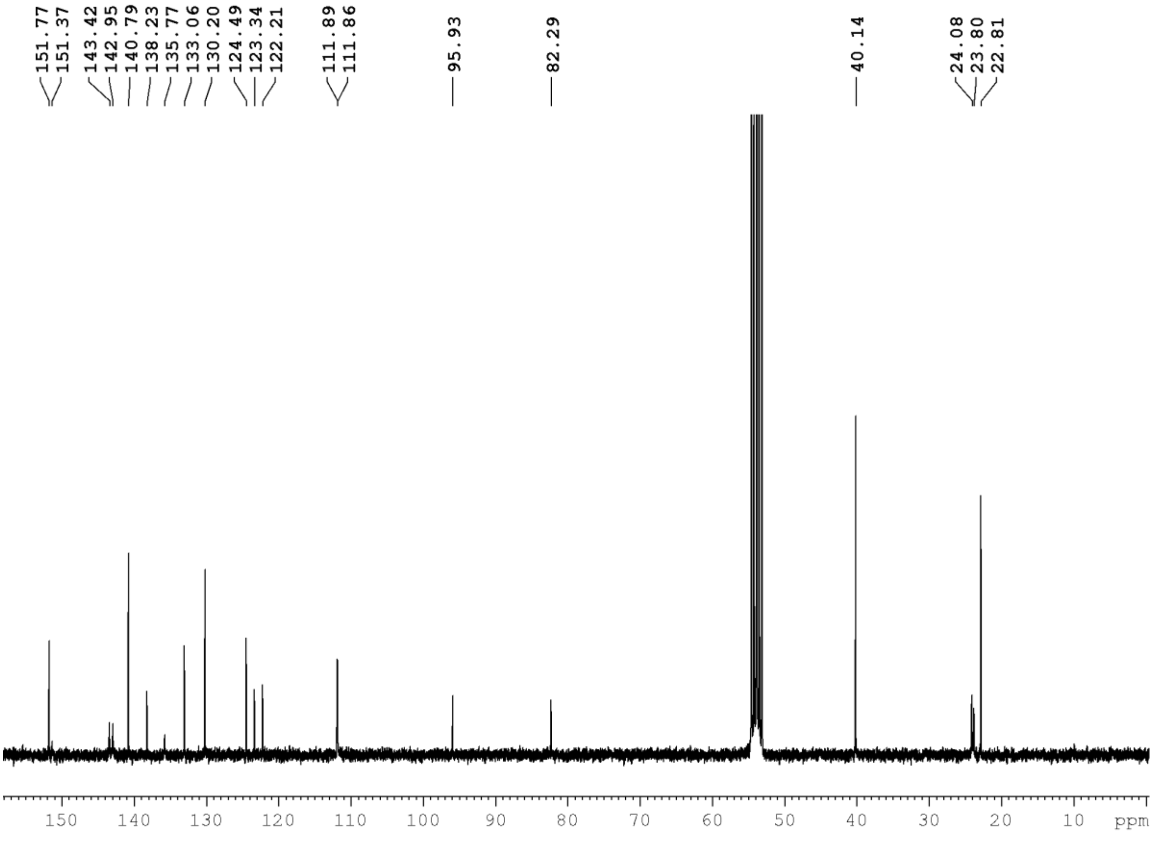


**Figure S2.** $^{13}$C{$^{1}$H} NMR spectrum of **3N** in $CD_2Cl_2$ at 75 MHz.

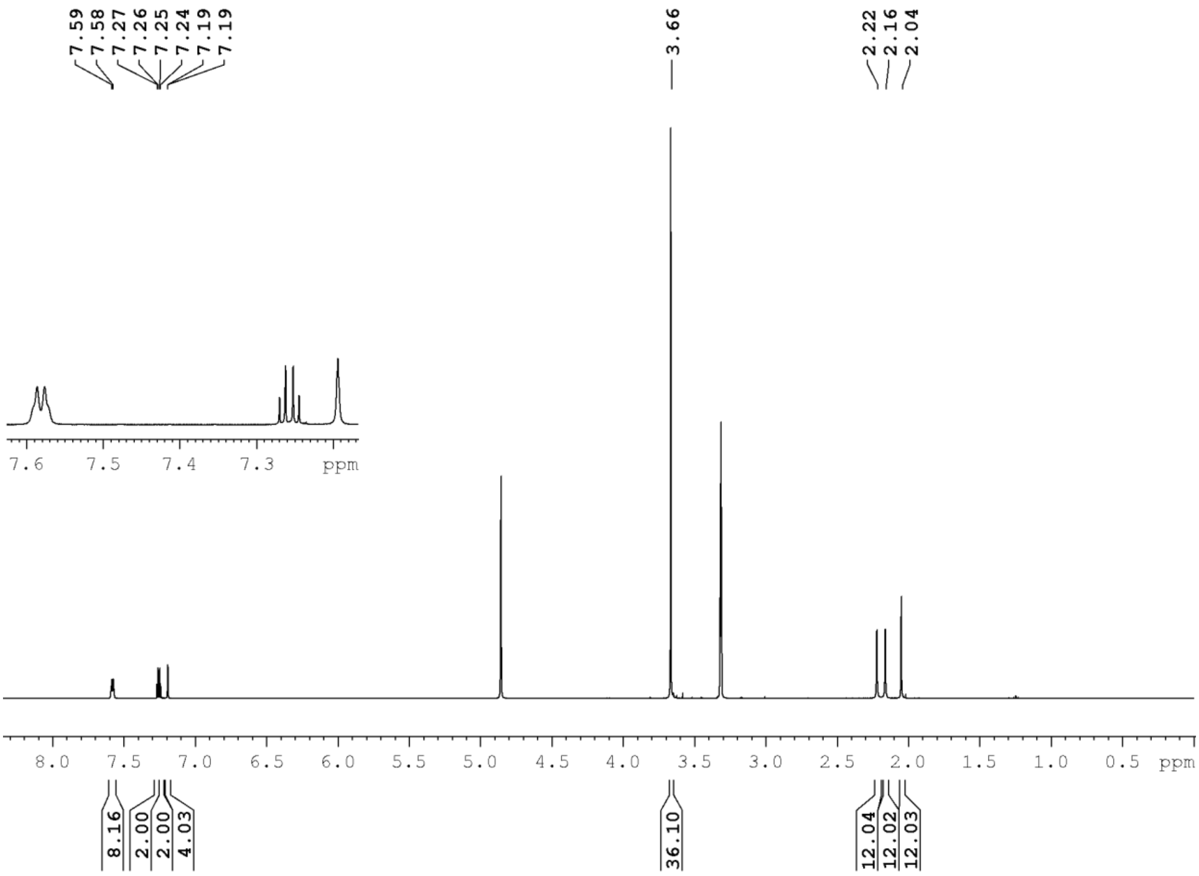


**Figure S3.** $^{1}$H NMR spectrum of **3** in $CD_3OD$ at 500 MHz.

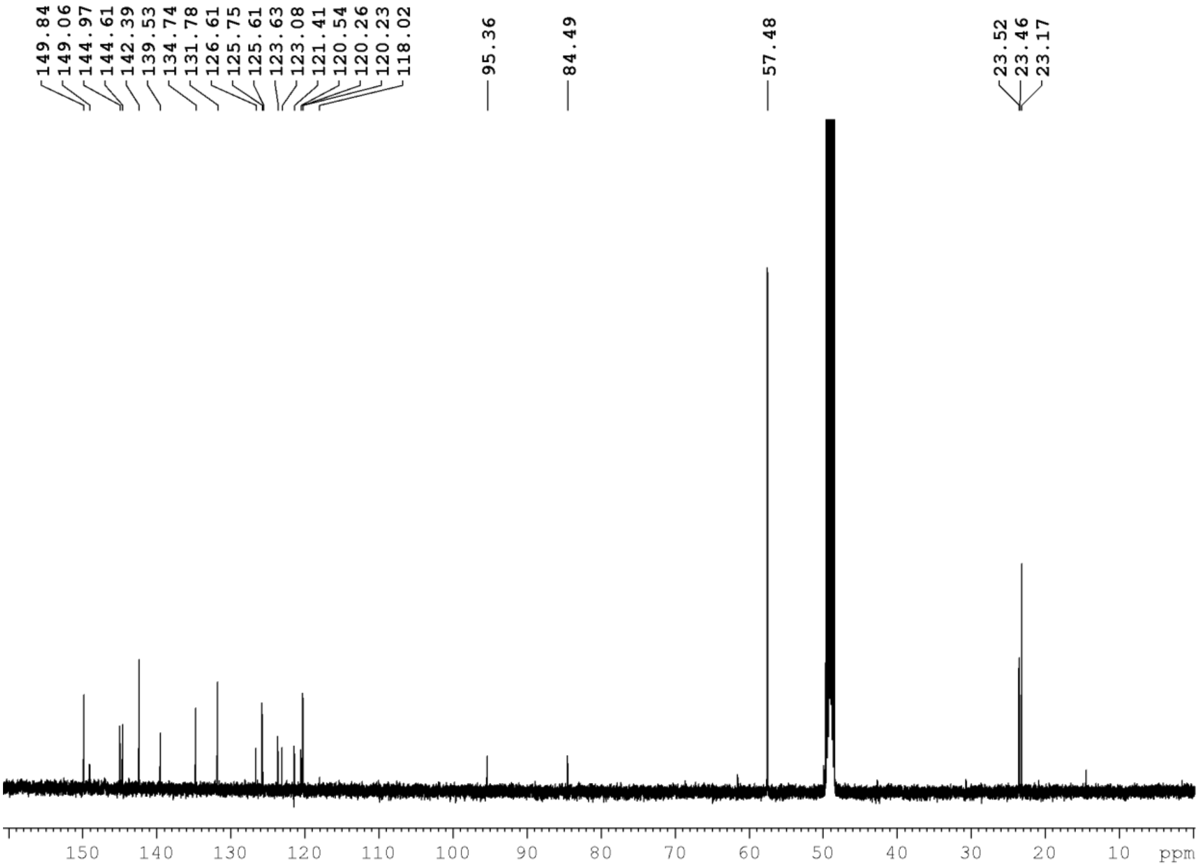


**Figure S4.** $^{13}C\{^{1}H\}$ NMR spectrum of **3** in $CD_3OD$ at 125 MHz.

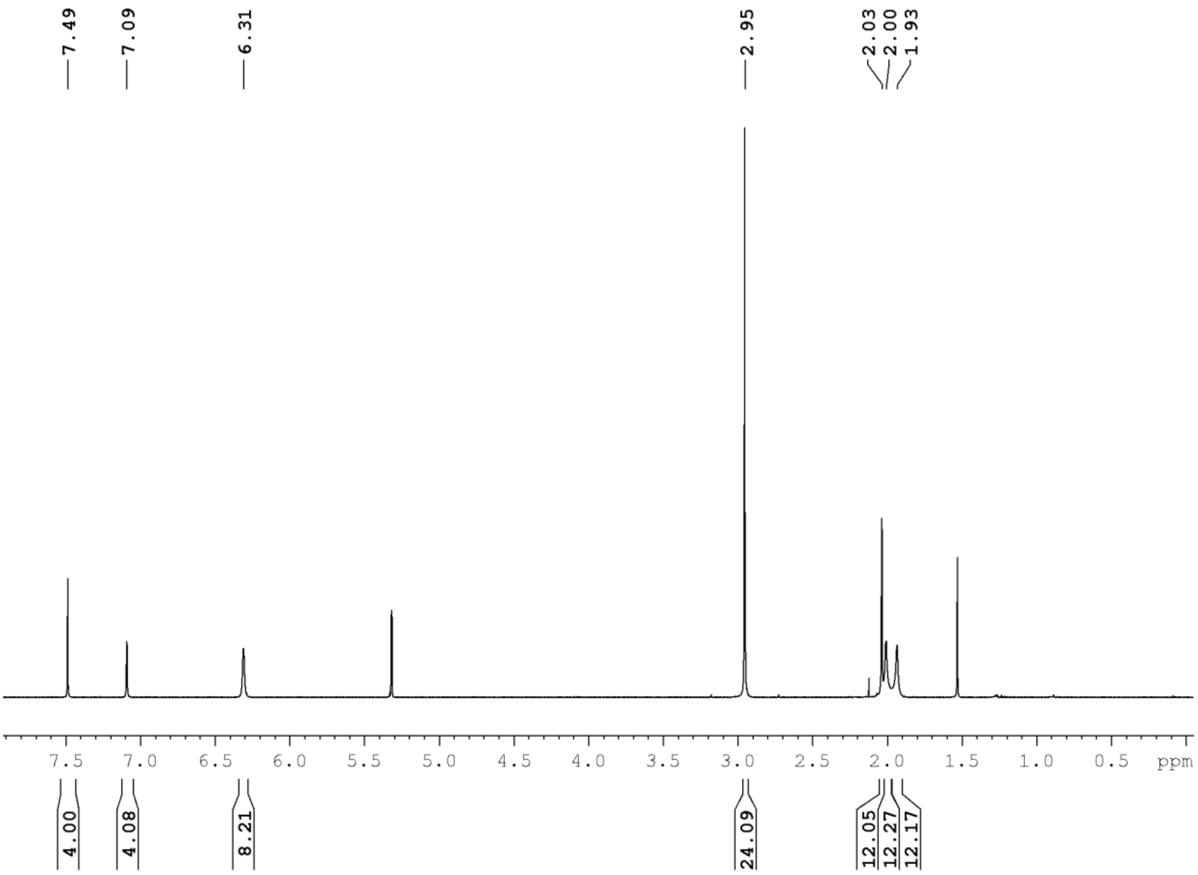


**Figure S5.** $^{1}H$ NMR spectrum of **4N** in $CD_2Cl_2$ at 300 MHz.

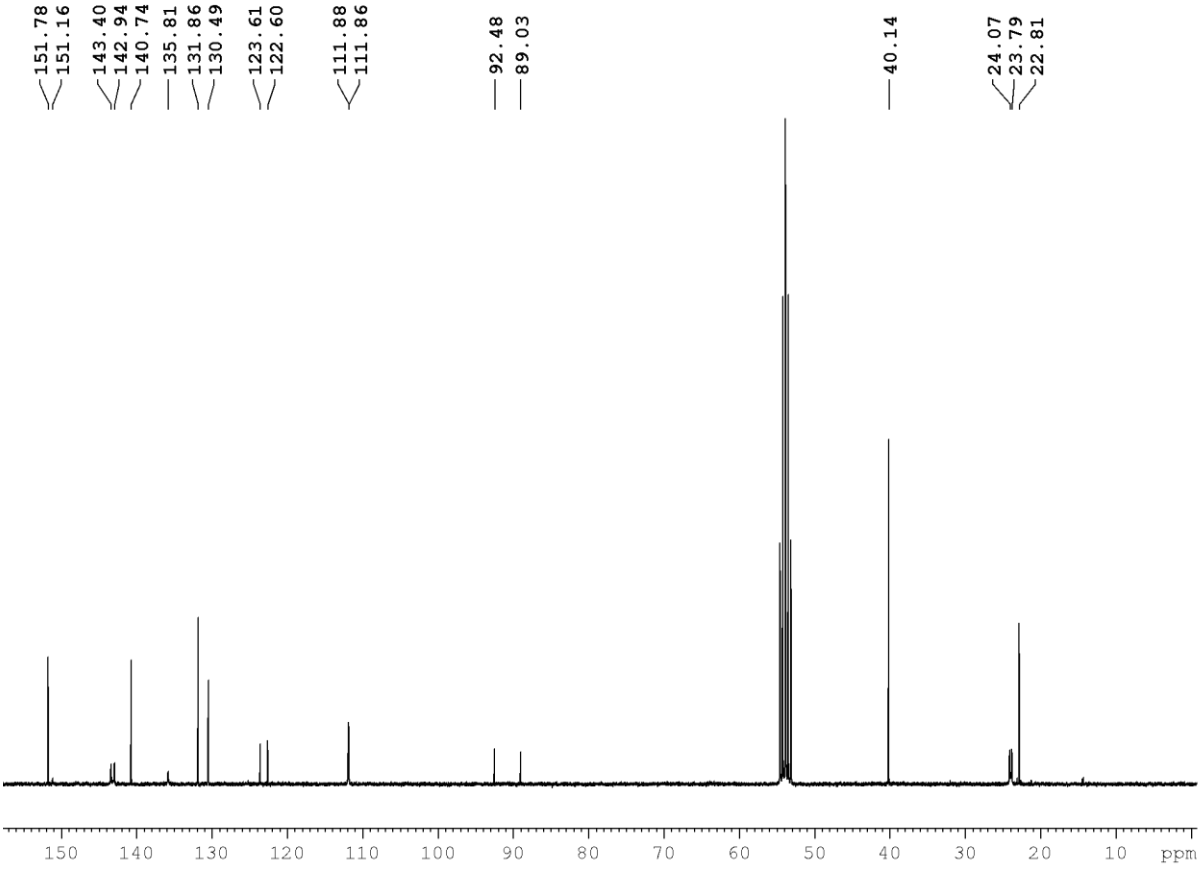


**Figure S6.** $^{13}C\{^{1}H\}$ NMR spectrum of **4N** in $CD_2Cl_2$ at 75 MHz.

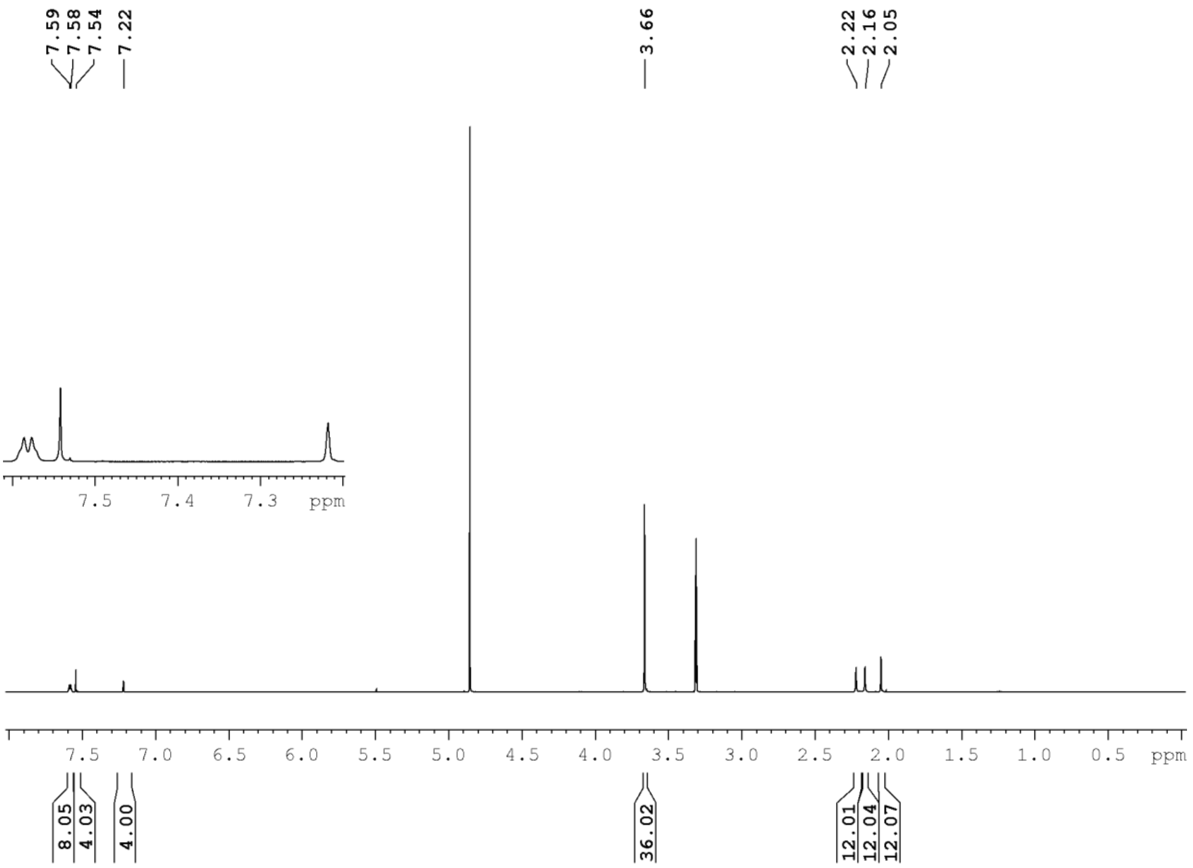

**Figure S7.** $^1$H NMR spectrum of **4** in $CD_3OD$ at 500 MHz.

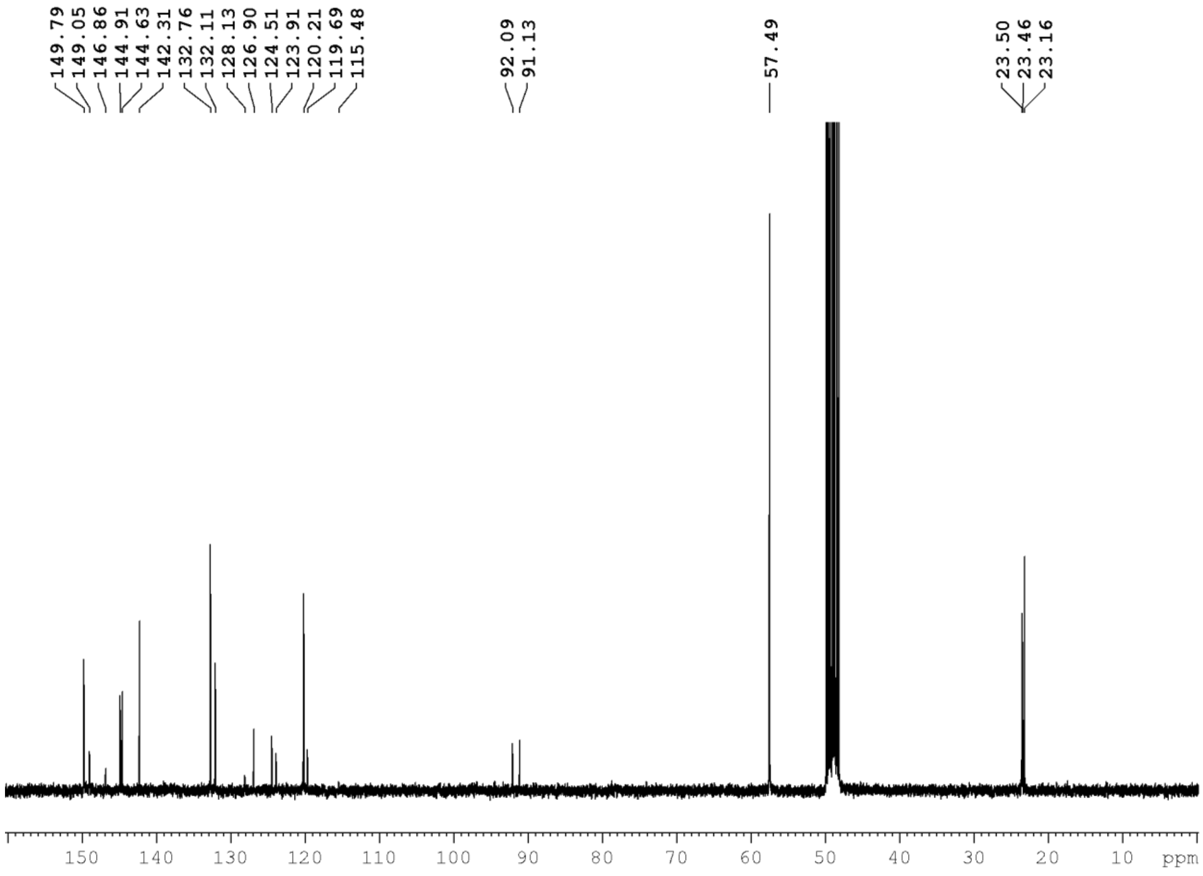

**Figure S8.** $^{13}C\{^1H\}$ NMR spectrum of **4** in $CD_3OD$ at 75 MHz.

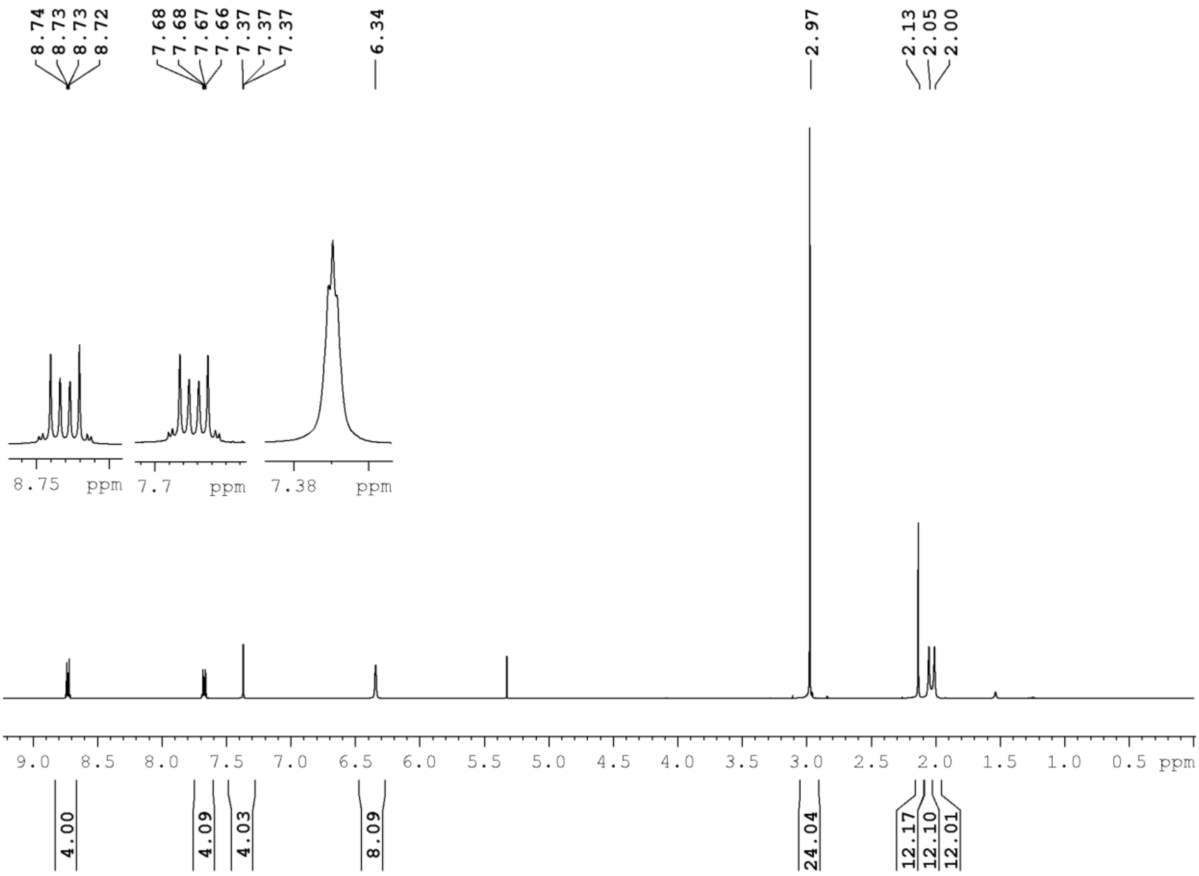


**Figure S9.** $^{1}H$ NMR spectrum of **5N** in $CD_2Cl_2$ at 300 MHz.

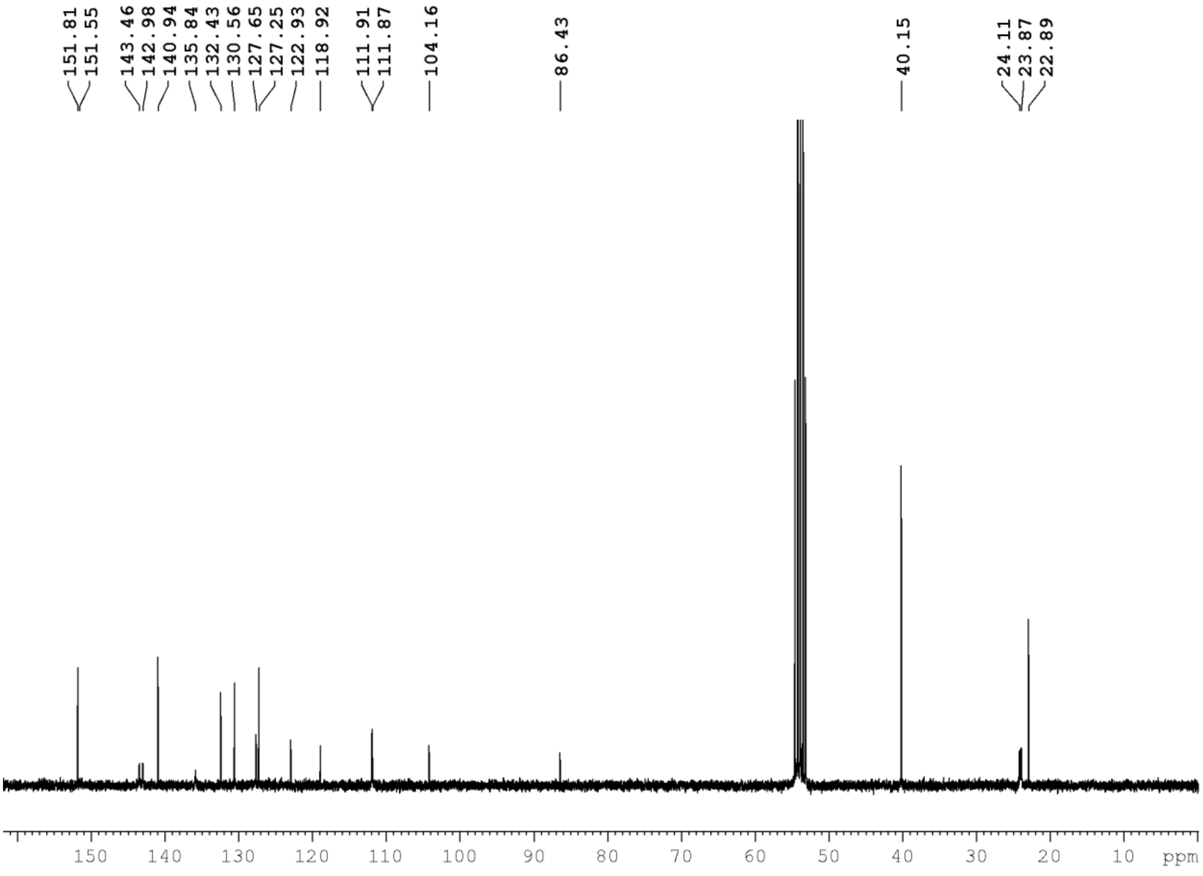


**Figure S10.** $^{13}C\{^{1}H\}$ NMR spectrum of **5N** in $CD_2Cl_2$ at 75 MHz.

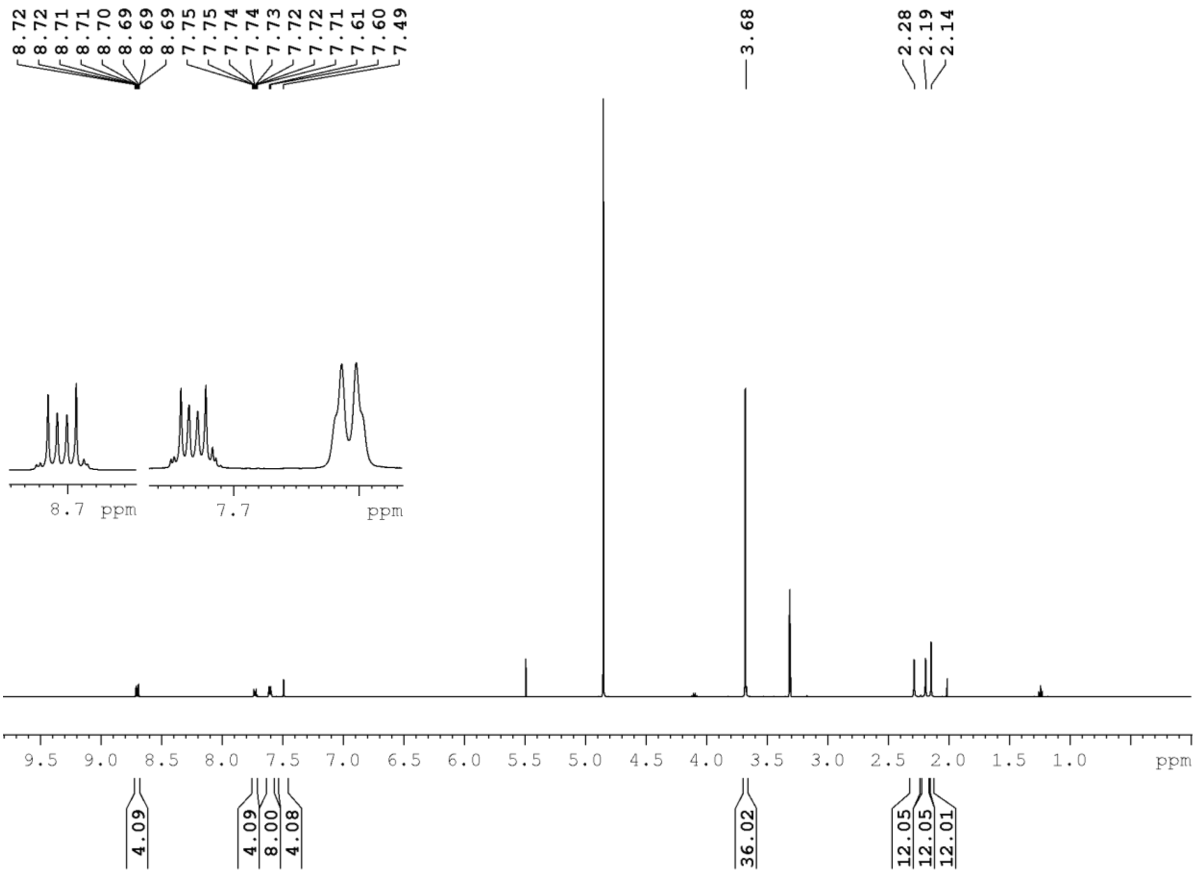


**Figure S11.** $^{1}H$ NMR spectrum of **5** in $CD_3OD$ at 500 MHz.

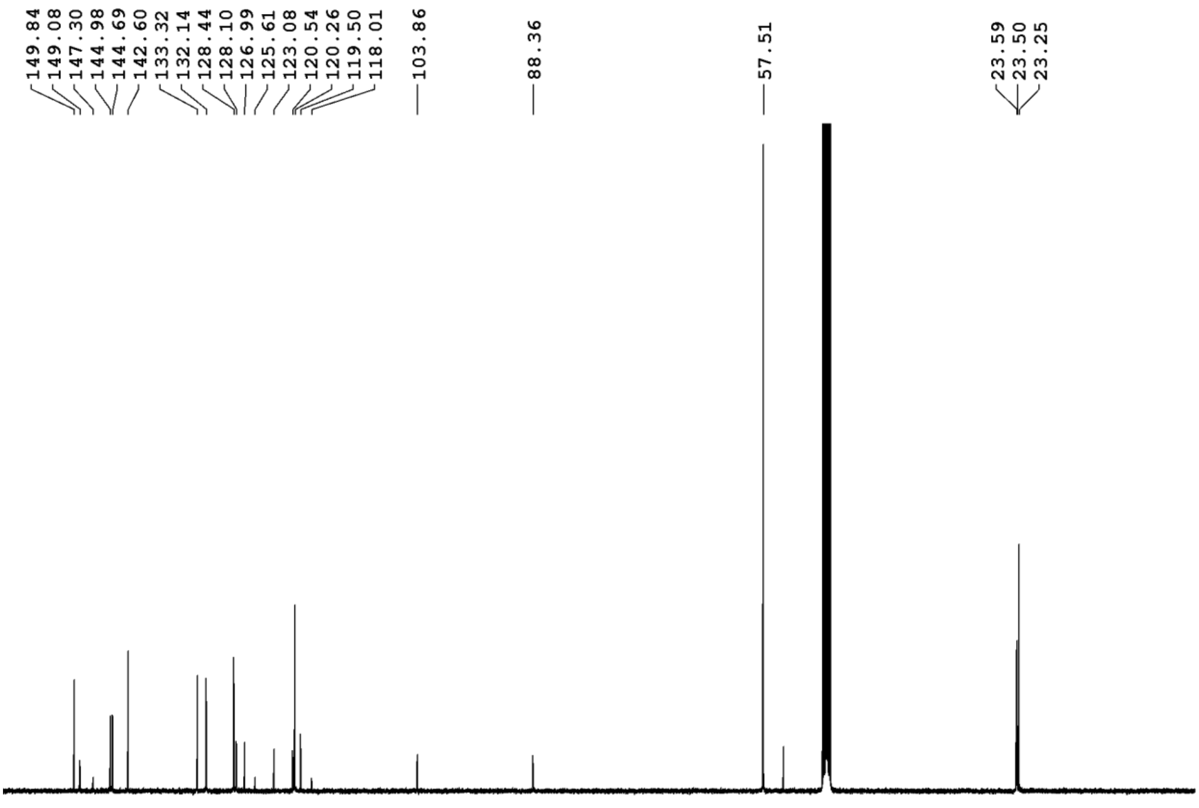


**Figure S12.** $^{13}C\{^{1}H\}$ NMR spectrum of **5** in $CD_3OD$ at 125 MHz.

# Single-Crystal X-Ray Diffraction

**Table S1.** Single-crystal X-ray diffraction data and structure refinements of compounds **3N** and **4N** of this study and of compound **C** of our earlier study.[2]

| Data | **3N** | **4N** | **C** |
|---|---|---|---|
| CCDC number | 1997113 | 1997114 | 1997115 |
| Empirical formula | $C_{68}H_{76}B_2N_4S_2\cdot C_4H_8O_2$ | $C_{66}H_{76}B_2N_4O_2\cdot C_4H_8O_2$ | $C_{33}H_{45}BN_2Si\cdot C_6H_{14}$ |
| w (g·mol$^{-1}$) | 1123.17 | 1035.03 | 594.78 |
| Temperature (K) | 100(2) | 100(2) | 120(2) |
| Radiation, λ (Å) | Mo-K$_\alpha$ 0.71073 | Mo-K$_\alpha$ 0.71073 | Cu-K$_\alpha$ 1.54184 |
| Crystal size (mm³) | 0.12×0.27×0.32 | 0.09×0.14×0.48 | 0.13×0.19×0.33 |
| Crystal color, habit | Yellow plate | Yellow plate | Yellow block |
| Crystal system | Monoclinic | Monoclinic | Monoclinic |
| Space group | $P2_1/n$ | $P2_1/n$ | $P2_1/c$ |
| *a* (Å) | 8.237(5) | 8.237(7) | 18.0602(3) |
| *b* (Å) | 42.45(3) | 38.52(3) | 31.5746(5) |
| *c* (Å) | 19.006(11) | 19.425(15) | 13.3605(2) |
| $\alpha$ (°) | 90 | 90 | 90 |
| $\beta$ (°) | 93.972(14) | 93.31(2) | 94.4160(10) |
| $\gamma$ (°) | 90 | 90 | 90 |
| Volume (Å$^3$) | 6630(7) | 6153(9) | 7596.1(2) |
| *Z* | 4 | 4 | 8 |
| $\rho_{calc}$ (g·cm$^{-3}$) | 1.125 | 1.117 | 1.040 |
| $\mu$ (mm$^{-1}$) | 0.127 | 0.066 | 0.727 |
| *F*(000) | 2408 | 2232 | 2608 |
| $\theta$ range (°) | 1.440 – 27.134 | 1.490 – 25.532 | 2.454 – 74.503 |
| Reflections collected | 61704 | 40724 | 79492 |
| Unique reflections | 14135 | 11380 | 15459 |
| Parameters / restraints | 876 / 114 | 849 / 418 | 906 / 102 |
| GooF on $F^2$ | 1.023 | 1.013 | 1.081 |
| $R_1$ [I>2σ(I)] | 0.0765 | 0.0732 | 0.0564 |
| $wR^2$ (all data) | 0.2232 | 0.2068 | 0.1601 |
| Max./min. residual electron density (e·Å$^{-3}$) | 0.700 / –0.375 | 0.624 / –0.401 | 0.468 / –0.269 |

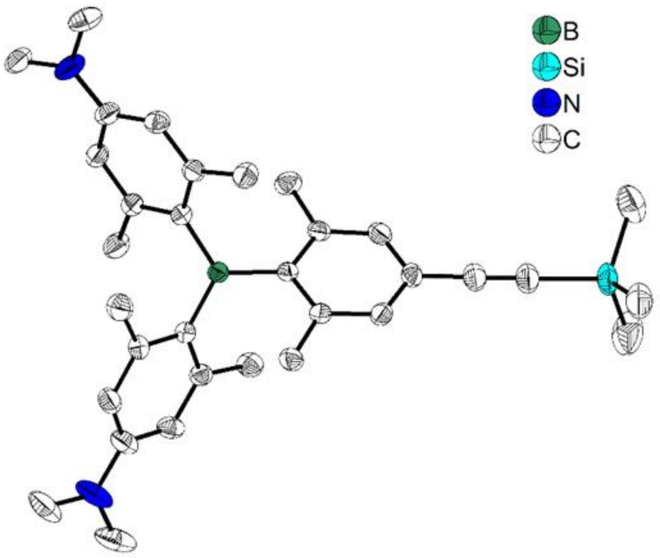


**Figure S13.** Molecular structure of compound **C** in the solid state at 120 K. Atomic displacement ellipsoids are drawn at the 50% probability level, and H atoms and co-crystallized solvent molecules (hexane) are omitted for clarity. Compound **C** contains two symmetry-independent molecules in the asymmetric unit. Only one of the two molecules is shown here. Selected angles for molecule 1: Sum (C–B1–C) 359.99(12)°, $BC_3$–Aryl (C1) 44.95(6)°, $BC_3$–Aryl (C2) 46.26(6)°, $BC_3$–Aryl (C3) 47.76(6)°. Selected angles for molecule 2: Sum (C–B1–C) 359.99(13)°, $BC_3$–Aryl (C1) 44.80(6)°, $BC_3$–Aryl (C2) 48.47(6)°, $BC_3$–Aryl (C3) 46.28(6)°.

# Linear Optical Properties

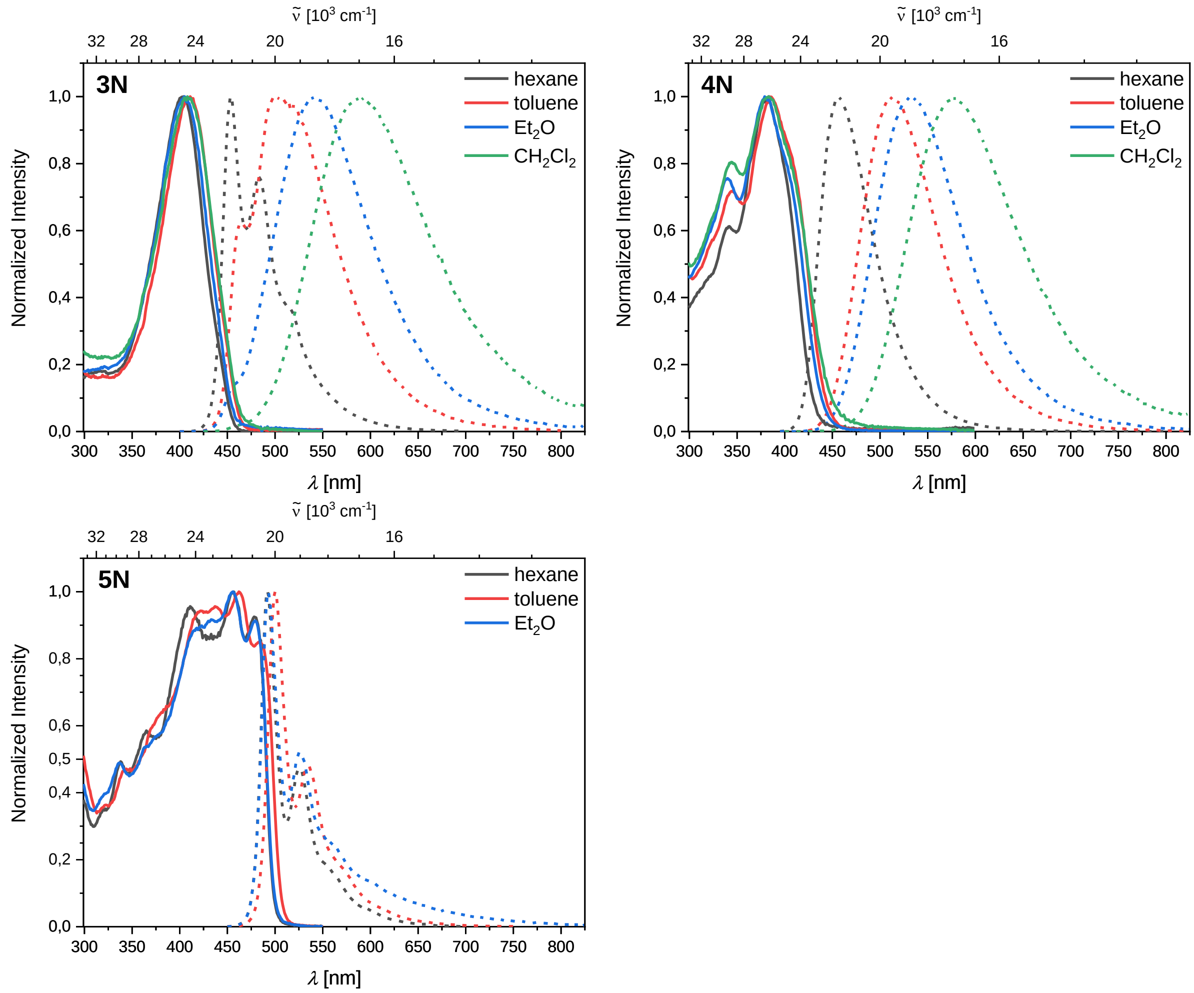


**Figure S14.** Absorption and emission spectra of **3N** (top, left), **4N** (top, right) and **5N** (bottom, left) in various solvents.

**Table S2.** Photophysical data for compounds **3N-5N** in various solvents.

| | solvent | $\lambda_{abs}$ / nm | $\varepsilon$ / M$^{-1}$ cm$^{-1}$ | $\lambda_{em}$ / nm | Stoke's shift / cm$^{-1}$ | $\Phi_f$ | $\tau$ / ns | $k_r$ / $10^8$ s$^{-1}$ | $k_{nr}$ / $10^8$ s$^{-1}$ |
|---|---|---|---|---|---|---|---|---|---|
| | hexane | 404 | 111 000 | 454 | 2 700 | 0.28 | <1 | - | - |
| **3N** | toluene | 411 | | 503 | 4 500 | 0.29 | 3.59 | 0.8 | 2.0 |
| | $Et_2O$ | 405 | | 542 | 6 200 | 0.26 | 6.00 | 0.4 | 1.2 |
| | $CH_2Cl_2$ | 408 | | 589 | 7 500 | 0.11 | 3.69 | 0.3 | 2.4 |
| | hexane | 383 | 82 000 | 458 | 4 300 | 0.12 | 1.79 | 0.7 | 4.9 |
| **4N** | toluene | 386 | | 513 | 6 400 | 0.20 | 4.07 | 0.5 | 2.0 |
| | $Et_2O$ | 379 | | 533 | 7 600 | 0.18 | 5.82 | 0.3 | 1.4 |
| | $CH_2Cl_2$ | 384 | | 579 | 8 800 | 0.13 | 5.47 | 0.2 | 1.6 |
| | hexane | 479 | 58 000 | 493 | 600 | 0.78 | 1.95 | 4.0 | 1.1 |
| **5N** | toluene | 483 | | 501 | 700 | 0.90 | 2.05 | 4.4 | 0.5 |
| | $Et_2O$ | 479 | | 493 | 600 | 0.28 | 3.68 | 0.8 | 2.0 |

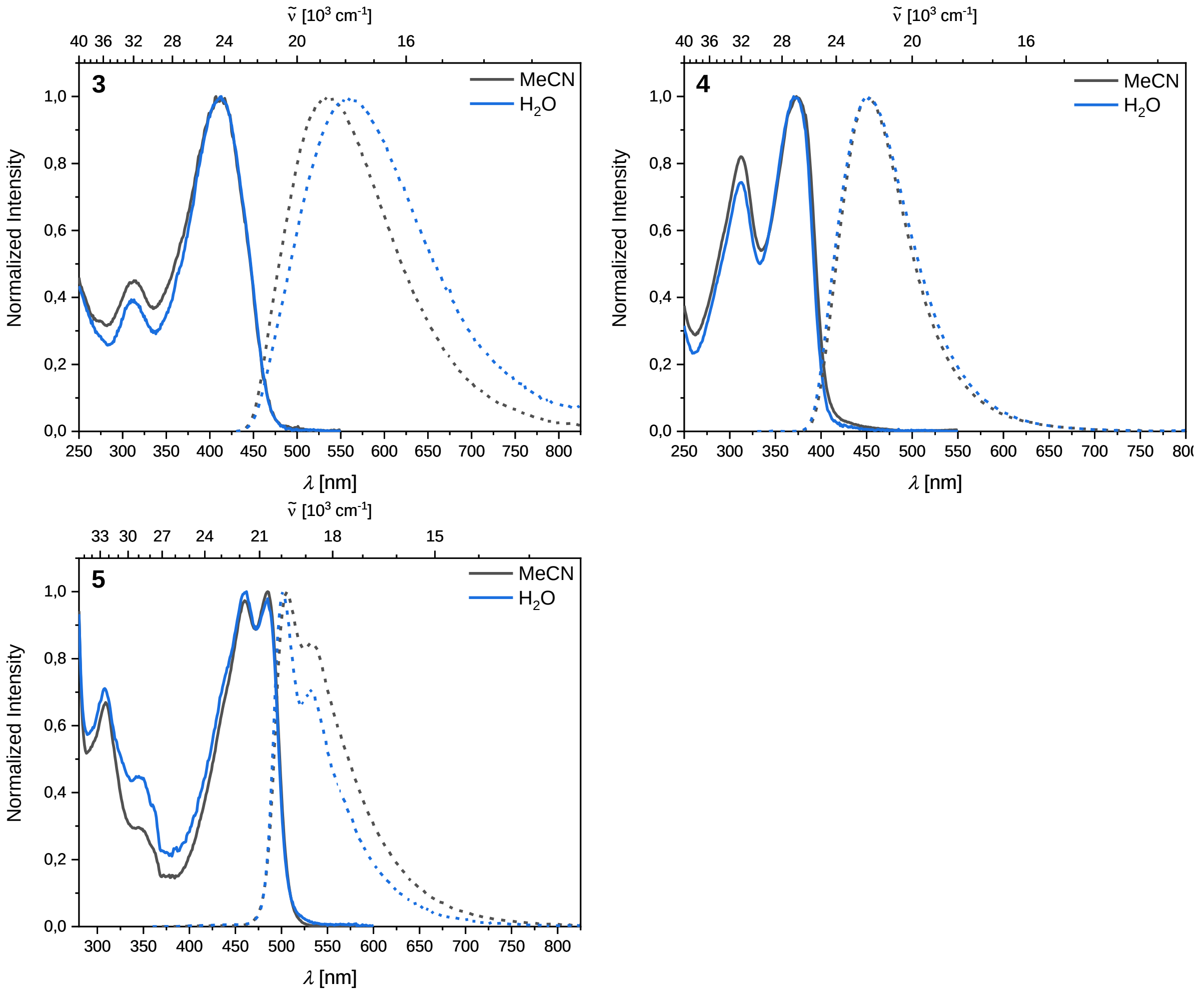


**Figure S15.** Absorption and emission spectra of **3** (top, left), **4** (top, right) and **5** (bottom, left) in various solvents.

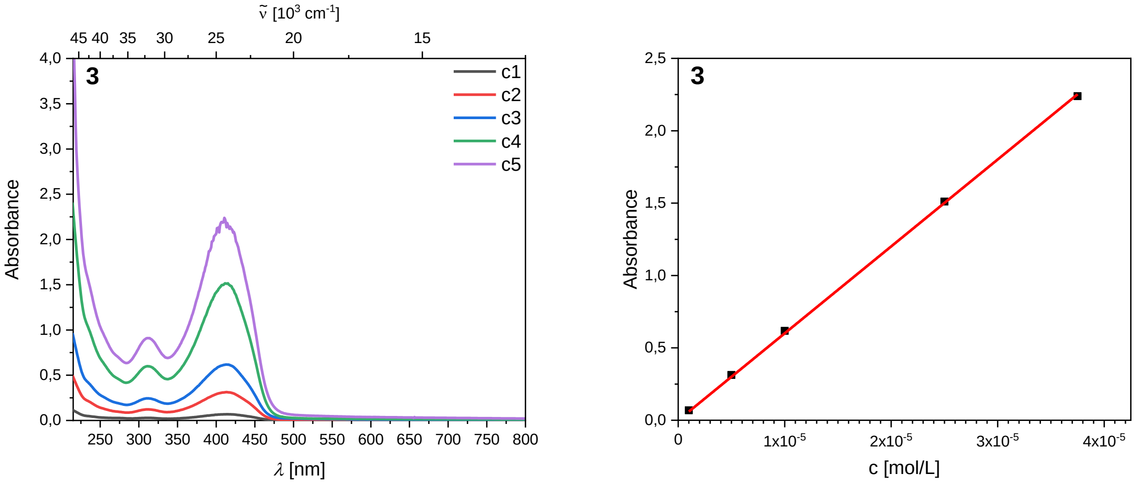


**Figure S16.** Linear dependence (—) of the absorbance at 413 nm in $H_2O$ on the concentration of compound **3** in the range from 1 $\times10^{-6}$ – 3.75 $\times10^{-5}$ M (■).

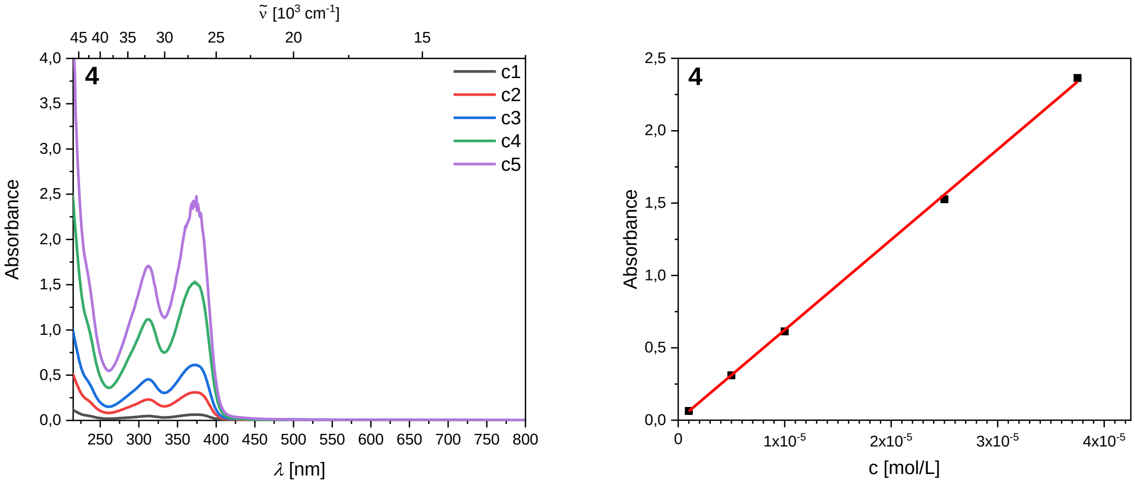


**Figure S17.** Linear dependence (—) of the absorbance at 371 nm in $H_2O$ on the concentration of compound **4** in the range from 1 $\times10^{-6}$ – 3.75 $\times10^{-5}$ M (■).

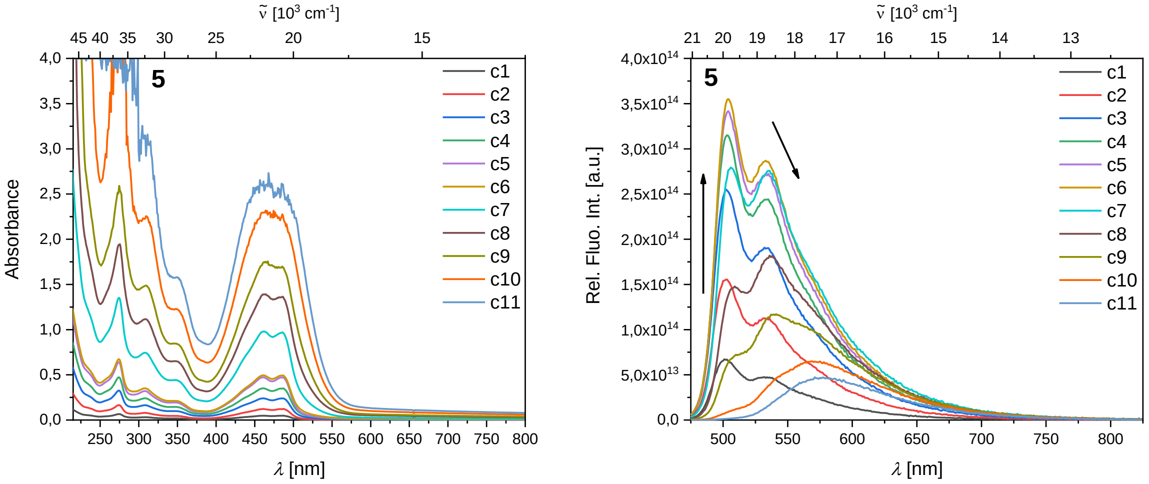


**Figure S18.** Left: Dependence of the absorbance of compound **5** on the concentration in the range from 1 $\times 10^{-6}$ – 1 $\times 10^{-4}$ M. Right: Dependence of the emission of compound **5** on the concentration in the range from 1 $\times 10^{-6}$ – 1 $\times 10^{-4}$ M.

# Optical Properties in Sodium Cacodylate

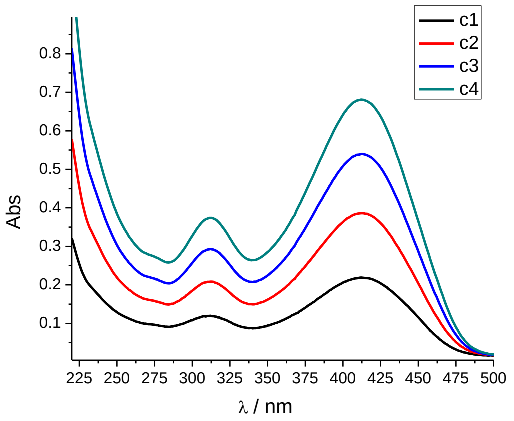


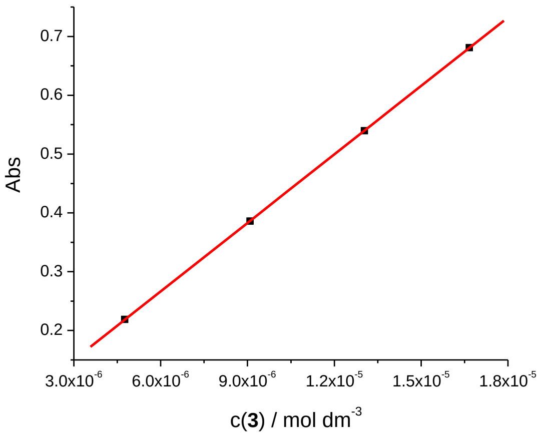


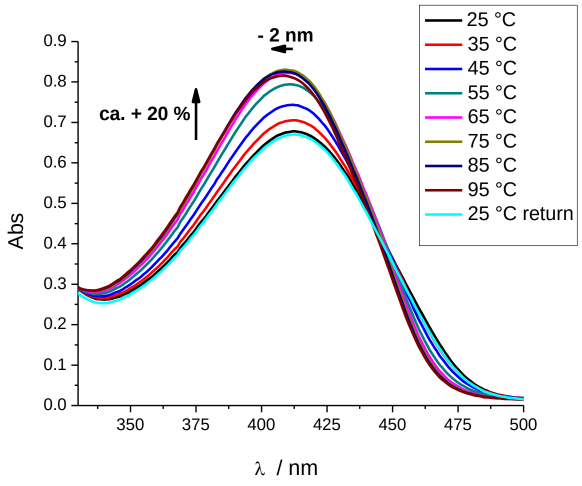


**Figure S19.** Top: UV/Vis spectra of **3**, c = 5 ×$10^{-6}$ – 1.7 ×$10^{-5}$ M (left); linear dependence (—) of the absorbance at 412 nm (■) on the **3** concentration (right); bottom: influence of temperature increase (T = 25 – 95 °C) on UV/Vis spectra of **3**, *c* = 1.7 ×$10^{-5}$ M. Measured in sodium cacodylate buffer, pH = 7.0, *I* = 0.05 M.

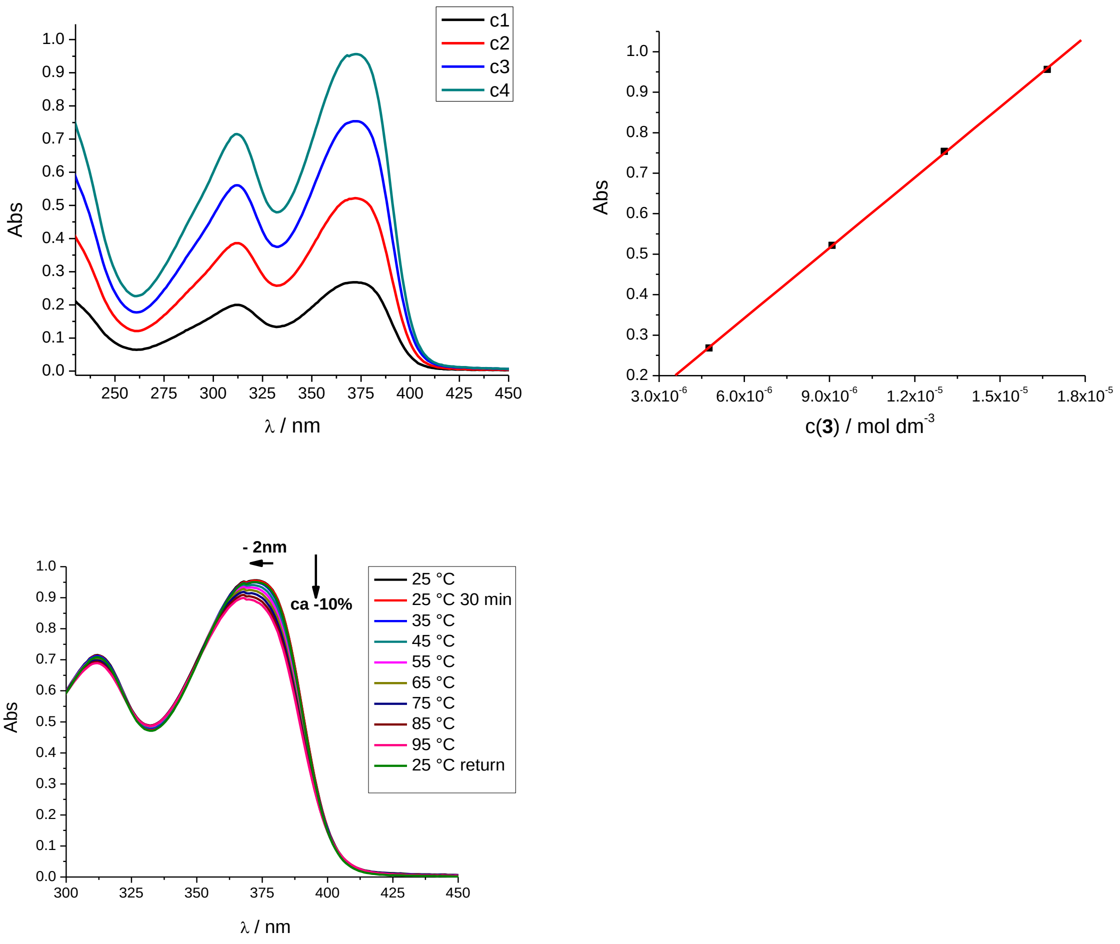


**Figure S20.** Top: UV/Vis spectra of **4**, c = 5 ×10$^{-6}$ – 1.7 ×10$^{-5}$ M (left); linear dependence (—) of the absorbance at 372 nm (■) on the **4** concentration (right); bottom: influence of temperature increase (T = 25 – 95 °C) on UV/Vis spectra of **4**, *c* = 1.7 ×10$^{-5}$ M. Measured in sodium cacodylate buffer, pH = 7.0, *I* = 0.05 M.

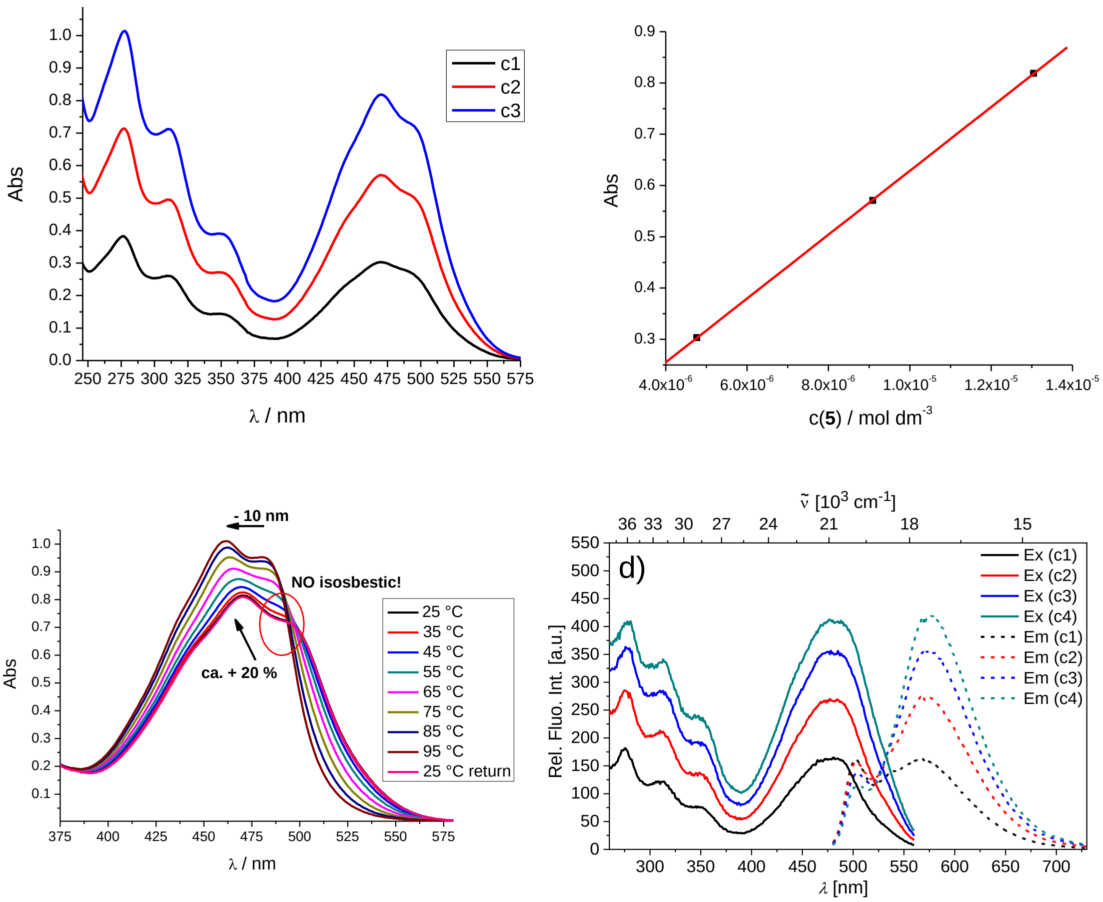


**Figure S21.** Top right: UV/Vis spectra of **5**, c = 5 $\times 10^{-6}$ – 1.3 $\times 10^{-5}$ M (left); top left: linear dependence (—) of the absorbance at 470 nm (■) on the **5** concentration (right); bottom left: influence of temperature increase (T = 25 – 95 °C) on UV/Vis spectra of **5**, *c* = 1.3 $\times 10^{-5}$ M (left).; bottom right: excitation / emission spectra overlap at *c*(**5**) = 5; 20; 35; 50 $\times 10^{-8}$ M. All measured in sodium cacodylate buffer, pH = 7.0, *I* = 0.05 M.

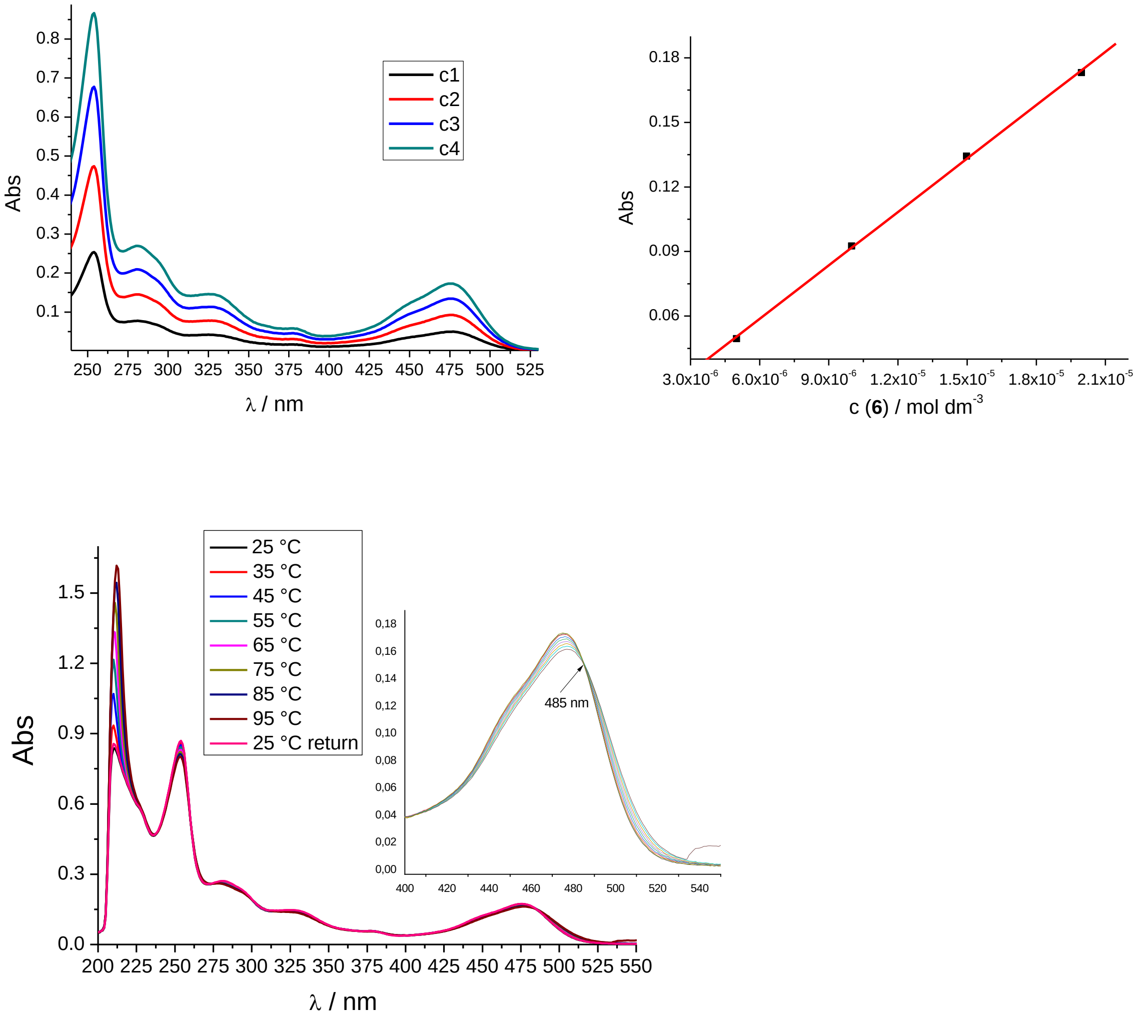


**Figure S22.** Top: UV/Vis spectra of **6**, c = 5 ×10$^{-6}$ – 2 ×10$^{-5}$ M (left); linear dependence (—) of the absorbance at 476 nm (■) on the **6** concentration (right); bottom: influence of temperature increase (T = 25 – 95 °C) on UV/Vis spectra of **6**, *c* = 2 ×10$^{-5}$ M. Measured in sodium cacodylate buffer, pH = 7.0, *I* = 0.05 M.

**Table S3.** Electronic absorption data of compounds **3**-**6.**

| **Compound** | **$\lambda_{max}$/nm** | **$\varepsilon \times 10^3$/mmol$^{-1}$ cm$^2$** |
|---|---|---|
| **3** | 412 | 38.8±0.1 |
| **4** | 372 | 57.9±0.4 |
| **5** | 470 | 62.2±0.2 |
| **6** | 476 | 8.3±0.1 |

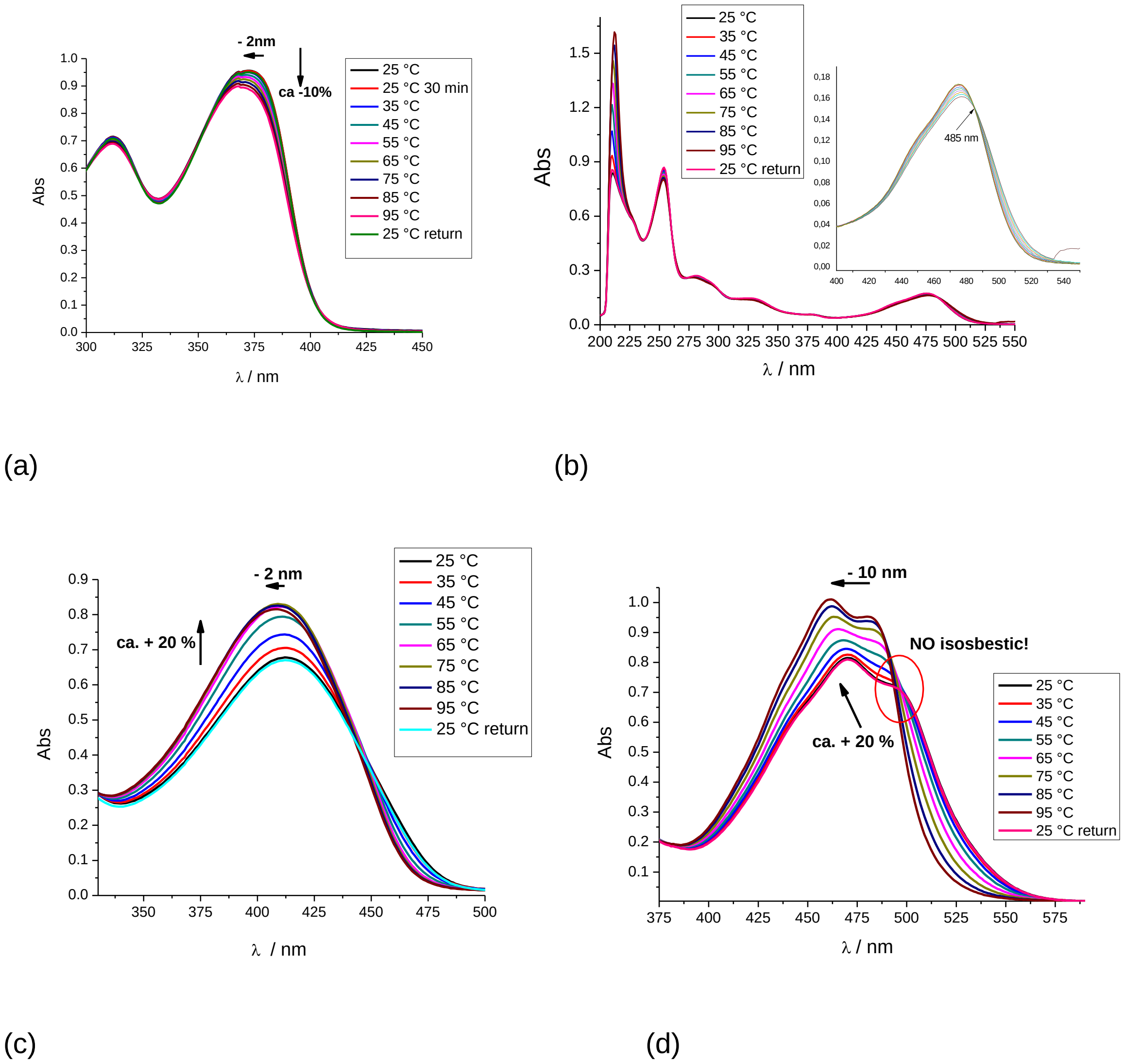


**Figure S23.** Temperature dependence of UV/Vis absorption spectra of compounds **3**-**6**: ($c$ = 1.7 ×$10^{-5}$ M) Note: ref. compound **4** (a) and **6** (b) hypochromic effect (> 10%) at variance to **3** (c) and **5** (d) hyperchromic effect (>+ 20%), suggesting that **3** and **5** display π-stacking interactions.

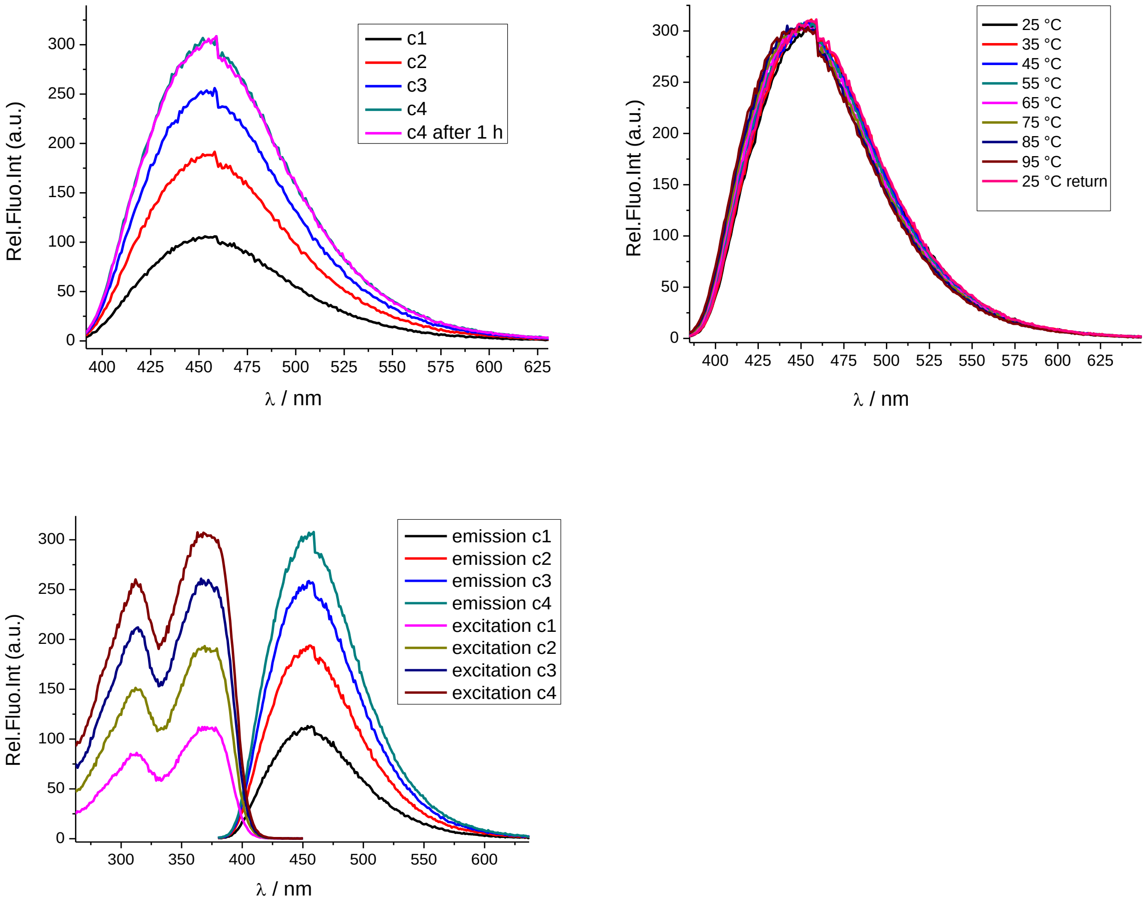


**Figure S24.** Top: fluorescence spectra of **4** ($\lambda_{exc}$ = 372 nm) in the concentration range c = 5 ×$10^{-8}$– 5 ×$10^{-7}$ M (left); influence of temperature increase (T = 25 – 95 °C) on fluorescence spectra of **4**, c = 5 ×$10^{-7}$ M (right); bottom: comparison of emission and excitation spectra of **4**. Measured in sodium cacodylate buffer (pH = 7.0, *I* = 0.05 M).

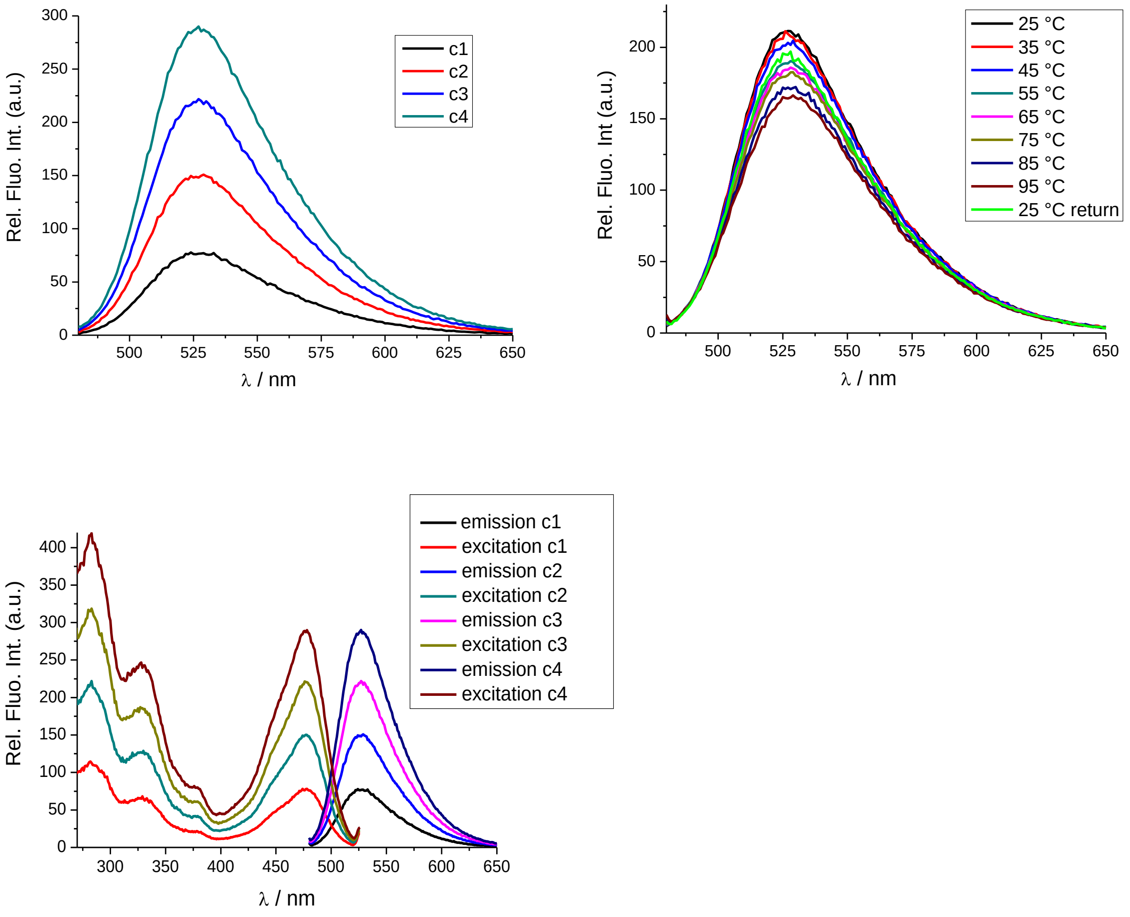


**Figure S25.** Top: fluorescence spectra of **6** ($\lambda_{exc}$ = 476 nm) in the concentration range c = 2 $\times 10^{-6}$– 8 $\times 10^{-6}$ M (left); influence of temperature increase (T = 25 – 95 °C) on fluorescence spectra of **6**, c = 8 $\times 10^{-6}$ M (right); bottom: comparison of emission and excitation spectra of **6**. Measured in sodium cacodylate buffer (pH = 7.0, *I* = 0.05 M).

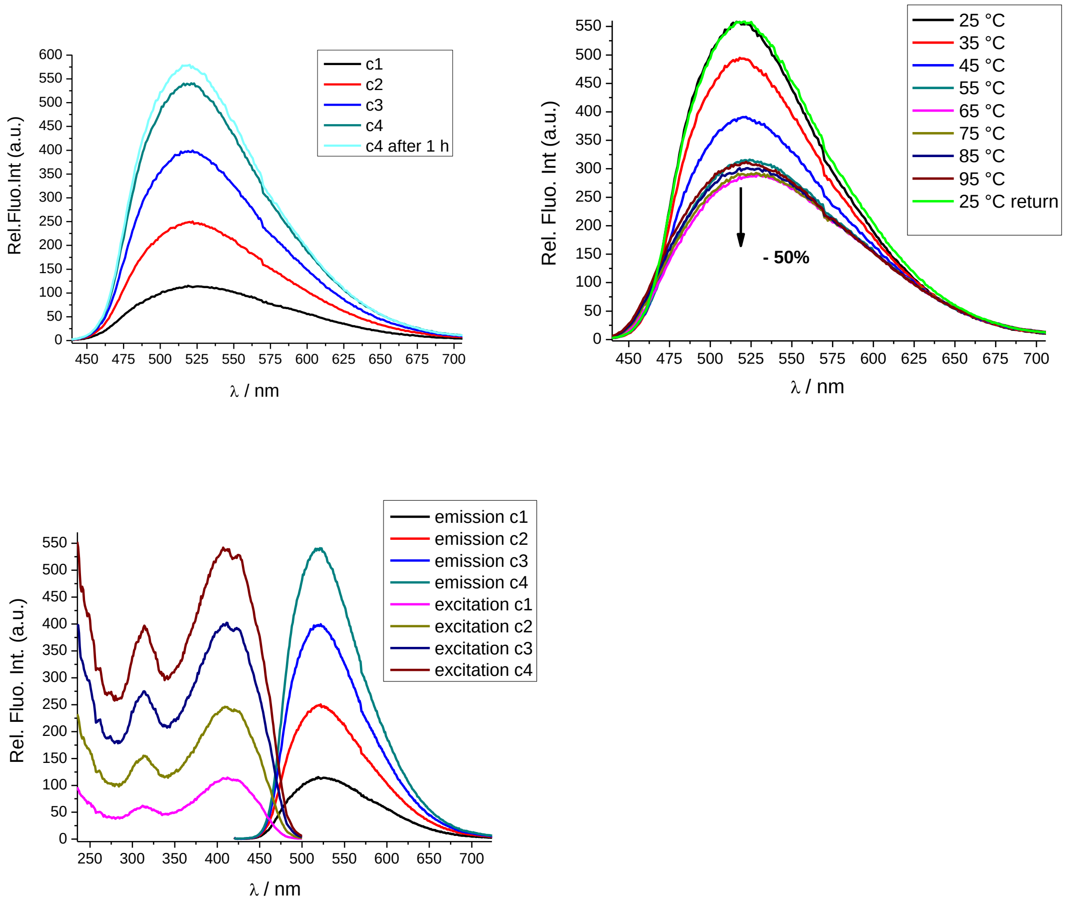


**Figure S26.** Top: fluorescence spectra of **3** ($\lambda_{exc}$ = 412 nm) in the concentration range c = 5 ×10$^{-8}$– 5 ×10$^{-7}$ M (left); influence of temperature increase (T = 25 – 95 °C) on fluorescence spectra of **3**, c = 5 ×10$^{-7}$ M (right); bottom: comparison of emission and excitation spectra of **3**. Measured in sodium cacodylate buffer (pH = 7.0, *I* = 0.05 M).

# Studies of Interactions with DNA and RNA

**Table S4.** Groove widths and depths for selected nucleic acid conformations.[30, 31]

| Structure type | Groove width [Å] | | Groove depth [Å] | |
|---|---|---|---|---|
| | major | minor | major | minor |
| poly rA – poly rU[a] | 3.8 | 10.9 | 13.5 | 2.8 |
| poly dA – poly dT[b] | 11.4 | 3.3 | 7.5 | 7.9 |
| poly dGdC – poly dGdC[c] | 13.5 | 9.5 | 10.0 | 7.2 |
| poly dAdT – poly dAdT[c] | 11.2 | 6.3 | 8.5 | 7.5 |

[a] A-helical structure (e.g. A-DNA); [b] C-helical structure (e.g. C-DNA); [c] B- helical structure (i.e. B-DNA)

## Thermal Melting Experiments

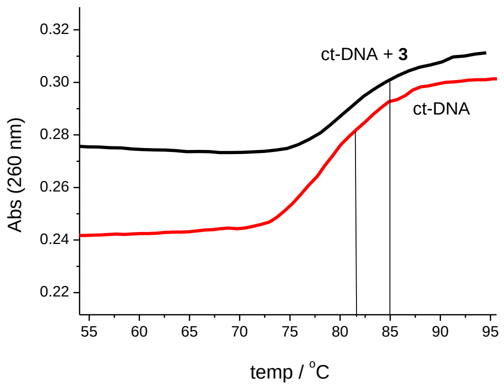


**Figure S27.** Thermal denaturation curves of ct-DNA (*c*(ct-DNA) = 2.5 × 10$^{-5}$ M, $r_{[3]/[ct\text{-}DNA]}$ = 0.1) at pH 7.0 (sodium cacodylate buffer, *I* = 0.05 M) upon addition of **3**. Error in ΔTm values: ±0.5 °C.

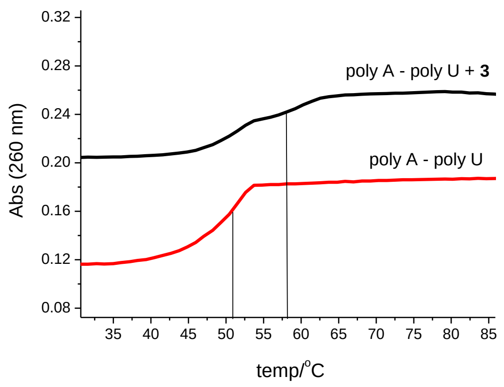


**Figure S28.** Thermal denaturation curves of poly A – poly U (*c*(poly A – poly U) = 2.5 × 10$^{-5}$ M, $r_{[3]/[poly\ A-poly\ U]}$ = 0.1) at pH 7.0 (sodium cacodylate buffer, *I* = 0.05 M) upon addition of **3**. Error in ΔTm values: ±0.5 °C.

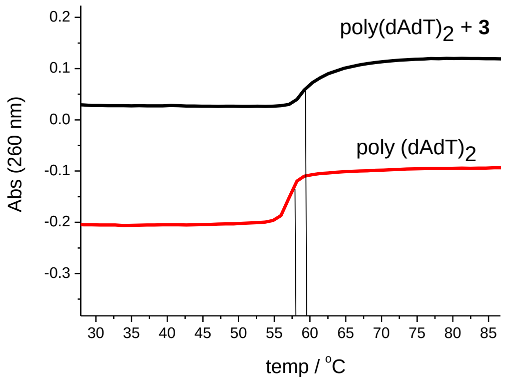


**Figure S29.** Thermal denaturation curves of poly (dAdT)$_2$ ($c$(poly (dAdT)$_2$) = 2.5 × 10$^{-5}$ M, $r_{[3]/[poly(dAdT)2]}$ = 0.1) at pH 7.0 (sodium cacodylate buffer, $I$ = 0.05 M) upon addition of **3**. Error in ΔTm values: ±0.5 °C.

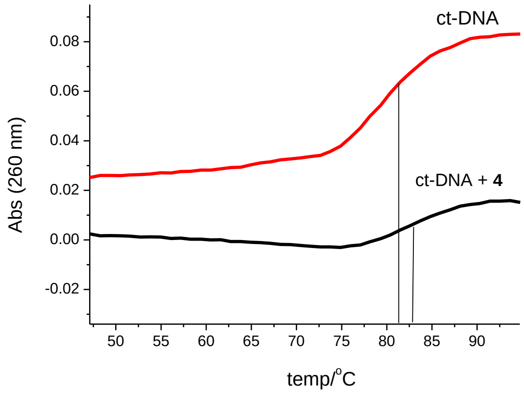


**Figure S30.** Thermal denaturation curves of ct-DNA ($c$(ct-DNA) = 2.5 × 10$^{-5}$ M, $r_{[4]/[ct\text{-}DNA]}$ = 0.1) at pH 7.0 (sodium cacodylate buffer, $I$ = 0.05 M) upon addition of **4**. Error in ΔTm values: ±0.5 °C.

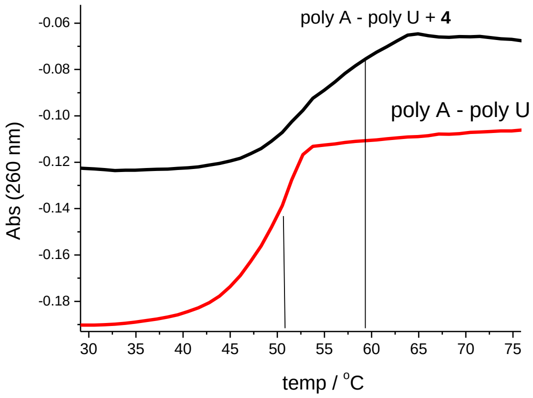


**Figure S31.** Thermal denaturation curves of poly A – poly U ($c$(poly A – poly U) = 2.5 × 10$^{-5}$ M, $r_{[4]/[poly\ A - poly\ U]}$ = 0.1) at pH 7.0 (sodium cacodylate buffer, $I$ = 0.05 M) upon addition of **4**. Error in ΔTm values: ±0.5 °C.

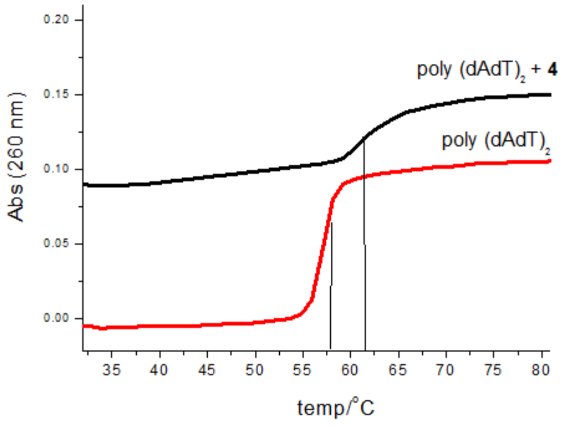


**Figure S32.** Thermal denaturation curves of poly (dAdT)$_2$ ($c$(poly (dAdT)$_2$) = 2.5 × $10^{-5}$ M, $r_{[4]/[\text{ poly(dAdT)2}]}$ = 0.1) at pH 7.0 (sodium cacodylate buffer, $I$ = 0.05 M) upon addition of **4**. Error in ΔTm values: ±0.5 °C.

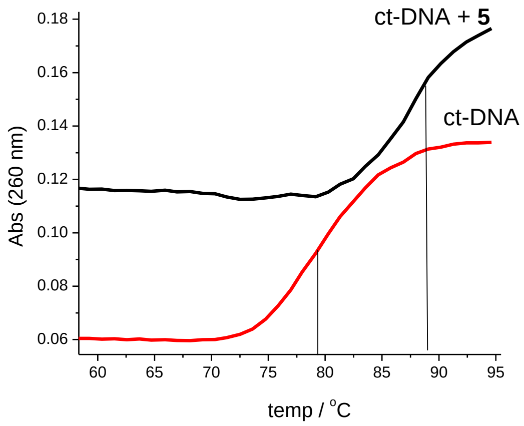


**Figure S33.** Thermal denaturation curves of ct-DNA ($c$(ct-DNA) = 2.5 × $10^{-5}$ M, $r_{[5]/[\text{ct-DNA}]}$ = 0.1) at pH 7.0 (sodium cacodylate buffer, $I$ = 0.05 M) upon addition of **5**. Error in ΔTm values: ±0.5 °C.

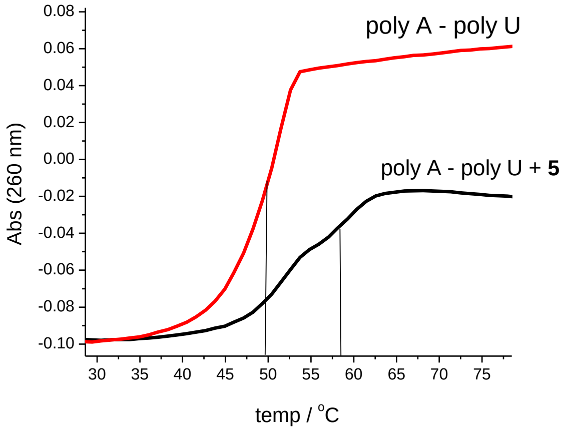


**Figure S34.** Thermal denaturation curves of poly A – poly U ($c$(poly A – poly U) = 2.5 × $10^{-5}$ M, $r_{[5]/[\text{poly A – poly U}]}$ = 0.1) at pH 7.0 (sodium cacodylate buffer, $I$ = 0.05 M) upon addition of **5**. Error in ΔTm values: ±0.5 °C.

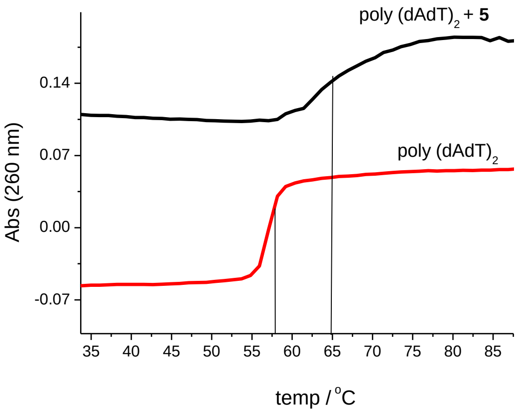


**Figure S35.** Thermal denaturation curves of poly (dAdT)2 ($c$(poly (dAdT)2) = 2.5 × $10^{-5}$ M, $r_{[5]/[poly(dAdT)2]}$ = 0.1) at pH 7.0 (sodium cacodylate buffer, $I$ = 0.05 M) upon addition of **5**. Error in ΔTm values: ±0.5 °C.

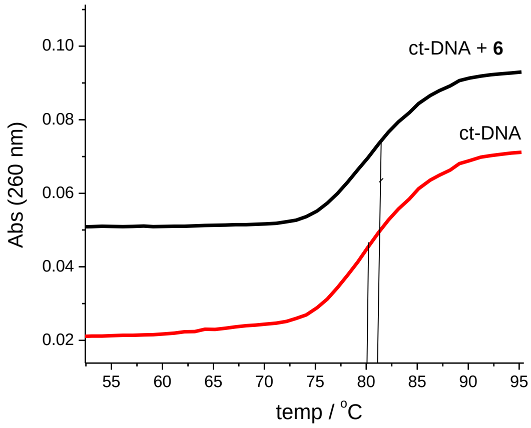


**Figure S36.** Thermal denaturation curves of ct-DNA ($c$(ct-DNA) = 2.5 × $10^{-5}$ M, $r_{[6]/[ct\text{-}DNA]}$ = 0.1) at pH 7.0 (sodium cacodylate buffer, $I$ = 0.05 M) upon addition of **6**. Error in ΔTm values: ±0.5 °C.

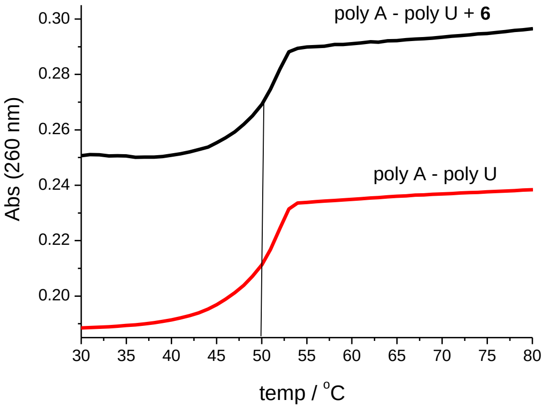


**Figure S37.** Thermal denaturation curves of poly A – poly U ($c$(poly A – poly U) = 2.5 × $10^{-5}$ M, $r_{[6]/[poly\ A - poly\ U]}$ = 0.3) at pH 7.0 (sodium cacodylate buffer, $I$ = 0.05 M) upon addition of **6**. Error in ΔTm values: ±0.5 °C.

## Fluorimetric Titrations

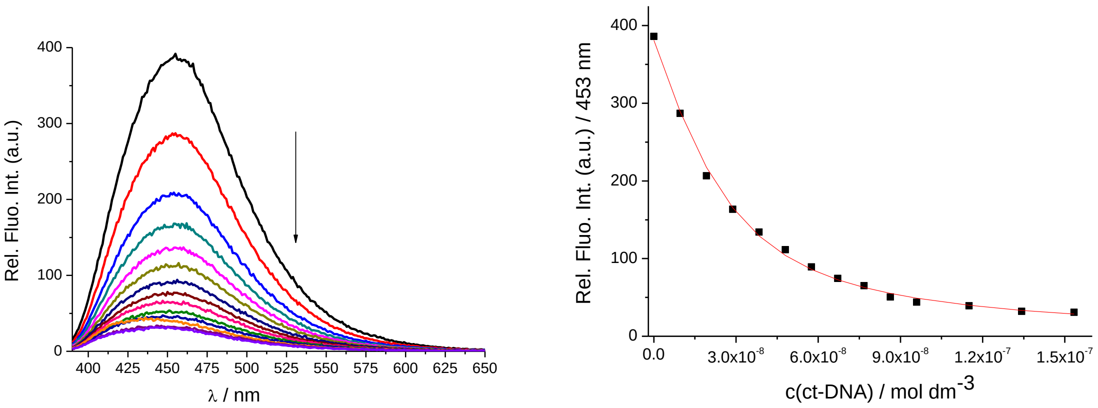


**Figure S38.** LEFT: Fluorimetric titration of **4** ($c$ = 5 $\times 10^{-9}$ M; $\lambda_{exc}$= 372 nm) with **ct-DNA**. RIGHT: Dependence of fluorescence at $\lambda_{max}$ = 453 nm on $c$(DNA), red line is non-linear least square fitting of Scatchard eq. (McGhee, von Hippel formalism) to the experimental data. Measured at pH 7, sodium cacodylate buffer, $I$ = 0.05 M

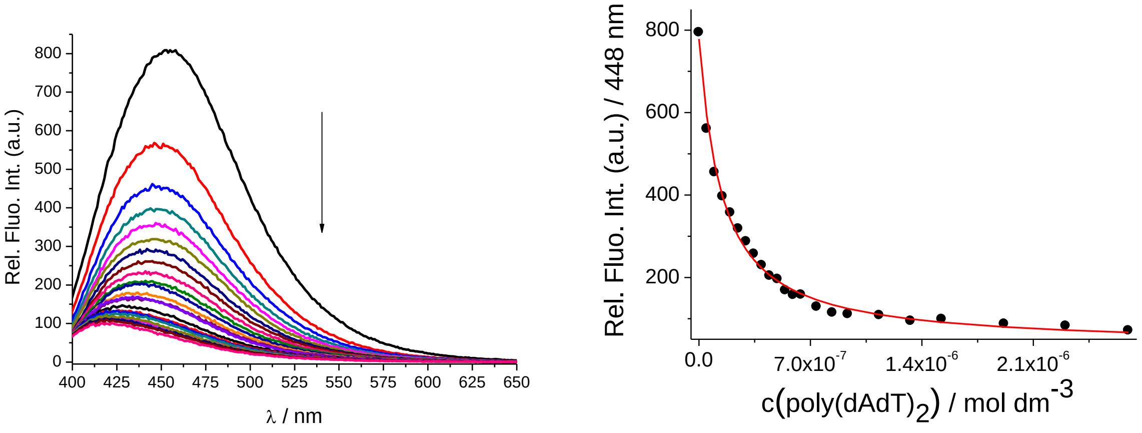


**Figure S39.** Left: Fluorimetric titration of **4** ($c$ = 5 $\times 10^{-9}$ M; $\lambda_{exc}$= 372 nm) with **poly (dAdT)$_2$**. Right: Dependence of fluorescence at $\lambda_{max}$ = 448 nm on $c$(DNA), red line is non-linear least square fitting of Scatchard eq. (McGhee, von Hippel formalism) to the experimental data. Measured at pH 7, sodium cacodylate buffer, $I$ = 0.05 M.

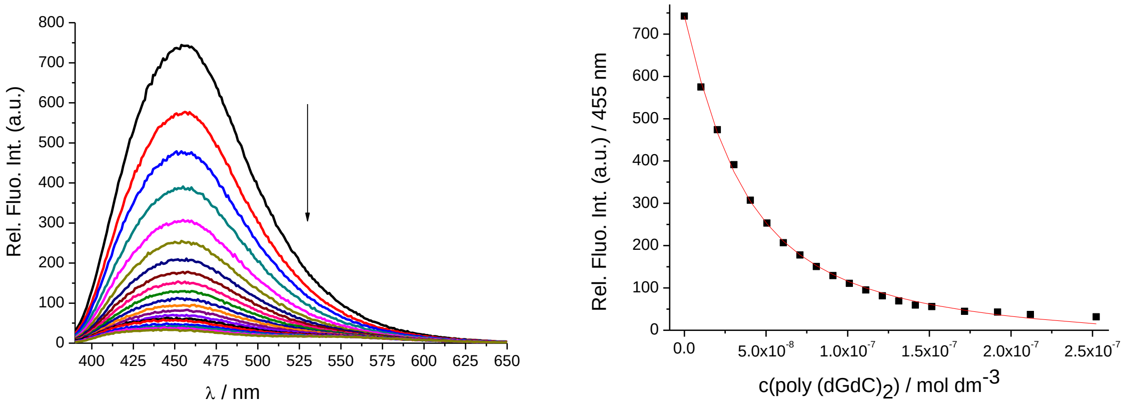


**Figure S40.** Left: Fluorimetric titration of **4** ($c$ = 5 ×$10^{-9}$ M; $\lambda_{exc}$= 372 nm) with **poly (dGdC)$_2$**. Right: Dependence of fluorescence at $\lambda_{max}$ = 455 nm on $c$(DNA), red line is non-linear least square fitting of Scatchard eq. (McGhee, von Hippel formalism) to the experimental data. Measured at pH 7, sodium cacodylate buffer, $I$ = 0.05 M.

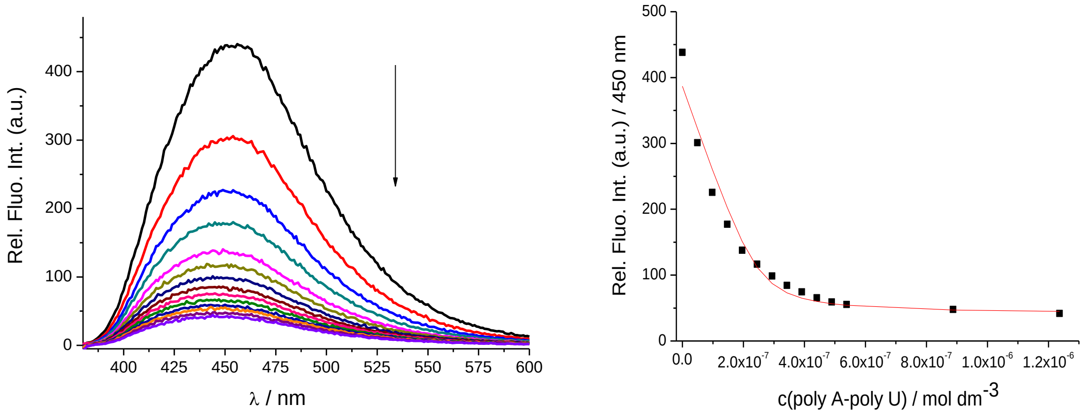


**Figure S41.** Left: Fluorimetric titration of **4** ($c$ = 5 ×$10^{-8}$ M; $\lambda_{exc}$= 372 nm) with **poly A - poly U**. Right: Dependence of fluorescence at $\lambda_{max}$ = 450 nm on $c$(RNA), red line is non-linear least square fitting of Scatchard eq. (McGhee, von Hippel formalism) to the experimental data. Measured at pH 7, sodium cacodylate buffer, $I$ = 0.05 M.

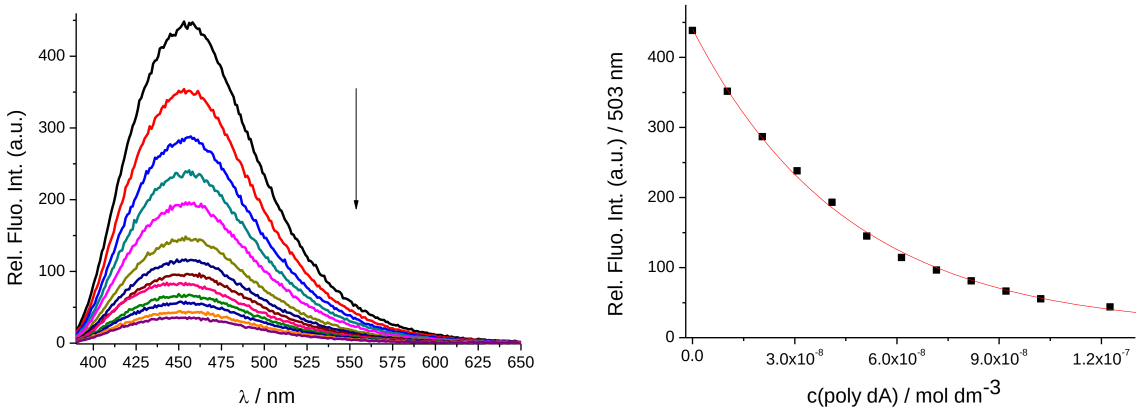


**Figure S42**. Left: Fluorimetric titration of **4** ($c$ = 5 ×$10^{-8}$ M; $\lambda_{exc}$= 372 nm) with **poly dA**. Right: Dependence of fluorescence at $\lambda_{max}$ = 503 nm on $c$(DNA), red line is non-linear least square fitting of Scatchard eq. (McGhee, von Hippel formalism) to the experimental data. Measured at pH 7, sodium cacodylate buffer, $I$ = 0.05 M.

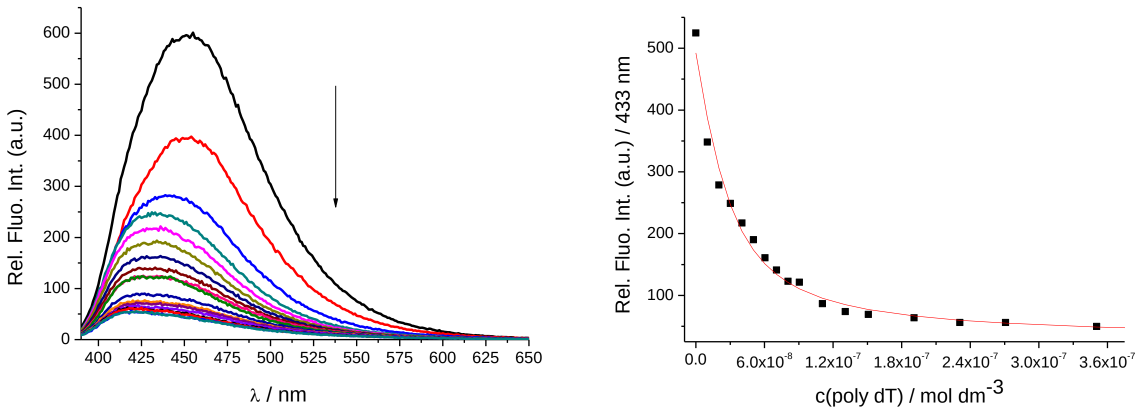


**Figure S43.** Left: Fluorimetric titration of **4** ($c$ = 5 $\times 10^{-9}$ M; $\lambda_{exc}$= 372 nm) with **poly dT**. Right: Dependence of fluorescence at $\lambda_{max}$ = 433 nm on $c$(DNA), red line is non-linear least square fitting of Scatchard eq. (McGhee, von Hippel formalism) to the experimental data. Measured at pH 7, sodium cacodylate buffer, $I$ = 0.05 M.

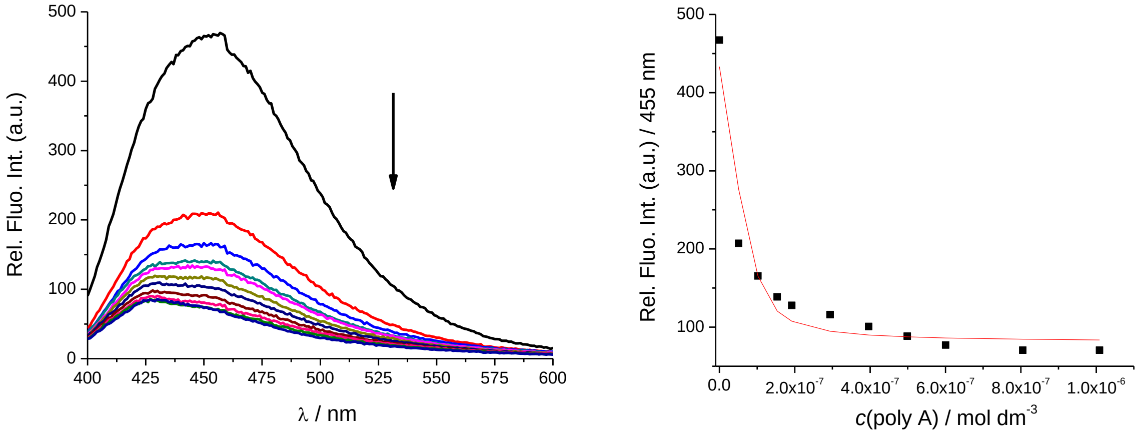


**Figure S44.** Left: Fluorimetric titration of **4** ($c$ = 5 $\times 10^{-8}$ M; $\lambda_{exc}$= 372 nm) with **poly A**. Right: Dependence of fluorescence at $\lambda_{max}$ = 455 nm on $c$(RNA), red line is non-linear least square fitting of Scatchard eq. (McGhee, von Hippel formalism) to the experimental data. Measured at pH 7, sodium cacodylate buffer, $I$ = 0.05 M.

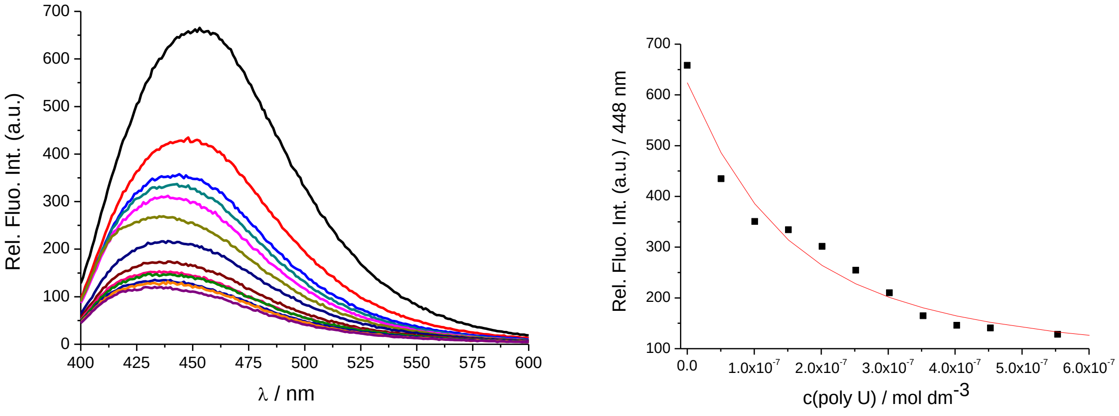


**Figure S45.** Left: Fluorimetric titration of **4** ($c$ = 5 $\times 10^{-8}$ M; $\lambda_{exc}$= 372 nm) with **poly U**. Right: Dependence of fluorescence at $\lambda_{max}$ = 448 nm on $c$(RNA), red line is non-linear least square fitting of Scatchard eq. (McGhee, von Hippel formalism) to the experimental data. Measured at pH 7, sodium cacodylate buffer, $I$ = 0.05 M.

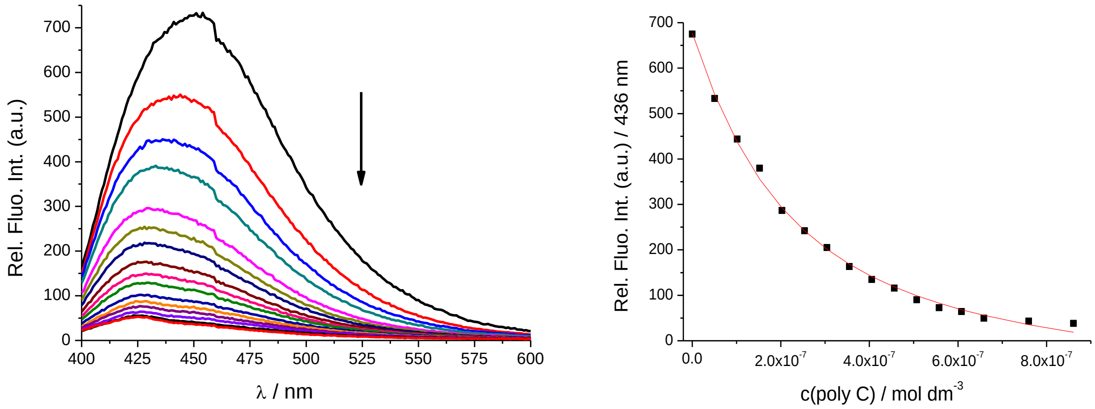


**Figure S46.** Left: Fluorimetric titration of **4** ($c$ = 5 $\times 10^{-8}$ M; $\lambda_{exc}$= 372 nm) with **poly C**. Right: Dependence of fluorescence at $\lambda_{max}$ = 436 nm on $c$(RNA), red line is non-linear least square fitting of Scatchard eq. (McGhee, von Hippel formalism) to the experimental data. Measured at pH 7, sodium cacodylate buffer, $I$ = 0.05 M.

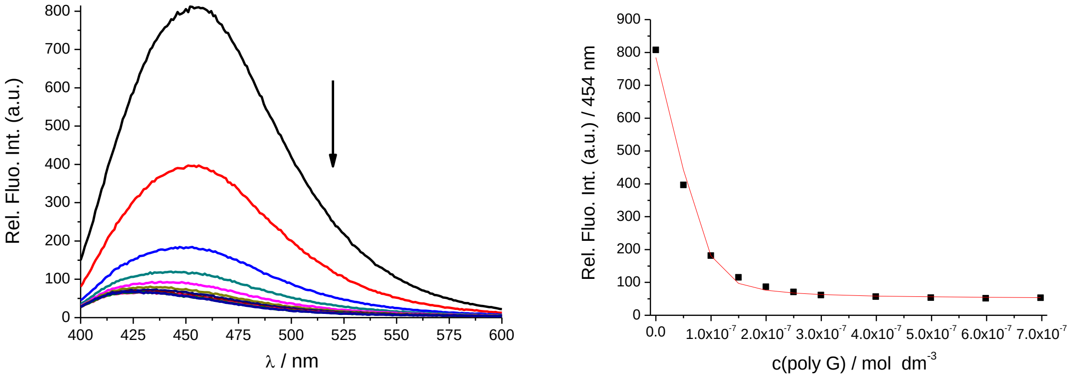


**Figure S47.** Left: Fluorimetric titration of **4** ($c$ = 5 $\times 10^{-8}$ M; $\lambda_{exc}$= 372 nm) with **poly G**. Right: Dependence of fluorescence at $\lambda_{max}$ = 454 nm on $c$(RNA), red line is non-linear least square fitting of Scatchard eq. (McGhee, von Hippel formalism) to the experimental data. Measured at pH 7, sodium cacodylate buffer, $I$ = 0.05 M.

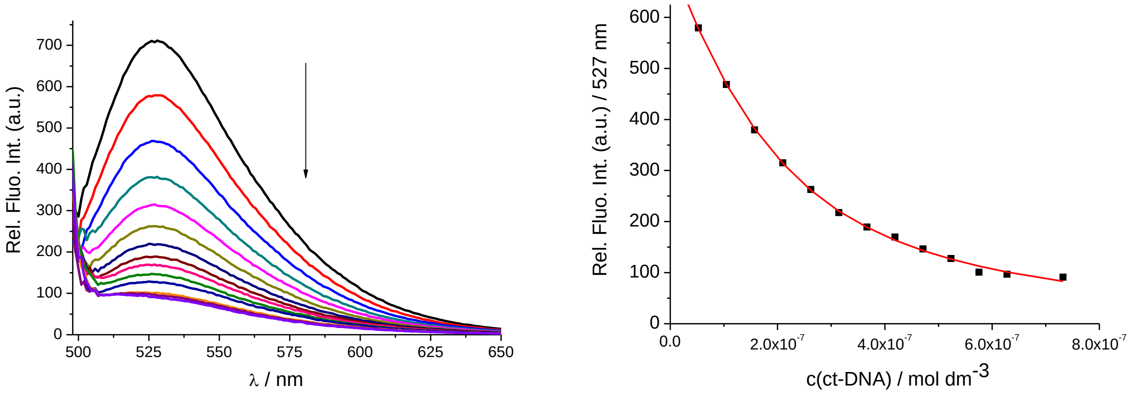


**Figure S48.** Left: Fluorimetric titration of **6** ($c$ = 5 $\times 10^{-8}$ M; $\lambda_{exc}$= 476 nm) with **ct-DNA**. Right: dependence of fluorescence at $\lambda_{max}$ = 527 nm on $c$(DNA), red line is non-linear least square fitting of Scatchard eq. (McGhee, von Hippel formalism) to the experimental data. Measured at pH 7, sodium cacodylate buffer, $I$ = 0.05 M.

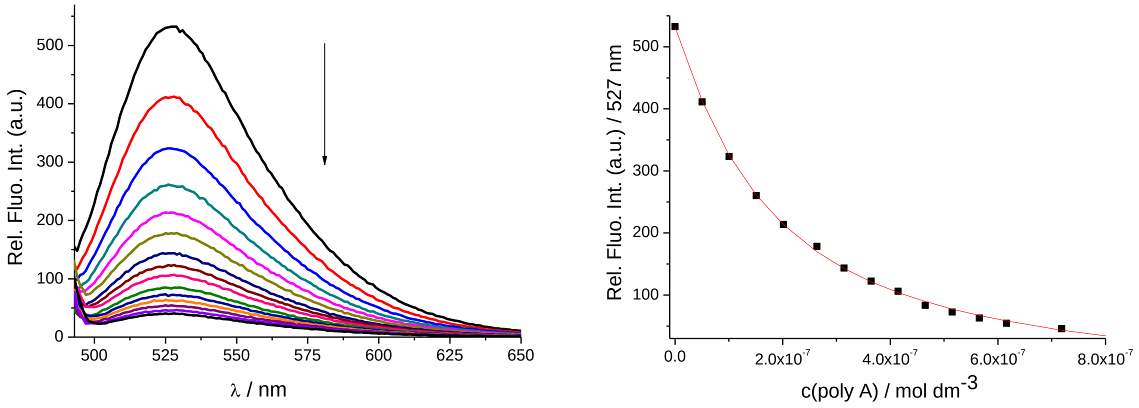


**Figure S49.** Left: Fluorimetric titration of **6** ($c$ = 5 ×$10^{-8}$ M; $\lambda_{exc}$= 476 nm) with **poly A**. Right: dependence of fluorescence at $\lambda_{max}$ = 527 nm on $c$(RNA), red line is non-linear least square fitting of Scatchard eq. (McGhee, von Hippel formalism) to the experimental data. Measured at pH 7, sodium cacodylate buffer, $I$ = 0.05 M.

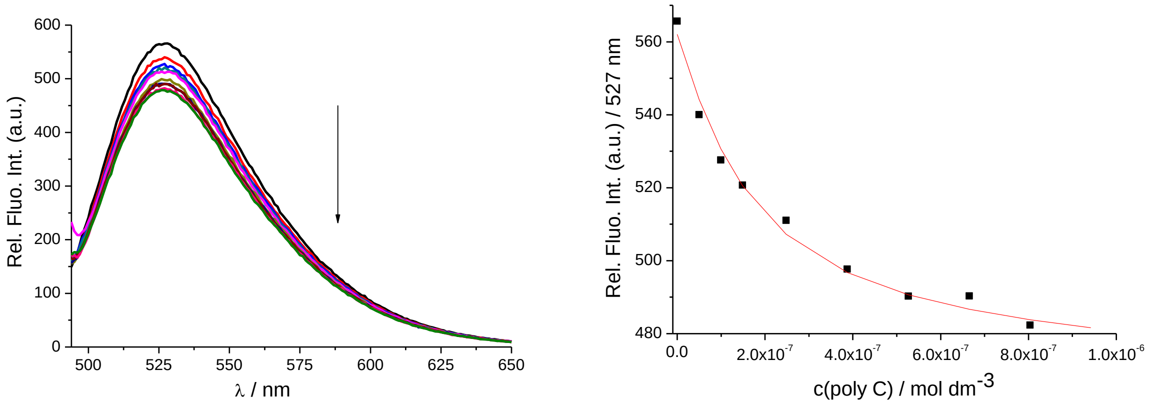


**Figure S50.** Left: Fluorimetric titration of **6** ($c$ = 5 ×$10^{-8}$ M; $\lambda_{exc}$= 476 nm) with **poly C** Right: dependence of fluorescence at $\lambda_{max}$ = 527 nm on $c$(RNA), red line is non-linear least square fitting of Scatchard eq. (McGhee, von Hippel formalism) to the experimental data. Measured at pH 7, sodium cacodylate buffer, $I$ = 0.05 M.

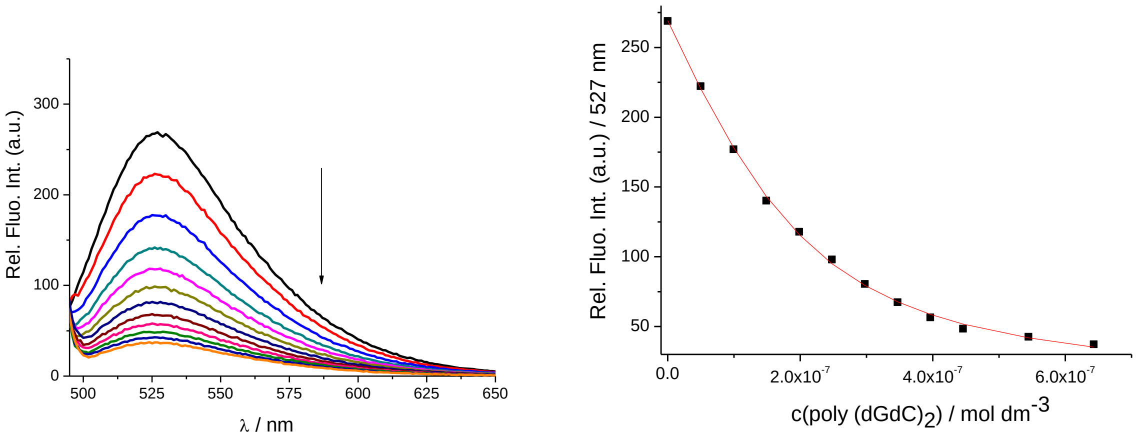


**Figure S51.** Left: Fluorimetric titration of **6** ($c$ = 5 ×$10^{-8}$ M; $\lambda_{exc}$= 476 nm) with **poly (dGdC)$_2$** Right: dependence of fluorescence at $\lambda_{max}$ = 527 nm on $c$(DNA), red line is non-linear least square fitting of Scatchard eq. (McGhee, von Hippel formalism) to the experimental data. Measured at pH 7, sodium cacodylate buffer, $I$ = 0.05 M.

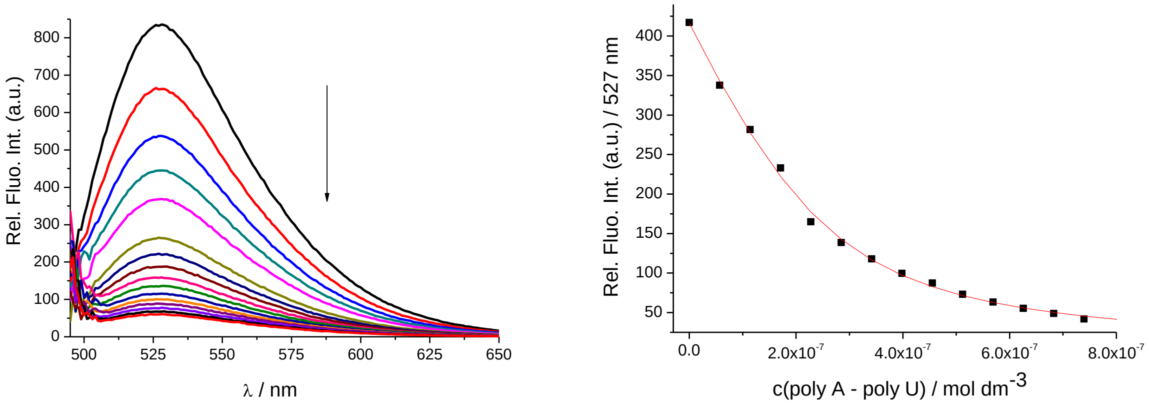


**Figure S52.** Left: Fluorimetric titration of **6** (c = 5 ×10-8 M; $\lambda_{exc}$= 476 nm) with **poly A – poly U**. Right: dependence of fluorescence at $\lambda_{max}$ = 527 nm on *c*(RNA), red line is non-linear least square fitting of Scatchard eq. (McGhee, von Hippel formalism) to the experimental data. Measured at pH 7, sodium cacodylate buffer, *I* = 0.05 M.

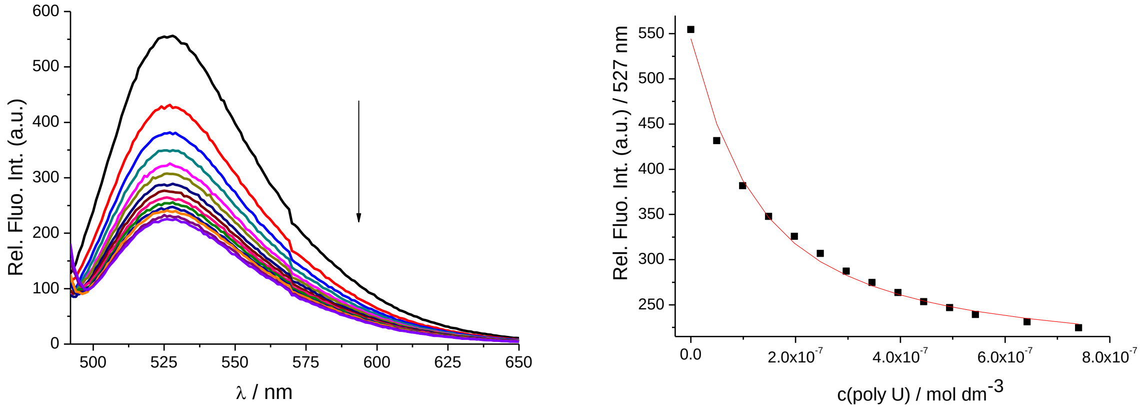


**Figure S53.** Left: Fluorimetric titration of **6** (*c* = 5 $\times 10^{-8}$ M; $\lambda_{exc}$= 476 nm) with **poly U**. Right: dependence of fluorescence at $\lambda_{max}$ = 527 nm on *c*(RNA), red line is non-linear least square fitting of Scatchard eq. (McGhee, von Hippel formalism) to the experimental data. Measured at pH 7, sodium cacodylate buffer, *I* = 0.05 M.

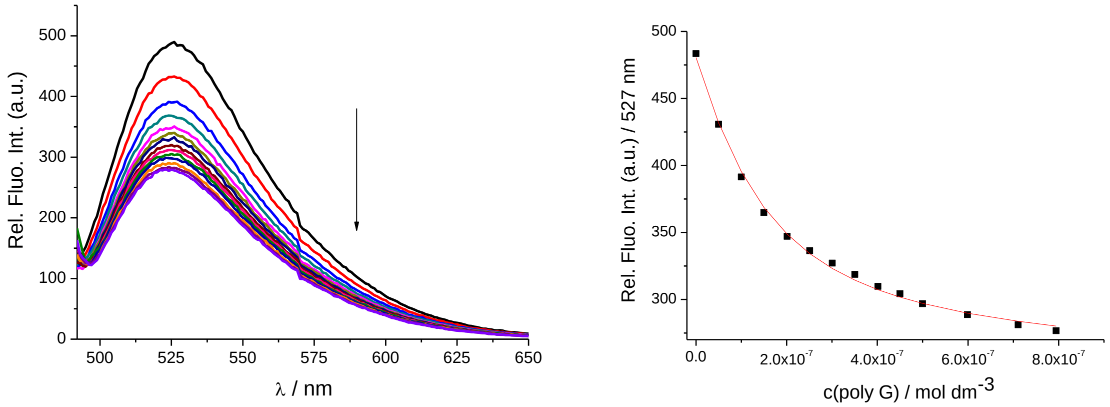


**Figure S54.** Left: Fluorimetric titration of **6** (*c* = 5 $\times 10^{-8}$ M; $\lambda_{exc}$= 476 nm) with **poly G**. Right: dependence of fluorescence at $\lambda_{max}$ = 527 nm on *c*(RNA), red line is non-linear least square fitting of Scatchard eq. (McGhee, von Hippel formalism) to the experimental data. Measured at pH 7, sodium cacodylate buffer, *I* = 0.05 M.

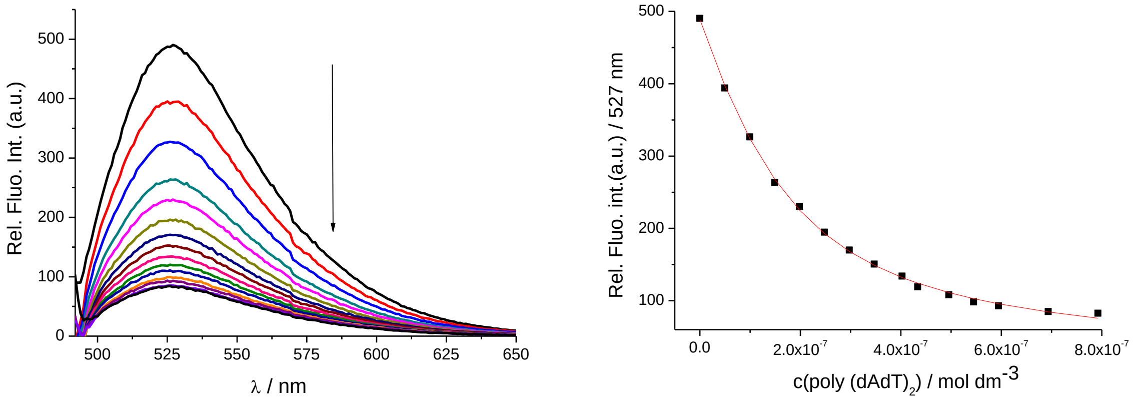


**Figure S55.** Left: Fluorimetric titration of **6** ($c$ = 5 $\times 10^{-8}$ M; $\lambda_{exc}$= 476 nm) with **poly (dAdT)$_2$**. Right: dependence of fluorescence at $\lambda_{max}$ = 527 nm on $c$(DNA), red line is non-linear least square fitting of Scatchard eq. (McGhee, von Hippel formalism) to the experimental data. Measured at pH 7, sodium cacodylate buffer, $I$ = 0.05 M.

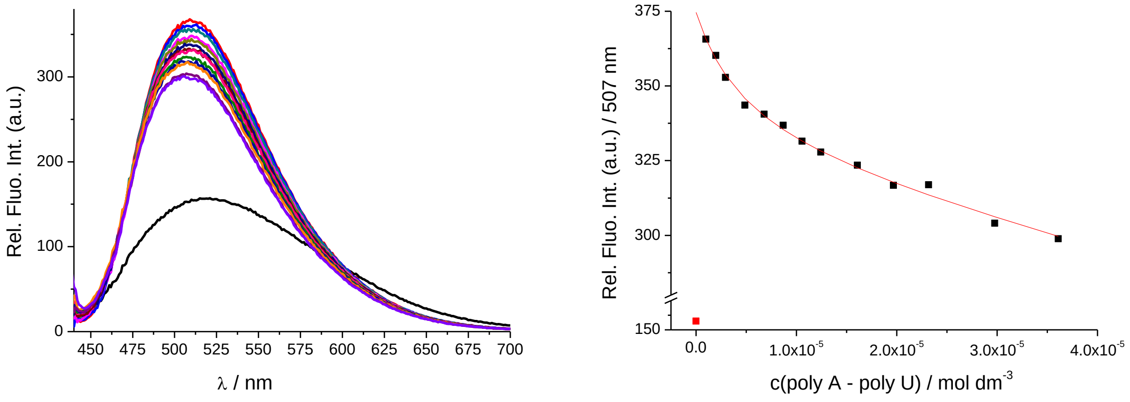


**Figure S56.** Left: Fluorimetric titration of **3** ($c$ = 5 $\times 10^{-8}$ M; $\lambda_{exc}$= 412 nm) with **poly A-poly U**. Right: dependence of fluorescence at $\lambda_{max}$ = 507 nm on $c$(RNA), red line is non-linear least square fitting of Scatchard eq. (McGhee, von Hippel formalism) to the experimental data. Measured at pH 7, sodium cacodylate buffer, $I$ = 0.05 M.

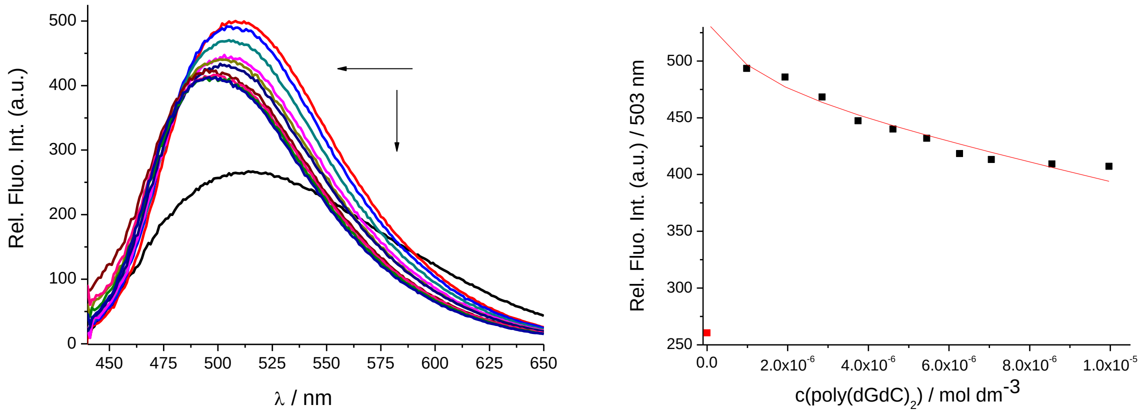


**Figure S57.** Left: Fluorimetric titration of **3** ($c$ = 5 ×10$^{-8}$ M; $\lambda_{exc}$= 412 nm) with **poly (dGdC)$_2$**. Right: dependence of fluorescence at $\lambda_{max}$ = 503 nm on $c$(DNA), red line is non-linear least square fitting of Scatchard eq. (McGhee, von Hippel formalism) to the experimental data. Measured at pH 7, sodium cacodylate buffer, $I$ = 0.05 M.

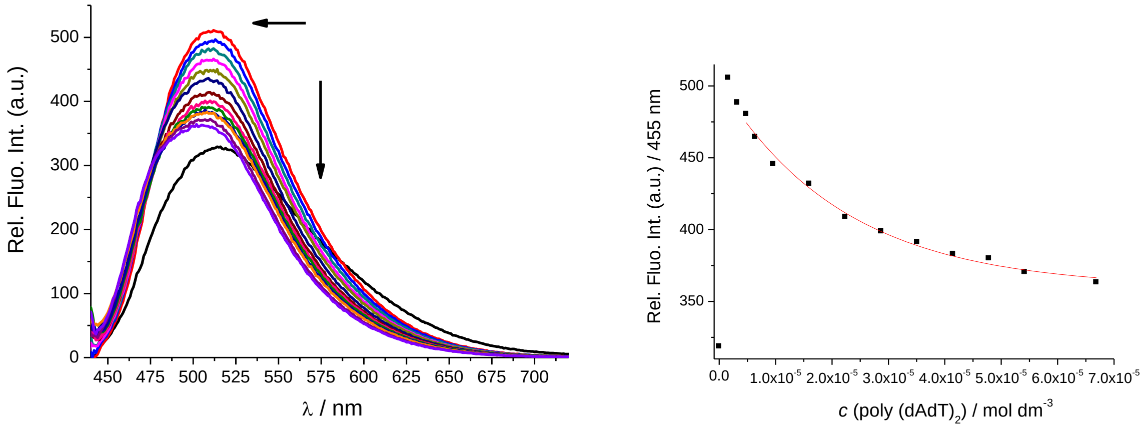


**Figure S58.** Left: Fluorimetric titration of **3** ($c$ = 5 ×10$^{-8}$ M; $\lambda_{exc}$= 412 nm) with **poly (dAdT)$_2$**. Right: dependence of fluorescence at $\lambda_{max}$ = 455 nm on $c$(DNA), red line is non-linear least square fitting of Scatchard eq. (McGhee, von Hippel formalism) to the experimental data. Measured at pH 7, sodium cacodylate buffer, $I$ = 0.05 M.

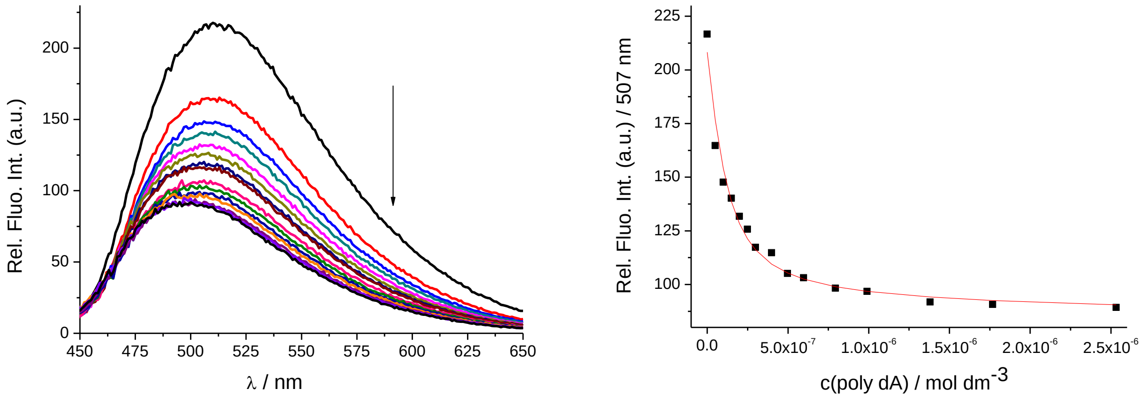


**Figure S59.** Left: Fluorimetric titration of **3** ($c$ = 5 ×10$^{-8}$ M; $\lambda_{exc}$= 412 nm) with **poly dA**. Right: dependence of fluorescence at $\lambda_{max}$ = 507 nm on $c$(DNA), red line is non-linear least square fitting of Scatchard eq. (McGhee, von Hippel formalism) to the experimental data. Measured at pH 7, sodium cacodylate buffer, $I$ = 0.05 M.

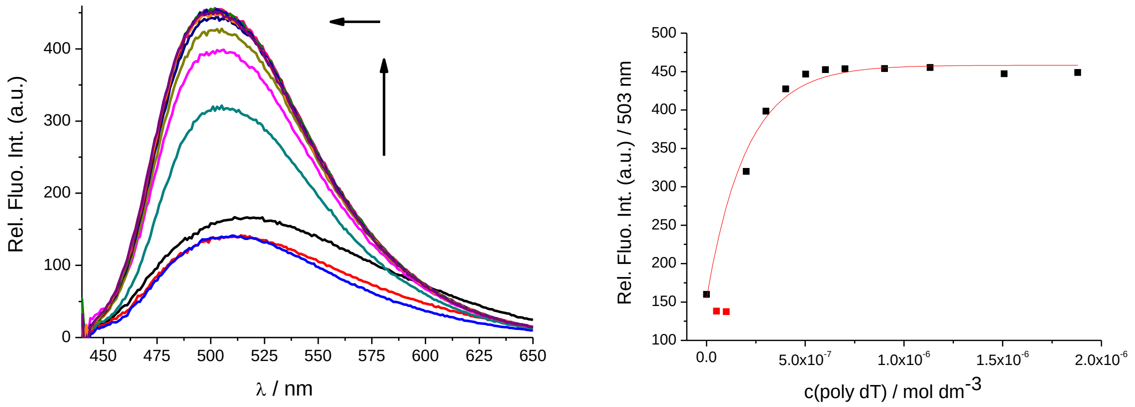


**Figure S60.** Left: Fluorimetric titration of **3** ($c$ = 5 $\times10^{-8}$ M; $\lambda_{exc}$= 412 nm) with **poly dT**. Right: dependence of fluorescence at $\lambda_{max}$ = 503 nm on $c$(DNA), red line is non-linear least square fitting of Scatchard eq. (McGhee, von Hippel formalism) to the experimental data. Measured at pH 7, sodium cacodylate buffer, $I$ = 0.05 M.

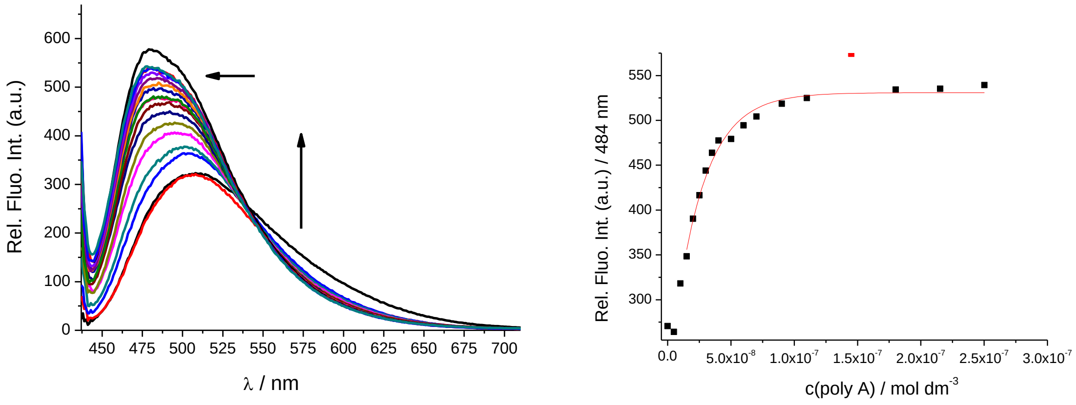


**Figure S61.** Left: Fluorimetric titration of **3** ($c$ = 5 $\times10^{-8}$ M; $\lambda_{exc}$= 412 nm) with **poly A**. Right: dependence of fluorescence at $\lambda_{max}$ = 484 nm on $c$(RNA), red line is non-linear least square fitting of Scatchard eq. (McGhee, von Hippel formalism) to the experimental data. Measured at pH 7, sodium cacodylate buffer, $I$ = 0.05 M.

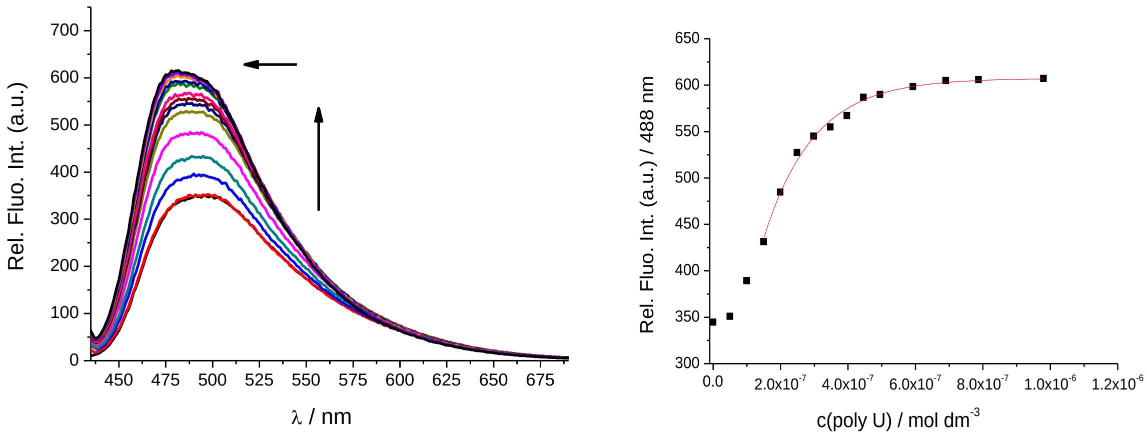


**Figure S62.** Left: Fluorimetric titration of **3** ($c$ = 5 $\times10^{-8}$ M; $\lambda_{exc}$= 412 nm) with **poly U**. Right: dependence of fluorescence at $\lambda_{max}$ = 488 nm on $c$(RNA), red line is non-linear least square fitting of Scatchard eq. (McGhee, von Hippel formalism) to the experimental data. Measured at pH 7, sodium cacodylate buffer, $I$ = 0.05 M.

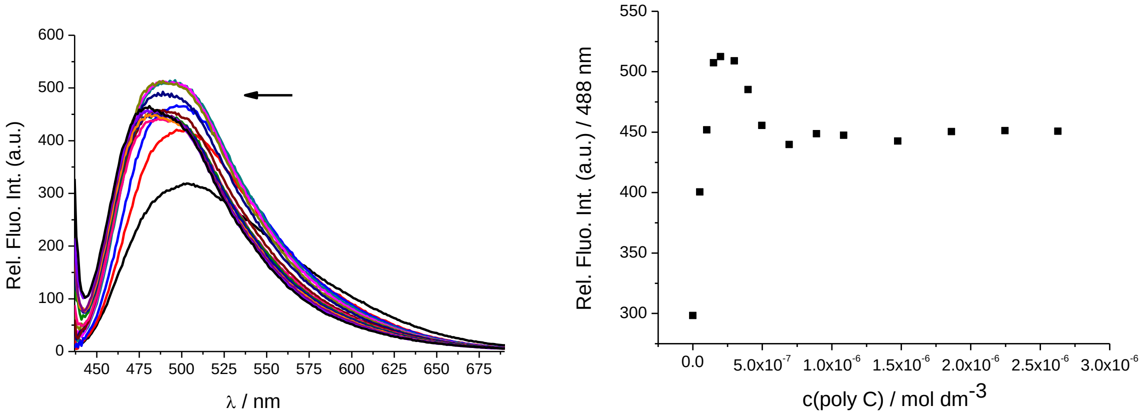


**Figure S63.** Left**:** Fluorimetric titration of **3** ($c$ = 5 $\times10^{-8}$ M; $\lambda_{exc}$= 412 nm) with **poly C**. Right: dependence of fluorescence at $\lambda_{max}$ = 488 nm. Measured at pH 7, sodium cacodylate buffer, $I$ = 0.05 M.

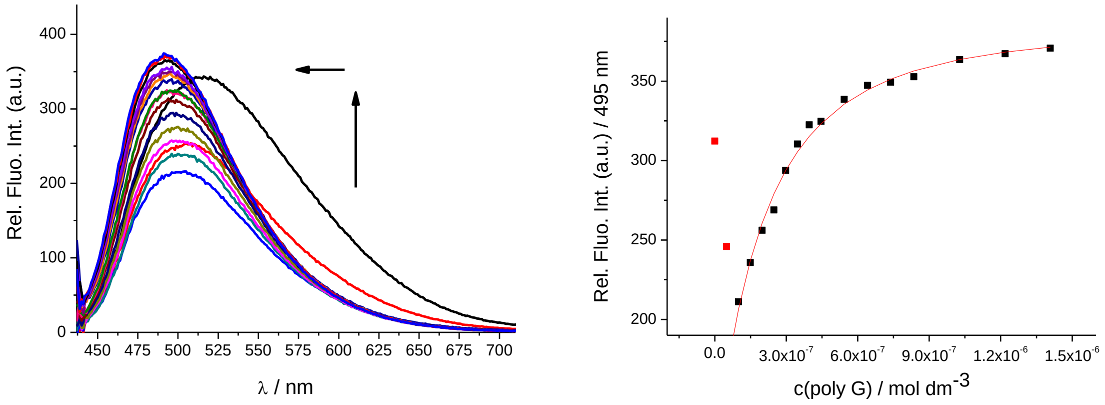


**Figure S64.** Left: Fluorimetric titration of **3** ($c$ = 5 $\times10^{-8}$ M; $\lambda_{exc}$= 412 nm) with **poly G**. Right: dependence of fluorescence at $\lambda_{max}$ = 495 nm on $c$(RNA), red line is non-linear least square fitting of Scatchard eq. (McGhee, von Hippel formalism) to the experimental data. Measured at pH 7, sodium cacodylate buffer, $I$ = 0.05 M.

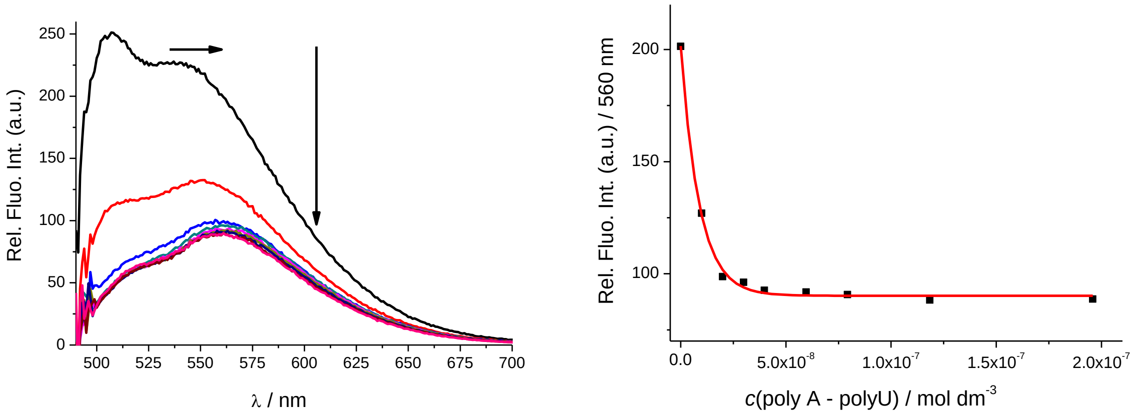


**Figure S65.** Left: Fluorimetric titration of **5** ($c$ = 1 $\times10^{-8}$ M; $\lambda_{exc}$= 470 nm) with **poly A- poly U**. Right: Dependence of fluorescence at $\lambda_{max}$ = 560 nm on $c$(RNA). Measured at pH 7, sodium cacodylate buffer, $I$ = 0.05 M.

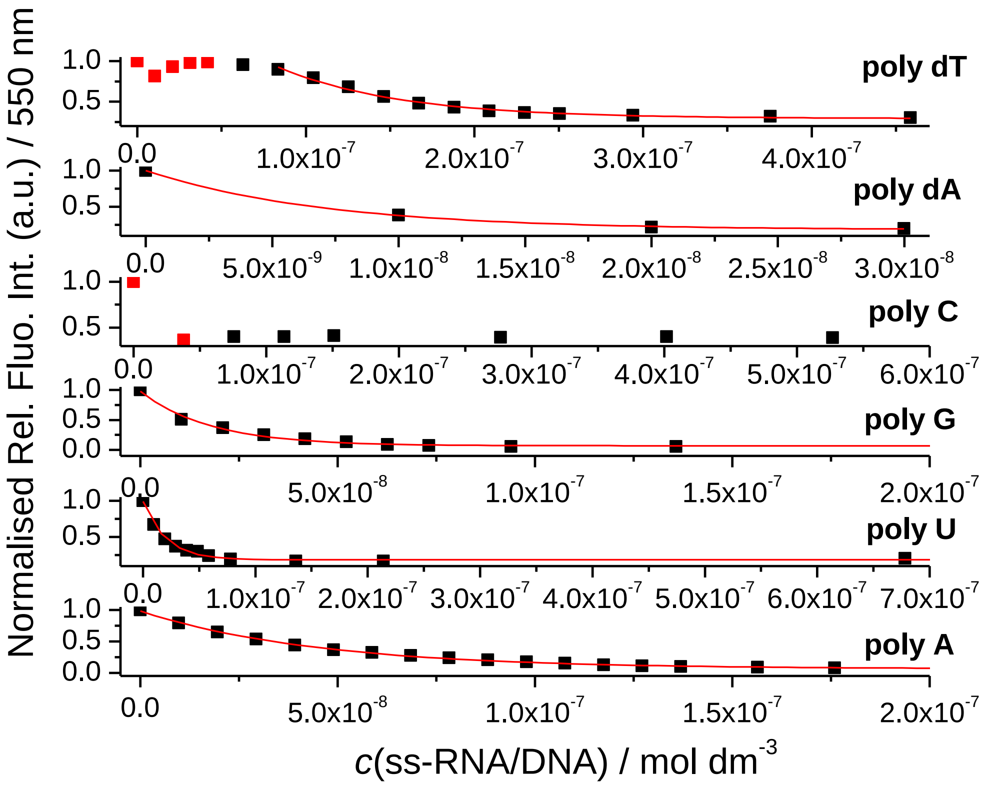


**Figure S66.** Fluorimetric titrations of **5** ($c$ = 1 ×$10^{-8}$ M; $\lambda_{exc}$= 470 nm) with **ss-DNA/RNA**; dependence of normalized fluorescence at $\lambda_{max}$ = 550 nm on $c$(DNA/RNA). Measured at pH 7, sodium cacodylate buffer, $I$ = 0.05 M.

# Circular Dichroism

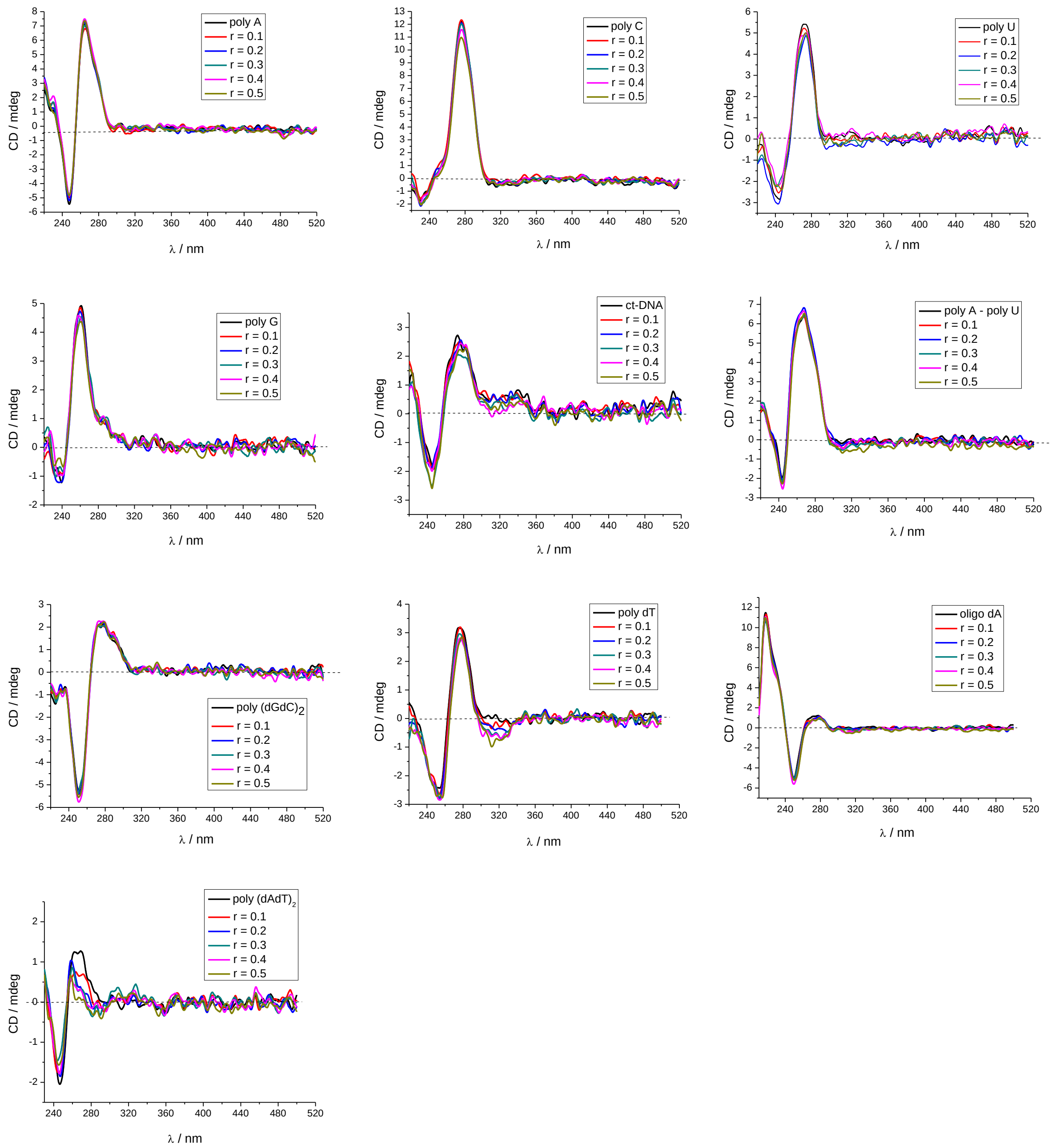


**Figure S67.** CD titration of **poly A**, **poly C**, **poly U**, **poly G**, **ct-DNA**, **poly A - poly U**, **poly (dGdC)₂**, **poly dT**, **oligo dA** and **poly (dAdT)₂** (all ss-DNA, DNA/RNA $c$ = 2×10$^{-5}$ M) with **6** at molar ratios $r$ = [compound] / [polynucleotide] (pH 7.0, sodium cacodylate buffer, $I$ = 0.05 M).

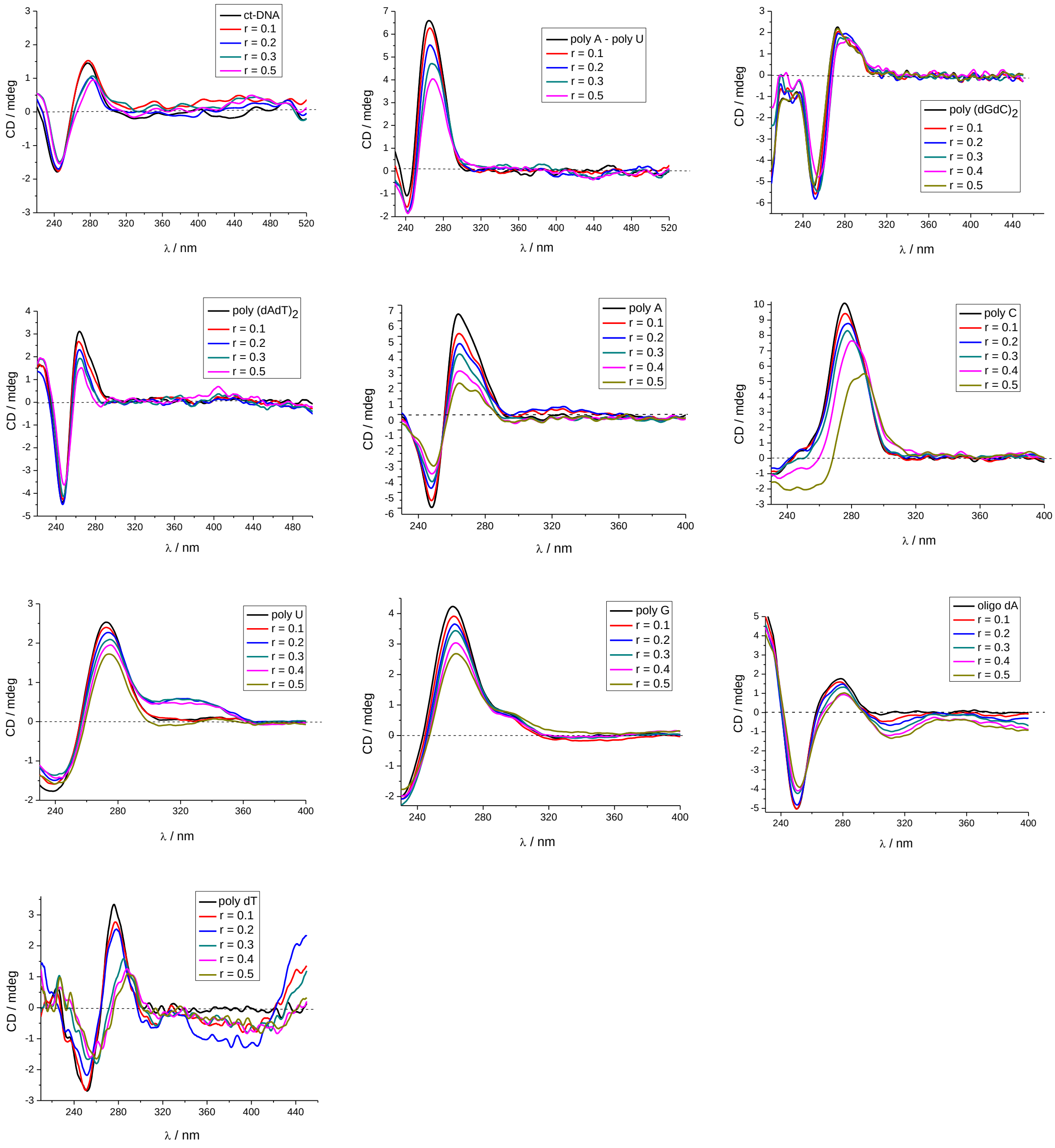


**Figure S68.** CD titration of **ctDNA**, **poly A - poly U**, **poly (dAdT)$_2$**, **poly (dGdC)$_2$**, **poly A** ($c$ = 1×10$^{-5}$ M), **poly C**, **poly U**, **poly G**, **oligo dA**, **poly dT** (all ss-DNA, DNA/RNA $c$ = 2×10$^{-5}$ M) with **3** at molar ratios $r$ = [compound] / [polynucleotide] (pH 7.0, sodium cacodylate buffer, $I$ = 0.05 M).

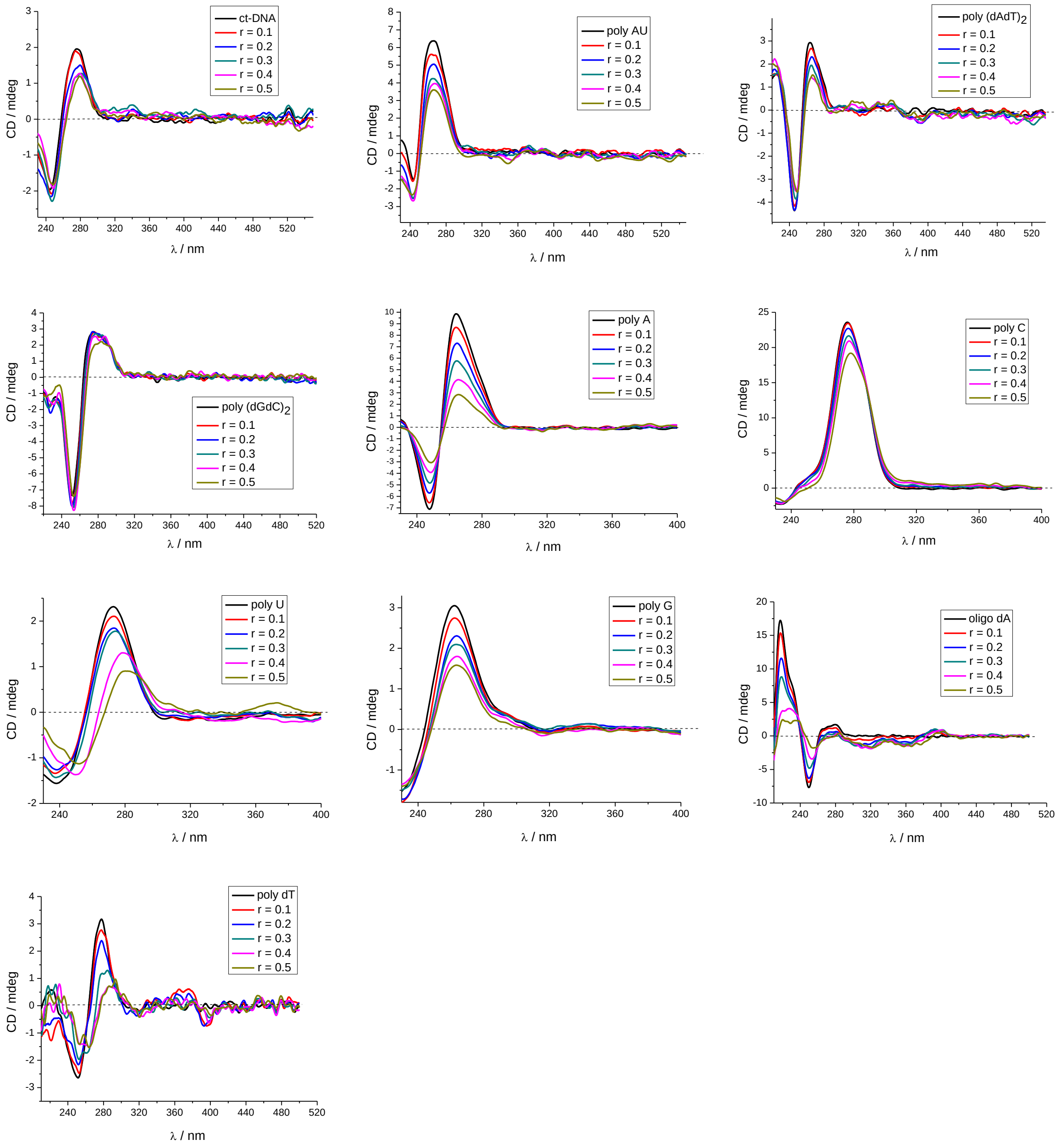


**Figure S69.** CD titration of **ctDNA**, **poly A - poly U**, **poly (dAdT)$_2$**, **poly (dGdC)$_2$**, **poly A** ($c$ = 1×10$^{-5}$ M)**, poly C**, **poly U**, **poly G**, **oligo dA**, **poly dT** (all ss-DNA, DNA/RNA $c$ = 2×10$^{-5}$ M) with **4** at molar ratios $r$ = [compound] / [polynucleotide] (pH 7.0, sodium cacodylate buffer, $I$ = 0.05 M).

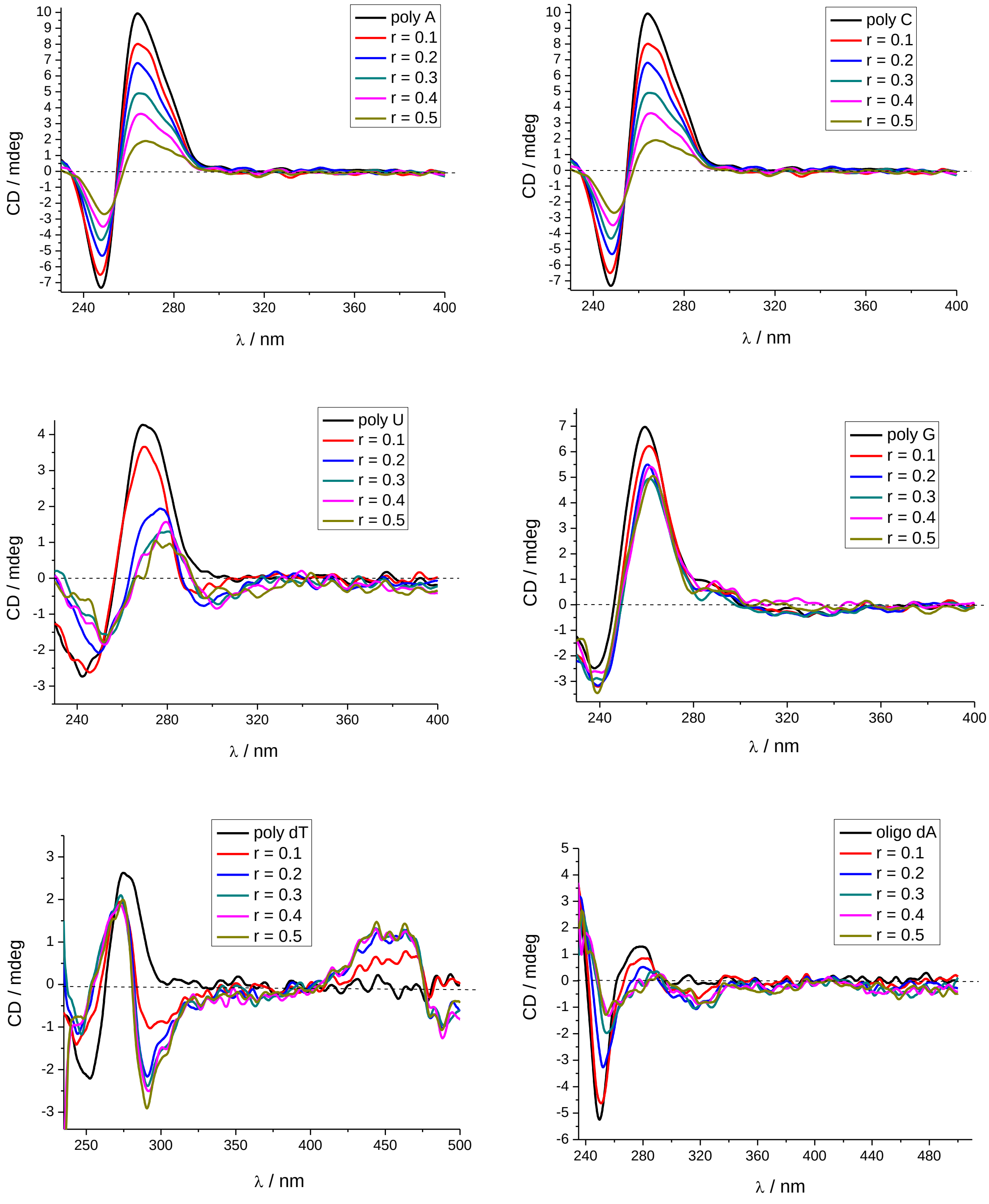


**Figure S70.** CD titrations of **poly A** ($c$ = 1×10$^{-5}$ M), **poly C**, **poly U**, **poly G**, **poly dT**, **oligo dA** (all ss-DNA/RNA $c$ = 2×10$^{-5}$ M), with compound **5** at molar ratios $r$ = [compound] / [polynucleotide] (pH 7.0, sodium cacodylate buffer, $I$ = 0.05 M).

# Raman and SERS Measurements

**Table S5.** A preliminary assignment of the Raman bands observed in the spectra of aqueous solution of **3**-**5** ($c$ = $1\times10^{-4}$ M).

| **Wavenumber / cm$^{-1}$** | | | **Assignment** |
|---|---|---|---|
| **3** | **4** | **5** | |
| | 2209 | 2182 | ν C≡C |
| 1595 | 1591 | 1594 | ν CC (aromatic linker) |
| | | 1556 | ν CC (anthracene) |
| | | 1481 | ν CC (anthracene) |
| 1472 | | | ν CC (thiophene) |
| 1453 | | | ν CS (thiophene) |
| | | 1257 | $\delta_{ip}$ CH (anthracene) |
| | | 1158 | $\delta_{ip}$ CH (anthracene) |

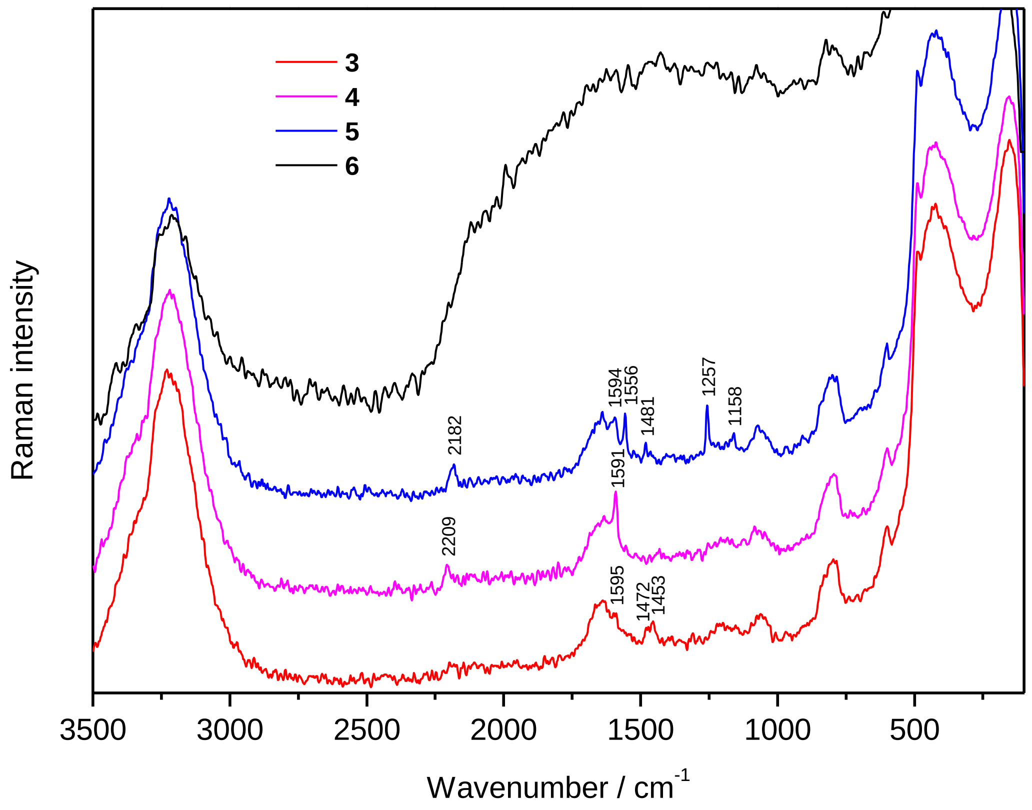


**Figure S71.** Raman spectra of aqueous solution of **3**-**5** ($c$ = 1 $\times10^{-4}$ M) and **6** ($c$ = 2 $\times10^{-3}$ M); $\lambda_{ex}$ = 1064 nm. Spectra are displaced for visual clarity.

**Table S6.** A preliminary assignment of the SERS bands observed in the spectra of **3**-**5** ($c$ = 1 $\times 10^{-6}$ M) and **6** ($c$ = 5 $\times 10^{-6}$ M).

| Wavenumber / $cm^{-1}$ | | | | Assignment |
|---|---|---|---|---|
| **3** | **4** | **5** | **6** | |
| 2196 | 2205 | 2184 | | $\nu$ C≡C |
| 1594 | 1591 | 1594 | 1588 | $\nu$ CC (aromatic linker) |
| | | 1557 | 1549 | $\nu$ CC (anthracene) |
| 1546 | | | | $\nu$ ring (thiophene) |
| | | 1483 | | $\nu$ CC (anthracene) |
| | | | 1463 | $\nu$ CC (phenyl) |
| 1473 | | | | $\nu$ CC (thiophene) |
| 1455 | | | | $\nu$ CS (thiophene) |
| | | | 1421 | $\nu$ CC (phenyl) |
| 1403 | 1395 | 1407 | 1405 | citrates |
| | | | 1329 | $\delta_{ip}$ CH (anthracene) |
| 1310 | | | | $\nu$ ring (thiophene) |
| | | 1257 | 1263 | $\delta_{ip}$ CH (anthracene) |
| 1242 | | | | $\delta_{ip}$ CH (thiophene) |
| | 1227 | | | $\delta_{ip}$ CH (phenyl) |
| | | | 1224 | $\delta_{ip}$ CH (anthracene) |
| 1192 | | | | $\delta_{ip}$ CH (thiophene) |
| | 1179 | | | $\delta_{ip}$ CH (phenyl) |
| | | 1169 | 1160 | $\delta_{ip}$ CH (anthracene) |
| | 1126 | | | $\delta_{ip}$ CH (phenyl) |
| 1083 | 1083 | 1073 | 1081 | $\nu$ B–$(C_{ar})_3$ |
| 1045 | | | | $\delta_{ip}$ CH (thiophene) |
| | | 1022 | 1040 | $\delta_{ip}$ CH (anthracene) |
| 951 | 953 | 946 | | $\delta_{ip}$ CH (phenyl) |
| 843 | | | | $\nu$ ring (thiophene) |
| 799 | 803 | 800 | 769 | $\delta_{oop}$ CH (phenyl) |
| 574 | 576 | 577 | | $\delta$ CC (phenyl) |

# Theoretical Studies

## TD-DFT Calculations

For compounds **3**-**5,** DFT and TD-DFT studies were carried out at the B3LYP/6-31G(d) (optimization) and CAM-B3LYP/6-31G(d) level of theory, respectively. The calculated lowest energy transitions for compounds **3**-**5** in the gas phase underestimate the transition energies as compared to the data obtained in MeCN solution ( **3**: $\lambda_{max, abs}$ (MeCN) = 413 nm, calc. $S_1 \leftarrow S_0$ = 483 nm; **4**: $\lambda_{max, abs}$ (MeCN) = 373 nm, calc. $S_1 \leftarrow S_0$ = 415 nm; **5**: $\lambda_{max, abs}$ (MeCN) = 483 nm, calc. $S_1 \leftarrow S_0$ = 538 nm). The trend in absorption energies between the three compounds is well reproduced (**4** > **3** > **5**). In all cases, the $S_1 \leftarrow S_0$ transitions can be predominantly attributed (contribution > 60%) to HOMO to LUMO transitions. In all three cases, the HOMO is localized at the bridge moiety (e.g. bithiophene, phenyl, or anthracene and alkynes, as well as minor contributions from the boron bound xylene moiety), and the LUMO is mainly localized at the two boron moieties (with minor contributions from the bridge bound xylene and terminal *N,N,N*,3,5-pentamethylbenzenaminium moieties).

Compound **3**:

**Calculated absorption spectra**

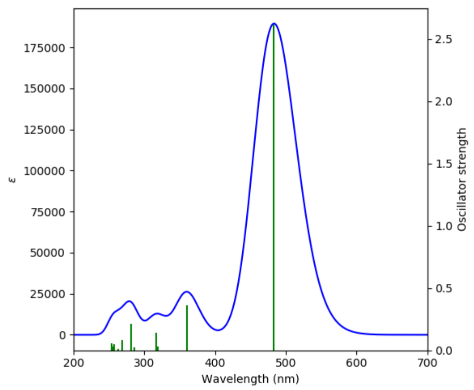


**TD-DFT CAM-B3LYP/6-31G(d), gas phase**

| Orbital | Energy [eV] | Symmetry |
|---|---|---|
| L+4 | -5.64 | $A_u$ |
| L+3 | -5.64 | $A_g$ |
| L+2 | -5.81 | $A_u$ |
| L+1 | -6.79 | $A_g$ |
| LUMO | -6.87 | $A_u$ |
| HOMO | -10.95 | $A_g$ |
| H-1 | -12.14 | $A_u$ |
| H-2 | -12.99 | $A_u$ |
| H-3 | -13.15 | $A_g$ |
| H-4 | -13.19 | $A_g$ |

**Orbitals relevant to the $S_1 \leftarrow S_0$ transition**

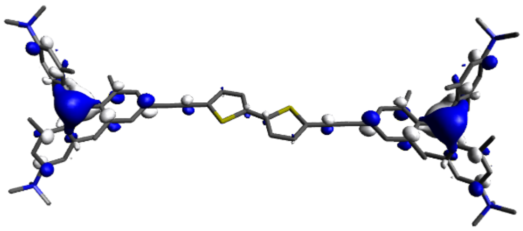

**LUMO**

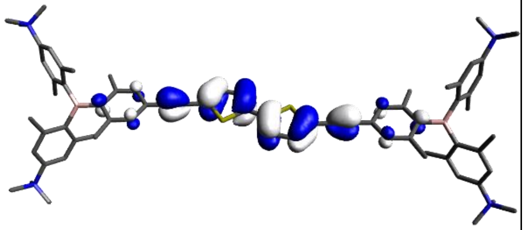

**HOMO**

**other relevant orbitals**

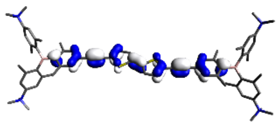

**HOMO-1**

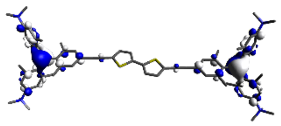

**LUMO+1**

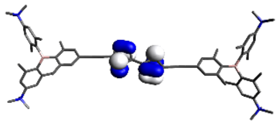

**HOMO-2**

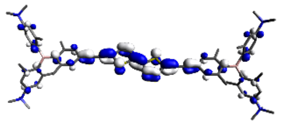

**LUMO+2**

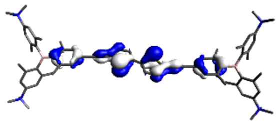

**HOMO-3**

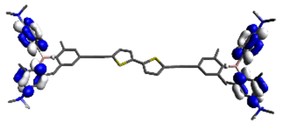

**LUMO+3**

**Table S7.** Lowest energy singlet electronic transition of **3** (TD-DFT CAM-B3LYP/6-31G(d), gas phase).

| State | E [eV] | λ [nm] | *f* | Symmetry | Major contributions |
|---|---|---|---|---|---|
| **1** | 2.56 | 483.41 | 2.616 | $A_U$ | H-1->L+1 (10%), HOMO->LUMO (66%), HOMO->L+2 (16%) |
| **2** | 2.92 | 425.33 | 0.0 | $A_G$ | H-1->LUMO (16%), HOMO->L+1 (73%) |
| **3** | 3.44 | 360.22 | 0.3595 | $A_U$ | HOMO->L+2 (65%) |
| **4** | 3.80 | 325.99 | 0.0 | $A_G$ | H-1->LUMO (45%), HOMO->L+1 (23%) |
| **5** | 3.89 | 318.76 | 0.0 | $A_G$ | H-6->L+1 (43%), H-5->LUMO (48%) |
| **6** | 3.89 | 318.75 | 0.0278 | $A_U$ | H-6->LUMO (47%), H-5->L+1 (43%) |
| **7** | 3.91 | 317.18 | 0.1435 | $A_U$ | H-3->LUMO (12%), H-1->L+1 (35%), HOMO->LUMO (23%) |
| **8** | 4.09 | 303.17 | 0.0 | $A_G$ | H-1->L+2 (18%), HOMO->L+5 (49%) |
| **9** | 4.35 | 285.33 | 0.0 | $A_G$ | HOMO->L+3 (83%) |
| **10** | 4.35 | 285.33 | 0.0221 | $A_U$ | HOMO->L+4 (83%) |
| **11** | 4.40 | 281.56 | 0.0 | $A_G$ | H-11->LUMO (42%), H-10->L+1 (42%) |
| **12** | 4.40 | 281.54 | 0.211 | $A_U$ | H-11->L+1 (42%), H-10->LUMO (42%) |
| **13** | 4.54 | 272.89 | 0.0 | $A_G$ | H-9->LUMO (12%), H-1->L+2 (21%), HOMO->L+5 (20%) |
| **14** | 4.57 | 271.54 | 0.0005 | $A_U$ | H-8->L+1 (17%), H-8->L+5 (10%), H-7->LUMO (35%), H-7->L+2 (23%) |
| **15** | 4.58 | 270.96 | 0.0 | $A_G$ | H-8->LUMO (32%), H-8->L+2 (22%), H-7->L+1 (18%), H-7->L+5 (11%) |
| **16** | 4.61 | 269.21 | 0.0846 | $A_U$ | H-1->L+5 (10%), HOMO->L+8 (18%), HOMO->L+10 (24%) |
| **17** | 4.66 | 266.00 | 0.0 | $A_G$ | H-2->LUMO (44%), H-2->L+2 (36%) |
| **18** | 4.72 | 262.49 | 0.0124 | $A_U$ | H-9->L+1 (11%), H-1->L+1 (28%) |
| **19** | 4.81 | 257.84 | 0.0 | $A_G$ | H-13->LUMO (22%), H-12->L+1 (26%), H-1->L+2 (11%) |
| **20** | 4.81 | 257.67 | 0.0506 | $A_U$ | H-4->LUMO (11%), H-3->LUMO (10%), HOMO->L+8 (18%) |
| **21** | 4.81 | 257.56 | 0.0 | $A_G$ | HOMO->L+7 (71%) |
| **22** | 4.81 | 257.56 | 0.0053 | $A_U$ | HOMO->L+6 (70%) |
| **23** | 4.85 | 255.43 | 0.0291 | $A_U$ | H-13->L+1 (22%), H-12->LUMO (15%), H-3->LUMO (10%) |
| **24** | 4.87 | 254.37 | 0.0 | $A_G$ | H-15->LUMO (21%), H-14->L+1 (22%), HOMO->L+7 (15%) |
| **25** | 4.87 | 254.34 | 0.0538 | $A_U$ | H-15->L+1 (27%), H-14->LUMO (26%), HOMO->L+6 (17%) |

Compound **4**:

<table>
<tr><th>Calculated absorption spectra</th><th>Orbital</th><th>Energy [eV]</th><th>Symmetry</th></tr>
<tr><td rowspan="10">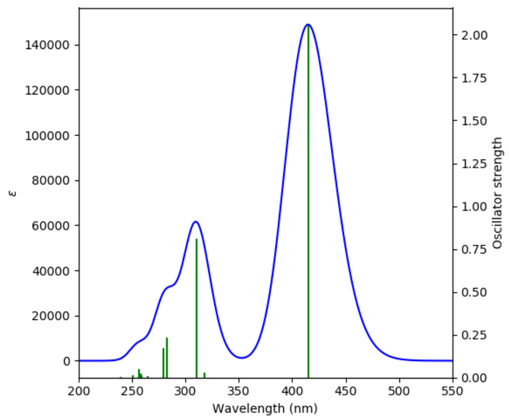

TD-DFT CAM-B3LYP/6-31+G(d, p), gas phase</td><td>L+4</td><td>-5.81</td><td>Ag</td></tr>
<tr><td>L+3</td><td>-5.81</td><td>Au</td></tr>
<tr><td>L+2</td><td>-5.90</td><td>Au</td></tr>
<tr><td>L+1</td><td>-6.97</td><td>Ag</td></tr>
<tr><td>LUMO</td><td>-7.08</td><td>Au</td></tr>
<tr><td>HOMO</td><td>-11.82</td><td>Ag</td></tr>
<tr><td>H-1</td><td>-12.92</td><td>Au</td></tr>
<tr><td>H-2</td><td>-13.42</td><td>Ag</td></tr>
<tr><td>H-3</td><td>-13.46</td><td>Au</td></tr>
<tr><td>H-4</td><td>-13.46</td><td>Ag</td></tr>
<tr><th>Orbitals relevant to the $S_1 \leftarrow S_0$ transition</th><th colspan="3">other relevant orbitals</th></tr>
<tr><td>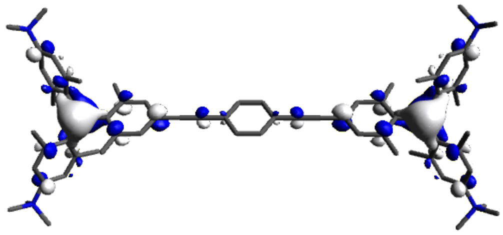<br>LUMO<br>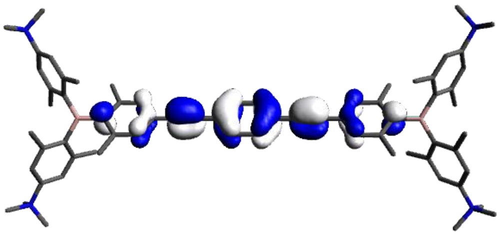<br>HOMO</td><td>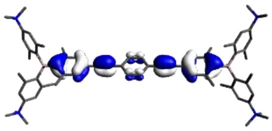<br>HOMO-1<br>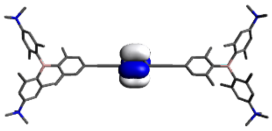<br>HOMO-2<br>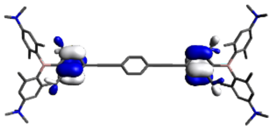<br>HOMO-3</td><td colspan="2">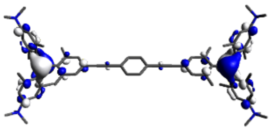<br>LUMO+1<br>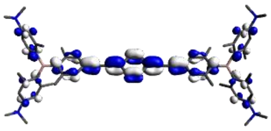<br>LUMO+2<br>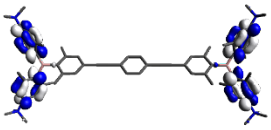<br>LUMO+3</td></tr>
</table>

**Table S 8.** Lowest energy singlet electronic transition of **4** (TD-DFT CAM-B3LYP/6-31+G(d), gas phase).

| State | E [eV] | λ [nm] | *f* | Symmetry | Major contributions |
|---|---|---|---|---|---|
| **1** | 2.99 | 414.88 | 2.0547 | AU | H-1->L+1 (15%), HOMO->LUMO (69%) |
| **2** | 3.27 | 379.47 | 0 | AG | H-1->LUMO (24%), HOMO->L+1 (63%) |
| **3** | 3.90 | 318.05 | 0 | AG | H-4->L+1 (41%), H-3->LUMO (49%) |
| **4** | 3.90 | 318.02 | 0.0274 | AU | H-4->LUMO (49%), H-3->L+1 (42%) |
| **5** | 4.00 | 310.10 | 0.8103 | AU | H-1->L+1 (15%), HOMO->L+2 (57%) |
| **6** | 4.30 | 288.19 | 0 | AG | H-6->L+1 (12%), H-1->LUMO (35%), HOMO->L+1 (33%) |
| **7** | 4.38 | 283.04 | 0 | AG | H-9->LUMO (44%), H-8->L+1 (43%) |
| **8** | 4.38 | 283.01 | 0.2341 | AU | H-9->L+1 (43%), H-8->LUMO (44%) |
| **9** | 4.43 | 279.79 | 0.174 | AU | H-6->LUMO (14%), H-1->L+1 (19%), HOMO->LUMO (28%), HOMO->L+2 (18%) |
| **10** | 4.55 | 272.61 | 0 | AG | H-5->LUMO (40%), H-5->L+2 (29%) |
| **11** | 4.57 | 271.22 | 0 | AU | H-7->LUMO (29%), H-7->L+2 (19%), H-5->L+1 (14%) |
| **12** | 4.68 | 264.78 | 0.0094 | AU | H-2->LUMO (37%), H-2->L+2 (29%), HOMO->L+18 (19%) |
| **13** | 4.69 | 264.61 | 0 | AG | H-1->L+2 (27%), HOMO->L+5 (32%), HOMO->L+9 (10%) |
| **14** | 4.79 | 258.76 | 0 | AG | H-1->L+3 (15%), HOMO->L+4 (73%) |
| **15** | 4.79 | 258.76 | 0.0134 | AU | H-1->L+4 (15%), HOMO->L+3 (73%) |
| **16** | 4.81 | 257.78 | 0.0249 | AU | H-11->LUMO (35%), H-10->L+1 (31%) |
| **17** | 4.81 | 257.60 | 0 | AG | H-11->L+1 (33%), H-10->LUMO (28%) |
| **18** | 4.84 | 256.23 | 0 | AG | H-13->LUMO (32%), H-12->L+1 (33%) |
| **19** | 4.84 | 256.22 | 0.0521 | AU | H-13->L+1 (33%), H-12->LUMO (33%) |
| **20** | 4.91 | 252.37 | 0 | AG | H-15->L+1 (33%), H-14->LUMO (32%), H-1->LUMO (11%) |
| **21** | 4.94 | 250.82 | 0.013 | AU | H-15->LUMO (37%), H-14->L+1 (28%), H-1->L+1 (15%) |
| **22** | 5.18 | 239.23 | 0.002 | AU | H-4->L+2 (31%), H-3->L+5 (10%), H-1->L+12 (11%), HOMO->L+11 (18%) |
| **23** | 5.18 | 239.23 | 0 | AG | H-4->L+5 (10%), H-3->L+2 (32%), H-1->L+11 (11%), HOMO->L+12 (18%) |
| **24** | 5.27 | 235.05 | 0 | AG | H-16->LUMO (12%), H-1->L+2 (10%), HOMO->L+5 (39%) |
| **25** | 5.31 | 233.58 | 0 | AG | H-1->L+6 (14%), HOMO->L+7 (81%) |

Compound **5**:

**Calculated absorption spectra**

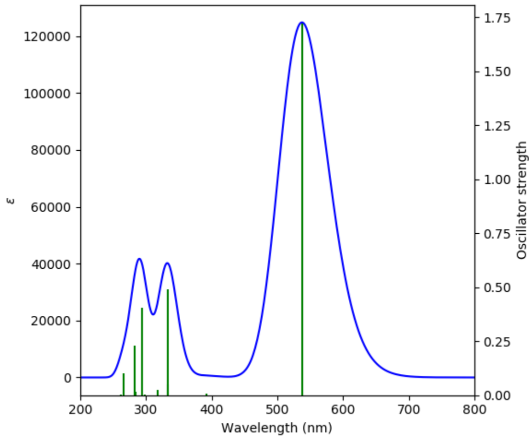


**TD-DFT CAM-B3LYP/6-31+G(d, p), gas phase**

| Orbital | Energy [eV] | Symmetry |
|---|---|---|
| L+4 | -5.82 | $A_g$ |
| L+3 | -5.82 | $A_u$ |
| L+2 | -6.21 | $A_u$ |
| L+1 | -6.95 | $A_g$ |
| LUMO | -7.12 | $A_u$ |
| HOMO | -10.92 | $A_g$ |
| H-1 | -12.62 | $A_g$ |
| H-2 | -12.81 | $A_g$ |
| H-3 | -12.84 | $A_u$ |
| H-4 | -13.14 | $A_u$ |

**Orbitals relevant to the $S_1 \leftarrow S_0$ transition**

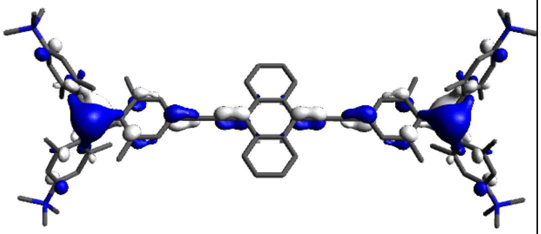

**LUMO**

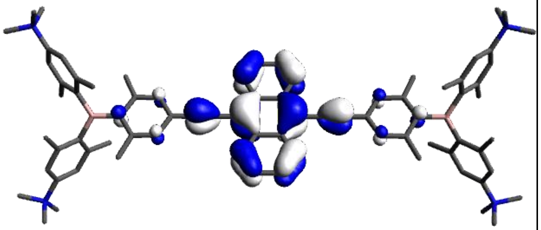

**HOMO**

**other relevant orbitals**

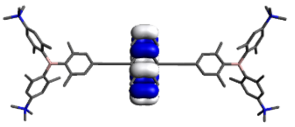

**HOMO-1**

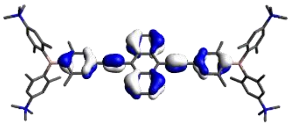

**HOMO-2**

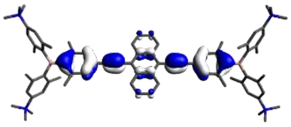

**HOMO-3**

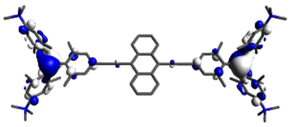

**LUMO+1**

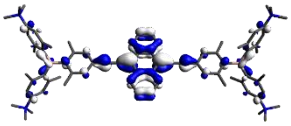

**LUMO+2**

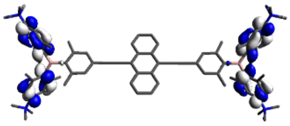

**LUMO+3**

**Table S9.** Lowest energy singlet electronic transition of **5** (TD-DFT CAM-B3LYP/6-31G(d), gas phase).

| State | E [eV] | λ [nm] | *f* | Symmetry | Major contributions |
|---|---|---|---|---|---|
| **1** | 2.31 | 537.61 | 1.7224 | $A_U$ | HOMO->LUMO (76%), HOMO->L+2 (17%) |
| **2** | 2.80 | 443.50 | 0.0 | $A_G$ | HOMO->L+1 (87%) |
| **3** | 3.16 | 392.72 | 0.0082 | $A_U$ | HOMO->LUMO (13%), HOMO->L+2 (72%) |
| **4** | 3.72 | 333.26 | 0.0487 | $A_U$ | H-1->LUMO (35%), H-1->L+2 (36%), HOMO->L+13 (22%) |
| **5** | 3.72 | 333.20 | 0.4889 | $A_U$ | H-3->L+1 (24%), H-2->LUMO (47%) |
| **6** | 3.73 | 332.23 | 0.0 | $A_G$ | H-3->LUMO (48%), H-2->L+1 (24%) |
| **7** | 3.89 | 318.40 | 0.0 | $A_G$ | H-6->LUMO (50%), H-5->L+1 (40%) |
| **8** | 3.89 | 318.38 | 0.0243 | $A_U$ | H-6->L+1 (40%), H-5->LUMO (50%) |
| **9** | 4.16 | 298.29 | 0.0 | $A_G$ | HOMO->L+4 (94%) |
| **10** | 4.16 | 298.29 | 0.0051 | $A_U$ | HOMO->L+3 (94%) |
| **11** | 4.17 | 297.10 | 0.0 | $A_G$ | HOMO->L+5 (57%), HOMO->L+9 (21%) |
| **12** | 4.22 | 294.14 | 0.4029 | $A_U$ | H-2->L+2 (22%), HOMO->L+10 (35%) |
| **13** | 4.27 | 290.40 | 0.0 | $A_G$ | H-4->LUMO (51%), H-4->L+2 (44%) |
| **14** | 4.37 | 283.57 | 0.0158 | $A_U$ | H-2->L+2 (26%), HOMO->L+10 (23%) |
| **15** | 4.39 | 282.38 | 0.0 | $A_G$ | H-12->LUMO (43%), H-11->L+1 (43%) |
| **16** | 4.39 | 282.35 | 0.2297 | $A_U$ | H-12->L+1 (43%), H-11->LUMO (43%) |
| **17** | 4.39 | 282.27 | 0.0004 | $A_U$ | H-7->LUMO (46%), H-7->L+2 (30%) |
| **18** | 4.46 | 277.92 | 0.0 | $A_G$ | H-8->LUMO (45%), H-8->L+2 (27%) |
| **19** | 4.53 | 273.90 | 0.0 | $A_G$ | H-3->LUMO (12%), H-3->L+2 (43%) |
| **20** | 4.65 | 266.53 | 0.0994 | $A_U$ | H-1->LUMO (60%), HOMO->L+13 (26%) |
| **21** | 4.66 | 265.84 | 0.0 | $A_G$ | H-1->L+1 (96%) |
| **22** | 4.68 | 265.17 | 0.0002 | $A_U$ | HOMO->L+6 (95%) |
| **23** | 4.68 | 265.16 | 0.0 | $A_G$ | HOMO->L+7 (95%) |
| **24** | 4.74 | 261.61 | 0.0025 | $A_U$ | HOMO->L+8 (67%), HOMO->L+10 (14%) |
| **25** | 4.74 | 261.43 | 0.0 | $A_G$ | HOMO->L+5 (22%), HOMO->L+9 (54%) |

## Calculated Raman Data

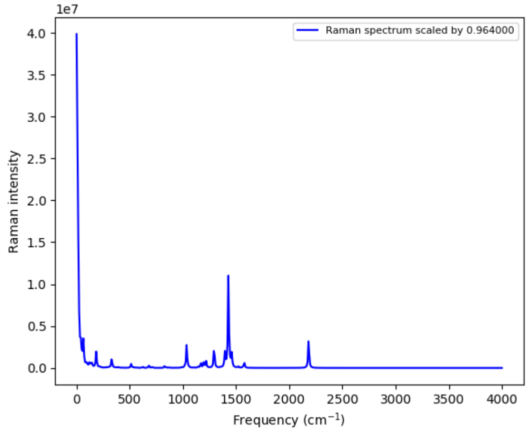


Compound **3**

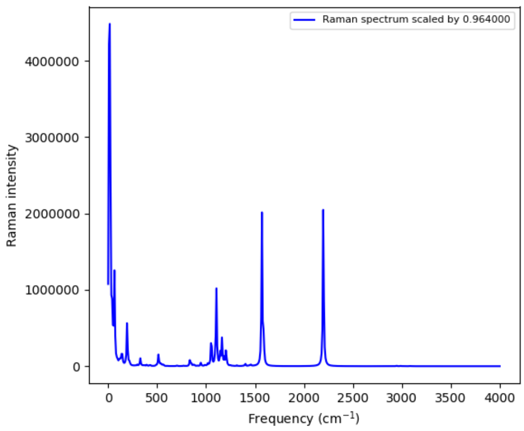


Compound **4**

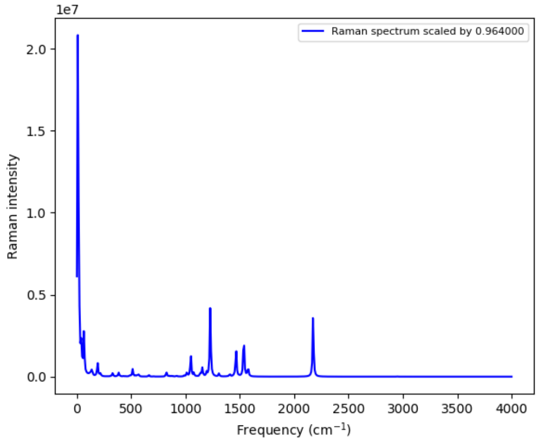


Compound **5**

**Table S10.** Comparison of experimentally observed (exp.) and calculated (calc.) Raman bands of compounds **3**-**5**.

| Wavenumber / cm$^{-1}$ | | | | | | Assignment |
|---|---|---|---|---|---|---|
| **3** | | **4** | | **5** | | |
| **exp.** | **calc.** | **exp.** | **calc.** | **exp.** | **calc.** | |
| - | 2183 | 2209 | 2198 | 2182 | 2174 | ν C≡C |
| 1595 | 1578 | 1591 | 1570 | 1594 | 1576 | ν CC (aromatic linker) |
| | | | | 1556 | 1535 | ν CC (anthracene) |
| | | | | 1481 | 1465 | ν CC (anthracene) |
| 1472 | | | | | | ν CC (thiophene) |
| 1453 | 1428 | | | | | ν CS (thiophene) |
| | | | | 1257 | 1227 | $\delta_{ip}$ CH (anthracene) |
| | | | | 1158 | 1154 | $\delta_{ip}$ CH (anthracene) |

## Cartesian Coordinates

Compound **3**

DFT B3LYP/6-31G(d), gas phase, $S_0$

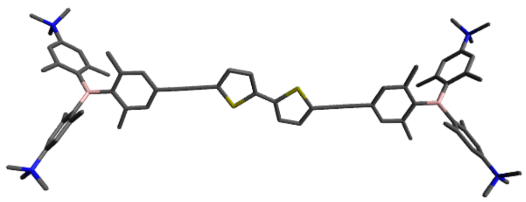

Point group: $C_i$

Total energy: – 2,422,550.60 kcal mol$^{-1}$

Dipole moment: 0 D

Immaginary frequencies: 0

| | | | |
|---|---|---|---|
| C | -0.66075900 | 0.20042800 | 0.21038100 |
| C | 0.66075900 | -0.20042800 | -0.21038100 |
| C | 1.01265800 | -1.13430300 | -1.17411100 |
| C | 2.40240900 | -1.28684100 | -1.33847200 |
| C | 3.15113900 | -0.47075900 | -0.50226600 |
| S | 2.09401400 | 0.50672000 | 0.50968900 |
| C | -1.01265800 | 1.13430300 | 1.17411100 |
| C | -2.40240900 | 1.28684100 | 1.33847200 |
| C | -3.15113900 | 0.47075900 | 0.50226600 |
| S | -2.09401400 | -0.50672000 | -0.50968900 |
| H | 0.28148100 | -1.69412900 | -1.74726000 |
| H | 2.85989400 | -1.96961400 | -2.04470900 |
| H | -0.28148100 | 1.69412900 | 1.74726000 |
| H | -2.85989400 | 1.96961400 | 2.04470900 |
| C | -4.54456100 | 0.37520500 | 0.41104100 |
| C | 4.54456100 | -0.37520500 | -0.41104100 |
| C | 5.76011400 | -0.29161800 | -0.33059000 |
| C | -5.76011400 | 0.29161800 | 0.33059000 |
| C | 7.17165200 | -0.21699300 | -0.25858700 |
| C | 7.97970700 | -1.04234800 | -1.06393800 |
| C | 7.81200600 | 0.68073800 | 0.61648000 |
| C | 9.36910400 | -1.00128200 | -0.99378800 |
| H | 7.49740000 | -1.72669300 | -1.75505800 |
| C | 9.19941600 | 0.77644400 | 0.68141600 |
| H | 7.20037100 | 1.31193100 | 1.25379200 |
| C | 10.02680900 | -0.07634500 | -0.11922000 |
| C | -7.17165200 | 0.21699300 | 0.25858700 |
| C | -7.81200600 | -0.68073800 | -0.61648000 |
| C | -7.97970700 | 1.04234800 | 1.06393800 |
| C | -9.19941600 | -0.77644400 | -0.68141600 |
| H | -7.20037100 | -1.31193100 | -1.25379200 |
| C | -9.36910400 | 1.00128200 | 0.99378800 |
| H | -7.49740000 | 1.72669300 | 1.75505800 |
| C | -10.02680900 | 0.07634500 | 0.11922000 |
| C | -10.11535100 | 1.94362100 | 1.92143700 |
| H | -9.43160400 | 2.36281900 | 2.66472400 |
| H | -10.56118900 | 2.78447400 | 1.37900000 |
| H | -10.92270900 | 1.44810900 | 2.47005900 |
| C | -9.75679900 | -1.78464900 | -1.67044700 |
| H | -8.96559800 | -2.13764600 | -2.33760400 |
| H | -10.17436100 | -2.66364500 | -1.16738500 |
| H | -10.54784900 | -1.36717800 | -2.30165800 |
| C | 9.75679900 | 1.78464900 | 1.67044700 |
| H | 10.17436100 | 2.66364500 | 1.16738500 |
| H | 8.96559800 | 2.13764600 | 2.33760400 |
| H | 10.54784900 | 1.36717800 | 2.30165800 |
| C | 10.11535100 | -1.94362100 | -1.92143700 |
| H | 10.56118900 | -2.78447400 | -1.37900000 |
| H | 9.43160400 | -2.36281900 | -2.66472400 |
| H | 10.92270900 | -1.44810900 | -2.47005900 |
| B | 11.58676400 | -0.00234400 | -0.04106100 |
| B | -11.58676400 | 0.00234400 | 0.04106100 |
| C | 12.47091800 | -1.34058600 | -0.00380900 |
| C | 13.53279800 | -1.57115100 | -0.91334400 |
| C | 12.20213400 | -2.34032800 | 0.97207400 |
| C | 14.27916600 | -2.75986200 | -0.86259900 |
| C | 12.97529900 | -3.50649400 | 1.02050300 |
| C | 13.99999800 | -3.71879400 | 0.10215700 |
| H | 15.06635700 | -2.89452300 | -1.59286200 |
| H | 12.74294800 | -4.23986800 | 1.78586300 |
| C | -12.47091800 | 1.34058600 | 0.00380900 |
| C | -12.20213400 | 2.34032800 | -0.97207400 |
| C | -13.53279800 | 1.57115100 | 0.91334400 |
| C | -12.97529900 | 3.50649400 | -1.02050300 |
| C | -14.27916600 | 2.75986200 | 0.86259900 |
| C | -13.99999800 | 3.71879400 | -0.10215700 |
| H | -12.74294800 | 4.23986800 | -1.78586300 |
| H | -15.06635700 | 2.89452300 | 1.59286200 |
| C | -12.34221000 | -1.41224500 | -0.00215200 |
| C | -12.08231700 | -2.38475500 | 1.00318500 |
| C | -13.28260100 | -1.73711100 | -1.01110700 |
| C | -12.74890300 | -3.61574500 | 0.98440000 |
| C | -13.92110300 | -2.98803700 | -1.02461200 |
| C | -13.65494500 | -3.91828800 | -0.02853600 |
| H | -12.52872400 | -4.32581800 | 1.77492600 |
| H | -14.61677800 | -3.19238700 | -1.82800000 |
| C | 12.34221000 | 1.41224500 | 0.00215200 |
| C | 13.28260100 | 1.73711100 | 1.01110700 |
| C | 12.08231700 | 2.38475500 | -1.00318500 |
| C | 13.92110300 | 2.98803700 | 1.02461200 |
| C | 12.74890300 | 3.61574500 | -0.98440000 |
| C | 13.65494500 | 3.91828800 | 0.02853600 |
| H | 14.61677800 | 3.19238700 | 1.82800000 |
| H | 12.52872400 | 4.32581800 | -1.77492600 |
| C | -13.90248900 | 0.57953400 | 1.99770700 |
| H | -14.32598300 | -0.33680800 | 1.57456300 |
| H | -13.03527200 | 0.28806400 | 2.59919600 |
| H | -14.64264000 | 1.00158100 | 2.68332900 |
| C | 13.90248900 | -0.57953400 | -1.99770700 |
| H | 13.03527200 | -0.28806400 | -2.59919600 |
| H | 14.32598300 | 0.33680800 | -1.57456300 |
| H | 14.64264000 | -1.00158100 | -2.68332900 |
| C | -11.08813600 | 2.21598500 | -1.99259500 |
| H | -10.10825900 | 2.37159200 | -1.52981400 |
| H | -11.05948100 | 1.22977300 | -2.46413600 |
| H | -11.20349100 | 2.95752000 | -2.78857200 |
| C | 11.08813600 | -2.21598500 | 1.99259500 |
| H | 11.05948100 | -1.22977300 | 2.46413600 |
| H | 10.10825900 | -2.37159200 | 1.52981400 |
| H | 11.20349100 | -2.95752000 | 2.78857200 |
| C | 13.62893100 | 0.78190000 | 2.13511200 |
| H | 12.73530200 | 0.41538500 | 2.65089800 |
| H | 14.17062800 | -0.09368200 | 1.76364800 |
| H | 14.25802800 | 1.26732100 | 2.88654400 |
| C | -13.62893100 | -0.78190000 | -2.13511200 |
| H | -14.17062800 | 0.09368200 | -1.76364800 |
| H | -12.73530200 | -0.41538500 | -2.65089800 |
| H | -14.25802800 | -1.26732100 | -2.88654400 |
| C | 11.09063500 | 2.16114700 | -2.12765000 |
| H | 11.20211900 | 1.17927400 | -2.59612000 |
| H | 10.05979700 | 2.22040500 | -1.76392000 |
| H | 11.21263600 | 2.91465000 | -2.91128100 |
| C | -11.09063500 | -2.16114700 | 2.12765000 |
| H | -10.05979700 | -2.22040500 | 1.76392000 |
| H | -11.20211900 | -1.17927400 | 2.59612000 |
| H | -11.21263600 | -2.91465000 | 2.91128100 |
| N | 14.32728800 | 5.27600300 | 0.01229100 |
| N | -14.32728800 | -5.27600300 | -0.01229100 |

| | | | |
|---|---|---|---|
| N | 14.78536500 | -5.01188700 | 0.18450800 |
| N | -14.78536500 | 5.01188700 | -0.18450800 |
| C | 13.84897800 | -6.19070300 | -0.00701400 |
| H | 14.43379600 | -7.11064100 | 0.04374700 |
| H | 13.09543400 | -6.18497100 | 0.77806500 |
| H | 13.37184200 | -6.09427200 | -0.98250300 |
| C | -13.84897800 | 6.19070300 | 0.00701400 |
| H | -13.09543400 | 6.18497100 | -0.77806500 |
| H | -14.43379600 | 7.11064100 | -0.04374700 |
| H | -13.37184200 | 6.09427200 | 0.98250300 |
| C | 13.27635300 | 6.36701100 | 0.10255600 |
| H | 13.77972200 | 7.33525900 | 0.10044800 |
| H | 12.60570700 | 6.29429700 | -0.75134800 |
| H | 12.71751200 | 6.22858200 | 1.02834700 |
| C | -13.27635300 | -6.36701100 | -0.10255600 |
| H | -12.60570700 | -6.29429700 | 0.75134800 |
| H | -13.77972200 | -7.33525900 | -0.10044800 |
| H | -12.71751200 | -6.22858200 | -1.02834700 |
| C | 15.28421100 | 5.47191200 | 1.16450300 |
| H | 14.73879400 | 5.39424300 | 2.10436700 |
| H | 16.07172200 | 4.72098500 | 1.11149500 |
| H | 15.71828000 | 6.46767400 | 1.07203600 |
| C | -15.28421100 | -5.47191200 | -1.16450300 |
| H | -16.07172200 | -4.72098500 | -1.11149500 |
| H | -14.73879400 | -5.39424300 | -2.10436700 |
| H | -15.71828000 | -6.46767400 | -1.07203600 |
| C | 15.11940500 | 5.44178900 | -1.27106000 |
| H | 15.86655800 | 4.64924700 | -1.31764500 |
| H | 14.44677800 | 5.37001900 | -2.12344200 |
| H | 15.60136100 | 6.42076100 | -1.25809200 |
| C | -15.11940500 | -5.44178900 | 1.27106000 |
| H | -14.44677800 | -5.37001900 | 2.12344200 |
| H | -15.86655800 | -4.64924700 | 1.31764500 |
| H | -15.60136100 | -6.42076100 | 1.25809200 |
| C | 15.46077100 | -5.11747600 | 1.53896400 |
| H | 14.70489500 | -5.11157500 | 2.32177200 |
| H | 16.02603400 | -6.05027000 | 1.57378900 |
| H | 16.12719400 | -4.26284500 | 1.65739700 |
| C | -15.46077100 | 5.11747600 | -1.53896400 |
| H | -16.02603400 | 6.05027000 | -1.57378900 |
| H | -14.70489500 | 5.11157500 | -2.32177200 |
| H | -16.12719400 | 4.26284500 | -1.65739700 |
| C | 15.86304100 | -5.11347000 | -0.86909700 |
| H | 15.40660500 | -5.07635100 | -1.85761200 |
| H | 16.57246100 | -4.29679800 | -0.74007200 |
| H | 16.37222000 | -6.06799200 | -0.73443400 |
| C | -15.86304100 | 5.11347000 | 0.86909700 |
| H | -16.57246100 | 4.29679800 | 0.74007200 |
| H | -15.40660500 | 5.07635100 | 1.85761200 |
| H | -16.37222000 | 6.06799200 | 0.73443400 |

DFT B3LYP/6-31+G(d, p), gas phase, $S_0$

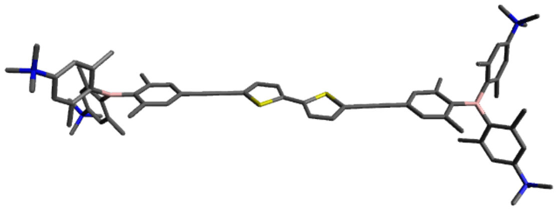

Point group: $C_1$

Total energy: –2,422,664.70 kcal mol$^{-1}$

Dipole moment: 0.13 D

Immaginary frequencies: 0

| | | | |
|---|---|---|---|
| C | 0.59369800 | 0.29359300 | -0.28246200 |
| C | 1.42704100 | -0.26962300 | -1.23979100 |
| C | 2.58860800 | 0.48529600 | -1.49743300 |
| C | 2.67128400 | 1.64712900 | -0.74091900 |
| S | 1.27222900 | 1.79428600 | 0.31506000 |
| C | -2.73418500 | -1.57131600 | 0.69078800 |
| C | 5.60719300 | 4.42197500 | -0.81598600 |
| C | 5.58936000 | 5.55311700 | 0.02323800 |
| C | 6.61394000 | 6.49779600 | 0.00122700 |
| C | 7.70535400 | 6.36806300 | -0.91759900 |
| C | 7.70799100 | 5.22555200 | -1.78183000 |
| C | 6.69072200 | 4.27652100 | -1.70466600 |
| B | 8.84974400 | 7.43507100 | -0.97858200 |
| C | 9.39414300 | 7.97184600 | -2.38913400 |
| C | 9.50395400 | 8.01571600 | 0.36620000 |
| C | 10.77090500 | 7.95043400 | -2.72522600 |
| C | 11.21441000 | 8.41559200 | -3.97565700 |
| C | 10.30625700 | 8.92991000 | -4.89261000 |
| C | 8.95054500 | 8.98028900 | -4.57721300 |
| C | 8.48291300 | 8.49790900 | -3.34779700 |
| C | 9.59490000 | 9.40666800 | 0.62250100 |
| C | 10.16444000 | 9.88331800 | 1.81624800 |
| C | 10.67224400 | 8.99212400 | 2.75328300 |
| C | 10.61163000 | 7.62112400 | 2.51530000 |
| C | 10.02436900 | 7.12280000 | 1.34497100 |
| C | 6.98751700 | 8.56676100 | -3.11023100 |
| C | 11.82790000 | 7.40677600 | -1.78540500 |
| C | 9.06595200 | 10.44665800 | -0.34430400 |
| C | 9.97634800 | 5.61561200 | 1.19182000 |
| C | 8.81508300 | 4.94441900 | -2.78234500 |
| C | 6.46913200 | 7.66957600 | 0.95598900 |
| C | 4.56952100 | 3.45780700 | -0.77259800 |
| C | 3.68436000 | 2.61522100 | -0.75555900 |
| N | 10.75115400 | 9.44128100 | -6.24758400 |
| N | 11.29505400 | 9.47288900 | 4.04805000 |
| C | 11.29626500 | 10.97865700 | 4.17761000 |
| C | 10.51853200 | 8.91550700 | 5.22810200 |
| C | 12.24338400 | 9.32816800 | -6.45905600 |
| C | 10.07414000 | 8.63943400 | -7.34533400 |
| C | -5.62558400 | -4.39060300 | 0.81942000 |
| C | -5.52430900 | -5.59544800 | 0.09675600 |
| C | -6.51545800 | -6.57317300 | 0.16023100 |
| C | -7.70101400 | -6.36032600 | 0.93588000 |
| C | -7.80804200 | -5.12363300 | 1.65120700 |
| C | -6.77936200 | -4.18503700 | 1.60132800 |
| B | -8.83524700 | -7.43791400 | 1.00111200 |
| C | -10.37768300 | -7.01187000 | 0.88352900 |
| C | -8.48082100 | -8.99102000 | 1.19038600 |
| C | -11.34220200 | -7.39222900 | 1.84990200 |
| C | -12.68294300 | -6.98707500 | 1.72729700 |
| C | -13.08699700 | -6.22712800 | 0.63676500 |

| | | | |
|---|---|---|---|
| C | -12.16170300 | -5.85898500 | -0.33707300 |
| C | -10.81535000 | -6.22884500 | -0.22166400 |
| C | -7.66025700 | -9.40307000 | 2.27226300 |
| C | -7.35394600 | -10.76297000 | 2.46021300 |
| C | -7.82959800 | -11.71986500 | 1.57325800 |
| C | -8.63236600 | -11.33784000 | 0.50034100 |
| C | -8.97171000 | -9.99268800 | 0.30903800 |
| C | -9.87879600 | -5.75360000 | -1.31446400 |
| C | -10.98806500 | -8.21870700 | 3.06958600 |
| C | -7.06713900 | -8.43170500 | 3.27391200 |
| C | -9.84747800 | -9.66490600 | -0.88267000 |
| C | -8.99315200 | -4.77495800 | 2.53418600 |
| C | -6.28065300 | -7.82333800 | -0.66902700 |
| C | -4.60128900 | -3.41287400 | 0.76430800 |
| C | -3.73057800 | -2.55590700 | 0.72920500 |
| N | -14.52387300 | -5.77489400 | 0.47469200 |
| N | -7.49322400 | -13.18804500 | 1.73705300 |
| C | -6.61787800 | -13.46106800 | 2.93876300 |
| C | -8.76842800 | -13.99303300 | 1.91090400 |
| C | -15.41899300 | -6.24288700 | 1.59896200 |
| C | -14.58351200 | -4.25760900 | 0.44746300 |
| C | -6.74797600 | -13.68281800 | 0.50991000 |
| C | 10.38077800 | 10.90654800 | -6.39231700 |
| C | 12.73809400 | 9.00787900 | 4.12958200 |
| C | -15.09435300 | -6.32484700 | -0.82038300 |
| C | -2.70243400 | -0.35972900 | 1.36905200 |
| C | -1.54792200 | 0.40241200 | 1.10093900 |
| C | -0.66944600 | -0.20469700 | 0.21332900 |
| S | -1.29073200 | -1.76151900 | -0.29633500 |
| H | 1.19909200 | -1.20037000 | -1.74667400 |
| H | 3.35078900 | 0.20383300 | -2.21379800 |
| H | 4.75310400 | 5.68521800 | 0.70243100 |
| H | 6.72551400 | 3.40030400 | -2.34425800 |
| H | 12.27331500 | 8.35761700 | -4.18933800 |
| H | 8.22352500 | 9.38179100 | -5.27501600 |
| H | 10.19079200 | 10.95368800 | 1.97087000 |
| H | 11.00537300 | 6.90647300 | 3.23009100 |
| H | 6.52952900 | 7.57581900 | -3.18505500 |
| H | 6.73869200 | 8.95301300 | -2.11892000 |
| H | 6.50532200 | 9.21526000 | -3.84620800 |
| H | 11.57112400 | 6.41136500 | -1.41194700 |
| H | 12.79558700 | 7.32368800 | -2.28610900 |
| H | 11.95836500 | 8.05617700 | -0.91461900 |
| H | 8.03324100 | 10.24209500 | -0.64099200 |
| H | 9.08171500 | 11.44399500 | 0.10189300 |
| H | 9.66528900 | 10.48167200 | -1.25892500 |
| H | 8.96228000 | 5.23493700 | 1.34708400 |
| H | 10.28596900 | 5.28580500 | 0.19722700 |
| H | 10.63194600 | 5.12873900 | 1.91835900 |
| H | 9.81399300 | 5.06164800 | -2.35231000 |
| H | 8.74159000 | 3.91795000 | -3.14935800 |
| H | 8.75615700 | 5.60677400 | -3.65198100 |
| H | 5.46573600 | 7.68405700 | 1.38804900 |
| H | 7.18096300 | 7.60972800 | 1.78543800 |
| H | 6.62254100 | 8.63562800 | 0.46610900 |
| H | 11.75974800 | 11.22978000 | 5.13124400 |
| H | 11.87534200 | 11.41249500 | 3.36377900 |
| H | 10.27099000 | 11.34523900 | 4.16128400 |
| H | 10.97365700 | 9.28478400 | 6.14790800 |
| H | 10.55670400 | 7.82880800 | 5.20532300 |
| H | 9.48574100 | 9.25401600 | 5.15006500 |
| H | 12.46867500 | 9.71676300 | -7.45179900 |
| H | 12.76244100 | 9.92117500 | -5.70752800 |
| H | 12.54030300 | 8.28215400 | -6.39951400 |
| H | 10.41904700 | 9.01298200 | -8.31024100 |
| H | 8.99575200 | 8.75633600 | -7.26593000 |
| H | 10.34558700 | 7.59136300 | -7.22207700 |
| H | -4.64806500 | -5.75989900 | -0.52231100 |
| H | -6.86230400 | -3.26797800 | 2.17591000 |
| H | -13.37392000 | -7.28672000 | 2.50388200 |
| H | -12.45455700 | -5.27019000 | -1.19975300 |
| H | -6.73321700 | -11.02542900 | 3.30644800 |
| H | -9.01239600 | -12.06601100 | -0.20818400 |
| H | -9.23749500 | -4.94124100 | -0.95940500 |
| H | -9.21449600 | -6.54525500 | -1.66929900 |
| H | -10.44053600 | -5.38495200 | -2.17659000 |
| H | -10.13237000 | -7.80496500 | 3.61065900 |
| H | -11.82369800 | -8.26244900 | 3.77230900 |
| H | -10.73115700 | -9.24583100 | 2.79381400 |
| H | -7.79011900 | -7.68759500 | 3.61589100 |
| H | -6.69655700 | -8.95954800 | 4.15638900 |
| H | -6.22936700 | -7.87838100 | 2.83836600 |
| H | -9.99900500 | -10.54352200 | -1.51445500 |
| H | -10.83191200 | -9.30692900 | -0.56752400 |
| H | -9.40595000 | -8.88661300 | -1.51204100 |
| H | -9.27566600 | -5.59105000 | 3.20546700 |
| H | -8.75722500 | -3.91103400 | 3.16002700 |
| H | -9.87898500 | -4.51837600 | 1.94442200 |
| H | -5.44273200 | -7.67154500 | -1.35355300 |
| H | -6.03822600 | -8.68787700 | -0.04283900 |
| H | -7.14778300 | -8.09628300 | -1.27751500 |
| H | -6.42973400 | -14.53370800 | 2.97544800 |
| H | -5.67501200 | -12.92730200 | 2.82971300 |
| H | -7.13639100 | -13.14912900 | 3.84413000 |
| H | -8.50147500 | -15.04271900 | 2.03914800 |
| H | -9.39377800 | -13.87495400 | 1.02893700 |
| H | -9.29134500 | -13.62212500 | 2.79200700 |
| H | -16.42444200 | -5.87368700 | 1.39884700 |
| H | -15.42766500 | -7.33147400 | 1.62684500 |
| H | -15.06029900 | -5.83441800 | 2.54252900 |
| H | -15.62593600 | -3.95361700 | 0.34499200 |
| H | -14.00440300 | -3.88801700 | -0.39571200 |
| H | -14.16557700 | -3.88083300 | 1.38057500 |
| H | -5.84071100 | -13.09000300 | 0.39698300 |
| H | -6.50169100 | -14.73535100 | 0.65498400 |
| H | -7.37943800 | -13.56721800 | -0.36802100 |
| H | 10.87133600 | 11.46523100 | -5.59572100 |
| H | 10.72041400 | 11.25526400 | -7.36831100 |
| H | 9.30161300 | 11.01670800 | -6.31428800 |
| H | 13.27840800 | 9.41277100 | 3.27433400 |
| H | 13.16862500 | 9.37524400 | 5.06189300 |
| H | 12.76994600 | 7.92095000 | 4.10953200 |
| H | -15.03921000 | -7.41254200 | -0.78557800 |
| H | -16.13100200 | -5.99775600 | -0.90911500 |
| H | -14.51409500 | -5.94923500 | -1.66006500 |
| H | -3.49816000 | -0.04560700 | 2.03348400 |
| H | -1.36416200 | 1.37646000 | 1.53985400 |

Compound **4**

DFT B3LYP/6-31G(d), gas phase, $S_0$

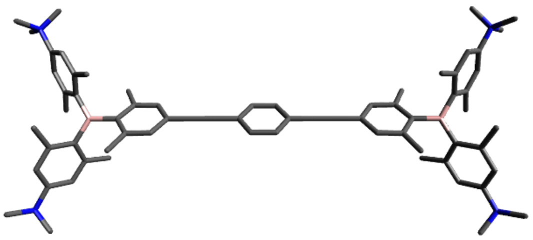

Point group: $C_i$
Total energy: –1,874,995.57 kcal mol$^{-1}$
Dipole moment: 0 D
Immaginary frequencies: 0

| | | | |
|---|---|---|---|
| C | -0.89702814 | -0.81899906 | -0.69335247 |
| C | -0.89702028 | -0.81901498 | 0.69328653 |
| C | 0.00022722 | 0.00002189 | -1.41254795 |
| C | -0.00022722 | -0.00002189 | 1.41254795 |
| C | 0.89702814 | 0.81899906 | 0.69335247 |
| C | 0.89702028 | 0.81901498 | -0.69328653 |

| | | | |
|---|---|---|---|
| C | 0.00020851 | 0.00005284 | -2.83492095 |
| C | 0.00019490 | 0.00009166 | -4.05329195 |
| C | 0.00014518 | 0.00007460 | -5.47365495 |
| C | 0.86595091 | 0.83864249 | -6.19830656 |
| C | -0.86576614 | -0.83852731 | -6.19818934 |
| C | 0.86484014 | 0.86603538 | -7.59134455 |
| H | 1.54920771 | 1.47998032 | -5.65008012 |
| C | -0.86481305 | -0.86597935 | -7.59120536 |
| H | -1.54897379 | -1.47981088 | -5.64983879 |
| C | 0.00000082 | 0.00000051 | -8.33297995 |
| C | 1.84952974 | 1.81731426 | -8.24745056 |
| C | -1.84965485 | -1.81715835 | -8.24721335 |
| B | -0.00008125 | -0.00001344 | -9.90036395 |
| H | 2.56780293 | 2.18689716 | -7.51043014 |
| H | 2.42618230 | 1.34566038 | -9.04968533 |
| H | 1.34824300 | 2.69038353 | -8.67942619 |
| H | -1.34847730 | -2.69003005 | -8.67969271 |
| H | -2.56760255 | -2.18704323 | -7.51002777 |
| H | -2.42668333 | -1.34526341 | -9.04904958 |
| C | 0.01463460 | 1.37776860 | -10.72057597 |
| C | -0.01479784 | -1.37780140 | -10.72057794 |
| C | 0.97355004 | 1.64343984 | -11.72954655 |
| C | -0.96225350 | 2.37628125 | -10.45104962 |
| C | -0.97357735 | -1.64343228 | -11.72970636 |
| C | 0.96205388 | -2.37633341 | -10.45098229 |
| C | 0.96618870 | 2.86307906 | -12.42613677 |
| C | 2.06362517 | 0.65645210 | -12.09557639 |
| C | -0.96625197 | 3.57414260 | -11.17574184 |
| C | -2.03273872 | 2.21507019 | -9.38990746 |
| C | -0.96611386 | -2.86303256 | -12.42635614 |
| C | -2.06362625 | -0.65646005 | -12.09584952 |
| C | 0.96614676 | -3.57416378 | -11.17573807 |
| C | 2.03243872 | -2.21522777 | -9.38971245 |
| C | -0.00254398 | 3.81896798 | -12.15043641 |
| H | 1.73183758 | 3.02306256 | -13.17397228 |
| H | 2.60544449 | 0.29680591 | -11.21503624 |
| H | 1.65325923 | -0.22154250 | -12.60470079 |
| H | 2.80041965 | 1.11209556 | -12.76305583 |
| H | -1.73462681 | 4.30523936 | -10.94643975 |
| H | -2.52508439 | 1.23944751 | -9.43569529 |
| H | -1.61069332 | 2.30789085 | -8.38413053 |
| H | -2.80847417 | 2.97857293 | -9.49885038 |
| C | 0.00258238 | -3.81893985 | -12.15057650 |
| H | -1.73164812 | -3.02297206 | -13.17431563 |
| H | -2.60604633 | -0.29743605 | -11.21542368 |
| H | -1.65314883 | 0.22191023 | -12.60422912 |
| H | -2.79992183 | -1.11189060 | -12.76402308 |
| H | 1.73449357 | -4.30526785 | -10.94636716 |
| H | 2.52476579 | -1.23959702 | -9.43530362 |
| H | 1.61029243 | -2.30818478 | -8.38398638 |
| H | 2.80819212 | -2.97870536 | -9.49869353 |
| C | -0.00020851 | -0.00005284 | 2.83492095 |
| C | -0.00019490 | -0.00009166 | 4.05329195 |
| C | -0.00014518 | -0.00007460 | 5.47365495 |
| C | 0.86576614 | 0.83852731 | 6.19818934 |
| C | -0.86595091 | -0.83864249 | 6.19830656 |
| C | 0.86481305 | 0.86597935 | 7.59120536 |
| H | 1.54897379 | 1.47981088 | 5.64983879 |
| C | -0.86484014 | -0.86603538 | 7.59134455 |
| H | -1.54920771 | -1.47998032 | 5.65008012 |
| C | -0.00000082 | -0.00000051 | 8.33297995 |
| C | 1.84965485 | 1.81715835 | 8.24721335 |
| C | -1.84952974 | -1.81731426 | 8.24745056 |
| B | 0.00008125 | 0.00001344 | 9.90036395 |
| H | 2.56760255 | 2.18704323 | 7.51002777 |
| H | 1.34847730 | 2.69003005 | 8.67969271 |
| H | 2.42668333 | 1.34526341 | 9.04904958 |
| H | -2.42618230 | -1.34566038 | 9.04968533 |
| H | -2.56780293 | -2.18689716 | 7.51043014 |
| H | -1.34824300 | -2.69038353 | 8.67942619 |
| C | -0.01463460 | -1.37776860 | 10.72057597 |
| C | 0.01479784 | 1.37780140 | 10.72057794 |
| C | 0.96225350 | -2.37628125 | 10.45104962 |
| C | -0.97355004 | -1.64343984 | 11.72954655 |
| C | -0.96205388 | 2.37633341 | 10.45098229 |
| C | 0.97357735 | 1.64343228 | 11.72970636 |
| C | 0.96625197 | -3.57414260 | 11.17574184 |
| C | 2.03273872 | -2.21507019 | 9.38990746 |
| C | -0.96618870 | -2.86307906 | 12.42613677 |
| C | -2.06362517 | -0.65645210 | 12.09557639 |
| C | -0.96614676 | 3.57416378 | 11.17573807 |
| C | -2.03243872 | 2.21522777 | 9.38971245 |
| C | 0.96611386 | 2.86303256 | 12.42635614 |
| C | 2.06362625 | 0.65646005 | 12.09584952 |
| C | 0.00254398 | -3.81896798 | 12.15043641 |
| H | 1.73462681 | -4.30523936 | 10.94643975 |
| H | 1.61069332 | -2.30789085 | 8.38413053 |
| H | 2.52508439 | -1.23944751 | 9.43569529 |
| H | 2.80847417 | -2.97857293 | 9.49885038 |
| H | -1.73183758 | -3.02306256 | 13.17397228 |
| H | -1.65325923 | 0.22154250 | 12.60470079 |
| H | -2.60544449 | -0.29680591 | 11.21503624 |
| H | -2.80041965 | -1.11209556 | 12.76305583 |
| C | -0.00258238 | 3.81893985 | 12.15057650 |
| H | -1.73449357 | 4.30526785 | 10.94636716 |
| H | -1.61029243 | 2.30818478 | 8.38398638 |
| H | -2.52476579 | 1.23959702 | 9.43530362 |
| H | -2.80819212 | 2.97870536 | 9.49869353 |
| H | 1.73164812 | 3.02297206 | 13.17431563 |
| H | 1.65314883 | -0.22191023 | 12.60422912 |
| H | 2.60604633 | 0.29743605 | 11.21542368 |
| H | 2.79992183 | 1.11189060 | 12.76402308 |
| H | 1.59158615 | 1.45179781 | -1.23681295 |
| H | 1.59160022 | 1.45176916 | 1.23677005 |
| H | -1.59160022 | -1.45176916 | -1.23677005 |
| H | -1.59158615 | -1.45179781 | 1.23681295 |
| N | 0.04103859 | -5.14287906 | -12.88617721 |
| N | -0.04103859 | 5.14287906 | 12.88617721 |
| N | 0.04150064 | -5.14283027 | 12.88614479 |
| C | -0.12021931 | -6.28192010 | 11.89638831 |
| H | -0.10046673 | -7.22459579 | 12.44575677 |
| H | -1.07461325 | -6.15799816 | 11.38419918 |
| H | 0.69532692 | -6.25372892 | 11.17644294 |
| C | 0.12030307 | 6.28187808 | 11.89655278 |
| H | -0.69487679 | 6.25378988 | 11.17618815 |
| H | 0.10039363 | 7.22455304 | 12.44591732 |
| H | 1.07494255 | 6.15783584 | 11.38485092 |
| C | -0.12030307 | -6.28187808 | -11.89655278 |
| H | -1.07494255 | -6.15783584 | -11.38485092 |
| H | -0.10039363 | -7.22455304 | -12.44591732 |
| H | 0.69487679 | -6.25378988 | -11.17618815 |
| N | -0.04150064 | 5.14283027 | -12.88614479 |
| C | 0.12021931 | 6.28192010 | -11.89638831 |
| H | 1.07461325 | 6.15799816 | -11.38419918 |
| H | 0.10046673 | 7.22459579 | -12.44575677 |
| H | -0.69532692 | 6.25372892 | -11.17644294 |
| C | -1.36505042 | 5.28871269 | -13.61273345 |
| H | -1.36759548 | 6.24299296 | -14.14201283 |
| H | -1.46290823 | 4.46195684 | -14.31660244 |
| H | -2.17909971 | 5.26205557 | -12.89106807 |
| C | 1.05784432 | 5.27669617 | -13.91430775 |
| H | 2.02612132 | 5.21234071 | -13.41913951 |
| H | 0.95207933 | 4.49004707 | -14.66058174 |
| H | 0.95217132 | 6.25178390 | -14.39025756 |
| C | -1.05735233 | -5.27646867 | -13.91489916 |
| H | -0.95156711 | -6.25151476 | -14.39090834 |
| H | -2.02580770 | -5.21211588 | -13.42008240 |
| H | -0.95127274 | -4.48974456 | -14.66105016 |
| C | 1.36541676 | -5.28882560 | -13.61244545 |
| H | 1.46365879 | -4.46204879 | -14.31623647 |
| H | 2.17921467 | -5.26234345 | -12.89049184 |
| H | 1.36799850 | -6.24307816 | -14.14177208 |
| C | -1.05784432 | -5.27669617 | 13.91430775 |
| H | -2.02612132 | -5.21234071 | 13.41913951 |
| H | -0.95217132 | -6.25178390 | 14.39025756 |
| H | -0.95207933 | -4.49004707 | 14.66058174 |
| C | 1.36505042 | -5.28871269 | 13.61273345 |
| H | 2.17909971 | -5.26205557 | 12.89106807 |
| H | 1.46290823 | -4.46195684 | 14.31660244 |
| H | 1.36759548 | -6.24299296 | 14.14201283 |
| C | 1.05735233 | 5.27646867 | 13.91489916 |
| H | 0.95127274 | 4.48974456 | 14.66105016 |
| H | 2.02580770 | 5.21211588 | 13.42008240 |
| H | 0.95156711 | 6.25151476 | 14.39090834 |

| | | | |
|---|---|---|---|
| C | -1.36541676 | 5.28882560 | 13.61244545 |
| H | -2.17921467 | 5.26234345 | 12.89049184 |
| H | -1.46365879 | 4.46204879 | 14.31623647 |
| H | -1.36799850 | 6.24307816 | 14.14177208 |

## DFT B3LYP/6-31G(d), gas phase, $S_0$

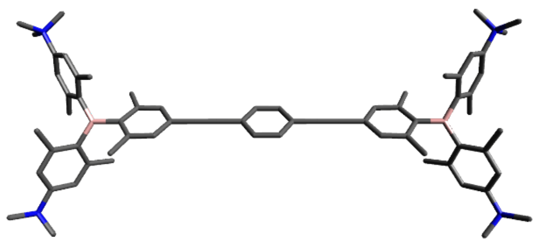

Point group: $C_1$
Total energy: –1,875,107.16 kcal mol$^{-1}$
Dipole moment: 0 D
Immaginary frequencies: 0

| | | | |
|---|---|---|---|
| C | -0.69412900 | -0.80247600 | 0.91221000 |
| C | 0.69410400 | -0.80247600 | 0.91221400 |
| C | -1.41418200 | 0.00001900 | -0.00030500 |
| C | 1.41416200 | 0.00002000 | -0.00029600 |
| C | 0.69410800 | 0.80251400 | -0.91281300 |
| C | -0.69412400 | 0.80251400 | -0.91281700 |
| C | -2.83811300 | 0.00002100 | -0.00030500 |
| C | -4.05764500 | 0.00001800 | -0.00029400 |
| C | -5.47987200 | -0.00000100 | -0.00024100 |
| C | -6.20476600 | 0.82437200 | -0.88066500 |
| C | -6.20467900 | -0.82438700 | 0.88024300 |
| C | -7.59934400 | 0.85193300 | -0.87880200 |
| H | -5.65761600 | 1.45423000 | -1.57494300 |
| C | -7.59925500 | -0.85197700 | 0.87849600 |
| H | -5.65745900 | -1.45422800 | 1.57448200 |
| C | -8.34072300 | -0.00003100 | -0.00012500 |
| C | -8.25814400 | 1.78593000 | -1.87821500 |
| C | -8.25795100 | -1.78597600 | 1.87797500 |
| B | -9.91040200 | -0.00001700 | -0.00005000 |
| H | -7.51971000 | 2.15985700 | -2.59136200 |
| H | -9.04625300 | 1.29717500 | -2.45862300 |
| H | -8.70784600 | 2.65507900 | -1.38762200 |
| H | -8.70765900 | -2.65515100 | 1.38743300 |
| H | -7.51945200 | -2.15986300 | 2.59107600 |
| H | -9.04603400 | -1.29723600 | 2.45843100 |
| C | -10.72901700 | 1.37878100 | -0.01366300 |
| C | -10.72905500 | -1.37879300 | 0.01368900 |
| C | -11.73812300 | 1.64513900 | -0.97270600 |
| C | -10.45726600 | 2.37817900 | 0.96277200 |
| C | -11.73804600 | -1.64510400 | 0.97286600 |
| C | -10.45748300 | -2.37820700 | -0.96278200 |
| C | -12.43270600 | 2.86753300 | -0.96601100 |
| C | -12.10651600 | 0.65800100 | -2.06193900 |
| C | -11.18097200 | 3.57781300 | 0.96641100 |
| C | -9.39707400 | 2.21494200 | 2.03365900 |
| C | -12.43267300 | -2.86747400 | 0.96627900 |
| C | -12.10627600 | -0.65793400 | 2.06212600 |
| C | -11.18123400 | -3.57781300 | -0.96631100 |
| C | -9.39744900 | -2.21501700 | -2.03383400 |
| C | -12.15593400 | 3.82416500 | 0.00257900 |
| H | -13.17931400 | 3.02804500 | -1.73220400 |
| H | -11.22682000 | 0.28667800 | -2.59484300 |
| H | -12.62795500 | -0.21174400 | -1.65088000 |
| H | -12.76299100 | 1.11795800 | -2.80454700 |
| H | -10.95042200 | 4.30789200 | 1.73479000 |
| H | -9.44471200 | 1.23959600 | 2.52430400 |
| H | -8.39164700 | 2.30620400 | 1.61188000 |
| H | -9.50556600 | 2.97725300 | 2.80931600 |
| C | -12.15606600 | -3.82412600 | -0.00233700 |
| H | -13.17918700 | -3.02794800 | 1.73257100 |
| H | -11.22650500 | -0.28662500 | 2.59491500 |
| H | -12.62774100 | 0.21181700 | 1.65111300 |
| H | -12.76267300 | -1.11785800 | 2.80482300 |
| H | -10.95082600 | -4.30790300 | -1.73472300 |
| H | -9.44512600 | -1.23967200 | -2.52447600 |
| H | -8.39195900 | -2.30631500 | -1.61221100 |
| H | -9.50609100 | -2.97732800 | -2.80946900 |
| C | 2.83809200 | 0.00002200 | -0.00028900 |
| C | 4.05762500 | 0.00002100 | -0.00026700 |
| C | 5.47985200 | 0.00001700 | -0.00019300 |
| C | 6.20475100 | 0.82440100 | -0.88060200 |
| C | 6.20465200 | -0.82437400 | 0.88029100 |
| C | 7.59932800 | 0.85197400 | -0.87872100 |
| H | 5.65760500 | 1.45425800 | -1.57488600 |
| C | 7.59922900 | -0.85195500 | 0.87856000 |
| H | 5.65742700 | -1.45423000 | 1.57451200 |
| C | 8.34070300 | 0.00001000 | -0.00003700 |
| C | 8.25813300 | 1.78597200 | -1.87813000 |
| C | 8.25791800 | -1.78596800 | 1.87803100 |
| B | 9.91038000 | -0.00000100 | 0.00004700 |
| H | 7.51970700 | 2.15987800 | -2.59129600 |
| H | 8.70780900 | 2.65513500 | -1.38753700 |
| H | 9.04626300 | 1.29722500 | -2.45851700 |
| H | 9.04597000 | -1.29722500 | 2.45852700 |
| H | 7.51940700 | -2.15989500 | 2.59109800 |
| H | 8.70766400 | -2.65511600 | 1.38747700 |
| C | 10.72901000 | -1.37879100 | 0.01365700 |
| C | 10.72903100 | 1.37877500 | -0.01353400 |
| C | 10.45739700 | -2.37811900 | -0.96288900 |
| C | 11.73800800 | -1.64520200 | 0.97279800 |
| C | 10.45737900 | 2.37812300 | 0.96298100 |
| C | 11.73810000 | 1.64514700 | -0.97261000 |
| C | 11.18112600 | -3.57773800 | -0.96653300 |
| C | 9.39733700 | -2.21481700 | -2.03389800 |
| C | 12.43261700 | -2.86758100 | 0.96609300 |
| C | 12.10625500 | -0.65813500 | 2.06214600 |
| C | 11.18113900 | 3.57772400 | 0.96665700 |
| C | 9.39724800 | 2.21486300 | 2.03392600 |
| C | 12.43273400 | 2.86751300 | -0.96588100 |
| C | 12.10640600 | 0.65805200 | -2.06191200 |
| C | 12.15597600 | -3.82414500 | -0.00260200 |
| H | 10.95068500 | -4.30776100 | -1.73499800 |
| H | 8.39185900 | -2.30612600 | -1.61225200 |
| H | 9.44502300 | -1.23943200 | -2.52446300 |
| H | 9.50593900 | -2.97706700 | -2.80959900 |
| H | 13.17913700 | -3.02813600 | 1.73236200 |
| H | 12.62775600 | 0.21163300 | 1.65121200 |
| H | 11.22648800 | -0.28684000 | 2.59495000 |
| H | 12.76262500 | -1.11814100 | 2.80481400 |
| C | 12.15605600 | 3.82409600 | 0.00278400 |
| H | 10.95066500 | 4.30776300 | 1.73509600 |
| H | 8.39179900 | 2.30619300 | 1.61221500 |
| H | 9.44487500 | 1.23948300 | 2.52450500 |
| H | 9.50582200 | 2.97712100 | 2.80962300 |
| H | 13.17931000 | 3.02803800 | -1.73210200 |
| H | 12.62785100 | -0.21172300 | -1.65092500 |
| H | 11.22666900 | 0.28677300 | -2.59477900 |
| H | 12.76284500 | 1.11803100 | -2.80453900 |
| H | -1.23601400 | 1.42263300 | -1.61898200 |
| H | 1.23600200 | 1.42263300 | -1.61897500 |
| H | -1.23602200 | -1.42259400 | 1.61837400 |
| H | 1.23599300 | -1.42259400 | 1.61838000 |
| N | -12.89014900 | -5.14885300 | -0.04001900 |
| N | 12.89013800 | 5.14881900 | 0.04061300 |
| N | 12.89003400 | -5.14888300 | -0.04040600 |
| C | 11.89951300 | -6.28864300 | 0.12192900 |
| H | 12.44952300 | -7.23031600 | 0.10614700 |
| H | 11.38450000 | -6.16300100 | 1.07394100 |
| H | 11.18253500 | -6.26390800 | -0.69553300 |
| C | 11.89962900 | 6.28859900 | -0.12166000 |
| H | 11.18265900 | 6.26383300 | 0.69581000 |
| H | 12.44965000 | 7.23026500 | -0.10584000 |

| | | | |
|---|---|---|---|
| H | 11.38460400 | 6.16300700 | -1.07367200 |
| C | -11.89965000 | -6.28861800 | 0.12241900 |
| H | -11.38462800 | -6.16289600 | 1.07441500 |
| H | -12.44968100 | -7.23028100 | 0.10673200 |
| H | -11.18267800 | -6.26397700 | -0.69505100 |
| N | -12.88995700 | 5.14892200 | 0.04036700 |
| C | -11.89942100 | 6.28864800 | -0.12211600 |
| H | -11.38449300 | 6.16294100 | -1.07416500 |
| H | -12.44940500 | 7.23033600 | -0.10633600 |
| H | -11.18237300 | 6.26393600 | 0.69528600 |
| C | -13.61657900 | 5.29787300 | 1.36544400 |
| H | -14.14694800 | 6.25085300 | 1.36452900 |
| H | -14.31877300 | 4.47090300 | 1.46728500 |
| H | -12.89492700 | 5.27625000 | 2.17883200 |
| C | -13.91941500 | 5.28386100 | -1.05826600 |
| H | -13.42678700 | 5.22025000 | -2.02735200 |
| H | -14.66833500 | 4.50040400 | -0.95235600 |
| H | -14.39289000 | 6.25944700 | -0.95179900 |
| C | -13.91952100 | -5.28371200 | 1.05870400 |
| H | -14.39302100 | -6.25929600 | 0.95233000 |
| H | -13.42681300 | -5.22005400 | 2.02774700 |
| H | -14.66843600 | -4.50024900 | 0.95281000 |
| C | -13.61688500 | -5.29781400 | -1.36503100 |
| H | -14.31906100 | -4.47082400 | -1.46683400 |
| H | -12.89529900 | -5.27623200 | -2.17847900 |
| H | -14.14728400 | -6.25077800 | -1.36405100 |
| C | 13.91940600 | -5.28386200 | 1.05830200 |
| H | 13.42669900 | -5.22030100 | 2.02735200 |
| H | 14.39289900 | -6.25943800 | 0.95182700 |
| H | 14.66832600 | -4.50039300 | 0.95248900 |
| C | 13.61676600 | -5.29773800 | -1.36543300 |
| H | 12.89517900 | -5.27607600 | -2.17887800 |
| H | 14.31895300 | -4.47074900 | -1.46716600 |
| H | 14.14715200 | -6.25070900 | -1.36453700 |
| C | 13.91948300 | 5.28381700 | -1.05811800 |
| H | 14.66835100 | 4.50028600 | -0.95239300 |
| H | 13.42674000 | 5.22037100 | -2.02715700 |
| H | 14.39304800 | 6.25935200 | -0.95157800 |
| C | 13.61690800 | 5.29762300 | 1.36562500 |
| H | 12.89534300 | 5.27596800 | 2.17908900 |
| H | 14.31907100 | 4.47060800 | 1.46732100 |
| H | 14.14732600 | 6.25057700 | 1.36473300 |

## Compound **5**

DFT B3LYP/6-31G(d), gas phase, $S_0$

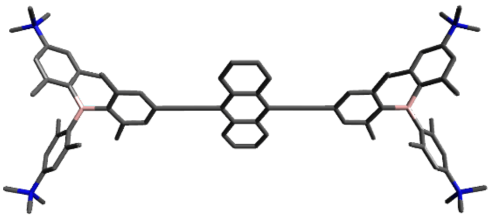

Point group: $C_i$

Total energy: –2067818.29 kcal mol$^{-1}$

Dipole moment: 0 D

Immaginary frequencies: 0

| | | | |
|---|---|---|---|
| C | 2.54661103 | -0.70979169 | 2.64895344 |
| C | 1.72577458 | -1.40014405 | 1.79413486 |
| C | 0.85870694 | -0.71917273 | 0.89081595 |
| C | 0.85873649 | 0.71909539 | 0.89085403 |
| C | 1.72583153 | 1.39998218 | 1.79421074 |
| C | 2.54663955 | 0.70955043 | 2.64899137 |
| C | -0.00002950 | -1.42705512 | -0.00003466 |
| C | 0.00002950 | 1.42705512 | 0.00003466 |
| C | -0.85870694 | 0.71917273 | -0.89081595 |
| C | -0.85873649 | -0.71909539 | -0.89085403 |
| C | -1.72583153 | -1.39998218 | -1.79421074 |
| H | -1.72247102 | -2.48444155 | -1.79328899 |
| C | -2.54663955 | -0.70955043 | -2.64899137 |
| C | -2.54661103 | 0.70979169 | -2.64895344 |
| C | -1.72577458 | 1.40014405 | -1.79413486 |
| H | 3.19906501 | -1.24777889 | 3.33040749 |
| H | 1.72237030 | -2.48460386 | 1.79315335 |
| H | 1.72247102 | 2.48444155 | 1.79328899 |
| H | 3.19911506 | 1.24747431 | 3.33047503 |
| H | -3.19911506 | -1.24747431 | -3.33047503 |
| H | -3.19906501 | 1.24777889 | -3.33040749 |
| H | -1.72237030 | 2.48460386 | -1.79315335 |
| C | -0.00005534 | -2.84413324 | -0.00006202 |
| C | -0.00001335 | -4.06529836 | -0.00009307 |
| C | -0.00000481 | -5.48331150 | -0.00010139 |
| C | -0.90300772 | -6.21046555 | -0.79850033 |
| C | 0.90300355 | -6.21046559 | 0.79827241 |
| C | -0.93080983 | -7.60268606 | -0.79468443 |
| H | -1.59601661 | -5.66380743 | -1.43074487 |
| C | 0.93082898 | -7.60269635 | 0.79442638 |
| H | 1.59601091 | -5.66382060 | 1.43053069 |
| C | 0.00001444 | -8.34615978 | -0.00012913 |
| C | -1.95569129 | -8.25749606 | -1.70350370 |
| C | 1.95573487 | -8.25748247 | 1.70323623 |
| B | 0.00000388 | -9.91197294 | -0.00010128 |
| H | -2.37784342 | -7.52012508 | -2.39187095 |
| H | -1.53031155 | -9.06057691 | -2.31391314 |
| H | -2.78861714 | -8.68816142 | -1.13710789 |
| H | 2.78862778 | -8.68820319 | 1.13683913 |
| H | 2.37792948 | -7.52008030 | 2.39154307 |
| H | 1.53036940 | -9.06052081 | 2.31371670 |
| C | -1.37587144 | -10.73259901 | 0.07904981 |
| C | 1.37586332 | -10.73264905 | -0.07915741 |
| C | -1.71000057 | -11.74237736 | -0.86341783 |
| C | -2.29974058 | -10.46739369 | 1.12253862 |
| C | 1.70990935 | -11.74236690 | 0.86339865 |
| C | 2.29978748 | -10.46758033 | -1.12263905 |
| C | -2.92308203 | -12.43312499 | -0.76590283 |
| C | -0.80305702 | -12.10303799 | -2.02190857 |
| C | -3.50075053 | -11.19113528 | 1.21569118 |
| C | -2.06523435 | -9.40805445 | 2.18179024 |
| C | 2.92296533 | -12.43318144 | 0.76598368 |
| C | 0.80290186 | -12.10295531 | 2.02186440 |
| C | 3.50076374 | -11.19137891 | -1.21568748 |
| C | 2.06533173 | -9.40834534 | -2.18200243 |
| C | -3.81173896 | -12.15979849 | 0.27108941 |
| H | -3.14633266 | -13.18297530 | -1.51780947 |
| H | -0.50534749 | -11.22157553 | -2.59915080 |
| H | 0.11494101 | -12.58798159 | -1.67534173 |
| H | -1.30010662 | -12.78877862 | -2.71382088 |
| H | -4.16658328 | -10.95755606 | 2.03621466 |
| H | -1.04836864 | -9.43238157 | 2.58283607 |
| H | -2.21971532 | -8.40258640 | 1.77663634 |
| H | -2.75280288 | -9.53804195 | 3.02265069 |
| C | 3.81166935 | -12.15998589 | -0.27099737 |
| H | 3.14615206 | -13.18297528 | 1.51796675 |
| H | 0.50466784 | -11.22141531 | 2.59869824 |
| H | -0.11480683 | -12.58846490 | 1.67530353 |
| H | 1.30012709 | -12.78821198 | 2.71413129 |
| H | 4.16664687 | -10.95788911 | -2.03619524 |
| H | 1.04857698 | -9.43295317 | -2.58331438 |
| H | 2.21946537 | -8.40282109 | -1.77686633 |
| H | 2.75314536 | -9.53822700 | -3.02267786 |
| C | 0.00005534 | 2.84413324 | 0.00006202 |
| C | 0.00001335 | 4.06529836 | 0.00009307 |
| C | 0.00000481 | 5.48331150 | 0.00010139 |
| C | -0.90300355 | 6.21046559 | -0.79827241 |

| | | | |
|---|---|---|---|
| C | 0.90300772 | 6.21046555 | 0.79850033 |
| C | -0.93082898 | 7.60269635 | -0.79442638 |
| H | -1.59601091 | 5.66382060 | -1.43053069 |
| C | 0.93080983 | 7.60268606 | 0.79468443 |
| H | 1.59601661 | 5.66380743 | 1.43074487 |
| C | -0.00001444 | 8.34615978 | 0.00012913 |
| C | -1.95573487 | 8.25748247 | -1.70323623 |
| C | 1.95569129 | 8.25749606 | 1.70350370 |
| B | -0.00000388 | 9.91197294 | 0.00010128 |
| H | -2.37792948 | 7.52008030 | -2.39154307 |
| H | -2.78862778 | 8.68820319 | -1.13683913 |
| H | -1.53036940 | 9.06052081 | -2.31371670 |
| H | 1.53031155 | 9.06057691 | 2.31391314 |
| H | 2.37784342 | 7.52012508 | 2.39187095 |
| H | 2.78861714 | 8.68816142 | 1.13710789 |
| C | 1.37587144 | 10.73259901 | -0.07904981 |
| C | -1.37586332 | 10.73264905 | 0.07915741 |
| C | 2.29974058 | 10.46739369 | -1.12253862 |
| C | 1.71000057 | 11.74237736 | 0.86341783 |
| C | -2.29978748 | 10.46758033 | 1.12263905 |
| C | -1.70990935 | 11.74236690 | -0.86339865 |
| C | 3.50075053 | 11.19113528 | -1.21569118 |
| C | 2.06523435 | 9.40805445 | -2.18179024 |
| C | 2.92308203 | 12.43312499 | 0.76590283 |
| C | 0.80305702 | 12.10303799 | 2.02190857 |
| C | -3.50076374 | 11.19137891 | 1.21568748 |
| C | -2.06533173 | 9.40834534 | 2.18200243 |
| C | -2.92296533 | 12.43318144 | -0.76598368 |
| C | -0.80290186 | 12.10295531 | -2.02186440 |
| C | 3.81173896 | 12.15979849 | -0.27108941 |
| H | 4.16658328 | 10.95755606 | -2.03621466 |
| H | 2.21971532 | 8.40258640 | -1.77663634 |
| H | 1.04836864 | 9.43238157 | -2.58283607 |
| H | 2.75280288 | 9.53804195 | -3.02265069 |
| H | 3.14633266 | 13.18297530 | 1.51780947 |
| H | -0.11494101 | 12.58798159 | 1.67534173 |
| H | 0.50534749 | 11.22157553 | 2.59915080 |
| H | 1.30010662 | 12.78877862 | 2.71382088 |
| C | -3.81166935 | 12.15998589 | 0.27099737 |
| H | -4.16664687 | 10.95788911 | 2.03619524 |
| H | -2.21946537 | 8.40282109 | 1.77686633 |
| H | -1.04857698 | 9.43295317 | 2.58331438 |
| H | -2.75314536 | 9.53822700 | 3.02267786 |
| H | -3.14615206 | 13.18297528 | -1.51796675 |
| H | 0.11480683 | 12.58846490 | -1.67530353 |
| H | -0.50466784 | 11.22141531 | -2.59869824 |
| H | -1.30012709 | 12.78821198 | -2.71413129 |
| N | 5.11030821 | 12.93784668 | -0.33278125 |
| N | -5.11030821 | -12.93784668 | 0.33278125 |
| N | -5.11020789 | 12.93809389 | 0.33259616 |
| N | 5.11020789 | -12.93809389 | -0.33259616 |
| C | 5.91468569 | -12.68975081 | 0.93015368 |
| H | 5.34419055 | -13.02467651 | 1.79420885 |
| H | 6.84987394 | -13.24794599 | 0.86165004 |
| H | 6.11243894 | -11.62031757 | 1.00575797 |
| C | -5.91468569 | 12.68975081 | -0.93015368 |
| H | -6.84987394 | 13.24794599 | -0.86165004 |
| H | -5.34419055 | 13.02467651 | -1.79420885 |
| H | -6.11243894 | 11.62031757 | -1.00575797 |
| C | 5.91478576 | 12.68958270 | 0.92998385 |
| H | 6.11248389 | 11.62014671 | 1.00568871 |
| H | 6.85000256 | 13.24772364 | 0.86141821 |
| H | 5.34431659 | 13.02462307 | 1.79401272 |
| C | -5.91478576 | -12.68958270 | -0.92998385 |
| H | -5.34431659 | -13.02462307 | -1.79401272 |
| H | -6.85000256 | -13.24772364 | -0.86141821 |
| H | -6.11248389 | -11.62014671 | -1.00568871 |
| C | 5.97616832 | -12.54478454 | -1.50656480 |
| H | 6.23623104 | -11.48992365 | -1.42622615 |
| H | 6.88153735 | -13.15100757 | -1.47054857 |
| H | 5.43886586 | -12.74077910 | -2.43382202 |
| C | -5.97616832 | 12.54478454 | 1.50656480 |
| H | -6.88153735 | 13.15100757 | 1.47054857 |
| H | -6.23623104 | 11.48992365 | 1.42622615 |
| H | -5.43886586 | 12.74077910 | 2.43382202 |
| C | -4.81569747 | -14.42054914 | 0.46125381 |
| H | -5.76283257 | -14.96009587 | 0.51261942 |
| H | -4.24601219 | -14.75252371 | -0.40447403 |
| H | -4.23745290 | -14.57759571 | 1.37204898 |
| C | 4.81569747 | 14.42054914 | -0.46125381 |
| H | 5.76283257 | 14.96009587 | -0.51261942 |
| H | 4.23745290 | 14.57759571 | -1.37204898 |
| H | 4.24601219 | 14.75252371 | 0.40447403 |
| C | -5.97624932 | -12.54440594 | 1.50672335 |
| H | -5.43895325 | -12.74034842 | 2.43399578 |
| H | -6.23626896 | -11.48954161 | 1.42630058 |
| H | -6.88164193 | -13.15059604 | 1.47075954 |
| C | 5.97624932 | 12.54440594 | -1.50672335 |
| H | 6.23626896 | 11.48954161 | -1.42630058 |
| H | 5.43895325 | 12.74034842 | -2.43399578 |
| H | 6.88164193 | 13.15059604 | -1.47075954 |
| C | -4.81554242 | 14.42079674 | 0.46094822 |
| H | -4.23729320 | 14.57789620 | 1.37173135 |
| H | -4.24584467 | 14.75268123 | -0.40480555 |
| H | -5.76265759 | 14.96038212 | 0.51226847 |
| C | 4.81554242 | -14.42079674 | -0.46094822 |
| H | 4.24584467 | -14.75268123 | 0.40480555 |
| H | 4.23729320 | -14.57789620 | -1.37173135 |
| H | 5.76265759 | -14.96038212 | -0.51226847 |

DFT B3LYP/6-31+G(d, p), gas phase, $S_0$

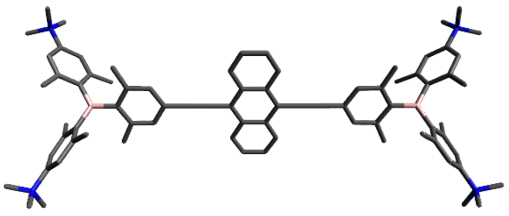

Point group: $C_i$

Total energy: –2,067,937.82

kcal mol$^{-1}$

Dipole moment: 0 D

Immaginary frequencies: 0

| | | | |
|---|---|---|---|
| C | 1.90216600 | -0.70838600 | 3.14808900 |
| C | 1.28939900 | -1.39953600 | 2.13227800 |
| C | 0.64174100 | -0.71899500 | 1.05834200 |
| C | 0.64140400 | 0.72020300 | 1.05772500 |
| C | 1.28874100 | 1.40196700 | 2.13107700 |
| C | 1.90183200 | 0.71197600 | 3.14748100 |
| C | 0.00033400 | -1.42844200 | 0.00061200 |
| C | -0.00033400 | 1.42844200 | -0.00061200 |
| C | -0.64174100 | 0.71899500 | -1.05834200 |
| C | -0.64140400 | -0.72020300 | -1.05772500 |
| C | -1.28874100 | -1.40196700 | -2.13107700 |
| H | -1.28631700 | -2.48590100 | -2.13139500 |
| C | -1.90183200 | -0.71197600 | -3.14748100 |
| C | -1.90216600 | 0.70838600 | -3.14808900 |
| C | -1.28939900 | 1.39953600 | -2.13227800 |
| H | 2.38788700 | -1.24604900 | 3.95653100 |
| H | 1.28748400 | -2.48347000 | 2.13352400 |
| H | 1.28631700 | 2.48590100 | 2.13139500 |
| H | 2.38730000 | 1.25056000 | 3.95546100 |
| H | -2.38730000 | -1.25056000 | -3.95546100 |
| H | -2.38788700 | 1.24604900 | -3.95653100 |
| H | -1.28748400 | 2.48347000 | -2.13352400 |

| | | | |
|---|---|---|---|
| C | 0.00066500 | -2.84678600 | 0.00122200 |
| C | 0.00094000 | -4.06861600 | 0.00173000 |
| C | 0.00122000 | -5.48861400 | 0.00223700 |
| C | -0.70666000 | -6.21637500 | -0.97388600 |
| C | 0.70939800 | -6.21539200 | 0.97887700 |
| C | -0.73485800 | -7.61019000 | -0.97440400 |
| H | -1.24578800 | -5.67126100 | -1.74214700 |
| C | 0.73814800 | -7.60919400 | 0.98040200 |
| H | 1.24832500 | -5.66951000 | 1.74673300 |
| C | 0.00178200 | -8.35274600 | 0.00327500 |
| C | -1.53728800 | -8.26841600 | -2.08259900 |
| C | 1.54086200 | -8.26631300 | 2.08904700 |
| B | 0.00206800 | -9.92096000 | 0.00386000 |
| H | -1.81682100 | -7.53022100 | -2.83803600 |
| H | -0.98092100 | -9.05828100 | -2.59576800 |
| H | -2.46224600 | -8.71581100 | -1.70504900 |
| H | 2.46594700 | -8.71369100 | 1.71179600 |
| H | 1.82018700 | -7.52745300 | 2.84391100 |
| H | 0.98477800 | -9.05597200 | 2.60284600 |
| C | -1.36283300 | -10.74012800 | -0.19296500 |
| C | 1.36726800 | -10.73947600 | 0.20131200 |
| C | -1.50168300 | -11.75019800 | -1.18399400 |
| C | -2.47771200 | -10.47306300 | 0.64325100 |
| C | 1.50647200 | -11.74877600 | 1.19307300 |
| C | 2.48208100 | -10.47255600 | -0.63503400 |
| C | -2.71187300 | -12.43898100 | -1.33352000 |
| C | -0.38027200 | -12.11294500 | -2.13572200 |
| C | -3.67470400 | -11.19621000 | 0.49230300 |
| C | -2.45893100 | -9.41495300 | 1.72892700 |
| C | 2.71692600 | -12.43697400 | 1.34314800 |
| C | 0.38515500 | -12.11129400 | 2.14500100 |
| C | 3.67935400 | -11.19511900 | -0.48351400 |
| C | 2.46292500 | -9.41521100 | -1.72144700 |
| C | -3.79119400 | -12.16505900 | -0.49592100 |
| H | -2.77983600 | -13.18735200 | -2.11576900 |
| H | 0.03853900 | -11.23177800 | -2.63012700 |
| H | 0.44091000 | -12.61126000 | -1.61210100 |
| H | -0.73198500 | -12.78699600 | -2.92057600 |
| H | -4.49083000 | -10.96177600 | 1.16257600 |
| H | -1.54393000 | -9.44188000 | 2.32514200 |
| H | -2.52657200 | -8.40966800 | 1.30166700 |
| H | -3.30073100 | -9.54424100 | 2.41397700 |
| C | 3.79617700 | -12.16322300 | 0.50540100 |
| H | 2.78515200 | -13.18475200 | 2.12594200 |
| H | -0.03421500 | -11.22992900 | 2.63857000 |
| H | -0.43568100 | -12.61054200 | 1.62172000 |
| H | 0.73715400 | -12.78445300 | 2.93049300 |
| H | 4.49542200 | -10.96083300 | -1.15390900 |
| H | 1.54795800 | -9.44292400 | -2.31767600 |
| H | 2.53014100 | -8.40960000 | -1.29488300 |
| H | 3.30480600 | -9.54463600 | -2.40637200 |
| C | -0.00066500 | 2.84678600 | -0.00122200 |
| C | -0.00094000 | 4.06861600 | -0.00173000 |
| C | -0.00122000 | 5.48861400 | -0.00223700 |
| C | -0.70939800 | 6.21539200 | -0.97887700 |
| C | 0.70666000 | 6.21637500 | 0.97388600 |
| C | -0.73814800 | 7.60919400 | -0.98040200 |
| H | -1.24832500 | 5.66951000 | -1.74673300 |
| C | 0.73485800 | 7.61019000 | 0.97440400 |
| H | 1.24578800 | 5.67126100 | 1.74214700 |
| C | -0.00178200 | 8.35274600 | -0.00327500 |
| C | -1.54086200 | 8.26631300 | -2.08904700 |
| C | 1.53728800 | 8.26841600 | 2.08259900 |
| B | -0.00206800 | 9.92096000 | -0.00386000 |
| H | -1.82018700 | 7.52745300 | -2.84391100 |
| H | -2.46594700 | 8.71369100 | -1.71179600 |
| H | -0.98477800 | 9.05597200 | -2.60284600 |
| H | 0.98092100 | 9.05828100 | 2.59576800 |
| H | 1.81682100 | 7.53022100 | 2.83803600 |
| H | 2.46224600 | 8.71581100 | 1.70504900 |
| C | 1.36283300 | 10.74012800 | 0.19296500 |
| C | -1.36726800 | 10.73947600 | -0.20131200 |
| C | 2.47771200 | 10.47306300 | -0.64325100 |
| C | 1.50168300 | 11.75019800 | 1.18399400 |
| C | -2.48208100 | 10.47255600 | 0.63503400 |
| C | -1.50647200 | 11.74877600 | -1.19307300 |
| C | 3.67470400 | 11.19621000 | -0.49230300 |

| | | | |
|---|---|---|---|
| C | 2.45893100 | 9.41495300 | -1.72892700 |
| C | 2.71187300 | 12.43898100 | 1.33352000 |
| C | 0.38027200 | 12.11294500 | 2.13572200 |
| C | -3.67935400 | 11.19511900 | 0.48351400 |
| C | -2.46292500 | 9.41521100 | 1.72144700 |
| C | -2.71692600 | 12.43697400 | -1.34314800 |
| C | -0.38515500 | 12.11129400 | -2.14500100 |
| C | 3.79119400 | 12.16505900 | 0.49592100 |
| H | 4.49083000 | 10.96177600 | -1.16257600 |
| H | 2.52657200 | 8.40966800 | -1.30166700 |
| H | 1.54393000 | 9.44188000 | -2.32514200 |
| H | 3.30073100 | 9.54424100 | -2.41397700 |
| H | 2.77983600 | 13.18735200 | 2.11576900 |
| H | -0.44091000 | 12.61126000 | 1.61210100 |
| H | -0.03853900 | 11.23177800 | 2.63012700 |
| H | 0.73198500 | 12.78699600 | 2.92057600 |
| C | -3.79617700 | 12.16322300 | -0.50540100 |
| H | -4.49542200 | 10.96083300 | 1.15390900 |
| H | -2.53014100 | 8.40960000 | 1.29488300 |
| H | -1.54795800 | 9.44292400 | 2.31767600 |
| H | -3.30480600 | 9.54463600 | 2.40637200 |
| H | -2.78515200 | 13.18475200 | -2.12594200 |
| H | 0.43568100 | 12.61054200 | -1.62172000 |
| H | 0.03421500 | 11.22992900 | -2.63857000 |
| H | -0.73715400 | 12.78445300 | -2.93049300 |
| N | 5.07686300 | 12.94093900 | 0.69670700 |
| N | -5.07686300 | -12.94093900 | -0.69670700 |
| N | -5.08214700 | 12.93844300 | -0.70680300 |
| N | 5.08214700 | -12.93844300 | 0.70680300 |
| C | 5.61766400 | -12.68810200 | 2.10577100 |
| H | 4.88911900 | -13.02790300 | 2.83834400 |
| H | 6.54995100 | -13.24159300 | 2.22409700 |
| H | 5.79110300 | -11.61853200 | 2.22086700 |
| C | -5.61766400 | 12.68810200 | -2.10577100 |
| H | -6.54995100 | 13.24159300 | -2.22409700 |
| H | -4.88911900 | 13.02790300 | -2.83834400 |
| H | -5.79110300 | 11.61853200 | -2.22086700 |
| C | 5.61253500 | 12.69182900 | 2.09583400 |
| H | 5.78638900 | 11.62241000 | 2.21170600 |
| H | 6.54461600 | 13.24576300 | 2.21371500 |
| H | 4.88389300 | 13.03188800 | 2.82819000 |
| C | -5.61253500 | -12.69182900 | -2.09583400 |
| H | -4.88389300 | -13.03188800 | -2.82819000 |
| H | -6.54461600 | -13.24576300 | -2.21371500 |
| H | -5.78638900 | -11.62241000 | -2.21170600 |
| C | 6.16632900 | -12.54464100 | -0.27028400 |
| H | 6.40590600 | -11.49026600 | -0.14110300 |
| H | 7.04581900 | -13.14958500 | -0.05162900 |
| H | 5.82894200 | -12.74309800 | -1.28649000 |
| C | -6.16632900 | 12.54464100 | 0.27028400 |
| H | -7.04581900 | 13.14958500 | 0.05162900 |
| H | -6.40590600 | 11.49026600 | 0.14110300 |
| H | -5.82894200 | 12.74309800 | 1.28649000 |
| C | -4.81750100 | -14.42557800 | -0.51342500 |
| H | -5.75769500 | -14.96129900 | -0.64953600 |
| H | -4.09098100 | -14.75983400 | -1.25054100 |
| H | -4.43001700 | -14.58502400 | 0.49245500 |
| C | 4.81750100 | 14.42557800 | 0.51342500 |
| H | 5.75769500 | 14.96129900 | 0.64953600 |
| H | 4.43001700 | 14.58502400 | -0.49245500 |
| H | 4.09098100 | 14.75983400 | 1.25054100 |
| C | -6.16116400 | -12.54686300 | 0.28013800 |
| H | -5.82365700 | -12.74444500 | 1.29647500 |
| H | -6.40117100 | -11.49267900 | 0.15019900 |
| H | -7.04041900 | -13.15232000 | 0.06195900 |
| C | 6.16116400 | 12.54686300 | -0.28013800 |
| H | 6.40117100 | 11.49267900 | -0.15019900 |
| H | 5.82365700 | 12.74444500 | -1.29647500 |
| H | 7.04041900 | 13.15232000 | -0.06195900 |
| C | -4.82338800 | 14.42331800 | -0.52458900 |
| H | -4.43600500 | 14.58364900 | 0.48119000 |
| H | -4.09697400 | 14.75733100 | -1.26191900 |
| H | -5.76379200 | 14.95856200 | -0.66112300 |
| C | 4.82338800 | -14.42331800 | 0.52458900 |
| H | 4.09697400 | -14.75733100 | 1.26191900 |
| H | 4.43600500 | -14.58364900 | -0.48119000 |
| H | 5.76379200 | -14.95856200 | 0.66112300 |

# References


[1] S. Griesbeck, Z. Zhang, M. Gutmann, T. Lühmann, R. M. Edkins, G. Clermont, A. N. Lazar, M. Haehnel, K. Edkins, A. Eichhorn, M. Blanchard-Desce, L. Meinel, T. B. Marder, *Chem. Eur. J.* **2016**, *22*, 14701-14706.

[2] H. Amini, Ž. Ban, M. Ferger, S. Lorenzen, F. Rauch, A. Friedrich, I. Crnolatac, A. Kenđel, S. Miljanić, I. Piantanida, T. B. Marder, *Chem. Eur. J.* **2020**, *26*, 6017-6028.

[3] S. Griesbeck, M. Ferger, C. Czernetzi, C. Wang, R. Bertermann, A. Friedrich, M. Haehnel, D. Sieh, M. Taki, S. Yamaguchi, T. B. Marder, *Chem. Eur. J.* **2019**, *25*, 7679-7688.

[4] M. Lübtow, I. Helmers, V. Stepanenko, R. Q. Albuquerque, T. B. Marder, G. Fernández, *Chem. Eur. J.* **2017**, *23*, 6198-6205.

[5] N. Miyaura, A. Suzuki, *Org. Synth.* **1990**, *68*, 130.

[6] S.-F. Liu, Q. Wu, H. L. Schmider, H. Aziz, N.-X. Hu, Z. Popović, S. Wang, *J. Am. Chem. Soc.* **2000**, *122*, 3671-3678.

[7] G. Sheldrick, *Acta Crystalogr.* **2015**, *A71*, 3-8.

[8] G. Sheldrick, *Acta Crystalogr.* **2008**, *A64*, 112-122.

[9] C. B. Hübschle, G. M. Sheldrick, B. Dittrich, *J. Appl. Crystallogr.* **2011**, *44*, 1281-1284.

[10] K. Brandenburg, Diamond (version 4.4.0) - Crystal and Molecular Structure Visualization, Crystal Impact H. Putz & K. Brandenburg GbR, Bonn (Germany), **2017**.

[11] J. B. Chaires, N. Dattagupta, D. M. Crothers, *Biochemistry* **1982**, *21*, 3933-3940.

[12] L.-M. Tumir, I. Piantanida, I. J. Cindrić, T. Hrenar, Z. Meić, M. Žinić, *J. Phys. Org. Chem.* **2003**, *16*, 891-899.

[13] J.-L. Mergny, L. Lacroix, *Oligonucleotides* **2003**, *13*, 515-537.

[14] G. Scatchard, *Ann. N. Y. Acad. Sci.* **1949**, *51*, 660-672.

[15] J. D. McGhee, P. H. von Hippel, *J. Mol. Biol.* **1974**, *86*, 469-489.

[16] D. A. Case, R. M. Betz, D. S. Cerutti, T. E. Cheatham, T. Darden, R. E. Duke, T. J. Giese, H. Gohlke, A. W. Goetz, N. Homeyer, S. Izadi, P. Janowski, J. Kaus, A. Kovalenko, T. S. Lee, S. LeGrand, P. Li, C. Lin, T. Luchko, R. Luo, B. Madej, D. Mermelstein, K. M. Merz, G. Monard, H. Nguyen, H. T. Nguyen, I. Omelyan, A. Onufriev, D. R. Roe, A. Roitberg, C. Sagui, C. Simmerling, W. M. Botello-Smith, J. Swails, R. C. Walker, J. Wang, R. M. Wolf, X. Wu, L. Xiao, P. A. Kollman, AMBER16, University of California, San Francisco, **2016**.

[17] J. Wang, R. M. Wolf, J. W. Caldwell, P. Kollman, *J. Comput. Chem.* **2004**, *25*, 1157-1174.

[18] M. Zgarbová, M. Otyepka, J. Šponer, A. Mládek, P. Banáš, T. E. Cheatham, P. Jurečka, *J. Chem. Theory Comput.* **2011**, *7*, 2886-2902.

[19] Ž. Ban, S. Griesbeck, S. Tomić, J. Nitsch, T. B. Marder, I. Piantanida, *Chem. Eur. J.* **2020**, *26*, 2195-2203.

[20] W. L. Jorgensen, J. Chandrasekhar, J. D. Madura, R. W. Impey, M. L. Klein, *J. Chem. Phys.* **1983**, *79*, 926-935.

[21] I. S. Joung, T. E. Cheatham, *J. Phys. Chem. B* **2008**, *112*, 9020-9041.

[22] R. J. Loncharich, B. R. Brooks, R. W. Pastor, *Biopolymers* **1992**, *32*, 523-535.

[23] H. J. C. Berendsen, J. P. M. Postma, W. F. v. Gunsteren, A. DiNola, J. R. Haak, *J. Chem. Phys.* **1984**, *81*, 3684-3690.

[24] M. J. Frisch, G. W. Trucks, H. B. Schlegel, G. E. Scuseria, M. A. Robb, J. R. Cheeseman, G. Scalmani, V. Barone, B. Mennucci, G. A. Petersson, H. Nakatsuji, M. Caricato, X. Li, H. P. Hratchian, A. F. Izmaylov, J. Bloino, G. Zheng, J. L. Sonnenberg, M. Hada, M. Ehara, K. Toyota, R. Fukuda, J. Hasegawa, M. Ishida, T. Nakajima, Y. Honda, O. Kitao, H. Nakai, T. Vreven, J. A. Montgomery, J. E. Peralta, F. Ogliaro, M. Bearpark, J. J. Heyd, E. Brothers, K. N. Kudin, V. N. Staroverov, R. Kobayashi, J. Normand, K. Raghavachari, A. Rendell, J. C. Burant, S. S. Iyengar, J. Tomasi, M. Cossi, N. Rega, J. M. Millam, M. Klene, J. E. Knox, J. B. Cross, V. Bakken, C. Adamo, J. Jaramillo, R. Gomperts, R. E. Stratmann, O. Yazyev, A. J. Austin, R. Cammi, C. Pomelli, J. W. Ochterski, R. L. Martin, K. Morokuma, V. G. Zakrzewski, G. A. Voth, P. Salvador, J. J. Dannenberg, S. Dapprich, A. D. Daniels, Farkas, J. B. Foresman, J. V. Ortiz, J. Cioslowski, D. J. Fox, Revision A.03 ed., Gaussian Inc., Wallingford CT, **2016**.

[25] M. D. Hanwell, D. E. Curtis, D. C. Lonie, T. Vandermeersch, E. Zurek, G. R. Hutchison, *J. Cheminformatics* **2012**, *4*, 17.

[26] T. Lu, F. Chen, *J. Comput. Chem.* **2012**, *33*, 580-592.

[27] C. Lee, W. Yang, R. G. Parr, *Phys. Rev. B* **1988**, *37*, 785-789.

[28] G. A. Petersson, A. Bennett, T. G. Tensfeldt, M. A. Al-Laham, W. A. Shirley, J. Mantzaris, *J. Chem. Phys.* **1988**, *89*, 2193-2218.

[29] G. A. Petersson, M. A. Al-Laham, *J. Chem. Phys.* **1991**, *94*, 6081-6090.

[30] W. Saenger, *Principles of nucleic acid structure*, Springer Science & Business Media, New York, **2013**.

[31] C. Cantor, P. Schimmel, in *Biophysical Chemistry Part III: The Behaviour of Biological Macromolecules*, Freeman and Company, Oxford, **1980**, pp. 1109-1181.
